# Significance of HLA in the development of Graves’ orbitopathy

**DOI:** 10.1038/s41435-023-00193-z

**Published:** 2023-01-13

**Authors:** Magdalena Stasiak, Katarzyna Zawadzka-Starczewska, Bogusław Tymoniuk, Bartłomiej Stasiak, Andrzej Lewiński

**Affiliations:** 1 Polish Mother’s Memorial Hospital - Research Institute, Department of Endocrinology and Metabolic Diseases, Lodz, Poland; 2 Medical University of Lodz, Department of Immunology, Rheumatology and Allergy, Lodz, Poland; 3 Lodz University of Technology, Institute of Information Technology, Lodz, Poland; 4 Medical University of Lodz, Department of Endocrinology and Metabolic Diseases, Lodz, Poland

**Keywords:** Next-generation sequencing, Autoimmunity

## Abstract

Graves’ disease (GD), similarly to most autoimmune disease, is triggered by environmental factors in genetically predisposed individuals. Particular HLA alleles increase or decrease GD risk. No such correlation was demonstrated for Graves’ orbitopathy (GO) in Caucasian population. *HLA-A, -B, -C, -DQB1* and *-DRB1* genotyping was performed using a high-resolution method in a total number of 2378 persons including 70 patients with GO, 91 patients with non-GO GD and 2217 healthy controls to compare allele frequencies between GO, non-GO and controls. Significant associations between GO and HLA profile were demonstrated, with HLA-A*01:01, -A*32:01, -B*37:01, -B*39:01, -B*42:01, -C*08:02, C*03:02, DRB1*03:01, DRB1*14:01 and DQB1*02:01 being genetic markers of increased risk of GO, and HLA-C*04:01, -C*03:04, -C*07:02 and -DRB1*15:02 being protective alleles. Moreover, correlations between HLA alleles and increased or decreased risk of non-GO GD, but with no impact on risk of GO development, were revealed. Identification of these groups of GO-related and GO-protective alleles, as well as the alleles strongly related to non-GO GD, constitutes an important step in a development of personalized medicine, with individual risk assessment and patient-tailored treatment.

## Introduction

Graves’ disease (GD) is an autoimmune thyroid disorder caused by production of specific antibodies against the thyrotropin (TSH) receptor (TRAb) [1] leading mainly to hyperthyroidism. The prevalence of GD in the Caucasian population is about 0.5–2.0% [1, 2]. Graves’ orbitopathy (GO) is the major extrathyroidal manifestation of GD, occurring more frequently in women, with the estimated incidence of 0.54–0.9 cases/100 000/year in men and 2.67–3.3 cases/100 000/year in women [3]. GO has significant impact on quality of life (QoL) and may even constitute a sight-threatening condition [3]. The knowledge on risk factors which are associated with GO occurrence seems crucial for the prevention and management of GO. Smoking, severe/unstable hyperthyroidism as well as high serum TRAb levels are well-known risk factors of GO development and progression [3]. However, it seems clear that these factors act on some specific genetic background which is pivotal for the disease development. Autoimmune diseases, including GD, are typically triggered by environmental factors in genetically predisposed individuals [2, 4]. Among genes associated with the immune response, human leukocyte antigen (HLA) genes seem to play a prominent role as a molecular background of GD [5, 6]. Many different HLA alleles were postulated as GD risk factors. However until recently, the data regarding Caucasian population were not consistent, most probably due to small study groups as well as different methods applied by the authors, including low resolution or serological methods [5]. We have previously demonstrated that application of next generation sequencing (NGS) methods allowed to reveal actual HLA-related susceptibility for subacute thyroiditis, which appeared to include much more alleles than just HLA-B*35, influencing the disease course [7–11]. Recently, our research team applied the same NGS methods to demonstrate that GD is strongly HLA-dependent in Caucasian population. We have proved significant association between the risk of GD and the presence of the following alleles: HLA-B*08:01, -B*39:06, -B*37:01, -C*07:01, -C*14:02, -C*03:02, -C*17:01, -DRB1*03:01, -DRB1*11:01, -DRB1*13:03, -DRB1*01:03, -DRB1*14:01, -DQB1*03:01, DQB1*02:01. We have also demonstrated the protective role of HLA-B*07:02, -C*07:02, -C*03:04, DRB1*07:01, -DQB1*02:02, -DQB1*03:03 [5].

There is currently no clear HLA-related susceptibility for GO confirmed in Caucasian population. Previously published studies on the relationship between HLA alleles and the occurrence of GO concerned almost exclusively Asian population and revealed highly inconsistent results. Using serological method in Japanese cohort, Inoue et al. observed that GO was associated with the following three HLA pairs: HLA-DQw4 without presence of -A31, HLA-A11 without presence of -DPw2, and a co-presence of HLA-B5 and -Dw12 [12]. However, in a study published a year earlier, Inoue et al. found only DQw3 as a GO risk factor [13]. Ohtsuka and Nakamura used the same methods, also in Japanese population, and obtained completely different results [14]. They postulated that HLA-DR14 and DQ1 antigens may be genetic markers of predisposition to severe GO, while HLA-B35, B54, DR4, and DQ4 may play protective role [14]. In 2017 Mehraji et al. reported lack of any differences in allelic distribution between GO and non-GO patients in Iranian cohort [15]. On the other hand, in Korean study, HLA-C*03:03 was found more frequently in GO as compared to non-GO patients [6]. In a very recent Chinese study, the HLA alleles including HLA-B*38:02, -DRB1*16:02, -DQA1*01:02 and -DQB1*05:02 were postulated as risk factors for GO [16]. The results of the previous studies were summarized in Table 1. Taking into account the inconsistency of the results, unambiguous conclusions are not possible to be drawn even in Asian population.

**Table 1:** Summary of the results of previous studies.

Authors	Ref.	Population	Method	No. of GO patients	No. of GD patients	No. of healthy controls	Result - HLA related to GO risk
Inoue et al.	[12]	Asian (Japanese)	serological	42	88	186	-DQw4 (+) and -A31 (-) -B5 ( + ) and -Dw12 (+) -A11 ( + ) and -DPw2 (+)
Inoue et al.	[13]	Asian (Japanese)	serological	23	88	186	DQw3 (+), DPQ2( + )
Ohtsuka and Nakamura	[14]	Asian (Japanese)	serological	48	94	767	-DR14 ( + ), DQ1 ( + ) -B35 (-), B54 (-), DR4 (-), DQ4 (-)
Mehraji et al.	[15]	Asian (Iranian)	SSP-PCR	45	80	180	none
Shin et al.	[16]	Asian (Korean)	NGS	35	71	142	-C*03:03 (+) -B*54:01 (-)
Huang et al.	[17]	Asian (Chinese)	NGS	82	272	411	-B*38:02 (+) -DQA1*01:02 + -DRB1*16:02 (+) -DQA1*01:02 + -DQB1*05:02 (+)

NGS – next generation sequencing.

SSP – single specific primer - polymerase chain reaction (low to intermediate resolution method).

There are almost no consistent reports indicating the relationship between HLA and GO in the Caucasian population. Yin et al. did not demonstrate the existence of HLA-related susceptibility to GO in the group of patients with GD and postulated the importance of environmental or epigenetic factors only [17]. However, the authors of that study focused only on the frequency of *HLA-DR3*, without assessing the frequencies of other alleles.

The purpose of our study was to perform HLA genotyping using the NGS method in Caucasians, to find out which alleles are eventually correlated with high risk of GO, as well as which of them can be considered protective. Identification of a group of GO-related and GO-protective HLA alleles would constitute a great step in a development of personalized medicine as it would provide a new precise diagnostic tool for the individual risk assessment.

## Subjects and methods

### GD group and control group

A total number of 2378 persons were included into the study, with 2217 healthy Polish hematopoietic stem cell potential donors who did not have any medical history of thyroid disease or orbitopathy (control group), and 161 unrelated patients with GD diagnosed in the Department of Endocrinology and Metabolic Diseases, Polish Mother’s Memorial Hospital-Research Institute, as well as in the Department-associated outpatient clinic. The GD group included 70 patients with GO (GO group) and 91 patients without GO (non-GO group). The large size of the control group was required to avoid any bias associated with potential diseases which might occur in currently healthy members of this group in future, and – additionally – to avoid any bias related to random changes (increase or decrease) in frequencies of some alleles in a smaller control group.

### Inclusion criteria

In all patients included into the GD study group, the diagnosis of GD was made on the basis of standard criteria [1], including hyperthyroidism, elevated TRAb level, as well as typical ultrasound (US) pattern. The diagnosis of GO, as well as the assessment of GO activity and severity, was performed on the basis of the EUGOGO guidelines actual at the time of diagnosis, i.e. 2021 version [3] or 2016 version [18] the latter version used for patients diagnosed before the time when 2021 version was available. Patients with other diseases which may have influenced the obtained results were excluded from the study, except for two patients with latent autoimmune diabetes in adults (LADA), who were not excluded from the GO group as no potential error related to their HLA results was expected. LADA is associated with the presence of HLA-DRB1*03 and -DQB1*02:01 as well as HLA-DRB1*04 and -DQB1*0302 [19]. Alleles HLA-DRB1*04 and -DQB1*03:02 were not found as more frequent in our GO group, while HLA-DRB1*03:01 is a well-known marker of many autoimmune diseases, and it is in linkage disequilibrium with DQB1*02:01. Therefore, taking into account the lack of any potential bias caused by LADA in these two patients, they were not excluded from the study.

### Diagnostic procedures

Serum levels of TSH, free thyroxine (FT4), free triiodothyronine (FT3) and TRAb were measured by the electrochemiluminescence immunoassay (ECLIA) using Cobas e601 analyzer (Roche Diagnostics, Indianapolis, IN, USA). In all patients, ultrasound examinations (US) were performed using a 7–14 MHz linear transducer (Toshiba Aplio XG; Toshiba, Japan). In all GO patients, magnetic resonance imaging (MRI) was performed to unambiguously confirm GO diagnosis and to exclude any other orbital or intracranial pathological process.

### HLA typing procedures

DNA was isolated from whole blood samples collected to the anticoagulant (EDTA)-containing tubes. *HLA-A, -B, -C, -DQB1* and *-DRB1* genotyping was performed using a standard high-resolution NGS method [20] with application of MIA FORA NGS FLEX 5 HT HLA Typing Kit [21] (Immucor Transplant Diagnostics, Inc. 35 Technology Drive South Warren, New Jersey 07059, USA) that supplies reagents for up to 1152 samples. However, during one run of high-resolution typing we performed genotyping of 576 samples. The MIA FORA NGS FLEX 5 HT HLA typing protocol uses long-range PCR to capture the clinically relevant Class I and II HLA genes. The core kit includes each of the Class I genes, *HLA-A, HLA -B*, and *HLA -C*, as well as the Class II genes, *HLA-DRB1* and *HLA-DQB1. HLA-A, HLA-B*, and *HLA-C* are sequenced in their entirety. We performed sample preparation divided into three distinct sections: long-range PCR, library preparation, and sequencing. During the first section we prepared six PCR mixes per sample. Each gene was amplified as one large piece in its entirety, except for DRB, which was amplified as two overlapping segments due to its large size. Within the MIA FORA system, these are referred to as DRB-S and DRB-L. Following gene amplification amplicons were quantitated by fluorescence detection using PicoGreen™ reagent and a fluorescent plate reader. The PCR products per sample were balanced and pooled before proceeding with enzymatic fragmentation, end repair, A-tailing, and cleaned with magnetic beads. Index adaptor ligation: each kit contains two sets of six individual index adaptor plates, with 96 adaptors per plate. These index adaptors contain index sequences (barcodes) and Illumina-compatible adaptor sequences that allow for sequencing in a multiplex format. Index Adaptors from identically-named Index Adaptor Plates cannot be combined into the same library. Each 96-well sample plate was consolidated into a single microcentrifuge tube and size-selected with the Pippin Prep before final PCR amplification. The library was quantitated by Qubit and concentration was adjusted according to the Illumina NextSeq library preparation protocol. This protocol describes semi-automated sample processing for high throughput sequencing, from long range PCR through library preparation, prior to sequencing on an Illumina instrument (Illumina 5200 Illumina Way San Diego, California 92122 U.S.A). Genomic library was cleaned with magnetic beads and denatured by 0.2 N NaOH before loading on NGS Illumina Platform. All automated sample processing was performed on the Biomek i7 Liquid Handler. Sequencing data were analyzed by MiaFora NGS software v. 4.5, IPD-IMGT/HLA database version 3.40. The data were considered sufficient whenever the coverage reached 40. We used advanced NGS HLA Genotyping Software MIA FOR A, a trademark owned by Sirona Genomics, Inc. Genotypes were computed from massive, paired-end sequencing reads derived from the Illumina Next Generation Sequencing (NGS) platform. The results of HLA-typing are available as Supplementary Materials.

### Statistical analysis

Statistical calculations were performed for all alleles in all loci regardless of the frequency of their occurrence in the population. Regardless of whether patients or control group individuals were homozygous or heterozygous, each of them was counted once only. To make the results more readable, we presented results only for these alleles for which the statistically significant differences were achieved. Allele frequencies were reported in percentages. The statistical significance of the differences between groups was evaluated by the chi-square test and by binomial logistic regression analysis, with *p* values ≤ 0.05 considered significant. For small groups, the statistical significance of the differences between the groups was evaluated by Fisher exact test with *p* values ≤ 0.05 considered significant. Odds ratio (OR) was calculated for all comparisons in which a given allele was present in both of the compared groups. The statistical analysis was carried out using Statistica v 13 software (Statsoft Polska, Kraków, Poland).

### Ethics procedures

All patients gave their informed consent for all procedures performed during the study. The consents were obtained after full explanation of the purpose and nature of all the procedures used in the study. The study was approved by the Ethics Committee of the Polish Mother’s Memorial Hospital—Research Institute, Lodz, Poland (approval code—108/2018).

## Results

The mean age of the patients at the time of diagnosis of GD was 43.63 ± 17.59 years, with a male to female ratio of 1:4.75. Statistically significant differences in the frequency of HLA alleles between patients within GO and non-GO groups as well as between either of them and the control group were found, with several alleles of higher frequency and others of lower frequency either in GO or non-GO group. The deviation of genotypic frequencies from the Hardy-Weinberg Equilibrium at each HLA locus was analyzed for the control group. The *p* value results from GENEPOP vs. 4.7.5: Hardy-Weinberg test were as follows: *HLA-A* – 0.4428, *HLA-B* – 0.9006, *HLA-C* – 0.9482, *HLA-DRB1*–0.5317, *HLA-DQB1*–0.3989.

### Comparison of GO and non-GO groups

**Fig. 1:**
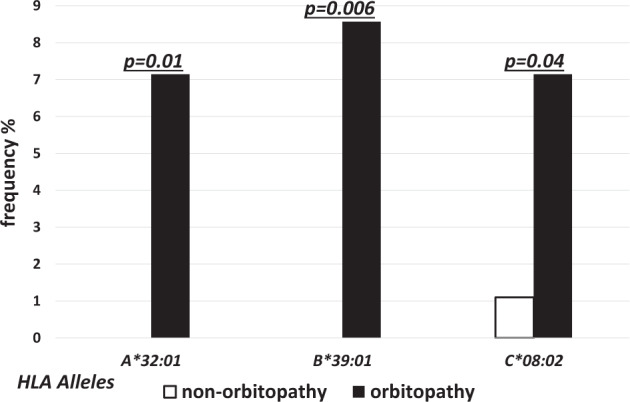
Frequencies (%) of human leukocyte antigen (HLA) over-represented alleles with statistically significant difference between non-GO (open bars) and GO patients (solid bars).

**Fig. 2:**
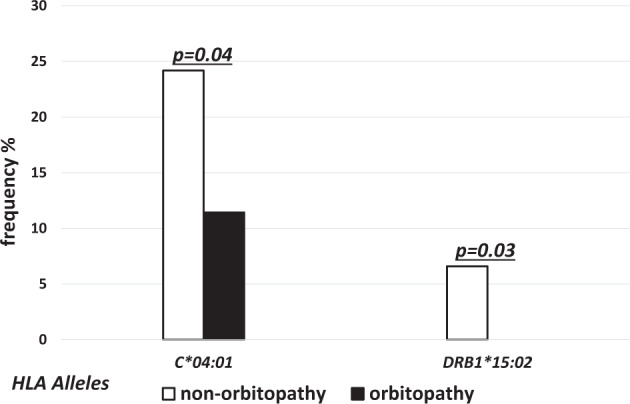
Frequencies (%) of human leukocyte antigen (HLA) under-represented alleles with statistically significant difference between non-GO (open bars) and GO patients (solid bars).

The alleles of higher frequency in GO as compared to non-GO group were found in MHC class I only. The differences were statistically significant for HLA-A*32:01 (7.14% vs 0.0%, *p* = 0.01), -B*39:01 (8.57% vs. 0.0%, *p* = 0.006) and -C*08:02 (7.14% vs. 1.0%, *p* = 0.04, OR 6.9) (Fig. 1). On the other hand, the frequency of HLA-C*04:01 and DRB1*15:02 was significantly lower in GO as compared to non-GO group (11.43% vs. 24.18%, *p* = 0.04, OR 0.4,and 0.0% vs. 6.59%, *p* = 0.03, respectively) (Fig. 2).

### Comparison of GO and control group

**Fig. 3:**
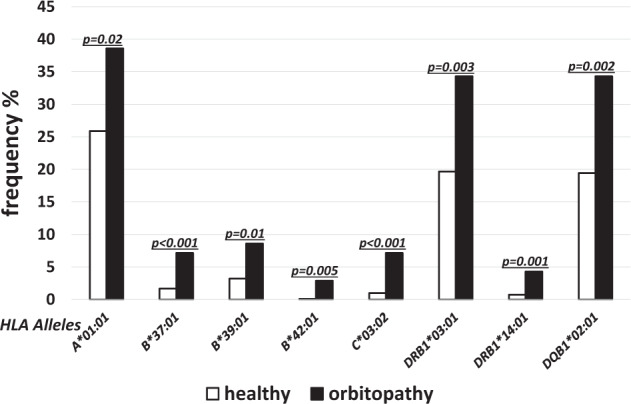
Frequencies (%) of human leukocyte antigen (HLA) over-represented alleles with statistically significant difference between healthy (open bars) and GO patients (solid bars).

The alleles of higher frequency in GO as compared to the controls were found in both MHC class I and class II. The differences were statistically significant for the following alleles of MHC class I: HLA-A*01:01(38.54% vs. 25.89%, *p* = 0.02, OR 1.8), -B*37:01 (7.14% vs. 1.67%, *p* < 0.001, OR 4.5), -B*39:01 (8.57% vs. 3.20%, *p* = 0.01, OR 2.8), -B*42:01 (2.86% vs. 0.09%, *p* = 0.005, OR 2.8) and -C*03:02 (7.14% vs. 0.99%, *p* < 0.001, OR 8.3) (Fig. 3). For the MHC class II, the frequencies of the following alleles were higher in GO as compared to the controls: HLA-DRB1*03:01 (34.29% vs. 19.67%, *p* = 0.003, OR 1.9), -DRB1*14:01 (4.29% vs.0.72%, *p* = 0.001, OR 6.2), DQB1*02:01 (34.29% vs. 19.44%, *p* = 0.002, OR 1.9) (Fig. 3).

**Fig. 4:**
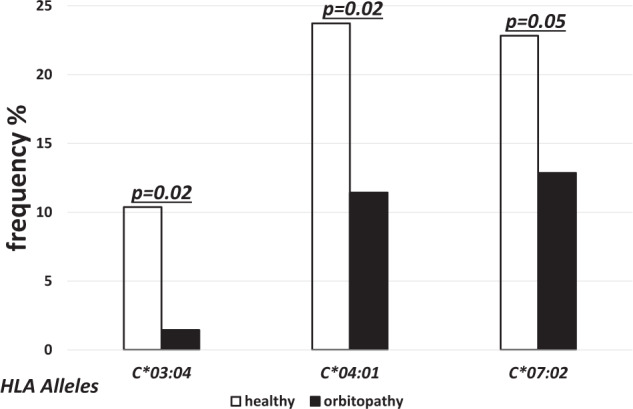
Frequencies (%) of human leukocyte antigen (HLA) under-represented alleles with statistically significant difference between healthy (open bars) and GO patients (solid bars).

On the other hand, the frequencies of HLA-C*04:01, -C*03:04 and -C*07:02 were significantly lower in GO as compared to the controls (11.43% vs. 23.73%, *p* = 0.02, OR 0.4; 1.43% vs. 10.37%, *p* = 0.02, OR 0.1; and 12.86% vs. 22.92%, *p* = 0.05, OR 0.2, respectively) (Fig. 4).

### Comparison of non-GO and control group

**Fig. 5:**
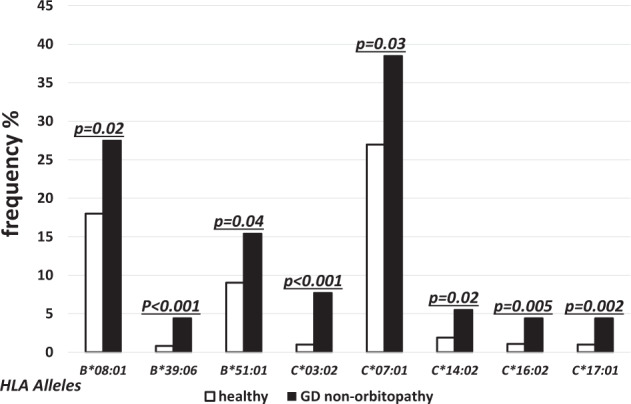
Frequencies (%) of human leukocyte antigen (HLA) over-represented alleles with statistically significant difference between healthy (open bars) and non-GO patients (solid bars) for major histocompatibility complex (MHC) class I alleles.

**Fig. 6:**
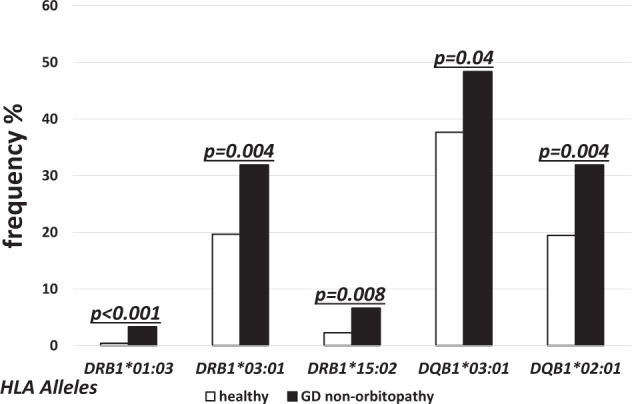
Frequencies (%) of human leukocyte antigen (HLA) over-represented alleles with statistically significant difference between healthy (open bars) and non-GO patients (solid bars) for major histocompatibility complex (MHC) class II alleles.

The alleles of higher frequency in non-GO as compared to the controls were found in both MHC class I and class II. The differences were statistically significant for the following alleles of MHC class I: HLA-B*08:01 (27.47% vs. 18.0%, *p* = 0.02, OR 1.7), -B*39:06 (4.40% vs. 0.81%, *p* < 0.001, OR 5.6), -B*51:01 (15.38% vs. 9.02%, *p* = 0.04, OR 1.8) (Fig. 5), HLA-C*03:02 (7.69% vs. 0.99%, *p* < 0.001, OR 7.7), -C*07:01 (38.46% vs. 26.97%, *p* = 0.03, OR 1.6), -C*14:02 (5.49% vs. 1.89%, *p* = 0.02, OR 3.0), -C*16:02 (4.40% vs. 1,08%, *p* = 0.005, OR 4.2), -C*17:01(4.40% vs. 0.99%, *p* = 0.002, OR 4.6) (Fig. 5). For the MHC class II, the frequencies of the following alleles were higher in non-GO as compared to the controls: HLA-DRB1*01:03 (3.30% vs. 0.41%, *p* < 0.001, OR 8.4), -DRB1*03:01 (31.87% vs. 19.44 %, *p* = 0.004, OR2.1), -DRB1*15:02 (6.59% vs. 2.26%, *p* = 0.008, OR 3.1),, -DQB1*03:01 (48.35% vs. 37.66%, *p* = 0.04, OR 1.5), -DQB1*02:01 (31.87% vs. 19.44%, *p* = 0.004, OR 2.2) (Fig. 6). No age-related difference in high risk allele frequency was found (data not presented). Comparison of high risk allele frequencies between males and females was not performed because of the incomparable sizes of males and females subgroups.

**Fig. 7:**
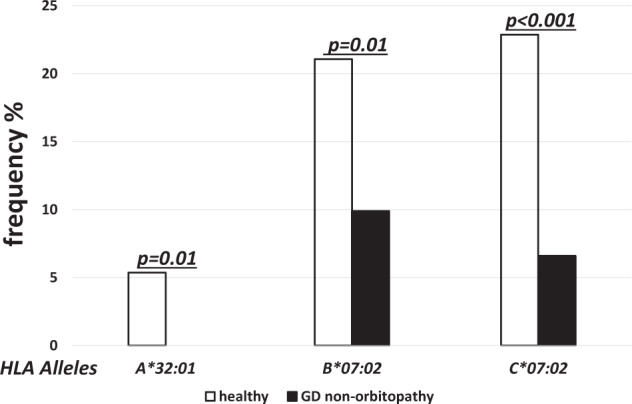
Frequencies (%) of human leukocyte antigen (HLA) under-represented alleles with statistically significant difference between healthy (open bars) and non-GO patients (solid bars).

On the other hand, the frequencies of HLA-A*32:01, -B*07:02 and -C*07:02 were significantly lower in non-GO as compared to the controls (0.0% vs. 5.37%, *p* = 0.01; 9.89% vs. 21.06%, *p* = 0.01, OR 0.4; and 6.59% vs. 22.87%, *p* < 0.001, OR 0.5, respectively) (Fig. 7).

The summary of the relationships between HLA and GO development as well as between HLA and non-GO GD are presented in Table 2.

**Table 2:** Summary of relationships between HLA and GO and non-GO GD development.

Increased risk of GO	*OR*	Increased risk of GO and non-GO^2^	*OR, OR*^*2*^	Increased risk of non-GO but not GO^2^	*OR*	Decreased risk of GO	*OR*	Decreased risk of non-GO^2^	*OR*
A*32:01^***a***^	*-*	C*03:02	*8.3; 7.7*^*b*^	B*08:01	*1.7*	C*04:01^***a,b***^	*0.4*^***a,b***^	B*07:02	*0.4*
B*39:01^***a,b***^	***2.8***^***b***^	DRB1*03:01	*1.9; 2.1*^*b*^	B*39:06	*5.6*	DRB1*15:02^***a***^	^***-***^	C*07:02	*0.5*
C*08:02^***a***^	***6.9***	DQB1*02:01	*1.9; 2.2*^*b*^	B*51:01	***1.8***	C*03:04^*b*^	*0.1*	A*32:01	*-*
A*01:01^***b***^	***1.8***			C*07:01	*1.6*	C*07:02^*b*^	*0.2*		
B*37:01^*b*^	*4.5*			C*14:02	*3.0*				
B*42:01^***b***^	***2.8***			C*16:02	***4.2***				
DRB1*14:01^*b*^	*6.2*			C*17:01	*4.6*				
				DRB1*01:03	*8.4*				
				DRB1*15:02	***3.1***				
				DQB1*03:01	*1.5*				

^a^vs non-GO; ^b^vs healthy controls, alleles not previously reported as GD related unless GO and non-GO groups were distinguished are presented in bold.

*GO* Graves’ orbitopathy, *OR* odds ratio.

### Frequencies of a single high risk allele and of co-presence of alleles

In 15 patients with GO (21.43%), only one of the alleles described above as correlated to a high risk of GO was found. These alleles were HLA-A*01:01, -A*32:01, -B*39:01, -C*03:02, -C*08:02 with HLA-A*01:01 and -B*39:01 being the most commonly present. Each of them occurred as a single high risk allele in 4 patients with GO (5.7%).

In 11 patients (15.71%), two of the high-risk alleles were present. Among this group, the co-presence of HLA-DRB1*03:01 and -DQB1*02:01 was observed the most frequently (27.27%) and these two alleles are in linkage disequilibrium (LD) [22]. The co-presence of different alleles which are not in LD was observed in the rest of patients among this group. Interestingly, among the group of patients with three high risk alleles, HLA-A*01:01 was most frequently present with -DRB1*03:01 and -DQB1*02:01. A combination of these three alleles – HLA-A*01:01, -DRB1*03:01- and DQB1*02:01 – occurred in 11 out of 70 GO patients (15.7%), while the co-presence of other sets of three alleles was found only in 5.7% of GO patients. Among the group of patients with four risk alleles, only patients with the most common combination of three alleles (i.e. HLA-A*01:01, -DRB1*03:01- and DQB1*02:01) with additional presence of HLA-A*32:01, -B*37:01 or -DRB1*14:01 were found. None of the patients had more than four high risk alleles.

## Discussion

In the last decades, it has become more and more clear that autoimmune diseases are triggered by environmental factors such as infections, stress, smoking, etc. in genetically predisposed individuals [5, 23]. This genetic susceptibility seems to be crucial also in the pathogenesis of GD. Very recently, our research group has demonstrated the complex correlation between HLA alleles and GD development [5]. Those results clarified the previously existing discrepancies between different reports available for Caucasian population. Significant divergences in the results presented by various authors could undoubtedly depend on the applied method and the size of the study group. We previously confirmed in patients with GD as well as with SAT, that the use of high-resolution methods can significantly change the results obtained with less specific older methods. Application of modern methods of genotyping, which allow to achieve allelic specificity, is currently a gold standard of research because these methods demonstrate high reliability and allow to avoid method-dependent errors. Less specific methods obtain results for the entire allelic group, not for a particular allele and – therefore – may lead to erroneous conclusions. In a strictly controlled group of HLA typing performed for the purposes of bone marrow transplantation between 1996 and 2011, discrepancies between results obtained with older methods and the NGS method were found in as many as 29.1% of cases [24]. Therefore, the results of our recent study may be considered highly reliable, as it included the largest Caucasian cohort to whom a modern high-resolution method was applied up to date [5].

Having identified the alleles related to high risk of GD and the protective ones [5], we made an attempt to fill the knowledge gap regarding HLA background of GO in Caucasian population. As it was stated above, the data on this issue are available almost exclusively for Asians but even in that population the results are so divergent that no clear conclusion is possible to be drawn. In Caucasians, Yin et al. postulated lack of any genetic susceptibility to GO and concluded that environmental and epigenetic factors played crucial role in GO development [17]. However, taking into account the fact that some GO patients are practically free from environmental and biochemical risk factors (no smoking history, slightly elevated TRAb and thyroid hormone levels) the importance of genetic factors seems pivotal. Recently, several reports on significance of various gene polymorphisms in GO development in Caucasians have been published [25–28]. Additionally, the impact of *CD28/CTLA-4/ICOS* haplotypes on susceptibility to GD and GO was also postulated [29]. These results strongly support the significance of genetic background of GO.

Our present results further confirmed the role of genetic background in GO development by demonstrating the significance of HLA for GO risk in Caucasians, with the application of NGS method. As it was stated above, other results on HLA-related susceptibility for GO are lacking in Caucasians, so our results cannot be directly compared to other studies, especially those which used the same method. Contrary to Yin et al. [17], we have confirmed a strong correlation between GO and HLA, including identification of both high-risk and protective alleles.

In the present study, we have demonstrated that HLA-A*32:01, -B*39:01, -C*08:02, -A*01:01, -B*37:01, B*42:01 and DRB1*14:01 are associated with increased risk of GO while they are not associated with non-GO GD course. On the basis of OR obtained for our study the highest risk of GO was associated with the presence of HLA-C*08:02 (OR 6.9) and -B*37:01 (OR 4.5). This is a very important finding, especially considering the fact that HLA-A*32:01, -B*39:01, -C*08:02 alleles were strongly GO-related as compared to non-GO group. HLA-A*01:01 is a very common allele in Caucasian population, therefore the difference between GO and non-GO was not statistically significant, however the significance was clear when GO group was compared to the healthy controls. There is no LD between these three alleles [30, 31], so the presence of any of them constitutes independent high risk factor. It should be stressed that HLA-DRB1*14:01 was previously postulated as GO-related in Japanese patients [14] (Table 1) and this is the only similarity between our results in Caucasians and currently published data for the Asian population. However, such a lack of consistency could be expected, as not only were the results in Asians highly divergent, but also HLA susceptibility for autoimmune diseases including GD often differs between the two populations [6, 12–16]. We have previously demonstrated that the only GD high risk allele confirmed for both Asians and Caucasians was HLA-DRB1*03:01 [5] whose specificity for GD is quite low, because it is an allele typical for many autoimmune disorders.

In addition to the novel finding of GO-related HLA alleles, we have also identified alleles potentially protective against GO, but not against non-GO course of GD. Among the two of them, the protective effect of HLA-C*04:01 was demonstrated when GO group was compared either to non-GO or to control group. This allele was previously described as SAT high risk one [7]. The present finding of its protective role against GO can to some extent explain the phenomenon of extremely rare co-presence of SAT and GO. Previously, potential significance of HLA background on the course of SAT and GD in patients with co-presence of these two diseases was postulated [10]. The is no LD between HLA-C*04:01 and the other GO protective alleles - HLA-C*03:01, -C*07:02 or DRB1*15:02 [22, 30, 31], thus each of them can be considered an independent protective factor. Interestingly, HLA-B*15:02 was simultaneously found to be associated with an increased risk of non-GO GD as compared to control group. In our cohort, none of GO patients was HLA-B*15:02 positive. This allele occurred exclusively in non-GO group. It is worth emphasizing that Chen et al. postulated a crucial role of this allele in GD development in Chinese cohort [32], however its frequency has never been analyzed separately in GO and non-GO groups.

All the three alleles related to the high risk of GO as compared to non-GO, i.e. HLA-A*32:01, -B*39:01 and -C*08:02, were not found to be GD high risk alleles in our previous study [5]. Such correlation is clearly visible only if GO group is separated and compared to non-GO group. These three alleles are not associated with non-GO, thus when the GO group was not analyzed separately but together with non-GO, as the whole GD group [5], the difference could not be significant. Similar situation regards protective effect of HLA-C*04:01 and DRB1*15:01 against GO which has been observed in the present study but in our previous report no correlation between this allele and the overall risk of GD development was found [5].

On the contrary, all alleles found in the present study as associated with GD either with GO or without GO, were also demonstrated as related to the high risk of GD in our previous study [5], with HLA-C*03:02 being an entirely novel finding there [5] and HLA-DRB1*03:01 and -DQB1*02:01 being earlier postulated by other authors [2, 4, 33–36]. Among this group of three alleles, HLA-C*03:02 is an independent risk factor not being with LD with others. HLA-DRB1*03:01 is in LD with -DQB1*02:01 [22, 37] and it should be kept in mind that susceptibility associated with alleles being in LD cannot be considered fully independent if both of them are present. However, a single presence of any of them constitutes the risk factor of the disease. Being aware of this fact is especially important in regard to our results in patients with multi-allele susceptibility, as in most of the patients with three or four high risk alleles the co-presence of HLA-DRB1*03:01 and -DQB1*02:01 was found. In such cases, these alleles cannot be considered independent risk factors.

The present study has also identified the alleles which are associated with high probability of non-GO course of GD but not with GO. Correlation between the presence of most of these alleles and the overall GD risk was demonstrated in our previous study [5]. However, the significance of HLA-B*51:01 and C*16:01 has never been found before, and HLA-DRB1*15:02 was only postulated as a high risk factor of GD in Asians, as it was stated above. The increased risk of GD in carriers of any of the rest three alleles, i.e. HLA-B*08:01, -C*07:01, and DQB1*03:01, had been postulated before [2, 4, 34] and confirmed in our previous [5] and present studies. It should be underlined that the entirely novel correlation of non-GO GD and HLA-B*51:01, reported here for the first time, should be considered potentially expected, because of LD between this allele and HLA-C*14:02, reported as a high risk factor of GD for the first time in our recent study [5, 30, 31]. Moreover, HLA-C*16:02 – the second allele demonstrated as related to non-GO GD – is in LD with -B*51:01 [30, 31]. Therefore, current demonstration of the significance of HLA-B*51:01 and -C*16:02 complements our previous findings. Among the rest of the alleles associated with increased risk of GD but not with GO, HLA-B*08:01 is in LD with C*07:01, while DRB1*01:03 is in LD with -DQB1*03:01 [30, 31]. Therefore, in this group, only HLA-B*39:06 can be considered fully independent, while the rest of them is not entirely independent if alleles being in LD are present together.

In our previous study [5], the protective effect of HLA-B*07:02 and -C*07:02 was described for the first time. The present study has confirmed the protective role of both of these alleles against non-GO GD but only -C*07:02 against GO. The same role of both of them in regard to non-GO GD can be further proved by LD between them in Caucasian population [30, 31]. Therefore, they cannot be considered independent. Similarly to the previously discussed alleles, the significant differences between GO and non-GO patients with GD are clearly visible here. Both of these alleles were described as protective in regard to overall GD development in our previous study [5]. However, in the present study, when GO and non-GO groups were analyzed separately, the protective effect of both of these alleles appeared to concern non-GO group only. In GO group, the difference did not reach statistical significance for HLA-B*07:02 allele. Interestingly, HLA-A*32:01, demonstrated here as associated with the high risk of GO, was found in none of non-GO patients. This fact can further confirm the role of this allele in GO development and one should not consider this allele as protective against non-GO course of GD, but rather as a highly potent GO risk factor. On the basis of our results, we can speculate that the presence of this allele constitutes such a strong susceptibility factor that all patients with GD and HLA-A*32:01 will develop GO. Similarly, we did not find any patient without GO and with HLA-B*39:01. The strength of the correlation between GO and these two alleles may be – therefore – similar.

The present study has demonstrated for the first time strong associations between GO and HLA alleles, with HLA-A*01:01, -A*32:01, -B*37:01, -B*39:01, -B*42:01, -C*08:02, C*03:02, DRB1*03:01, DRB1*14:01 and DQB1*02:01 being genetic markers of increased risk of GO, and HLA-C*04:01, -C*03:04, -C*07:02 and DRB1*15:02 being the protective alleles. Moreover, we have found which alleles are associated with increased and decreased probability of non-GO GD but have no correlation with the risk of GO development. Identification of these groups of GO-related and GO-protective alleles, as well as the alleles strongly related to non-GO GD, fills the existing gap in the knowledge on genetic background of GO and constitutes a significant step in the development of personalized medicine. The present findings provide a precise diagnostic tool for the individual GO risk assessment, which can significantly facilitate tailoring the prevention strategy and treatment modality in an individual patient.

## Supplementary information

GO and non-GO HLA results

HLA results for healthy controls

## Data Availability

The source data are included as Supplementary Materials.
