# Supplementary material for: Significance of HLA in the development of Graves’ orbitopathy
Source: Genes Immun. 2023 Jan 13;24(1):32–8. doi: 10.1038/s41435-023-00193-z (PMC9935388; doi:10.1038/s41435-023-00193-z)
Supplement: Supplementary file 1 — GO and non-GO HLA results [file 41435_2023_193_MOESM1_ESM.pdf]

SAMPLE	HLA-AAG1	HLA-AAG2	HLA-BAG1	HLA-BAG2	HLA-CAG2	HLA-CAG1	HLA-DRB1	HLA-DQB1
ORGD0001	01:01	02:01	08:01	27:02	02:02	07:01	03:01	16:01
ORGD0002	02:01	31:01	15:18	44:02	05:01	07:04	13:01	16:01
ORGD0003	02:01	03:01	18:01	27:05	02:02		01:01	04:01
ORGD0004	03:01	11:01	15:01	40:02	02:02	03:02	07:01	11:01
ORGD0005	11:01		15:01	55:01	03:03		01:01	04:01
ORGD0006	02:01	03:01	38:01	39:01	12:02	12:03	11:01	16:01
ORGD0007	02:01	26:01	38:01	39:01	03:02	12:03	04:04	04:07
ORGD0008	02:01	03:01	35:01		04:01		03:01	11:01
ORGD0009	01:01	25:01	35:01	35:24	04:01		01:01	13:02
ORGD0010	01:01	11:01	39:06		07:01	07:02	03:01	08:01
ORGD0011	03:01	31:01	07:02	39:01	06:02	12:03	15:01	16:01
ORGD0012	01:01	32:01	08:01	15:01	03:04	07:01	03:01	04:04
ORGD0013	01:01	03:01	14:02	52:01	08:02	12:02	03:01	11:01
ORGD0014	01:01	30:01	07:02	55:02	06:02	07:01	07:01	15:01
ORGD0015	02:01	26:01	44:05		02:02		01:01	16:01
ORGD0016	02:01	33:01	27:05	41:01	02:02	17:01	01:01	11:01
ORGD0017	02:01	24:02	51:01		14:02		08:01	11:01
ORGD0018	24:02	26:01	07:02	18:01	07:02	12:03	07:01	15:01
ORGD0019	02:01	11:01	18:01	35:01	04:01	12:04	01:01	11:01
ORGD0020	01:01	02:01	07:02	42:01	07:01	17:01	03:01	15:01
ORGD0021	24:02	25:01	18:01		12:02		11:03	11:04
ORGD0022	02:01	24:02	07:02	27:05	01:02	07:01	11:01	15:01
ORGD0023	01:01	25:01	08:01	18:01	07:01	12:03	03:01	16:01
ORGD0024	02:01	26:01	13:02	48:01	06:02	08:03	12:01	
ORGD0025	01:01	25:01	15:17	18:01	07:01	12:03	13:01	15:01
ORGD0026	02:01		13:02	51:01	06:02	14:02	03:01	11:03
ORGD0027	01:01	23:01	08:01		07:01		03:01	
ORGD0028	01:01	02:01	07:02	14:01	07:02	08:02	07:01	16:01
ORGD0029	03:01	24:02	35:01	56:01	01:02	15:02	01:01	11:01
ORGD0030	01:01		08:01	39:03	07:01	07:02	03:01	08:01
ORGD0031	02:01	68:01	15:01	40:01	03:02	07:01	03:01	13:01
ORGD0032	01:01	02:01	08:01	44:02	05:01	07:01	03:01	13:02
ORGD0033	03:01	25:01	27:05	37:01	02:02	06:02	03:01	04:01
ORGD0034	01:01	26:01	08:01	37:01	06:02	07:01	03:01	08:01
ORGD0035	03:01	26:01	35:03		04:01		03:01	08:01
ORGD0036	02:01	24:02	39:01	44:03	04:01	06:02	04:02	11:04
ORGD0037	01:01	26:08	08:01	55:01	03:03	07:01	03:01	14:01
ORGD0038	03:01	25:01	18:01	57:01	06:02	12:03	07:01	13:02
ORGD0039	02:01	03:01	07:02	42:01	07:01	07:02	03:01	15:01
ORGD0040	01:01	02:01	08:01	44:02	02:02	07:01	03:01	16:01
ORGD0041	11:01	66:01	08:01	51:01	15:02	17:01	11:01	13:03
ORGD0042	03:01		07:02	15:01	03:03	07:02	07:01	15:01
ORGD0043	25:01	32:01	07:02	56:01	01:02	07:01	01:01	07:01
ORGD0044	02:01		13:02	15:01	03:03	06:02	07:01	13:01
ORGD0045	02:01	68:01	13:02	40:01	03:02	06:02	04:04	07:01
ORGD0046	30:01	31:01	39:01	44:02	05:01	12:03	04:01	13:01
ORGD0047	01:01	26:01	37:01	38:01	06:02	12:03	04:07	15:01
ORGD0048	02:01	03:01	13:02	35:01	04:01	06:02	12:01	13:03
ORGD0049	24:02	32:01	14:02	39:01	08:02	12:03	13:03	16:01

ORGD0050	02:01	03:01	15:01	44:02	03:03	05:01	04:04	15:01
ORGD0051	02:01	32:01	27:02	27:05	01:02	02:02	01:01	16:01
ORGD0052	03:01	24:02	37:01	51:01	06:02	12:03	01:01	14:01
ORGD0053	01:01		08:01		07:01		03:01	
ORGD0054	02:01	25:01	40:02	50:01	02:02	06:02	07:01	11:01
ORGD0055	01:01	11:01	56:01	57:01	01:02	06:02	01:01	14:01
ORGD0056	01:01		08:01		07:01		03:01	
ORGD0057	11:01	24:02	35:01	38:01	04:01	12:03	11:01	13:01
ORGD0058	11:01	24:02	07:02	51:01	07:02	15:02	04:04	15:01
ORGD0059	01:01	33:01	14:01	44:02	02:02	08:02	01:02	07:01
ORGD0060	01:01	68:01	08:01	44:02	07:01	07:04	01:01	03:01
ORGD0061	03:01	29:02	15:01	47:02	06:02	08:02	07:01	11:01
ORGD0062	01:01	03:01	18:01	37:01	02:02	07:01	04:01	11:01
ORGD0063	01:01	02:01	27:05	57:01	02:02	06:02	10:01	13:03
ORGD0064	01:01		08:01		07:01		03:01	13:01
ORGD0065	02:01	03:01	38:01	44:02	03:03	12:03	12:01	13:01
ORGD0066	01:01		08:01		07:01		03:01	11:01
ORGD0067	03:01	23:01	35:01	44:03	04:01		01:01	11:01
ORGD0068	01:01	24:02	03:01	05:01	07:01	07:02	03:01	04:04
ORGD0069	02:01	26:01	07:02	40:01	03:02	07:02	04:01	08:01
ORGD0070	02:01	32:01	15:01	41:02	03:03	17:01	11:04	13:03
NOGD0001	02:01	11:01	35:01	44:03	04:01	16:02	04:02	11:01
NOGD0002	26:01		44:02	49:01	05:01	07:01	01:01	12:01
NOGD0003	01:01	03:01	35:03	41:01	04:01	07:01	03:01	
NOGD0004	02:01		07:04	44:02	06:02	07:04	15:01	16:01
NOGD0005	02:01	66:01	18:01	41:02	07:01	17:01	11:03	13:03
NOGD0006	02:01		27:04	55:01	01:02	03:03	01:01	04:01
NOGD0007	24:02	29:02	35:03	45:01	04:01	06:02	04:01	11:01
NOGD0008	02:01		15:01	27:02	03:03	15:01	11:01	15:02
NOGD0009	02:01	31:01	08:01	15:01	03:02	07:01	03:01	11:01
NOGD0010	01:01	03:01	08:01	37:01	06:02	07:01	03:01	13:03
NOGD0011	01:01	30:01	08:01	15:03	06:02	12:03	03:01	07:01
NOGD0012	02:01	24:02	27:02	35:01	02:02	04:01	01:01	16:01
NOGD0013	02:01	11:01	27:02	35:01	02:02	04:01	13:03	16:01
NOGD0014	02:01	24:02	08:01	27:02	02:02	07:01	04:04	13:01
NOGD0015	02:01		40:01	55:01	03:03	03:04	03:01	15:01
NOGD0016	03:01	11:01	40:01	56:01	01:02	03:04	13:03	15:01
NOGD0017	02:01	26:01	38:01	40:01	03:04	12:03	04:04	13:01
NOGD0018	02:01	24:02	15:01	52:01	03:03	12:02	04:04	07:01
NOGD0019	01:01	03:01	07:02	35:02	06:02	07:02	11:04	15:01
NOGD0020	01:01	36:01	08:01	41:02	07:01	17:01	03:01	13:03
NOGD0021	01:01	11:01	08:01	35:01	04:01	07:01	12:01	13:01
NOGD0022	02:01	68:01	38:01		12:03		13:01	
NOGD0023	01:01	25:01	15:01	57:01	03:03	06:02	07:01	11:01
NOGD0024	02:01		27:05	51:01	02:02	14:02	01:01	08:03
NOGD0025	01:01	03:01	07:02	38:01	06:02	12:03	15:01	
NOGD0026	02:01	24:02	15:01	18:01	03:03	07:01	01:01	13:01
NOGD0027	01:01	26:01	39:06	52:01	06:02	12:02	01:01	15:02
NOGD0028	03:01	30:01	08:01	13:02	06:02	07:01	03:01	11:04
NOGD0029	11:01	24:02	35:01	39:06	04:01		01:01	04:01

NOGD0030	03:01	68:01	38:01	56:01	01:02	06:02	08:01	11:04
NOGD0031	01:01	02:01	08:01	15:01	03:02	07:01	03:01	13:01
NOGD0032	02:01	11:01	08:01	44:03	04:01		03:01	07:01
NOGD0033	01:01	25:01	08:01	18:01	07:01	12:03	03:01	04:01
NOGD0034	02:01		07:02	57:01	06:02	07:02	01:01	07:01
NOGD0035	02:01		07:02	42:01	07:01	17:01	03:01	15:01
NOGD0036	01:01	03:01	07:02	39:06	07:02	17:01	08:01	12:01
NOGD0037	26:01		35:01	51:07	04:01	14:02	13:02	14:01
NOGD0038	02:01	31:01	51:01		12:09	14:02	03:01	07:01
NOGD0039	02:01	11:01	15:01	40:02	02:02	03:03	11:01	13:01
NOGD0040	01:01	25:01	08:01	18:01	07:01	12:03	03:01	15:01
NOGD0041	02:01	23:01	15:01	50:01	04:01		08:01	11:01
NOGD0042	24:02	66:01	18:01	51:01	07:01	16:02	11:02	15:01
NOGD0043	11:01	24:02	35:01	56:01	01:03	04:01	11:01	13:01
NOGD0044	01:01	11:01	14:02	35:01	04:01	08:02	01:01	07:01
NOGD0045	02:17	11:01	51:01		15:02		03:01	04:01
NOGD0046	02:01	11:01	27:05	51:01	01:02	15:02	01:03	04:01
NOGD0047	02:01	68:01	15:01		03:02	03:03	01:01	04:04
NOGD0048	02:01	23:01	08:01	18:01	05:01	07:01	03:01	04:01
NOGD0049	24:02	24:03	18:01	51:01	07:01	12:02	15:01	15:02
NOGD0050	03:01	66:01	08:01		07:01		03:01	
NOGD0051	02:01	03:01	07:02	51:01	07:02	15:02	01:03	11:01
NOGD0052	02:01	25:01	08:01	18:01	07:01	12:03	03:01	13:01
NOGD0053	01:01	24:02	08:01		07:01		03:01	
NOGD0054	02:01	03:01	35:03	51:01	02:02	04:01	11:01	13:02
NOGD0055	02:01	30:01	07:02	13:02	06:02	07:01	15:01	
NOGD0056	23:01	68:01	40:01	44:02	04:01		13:01	15:01
NOGD0057	23:01	25:01	44:03	52:01	04:01	12:02	07:01	15:02
NOGD0058	02:01		44:02	51:01	05:01	16:02	04:03	15:01
NOGD0059	02:01	03:01	08:01	44:03	04:01	07:01	03:01	07:01
NOGD0060	02:01	24:02	39:06	44:02	05:01	07:02	04:01	08:01
NOGD0061	03:01	26:01	27:05	44:03	01:02	04:01	07:01	11:01
NOGD0062	02:01	02:05	51:01	58:01	03:02	16:02	04:05	13:02
NOGD0063	02:01		27:05	51:01	01:02	07:01	01:01	13:01
NOGD0064	01:01	24:02	08:01	44:02	02:02	07:01	03:01	11:04
NOGD0065	03:01	30:01	18:01	35:01	04:01	07:01	11:01	11:04
NOGD0066	01:01		08:01		07:01		03:01	07:01
NOGD0067	26:01	31:01	08:01	35:01	04:01	07:01	03:01	12:01
NOGD0068	01:01	03:02	08:01	18:01	07:01		03:01	04:04
NOGD0069	03:02	26:01	07:02	15:01	03:03	07:02	07:01	15:02
NOGD0070	02:01	29:02	40:01	44:03	03:03	16:01	04:03	15:01
NOGD0071	01:01	11:01	08:01	35:01	04:01	07:01	03:01	11:01
NOGD0072	02:01	24:02	13:02	44:02	06:02	07:04	07:01	16:01
NOGD0073	01:01	02:01	51:01	52:01	12:02	14:02	08:01	15:02
NOGD0074	01:01	25:01	18:01	57:01	06:02	12:03	07:01	08:01
NOGD0075	02:01	11:01	18:01	35:01	12:03		01:01	04:01
NOGD0076	03:01	68:01	44:02	45:01	06:02	07:04	07:01	11:01
NOGD0077	01:01	26:01	08:01	39:03	07:01	12:03	03:01	04:02
NOGD0078	01:01	02:01	08:01	40:02	02:02	07:01	03:01	11:01
NOGD0079	02:01	25:01	18:01	44:02	07:04	12:03	15:01	16:01

NOGD0080	26:01	31:01	35:03		03:02	12:03	11:13	14:01
NOGD0081	02:01	29:01	18:01	27:05	01:02	07:01	01:03	11:01
NOGD0082	25:01	29:02	37:01	55:01	03:02	06:02	12:01	15:01
NOGD0083	03:01		08:01	35:01	04:01	18:01	03:01	15:01
NOGD0084	02:01	33:01	14:01	44:02	07:04		01:01	11:01
NOGD0085	01:01	02:01	38:01	51:01	12:03	14:02	04:01	13:03
NOGD0086	02:01	03:01	15:01	44:03	03:04	04:01	07:01	11:01
NOGD0087	03:01	31:01	15:01	44:02	03:03	05:01	04:01	11:01
NOGD0088	02:01	30:01	08:01	13:02	06:02	07:01	03:01	11:04
NOGD0089	02:01	03:01	51:01	52:01	15:02		03:01	04:03
NOGD0090	01:01	02:01	18:01		06:02	12:03	04:01	11:01
NOGD0091	03:01	24:02	07:02	40:01	03:02	07:01	11:01	13:02

HLA-DRB1AG1	HLA-DRB1AG2
02:01	05:02
05:02	06:03
03:01	05:01
03:01	03:02
03:02	05:01
03:01	05:02
03:01	03:02
02:01	03:01
05:01	06:04
02:01	04:02
05:02	06:02
02:01	03:02
02:01	03:01
02:02	06:02
05:01	05:02
03:01	05:01
03:01	04:02
03:03	06:02
03:01	05:01
02:01	06:02
03:01	
03:01	
02:01	06:02
03:01	
05:01	06:02
02:01	03:01
02:01	
02:02	05:02
03:01	05:01
02:01	04:02
02:01	06:03
02:01	06:04
02:01	03:01
02:01	04:02
02:01	04:02
03:01	03:02
02:01	05:03
03:03	06:04
02:01	06:02
02:01	05:02
03:01	
02:02	06:02
02:02	05:01
02:02	06:03
02:02	03:02
03:01	06:03
03:01	06:02
03:01	
03:01	05:02

03:02	06:02
05:01	05:02
05:01	05:03
02:01	
02:02	03:01
05:01	05:03
02:01	
03:01	06:03
03:02	06:02
03:03	05:01
02:01	05:01
02:02	03:01
03:01	03:02
03:01	05:01
02:01	06:03
03:01	06:03
02:01	03:01
03:01	05:01
02:01	03:02
03:02	04:02
03:01	06:02
03:01	03:02
03:01	05:01
02:01	
05:02	06:02
03:01	
03:02	05:01
03:01	
03:01	06:02
03:01	
02:01	03:01
02:01	02:02
05:01	05:02
03:01	05:02
03:02	06:03
02:01	06:02
03:01	06:02
03:02	06:03
02:02	03:02
03:01	06:02
02:01	03:01
03:01	06:03
06:03	
03:01	03:02
03:01	05:01
06:02	
05:01	06:03
05:01	06:01
02:01	03:01
03:02	05:01

03:01	
02:01	06:03
02:01	02:02
02:01	03:02
03:03	05:01
02:01	06:03
03:01	04:02
05:03	06:05
02:01	02:02
03:01	06:03
02:01	06:02
03:01	04:02
03:01	06:02
03:01	06:03
03:03	05:01
02:01	03:02
03:01	03:02
03:02	
02:01	03:01
06:01	06:02
02:01	
03:01	05:01
02:01	06:03
02:01	
03:01	06:04
06:02	
06:02	06:04
02:02	06:01
03:04	06:02
02:01	02:02
03:01	04:02
02:02	03:01
03:02	06:09
05:01	06:03
02:01	03:01
03:01	
02:01	03:03
02:01	03:01
02:01	03:02
02:02	06:01
03:04	06:02
02:01	03:01
02:02	05:02
04:02	06:01
03:03	04:02
03:01	03:02
02:02	03:01
02:01	03:02
02:01	03:01
05:02	06:02

03:01	05:03
03:01	05:01
03:01	06:02
02:01	06:02
03:01	05:01
03:01	03:02
02:01	03:01
03:01	03:04
02:01	03:01
02:01	03:02
03:01	03:02
03:01	06:04
