# Supplementary material for: Significance of HLA in the development of Graves’ orbitopathy
Source: Genes Immun. 2023 Jan 13;24(1):32–8. doi: 10.1038/s41435-023-00193-z (PMC9935388; doi:10.1038/s41435-023-00193-z)
Supplement: Supplementary file 2 — HLA results for healthy controls [file 41435_2023_193_MOESM2_ESM.pdf]

SAMPLE	healthy cont	HLA-A	HLA-B	HLA-C	HLA-DRB1	HLA-DQB1
HC0001	CONTIG1	A*01:01	B*07:02	C*04:01	DRB1*08:01	DQB1*04:02
	CONTIG2	A*03:01	B*35:01	C*07:02	DRB1*14:54	DQB1*05:03
HC0002	CONTIG1	A*02:01	B*13:02	C*04:01	DRB1*07:01	DQB1*02:02
	CONTIG2	A*24:02	B*35:03	C*06:02	DRB1*12:01	DQB1*03:01
HC0003	CONTIG1	A*01:01	B*08:01	C*05:01	DRB1*03:01	DQB1*02:01
	CONTIG2	A*24:02	B*44:02	C*07:01	DRB1*11:02	DQB1*03:19
HC0004	CONTIG1	A*02:01	B*35:01	C*04:01	DRB1*11:04	DQB1*03:01
	CONTIG2	A*26:01	B*44:27	C*05:01	DRB1*16:01	DQB1*05:02
HC0005	CONTIG1	A*11:01	B*39:01	C*01:02	DRB1*12:01	DQB1*03:01
	CONTIG2	A*31:01	B*56:01	C*12:03	DRB1*15:01	DQB1*06:02
HC0006	CONTIG1	A*02:01	B*07:02	C*01:02	DRB1*01:01	DQB1*05:01
	CONTIG2	A*03:01	B*27:05	C*07:02	DRB1*15:01	DQB1*06:02
HC0007	CONTIG1	A*24:02	B*13:02	C*06:02	DRB1*07:01	DQB1*02:02
	CONTIG2	A*66:01	B*49:01	C*07:01	DRB1*11:01	DQB1*03:01
HC0008	CONTIG1	A*03:01	B*07:02	C*07:02	DRB1*11:01	DQB1*03:01
	CONTIG2	A*24:02	-	-	DRB1*15:01	DQB1*06:02
HC0009	CONTIG1	A*01:01	B*08:01	C*04:01	DRB1*03:01	DQB1*02:01
	CONTIG2	A*24:02	B*44:03	C*07:01	DRB1*11:01	DQB1*03:01
HC0010	CONTIG1	A*02:01	B*15:01	C*03:04	DRB1*04:01	DQB1*03:02
	CONTIG2	A*23:01	B*50:01	C*06:02	DRB1*16:01	DQB1*05:02
HC0011	CONTIG1	A*02:01	B*07:02	C*05:01	DRB1*04:01	DQB1*03:01
	CONTIG2	A*31:01	B*44:02	C*07:02	DRB1*15:01	DQB1*06:02
HC0012	CONTIG1	A*01:01	B*07:02	C*07:01	DRB1*03:01	DQB1*02:01
	CONTIG2	A*03:01	B*08:01	C*07:02	DRB1*16:01	DQB1*05:02
HC0013	CONTIG1	A*02:01	B*35:01	C*04:01	DRB1*04:04	DQB1*03:01
	CONTIG2	A*11:01	B*44:02	C*07:04	DRB1*13:03	DQB1*03:02
HC0014	CONTIG1	A*24:02	B*14:02	C*07:02	DRB1*01:02	DQB1*05:01
	CONTIG2	A*66:01	B*39:06	C*08:02	DRB1*16:01	DQB1*05:02
HC0015	CONTIG1	A*11:01	B*13:02	C*06:02	DRB1*04:04	DQB1*02:02
	CONTIG2	A*30:01	B*51:01	C*15:02	DRB1*07:01	DQB1*03:02
HC0016	CONTIG1	A*02:01	B*35:02	C*06:02	DRB1*01:01	DQB1*02:01
	CONTIG2	A*26:01	B*51:01	C*14:02	DRB1*03:01	DQB1*05:01
HC0017	CONTIG1	A*02:01	B*13:02	C*04:01	DRB1*07:01	DQB1*02:02
	CONTIG2	A*11:01	B*35:01	C*06:02	DRB1*11:01	DQB1*03:01
HC0018	CONTIG1	A*02:01	B*35:01	C*05:01	DRB1*04:01	DQB1*03:01
	CONTIG2	A*03:01	B*44:02	C*07:02	DRB1*11:01	DQB1*03:01
HC0019	CONTIG1	A*02:01	B*08:01	C*04:01	DRB1*03:01	DQB1*02:01
	CONTIG2	-	B*15:01	C*07:01	DRB1*13:01	DQB1*06:03
HC0020	CONTIG1	A*02:01	B*07:02	C*07:02	DRB1*13:01	DQB1*06:02
	CONTIG2	A*24:02	-	-	DRB1*15:01	DQB1*06:03
HC0021	CONTIG1	A*02:01	B*18:01	C*05:01	DRB1*01:01	DQB1*05:01
	CONTIG2	A*25:01	B*44:02	C*12:03	DRB1*10:01	-
HC0022	CONTIG1	A*02:01	B*18:01	C*12:03	DRB1*13:01	DQB1*06:03
	CONTIG2	A*25:01	B*51:01	C*15:02	-	-
HC0023	CONTIG1	A*02:01	B*07:02	C*05:01	DRB1*04:01	DQB1*03:01
	CONTIG2	A*31:01	B*44:02	C*07:02	DRB1*15:01	DQB1*06:02
HC0024	CONTIG1	A*11:01	B*49:01	C*01:02	DRB1*01:01	DQB1*05:01
	CONTIG2	A*26:01	B*56:01	C*07:01	-	-
HC0025	CONTIG1	A*01:01	B*08:01	C*02:02	DRB1*01:01	DQB1*02:01

	CONTIG2	A*24:02	B*27:02	C*07:01	DRB1*03:01	DQB1*05:01
HC0026	CONTIG1	A*02:01	B*07:02	C*02:02	DRB1*07:01	DQB1*03:03
	CONTIG2	A*11:01	B*27:05	C*07:02	DRB1*15:01	DQB1*06:02
HC0027	CONTIG1	A*01:01	B*27:05	C*01:02	DRB1*01:03	DQB1*03:01
	CONTIG2	A*26:01	-	-	DRB1*11:01	-
HC0028	CONTIG1	A*02:01	B*27:02	C*02:02	DRB1*11:01	DQB1*03:01
	CONTIG2	-	B*35:03	C*04:01	-	-
HC0029	CONTIG1	A*02:01	B*27:02	C*02:02	DRB1*01:01	DQB1*05:01
	CONTIG2	A*11:01	B*44:02	C*07:04	DRB1*16:01	DQB1*05:02
HC0030	CONTIG1	A*01:01	B*08:01	C*05:01	DRB1*03:01	DQB1*02:01
	CONTIG2	A*02:01	B*44:02	C*07:01	DRB1*13:01	DQB1*06:03
HC0031	CONTIG1	A*01:01	B*08:01	C*06:02	DRB1*03:01	DQB1*02:01
	CONTIG2	A*02:01	B*57:01	C*07:01	DRB1*07:01	DQB1*03:03
HC0032	CONTIG1	A*29:02	B*13:02	C*06:02	DRB1*07:01	DQB1*02:02
	CONTIG2	A*68:01	B*44:03	C*16:01	DRB1*09:01	DQB1*03:03
HC0033	CONTIG1	A*02:01	B*18:01	C*07:01	DRB1*12:01	DQB1*03:01
	CONTIG2	A*03:01	B*38:01	C*12:03	DRB1*14:54	DQB1*05:03
HC0034	CONTIG1	A*02:01	B*50:01	C*06:02	DRB1*07:01	DQB1*02:02
	CONTIG2	A*02:05	B*57:01	-	DRB1*13:01	DQB1*06:03
HC0035	CONTIG1	A*02:01	B*15:01	C*03:03	DRB1*13:01	DQB1*06:02
	CONTIG2	A*25:01	B*18:01	C*12:03	DRB1*15:01	DQB1*06:03
HC0036	CONTIG1	A*03:01	B*07:02	C*07:02	DRB1*10:01	DQB1*05:01
	CONTIG2	A*11:01	B*35:01	C*15:02	DRB1*15:01	DQB1*06:02
HC0037	CONTIG1	A*02:01	B*07:04	C*07:01	DRB1*12:01	DQB1*03:01
	CONTIG2	A*32:01	B*15:17	C*07:02	DRB1*15:01	DQB1*06:02
HC0038	CONTIG1	A*02:01	B*35:01	C*06:02	DRB1*03:01	DQB1*02:01
	CONTIG2	-	B*57:01	C*15:02	DRB1*11:01	DQB1*03:01
HC0039	CONTIG1	A*02:01	B*07:02	C*07:01	DRB1*03:01	DQB1*02:01
	CONTIG2	A*11:01	B*08:01	C*07:02	DRB1*15:01	DQB1*06:02
HC0040	CONTIG1	A*25:01	B*18:01	C*03:02	DRB1*15:01	DQB1*05:02
	CONTIG2	A*33:03	B*58:01	C*12:03	DRB1*16:01	DQB1*06:02
HC0041	CONTIG1	A*02:01	B*07:02	C*07:02	DRB1*01:01	DQB1*05:01
	CONTIG2	A*25:01	B*51:01	C*14:02	DRB1*15:01	DQB1*06:02
HC0042	CONTIG1	A*03:01	B*44:02	C*05:01	DRB1*01:01	DQB1*03:01
	CONTIG2	A*23:01	B*49:01	C*07:01	DRB1*11:01	DQB1*05:01
HC0043	CONTIG1	A*03:01	B*07:02	C*07:02	DRB1*15:01	DQB1*05:02
	CONTIG2	A*24:02	B*38:01	C*12:03	DRB1*16:01	DQB1*06:02
HC0044	CONTIG1	A*01:01	B*08:01	C*06:02	DRB1*03:01	DQB1*02:01
	CONTIG2	-	B*37:01	C*07:01	DRB1*11:03	DQB1*03:01
HC0045	CONTIG1	A*02:01	B*14:02	C*03:04	DRB1*13:02	DQB1*03:01
	CONTIG2	A*68:02	B*39:01	C*08:02	DRB1*13:03	DQB1*06:04
HC0046	CONTIG1	A*02:01	B*07:02	C*06:02	DRB1*07:01	DQB1*02:02
	CONTIG2	A*24:02	B*13:02	C*07:02	DRB1*15:01	DQB1*06:02
HC0047	CONTIG1	A*11:01	B*14:02	C*08:02	DRB1*01:01	DQB1*05:01
	CONTIG2	A*33:01	B*52:01	C*12:02	DRB1*01:02	DQB1*05:01
HC0048	CONTIG1	A*02:01	B*40:02	C*02:02	DRB1*07:01	DQB1*02:02
	CONTIG2	A*02:01	B*44:03	C*04:01	DRB1*11:01	DQB1*03:01
HC0049	CONTIG1	A*11:01	B*38:01	C*04:01	DRB1*04:04	DQB1*03:01
	CONTIG2	A*68:01	B*51:01	C*12:03	DRB1*11:04	DQB1*03:02
HC0050	CONTIG1	A*02:01	B*07:02	C*02:02	DRB1*15:01	DQB1*05:02

	CONTIG2	A*11:01	B*27:02	C*07:02	DRB1*16:01	DQB1*06:02
HC0051	CONTIG1	A*01:01	B*08:01	C*01:02	DRB1*01:01	DQB1*02:01
	CONTIG2	A*25:01	B*27:05	C*07:01	DRB1*03:01	DQB1*05:01
HC0052	CONTIG1	A*02:01	B*35:01	C*03:03	DRB1*04:02	DQB1*03:02
	CONTIG2	A*26:01	B*38:01	C*12:03	DRB1*08:01	DQB1*04:02
HC0053	CONTIG1	A*02:01	B*18:03	C*06:02	DRB1*04:04	DQB1*02:02
	CONTIG2	A*11:01	B*50:01	C*07:01	DRB1*07:01	DQB1*03:02
HC0054	CONTIG1	A*02:01	B*35:01	C*01:02	DRB1*14:01	DQB1*05:03
	CONTIG2	A*11:01	B*51:01	C*04:01	DRB1*15:01	DQB1*06:02
HC0055	CONTIG1	A*02:01	B*44:02	C*03:03	DRB1*11:04	DQB1*03:01
	CONTIG2	A*26:01	B*55:01	C*05:01	DRB1*14:54	DQB1*05:03
HC0056	CONTIG1	A*02:01	B*41:02	C*01:02	DRB1*13:03	DQB1*03:01
	CONTIG2	A*31:01	B*56:01	C*17:03	DRB1*14:54	DQB1*05:03
HC0057	CONTIG1	A*24:02	B*44:02	C*05:32	DRB1*07:01	DQB1*03:03
	CONTIG2	A*68:01	B*51:01	C*07:02	DRB1*16:01	DQB1*05:02
HC0058	CONTIG1	A*02:01	B*07:02	C*07:01	DRB1*03:01	DQB1*02:01
	CONTIG2	A*03:01	B*08:01	C*07:02	DRB1*15:01	DQB1*06:02
HC0059	CONTIG1	A*02:01	B*07:02	C*07:02	DRB1*15:01	DQB1*06:02
	CONTIG2	A*03:01	-	-	-	-
HC0060	CONTIG1	A*26:01	B*13:02	C*03:03	DRB1*07:01	DQB1*02:02
	CONTIG2	A*30:01	B*44:02	C*06:02	DRB1*11:03	DQB1*03:01
HC0061	CONTIG1	A*03:01	B*44:03	C*04:01	DRB1*07:01	DQB1*02:02
	CONTIG2	A*23:01	-	-	-	-
HC0062	CONTIG1	A*02:01	B*27:05	C*02:02	DRB1*07:01	DQB1*03:03
	CONTIG2	-	B*35:01	C*04:01	DRB1*14:01	DQB1*05:03
HC0063	CONTIG1	A*02:01	B*15:01	C*03:04	DRB1*01:01	DQB1*05:01
	CONTIG2	-	B*40:01	C*07:02	-	-
HC0064	CONTIG1	A*01:01	B*08:01	C*06:02	DRB1*03:01	DQB1*02:01
	CONTIG2	-	B*57:01	C*07:01	DRB1*13:05	DQB1*03:01
HC0065	CONTIG1	A*02:01	B*13:02	C*03:04	DRB1*04:03	DQB1*02:02
	CONTIG2	A*11:01	B*40:01	C*06:02	DRB1*07:01	DQB1*03:02
HC0066	CONTIG1	A*02:01	B*18:01	C*01:02	DRB1*12:01	DQB1*03:01
	CONTIG2	-	B*44:05	C*02:02	DRB1*14:54	DQB1*05:03
HC0067	CONTIG1	A*01:01	B*08:01	C*07:01	DRB1*03:01	DQB1*02:01
	CONTIG2	A*23:01	B*49:01	C*07:01	-	-
HC0068	CONTIG1	A*02:01	B*13:02	C*04:01	DRB1*03:01	DQB1*02:01
	CONTIG2	A*23:01	B*44:03	C*06:02	DRB1*07:01	DQB1*02:02
HC0069	CONTIG1	A*02:01	B*18:01	C*06:02	DRB1*07:01	DQB1*03:02
	CONTIG2	A*32:01	B*57:01	C*07:01	DRB1*11:04	DQB1*03:03
HC0070	CONTIG1	A*11:01	B*07:02	C*04:01	DRB1*01:01	DQB1*03:02
	CONTIG2	A*11:01	B*35:01	C*07:02	DRB1*04:04	DQB1*05:01
HC0071	CONTIG1	A*02:05	B*35:03	C*04:01	DRB1*07:01	DQB1*02:02
	CONTIG2	A*03:01	B*50:01	C*06:02	DRB1*11:01	DQB1*03:01
HC0072	CONTIG1	A*03:01	B*55:01	C*01:02	DRB1*07:01	DQB1*03:03
	CONTIG2	A*11:01	B*56:01	-	DRB1*15:01	DQB1*06:02
HC0073	CONTIG1	A*03:01	B*07:02	C*07:02	DRB1*15:01	DQB1*05:02
	CONTIG2	-	-	-	DRB1*16:01	DQB1*06:02
HC0074	CONTIG1	A*02:01	B*14:01	C*02:02	DRB1*07:01	DQB1*02:02
	CONTIG2	A*26:01	B*27:02	C*08:02	DRB1*16:01	DQB1*05:02
HC0075	CONTIG1	A*24:02	B*08:01	C*03:03	DRB1*03:01	DQB1*02:01

	CONTIG2	A*33:03	B*15:01	C*07:01	DRB1*11:03	DQB1*03:01
HC0076	CONTIG1	A*02:01	B*27:02	C*02:02	DRB1*16:01	DQB1*05:02
	CONTIG2	A*68:01	B*44:27	C*07:04	-	-
HC0077	CONTIG1	A*02:01	B*18:01	C*04:01	DRB1*08:01	DQB1*03:01
	CONTIG2	A*03:01	B*35:03	C*07:01	DRB1*11:04	DQB1*04:02
HC0078	CONTIG1	A*02:01	B*27:05	C*02:02	DRB1*01:01	DQB1*03:02
	CONTIG2	A*24:02	B*51:01	C*15:02	DRB1*04:04	DQB1*05:01
HC0079	CONTIG1	A*03:01	B*15:01	C*03:03	DRB1*11:01	DQB1*03:01
	CONTIG2	A*24:02	B*44:03	C*04:01	DRB1*13:01	DQB1*06:03
HC0080	CONTIG1	A*02:01	B*27:05	C*02:02	DRB1*04:03	DQB1*03:01
	CONTIG2	A*31:01	B*40:01	C*03:04	DRB1*11:27	DQB1*03:02
HC0081	CONTIG1	A*01:01	B*08:01	C*07:01	DRB1*03:01	DQB1*02:01
	CONTIG2	-	-	-	-	-
HC0082	CONTIG1	A*02:01	B*27:05	C*02:02	DRB1*07:01	DQB1*03:03
	CONTIG2	-	B*40:01	C*03:04	-	-
HC0083	CONTIG1	A*03:01	B*18:01	C*07:04	DRB1*01:01	DQB1*05:02
	CONTIG2	A*25:01	B*44:27	C*12:03	DRB1*16:01	DQB1*05:04
HC0084	CONTIG1	A*03:01	B*07:02	C*04:01	DRB1*01:01	DQB1*05:01
	CONTIG2	A*33:03	B*35:08	C*07:02	DRB1*15:01	DQB1*06:02
HC0085	CONTIG1	A*02:01	B*15:01	C*04:01	DRB1*01:01	DQB1*02:02
	CONTIG2	A*68:01	B*35:03	C*04:01	DRB1*07:01	DQB1*05:01
HC0086	CONTIG1	A*02:01	B*44:02	C*05:01	DRB1*04:03	DQB1*03:04
	CONTIG2	A*68:01	B*44:27	C*07:04	DRB1*13:01	DQB1*06:03
HC0087	CONTIG1	A*01:01	B*07:02	C*06:02	DRB1*07:01	DQB1*03:01
	CONTIG2	A*03:01	B*37:01	C*07:02	DRB1*11:03	DQB1*03:03
HC0088	CONTIG1	A*02:01	B*15:01	C*04:01	DRB1*01:01	DQB1*03:02
	CONTIG2	A*25:01	B*18:01	C*12:03	DRB1*04:01	DQB1*05:01
HC0089	CONTIG1	A*03:01	B*07:02	C*06:02	DRB1*07:01	DQB1*03:03
	CONTIG2	A*25:01	B*57:01	C*07:02	DRB1*15:01	DQB1*06:02
HC0090	CONTIG1	A*02:01	B*08:01	C*04:01	DRB1*01:01	DQB1*02:01
	CONTIG2	A*11:01	B*35:01	C*07:01	DRB1*03:01	DQB1*05:01
HC0091	CONTIG1	A*01:01	B*08:01	C*04:01	DRB1*03:01	DQB1*02:01
	CONTIG2	A*68:01	B*35:03	C*07:01	DRB1*04:01	DQB1*03:02
HC0092	CONTIG1	A*02:01	B*07:02	C*02:02	DRB1*07:01	DQB1*03:01
	CONTIG2	A*03:01	B*27:05	C*07:02	DRB1*13:03	DQB1*03:03
HC0093	CONTIG1	A*02:01	B*15:01	C*03:04	DRB1*01:01	DQB1*03:02
	CONTIG2	A*03:01	B*35:01	C*04:01	DRB1*04:01	DQB1*05:01
HC0094	CONTIG1	A*01:01	B*08:01	C*04:01	DRB1*01:01	DQB1*02:01
	CONTIG2	A*11:01	B*35:01	C*07:01	DRB1*03:01	DQB1*05:01
HC0095	CONTIG1	A*02:01	B*13:02	C*04:01	DRB1*01:01	DQB1*02:02
	CONTIG2	A*03:01	B*35:03	C*06:02	DRB1*07:01	DQB1*05:01
HC0096	CONTIG1	A*01:01	B*08:01	C*07:01	DRB1*03:01	DQB1*02:01
	CONTIG2	A*68:01	B*51:01	C*15:02	DRB1*13:01	DQB1*06:03
HC0097	CONTIG1	A*02:01	B*15:01	C*03:03	DRB1*07:01	DQB1*03:01
	CONTIG2	A*24:02	B*15:01	C*04:01	DRB1*11:03	DQB1*03:03
HC0098	CONTIG1	A*24:02	B*27:05	C*01:02	DRB1*01:01	DQB1*03:03
	CONTIG2	A*32:01	B*57:01	C*06:02	DRB1*07:01	DQB1*05:01
HC0099	CONTIG1	A*03:01	B*40:01	C*03:04	DRB1*15:01	DQB1*05:02
	CONTIG2	A*24:02	B*57:01	C*06:02	DRB1*16:01	DQB1*06:02
HC0100	CONTIG1	A*11:01	B*08:01	C*03:03	DRB1*03:01	DQB1*02:01

	CONTIG2	A*68:01	B*55:01	C*07:01	DRB1*11:01	DQB1*03:01
HC0101	CONTIG1	A*03:01	B*07:02	C*02:02	DRB1*11:01	DQB1*03:01
	CONTIG2	A*25:01	B*27:05	C*07:02	DRB1*15:01	DQB1*06:02
HC0102	CONTIG1	A*03:01	B*07:02	C*07:01	DRB1*04:08	DQB1*03:03
	CONTIG2	A*26:01	B*49:01	C*07:02	DRB1*07:01	DQB1*03:04
HC0103	CONTIG1	A*01:01	B*08:01	C*04:01	DRB1*13:01	DQB1*05:03
	CONTIG2	A*30:01	B*35:01	C*07:01	DRB1*14:01	DQB1*06:03
HC0104	CONTIG1	A*02:01	B*15:01	C*03:04	DRB1*04:01	DQB1*03:01
	CONTIG2	-	B*44:02	C*07:04	DRB1*11:01	DQB1*03:02
HC0105	CONTIG1	A*02:01	B*13:02	C*06:02	DRB1*04:01	DQB1*03:02
	CONTIG2	-	B*39:01	C*07:02	DRB1*15:01	DQB1*06:02
HC0106	CONTIG1	A*02:01	B*08:01	C*03:04	DRB1*03:01	DQB1*02:01
	CONTIG2	A*68:01	B*40:01	C*07:01	DRB1*07:01	DQB1*02:02
HC0107	CONTIG1	A*02:01	B*15:01	C*03:03	DRB1*13:01	DQB1*05:02
	CONTIG2	A*24:02	B*44:27	C*07:04	DRB1*16:01	DQB1*06:03
HC0108	CONTIG1	A*02:01	B*07:02	C*03:04	DRB1*08:01	DQB1*03:01
	CONTIG2	A*24:02	B*40:01	C*07:02	DRB1*12:01	DQB1*04:02
HC0109	CONTIG1	A*01:01	B*07:02	C*07:01	DRB1*03:01	DQB1*02:01
	CONTIG2	A*02:01	B*08:01	C*07:02	DRB1*16:01	DQB1*05:02
HC0110	CONTIG1	A*02:01	B*15:01	C*04:01	DRB1*04:01	DQB1*03:01
	CONTIG2	-	B*44:02	C*05:01	DRB1*07:01	DQB1*03:03
HC0111	CONTIG1	A*02:01	B*07:02	C*02:02	DRB1*01:01	DQB1*05:01
	CONTIG2	A*24:02	B*27:05	C*07:02	DRB1*15:01	DQB1*06:02
HC0112	CONTIG1	A*02:01	B*07:02	C*07:01	DRB1*07:01	DQB1*02:02
	CONTIG2	A*24:02	B*18:01	C*07:02	DRB1*08:01	DQB1*04:02
HC0113	CONTIG1	A*02:01	B*15:01	C*02:02	DRB1*04:01	DQB1*03:02
	CONTIG2	A*31:01	B*27:05	C*03:03	DRB1*16:01	DQB1*05:02
HC0114	CONTIG1	A*03:01	B*35:03	C*04:01	DRB1*08:01	DQB1*04:02
	CONTIG2	A*11:01	B*52:01	C*12:02	DRB1*15:01	DQB1*06:02
HC0115	CONTIG1	A*01:01	B*35:02	C*06:02	DRB1*13:02	DQB1*05:03
	CONTIG2	A*66:01	B*41:02	C*17:03	DRB1*14:54	DQB1*06:09
HC0116	CONTIG1	A*01:01	B*35:01	C*04:01	DRB1*07:01	DQB1*02:02
	CONTIG2	A*03:01	B*52:01	C*12:02	DRB1*13:01	DQB1*06:03
HC0117	CONTIG1	A*11:01	B*15:01	C*04:01	DRB1*08:01	DQB1*04:02
	CONTIG2	-	B*35:01	-	DRB1*15:01	DQB1*06:02
HC0118	CONTIG1	A*02:01	B*15:01	C*04:01	DRB1*08:01	DQB1*03:01
	CONTIG2	-	B*39:01	C*12:03	DRB1*12:01	DQB1*04:02
HC0119	CONTIG1	A*03:01	B*07:02	C*07:02	DRB1*13:05	DQB1*03:01
	CONTIG2	A*24:02	-	-	DRB1*16:01	DQB1*05:02
HC0120	CONTIG1	A*02:05	B*38:01	C*06:02	DRB1*07:01	DQB1*02:02
	CONTIG2	A*26:01	B*50:01	C*12:03	DRB1*15:01	DQB1*06:03
HC0121	CONTIG1	A*02:01	B*15:01	C*03:04	DRB1*04:01	DQB1*03:02
	CONTIG2	A*11:01	B*57:01	C*06:02	DRB1*13:22	DQB1*06:03
HC0122	CONTIG1	A*24:02	B*27:02	C*02:02	DRB1*03:01	DQB1*02:01
	CONTIG2	A*26:01	B*44:03	C*07:06	DRB1*07:01	DQB1*02:02
HC0123	CONTIG1	A*25:01	B*18:01	C*05:01	DRB1*15:01	DQB1*05:02
	CONTIG2	A*31:01	B*44:02	C*12:03	DRB1*16:01	DQB1*06:02
HC0124	CONTIG1	A*02:01	B*07:02	C*05:01	DRB1*13:01	DQB1*06:02
	CONTIG2	A*03:01	B*44:02	C*07:02	DRB1*15:01	DQB1*06:03
HC0125	CONTIG1	A*01:01	B*35:02	C*05:01	DRB1*11:04	DQB1*03:01

	CONTIG2	A*02:01	B*44:02	C*06:02	-	DQB1*03:01
HC0126	CONTIG1	A*02:01	B*15:01	C*03:03	DRB1*11:03	DQB1*03:01
	CONTIG2	A*26:01	B*55:01	-	DRB1*13:01	DQB1*06:03
HC0127	CONTIG1	A*11:01	B*35:01	C*04:01	DRB1*01:01	DQB1*03:04
	CONTIG2	A*68:01	B*35:03	C*12:03	DRB1*04:08	DQB1*05:01
HC0128	CONTIG1	A*01:01	B*08:01	C*03:03	DRB1*03:01	DQB1*02:01
	CONTIG2	A*24:02	B*55:01	C*07:01	DRB1*16:01	DQB1*05:02
HC0129	CONTIG1	A*02:01	B*15:01	C*01:02	DRB1*04:01	DQB1*03:01
	CONTIG2	A*24:02	B*27:05	C*07:02	DRB1*11:01	DQB1*03:02
HC0130	CONTIG1	A*01:01	B*08:01	C*07:01	DRB1*03:01	DQB1*02:01
	CONTIG2	A*31:01	B*18:01	-	DRB1*12:01	DQB1*03:01
HC0131	CONTIG1	A*01:01	B*18:01	C*05:01	DRB1*11:04	DQB1*03:01
	CONTIG2	A*24:02	B*44:02	C*06:02	DRB1*12:01	DQB1*03:02
HC0132	CONTIG1	A*02:01	B*08:01	C*06:02	DRB1*03:01	DQB1*02:01
	CONTIG2	A*24:02	B*37:01	C*07:02	DRB1*15:01	DQB1*06:03
HC0133	CONTIG1	A*01:01	B*08:01	C*02:02	DRB1*01:01	DQB1*02:01
	CONTIG2	A*23:01	B*27:05	C*07:01	DRB1*03:01	DQB1*05:01
HC0134	CONTIG1	A*01:01	B*40:02	C*02:02	DRB1*07:01	DQB1*03:01
	CONTIG2	A*32:01	B*57:01	C*06:02	DRB1*11:01	DQB1*03:03
HC0135	CONTIG1	A*01:01	B*08:01	C*06:02	DRB1*03:01	DQB1*02:01
	CONTIG2	-	B*37:01	C*07:01	DRB1*16:01	DQB1*05:02
HC0136	CONTIG1	A*11:01	B*14:02	C*08:02	DRB1*04:04	DQB1*03:01
	CONTIG2	A*33:01	B*51:01	C*15:02	DRB1*11:01	DQB1*03:02
HC0137	CONTIG1	A*02:01	B*35:01	C*04:01	DRB1*01:01	DQB1*05:01
	CONTIG2	-	B*44:27	C*07:04	DRB1*16:01	DQB1*05:02
HC0138	CONTIG1	A*01:01	B*08:01	C*07:01	DRB1*13:02	DQB1*06:02
	CONTIG2	A*03:01	B*14:02	C*08:02	DRB1*15:01	DQB1*06:09
HC0139	CONTIG1	A*02:01	B*18:01	C*04:01	DRB1*07:01	DQB1*02:02
	CONTIG2	A*26:01	B*44:03	C*07:01	DRB1*11:04	DQB1*03:01
HC0140	CONTIG1	A*02:01	B*07:04	C*04:01	DRB1*07:01	DQB1*02:02
	CONTIG2	A*03:01	B*35:01	C*07:02	DRB1*15:01	DQB1*06:02
HC0141	CONTIG1	A*03:01	B*13:02	C*06:02	DRB1*11:04	DQB1*03:01
	CONTIG2	A*11:01	B*35:03	C*12:03	DRB1*15:01	DQB1*06:02
HC0142	CONTIG1	A*01:01	B*08:01	C*04:01	DRB1*01:01	DQB1*02:01
	CONTIG2	A*03:01	B*35:01	C*07:01	DRB1*03:01	DQB1*05:01
HC0143	CONTIG1	A*03:01	B*07:02	C*07:02	DRB1*12:01	DQB1*03:01
	CONTIG2	A*24:02	B*51:01	C*16:02	DRB1*15:01	DQB1*06:02
HC0144	CONTIG1	A*01:01	B*18:01	C*04:01	DRB1*07:01	DQB1*02:02
	CONTIG2	A*02:01	B*44:03	C*12:03	DRB1*15:01	DQB1*06:02
HC0145	CONTIG1	A*02:01	B*07:02	C*03:03	DRB1*15:01	DQB1*06:02
	CONTIG2	A*03:01	B*15:01	C*07:02	-	-
HC0146	CONTIG1	A*03:01	B*07:02	C*06:02	DRB1*07:01	DQB1*02:02
	CONTIG2	A*32:01	B*13:02	C*07:02	DRB1*11:01	DQB1*03:01
HC0147	CONTIG1	A*02:01	B*15:39	C*04:01	DRB1*11:01	DQB1*03:01
	CONTIG2	-	B*35:01	C*04:01	-	-
HC0148	CONTIG1	A*02:01	B*14:02	C*03:04	DRB1*13:03	DQB1*03:01
	CONTIG2	A*03:01	B*15:01	C*08:02	-	-
HC0149	CONTIG1	A*02:01	B*15:01	C*04:01	DRB1*07:01	DQB1*03:03
	CONTIG2	A*25:01	B*18:01	C*12:03	DRB1*15:01	DQB1*06:02
HC0150	CONTIG1	A*02:01	B*15:01	C*03:03	DRB1*11:02	DQB1*03:19

	CONTIG2	A*02:05	B*58:01	C*07:18	DRB1*13:01	DQB1*06:03
HC0151	CONTIG1	A*01:01	B*08:01	C*01:02	DRB1*01:01	DQB1*05:01
	CONTIG2	A*02:01	B*27:05	C*07:01	DRB1*15:01	DQB1*06:02
HC0152	CONTIG1	A*01:01	B*45:01	C*06:02	DRB1*01:01	DQB1*03:03
	CONTIG2	A*29:02	B*57:01	-	DRB1*07:01	DQB1*05:01
HC0153	CONTIG1	A*01:01	B*08:01	C*06:02	DRB1*07:01	DQB1*02:02
	CONTIG2	A*02:05	B*50:01	C*07:01	DRB1*13:01	DQB1*06:03
HC0154	CONTIG1	A*02:01	B*35:01	C*04:01	DRB1*14:01	DQB1*05:02
	CONTIG2	A*11:01	B*44:27	C*07:04	DRB1*16:02	DQB1*05:03
HC0155	CONTIG1	A*02:01	B*27:02	C*02:02	DRB1*16:01	DQB1*05:02
	CONTIG2	A*25:01	B*44:27	C*07:04	-	-
HC0156	CONTIG1	A*02:01	B*38:01	C*12:03	DRB1*13:01	DQB1*03:01
	CONTIG2	A*66:01	B*41:02	C*17:03	DRB1*13:03	DQB1*06:03
HC0157	CONTIG1	A*02:01	B*18:01	C*05:01	DRB1*03:01	DQB1*02:01
	CONTIG2	A*30:02	B*44:02	C*05:01	DRB1*11:03	DQB1*03:01
HC0158	CONTIG1	A*01:01	B*07:02	C*01:02	DRB1*01:01	DQB1*05:01
	CONTIG2	A*11:01	B*27:05	C*07:02	DRB1*15:01	DQB1*06:02
HC0159	CONTIG1	A*02:01	B*27:05	C*01:02	DRB1*01:01	DQB1*05:01
	CONTIG2	-	B*44:27	C*03:04	DRB1*16:01	DQB1*05:02
HC0160	CONTIG1	A*02:01	B*13:02	C*06:02	DRB1*07:01	DQB1*02:02
	CONTIG2	A*24:02	B*41:02	C*17:03	DRB1*13:03	DQB1*03:01
HC0161	CONTIG1	A*03:01	B*08:01	C*07:02	DRB1*01:01	DQB1*02:01
	CONTIG2	A*32:01	B*52:01	C*12:02	DRB1*03:01	DQB1*05:01
HC0162	CONTIG1	A*02:01	B*18:01	C*02:02	DRB1*13:01	DQB1*05:02
	CONTIG2	A*11:01	B*27:02	C*07:01	DRB1*16:01	DQB1*06:03
HC0163	CONTIG1	A*02:01	B*07:02	C*04:01	DRB1*07:01	DQB1*02:02
	CONTIG2	A*03:01	B*13:02	C*07:02	DRB1*15:01	DQB1*06:02
HC0164	CONTIG1	A*01:01	B*35:01	C*04:01	DRB1*01:01	DQB1*03:04
	CONTIG2	A*03:01	B*35:03	C*12:03	DRB1*04:08	DQB1*05:01
HC0165	CONTIG1	A*02:01	B*51:01	C*12:30	DRB1*11:01	DQB1*03:01
	CONTIG2	A*11:01	B*52:01	C*14:02	DRB1*14:54	DQB1*05:03
HC0166	CONTIG1	A*02:01	B*44:02	C*05:01	DRB1*07:01	DQB1*02:02
	CONTIG2	A*29:02	B*44:03	C*16:01	DRB1*12:01	DQB1*03:01
HC0167	CONTIG1	A*03:01	B*07:02	C*07:01	DRB1*01:01	DQB1*05:01
	CONTIG2	-	B*15:17	C*07:02	DRB1*13:02	DQB1*06:07
HC0168	CONTIG1	A*01:01	B*35:01	C*06:02	DRB1*07:01	DQB1*03:03
	CONTIG2	-	B*57:01	C*15:02	DRB1*10:01	DQB1*05:01
HC0169	CONTIG1	A*03:01	B*07:02	C*02:02	DRB1*01:01	DQB1*05:01
	CONTIG2	A*68:01	B*35:01	C*04:01	DRB1*15:01	DQB1*06:02
HC0170	CONTIG1	A*02:01	B*07:02	C*04:01	DRB1*11:01	DQB1*03:01
	CONTIG2	A*11:01	B*35:01	C*07:02	DRB1*15:01	DQB1*06:02
HC0171	CONTIG1	A*01:01	B*07:02	C*07:01	DRB1*01:01	DQB1*02:01
	CONTIG2	A*24:02	B*08:01	C*07:02	DRB1*03:01	DQB1*05:01
HC0172	CONTIG1	A*02:01	B*37:01	C*02:02	DRB1*11:01	DQB1*03:01
	CONTIG2	A*31:01	B*40:01	C*03:04	DRB1*15:01	DQB1*05:02
HC0173	CONTIG1	A*32:01	B*15:01	C*04:01	DRB1*04:01	DQB1*03:02
	CONTIG2	A*68:01	B*35:03	C*12:03	DRB1*08:01	-
HC0174	CONTIG1	A*24:02	B*13:02	C*03:04	DRB1*07:01	DQB1*02:02
	CONTIG2	-	B*40:01	C*06:02	DRB1*13:02	DQB1*06:04
HC0175	CONTIG1	A*01:01	B*08:01	C*07:01	DRB1*01:01	DQB1*05:01

	CONTIG2	A*24:02	B*38:01	C*12:03	-	-
HC0176	CONTIG1	A*02:01	B*18:01	C*04:01	DRB1*01:01	DQB1*03:01
	CONTIG2	A*25:01	B*35:01	C*07:01	DRB1*11:04	DQB1*05:01
HC0177	CONTIG1	A*01:01	B*08:01	C*01:02	DRB1*03:01	DQB1*02:01
	CONTIG2	A*02:01	B*51:01	C*07:01	DRB1*11:01	DQB1*03:01
HC0178	CONTIG1	A*24:02	B*15:17	C*05:01	DRB1*13:01	DQB1*06:03
	CONTIG2	A*31:01	B*51:01	C*07:01	DRB1*13:02	DQB1*06:04
HC0179	CONTIG1	A*25:01	B*18:01	C*12:03	DRB1*04:01	DQB1*03:02
	CONTIG2	-	-	-	DRB1*15:01	DQB1*06:02
HC0180	CONTIG1	A*02:01	B*27:05	C*02:02	DRB1*01:02	DQB1*05:01
	CONTIG2	A*03:01	B*44:02	C*05:01	-	-
HC0181	CONTIG1	A*01:01	B*35:02	C*04:01	DRB1*07:01	DQB1*03:01
	CONTIG2	A*23:01	B*44:03	C*06:02	DRB1*11:04	DQB1*03:03
HC0182	CONTIG1	A*02:01	B*35:03	C*04:01	DRB1*08:01	DQB1*03:01
	CONTIG2	A*03:01	B*51:01	C*07:01	DRB1*11:04	DQB1*04:02
HC0183	CONTIG1	A*03:01	B*18:44	C*12:03	DRB1*01:01	DQB1*05:01
	CONTIG2	A*25:01	B*51:01	-	DRB1*15:01	DQB1*06:02
HC0184	CONTIG1	A*03:01	B*07:02	C*06:02	DRB1*07:01	DQB1*02:02
	CONTIG2	A*24:02	B*13:02	C*07:02	DRB1*11:01	DQB1*03:01
HC0185	CONTIG1	A*11:01	B*35:03	C*07:04	DRB1*07:01	DQB1*02:02
	CONTIG2	A*26:01	B*51:01	C*12:03	-	DQB1*03:03
HC0186	CONTIG1	A*03:01	B*13:02	C*06:02	DRB1*11:04	DQB1*03:01
	CONTIG2	A*30:01	-	-	DRB1*16:01	DQB1*05:02
HC0187	CONTIG1	A*01:01	B*37:01	C*06:02	DRB1*11:04	DQB1*03:01
	CONTIG2	A*02:01	B*51:01	C*07:01	DRB1*13:03	-
HC0188	CONTIG1	A*11:01	B*07:02	C*07:02	DRB1*13:01	DQB1*06:02
	CONTIG2	A*24:02	B*44:02	C*07:04	DRB1*15:01	DQB1*06:03
HC0189	CONTIG1	A*02:01	B*15:01	C*03:03	DRB1*04:04	DQB1*03:02
	CONTIG2	A*24:02	B*52:01	C*12:02	DRB1*15:02	DQB1*06:01
HC0190	CONTIG1	A*02:01	B*38:01	C*07:04	DRB1*04:02	DQB1*03:02
	CONTIG2	A*03:01	B*44:27	C*12:03	DRB1*16:01	DQB1*05:02
HC0191	CONTIG1	A*01:01	B*13:02	C*06:02	DRB1*07:01	DQB1*02:02
	CONTIG2	A*03:01	B*18:01	C*07:01	DRB1*13:01	DQB1*06:03
HC0192	CONTIG1	A*03:01	B*35:03	C*03:04	DRB1*01:01	DQB1*03:02
	CONTIG2	A*68:01	B*40:01	C*04:01	DRB1*08:01	DQB1*05:01
HC0193	CONTIG1	A*02:01	B*27:05	C*02:02	DRB1*08:01	DQB1*04:02
	CONTIG2	-	B*51:01	C*14:02	DRB1*15:01	DQB1*06:02
HC0194	CONTIG1	A*11:01	B*07:02	C*07:02	DRB1*13:02	DQB1*06:01
	CONTIG2	A*24:02	B*52:01	C*12:02	DRB1*15:02	DQB1*06:04
HC0195	CONTIG1	A*02:01	B*13:02	C*02:02	DRB1*01:01	DQB1*02:02
	CONTIG2	A*03:01	B*27:05	C*06:02	DRB1*07:01	DQB1*05:01
HC0196	CONTIG1	A*01:01	B*35:08	C*03:04	DRB1*04:04	DQB1*03:02
	CONTIG2	A*11:01	B*40:01	C*06:02	DRB1*07:01	DQB1*03:03
HC0197	CONTIG1	A*03:01	B*39:06	C*07:01	DRB1*01:01	DQB1*05:01
	CONTIG2	A*26:01	B*49:01	C*07:02	-	DQB1*05:04
HC0198	CONTIG1	A*02:01	B*07:02	C*07:01	DRB1*11:01	DQB1*03:01
	CONTIG2	A*32:01	B*49:01	C*07:02	DRB1*14:54	DQB1*05:03
HC0199	CONTIG1	A*01:01	B*35:03	C*04:01	DRB1*07:01	DQB1*03:03
	CONTIG2	A*03:01	B*57:01	C*06:02	DRB1*08:01	DQB1*04:02
HC0200	CONTIG1	A*02:01	B*15:01	C*03:04	DRB1*04:01	DQB1*03:02

	CONTIG2	A*02:05	B*58:01	C*07:18	DRB1*13:02	DQB1*06:09
HC0201	CONTIG1	A*02:01	B*51:01	C*05:01	DRB1*13:01	DQB1*06:02
	CONTIG2	A*26:01	-	C*14:02	DRB1*15:01	DQB1*06:03
HC0202	CONTIG1	A*02:01	B*07:02	C*04:01	DRB1*12:01	DQB1*03:01
	CONTIG2	A*24:02	B*35:03	C*07:02	DRB1*13:01	DQB1*06:03
HC0203	CONTIG1	A*11:01	B*27:05	C*02:02	DRB1*11:01	DQB1*03:01
	CONTIG2	A*24:02	-	-	DRB1*13:01	DQB1*06:03
HC0204	CONTIG1	A*25:01	B*13:02	C*06:02	DRB1*07:01	DQB1*02:02
	CONTIG2	A*30:01	B*18:01	C*12:03	DRB1*15:01	DQB1*06:02
HC0205	CONTIG1	A*24:02	B*38:01	C*06:02	DRB1*07:01	DQB1*03:03
	CONTIG2	A*32:01	B*57:01	C*12:03	DRB1*13:01	DQB1*06:03
HC0206	CONTIG1	A*02:01	B*50:01	C*01:02	DRB1*01:01	DQB1*02:01
	CONTIG2	A*23:01	B*56:01	C*06:02	DRB1*03:01	DQB1*05:01
HC0207	CONTIG1	A*02:01	B*18:01	C*05:01	DRB1*01:01	DQB1*03:01
	CONTIG2	A*03:01	B*44:02	C*07:01	DRB1*11:01	DQB1*05:01
HC0208	CONTIG1	A*02:01	B*44:02	C*07:04	DRB1*04:04	DQB1*03:01
	CONTIG2	A*68:01	B*51:01	C*15:02	DRB1*11:01	DQB1*03:02
HC0209	CONTIG1	A*01:01	B*08:01	C*07:01	DRB1*03:01	DQB1*02:01
	CONTIG2	-	-	-	-	-
HC0210	CONTIG1	A*02:01	B*27:02	C*02:02	DRB1*11:04	DQB1*03:01
	CONTIG2	-	B*44:03	C*04:01	DRB1*16:01	DQB1*05:02
HC0211	CONTIG1	A*01:01	B*08:01	C*02:02	DRB1*15:01	DQB1*05:02
	CONTIG2	A*02:01	B*27:02	C*07:01	DRB1*16:01	DQB1*06:02
HC0212	CONTIG1	A*02:01	B*07:02	C*07:01	DRB1*07:01	DQB1*03:01
	CONTIG2	A*68:01	B*57:01	C*07:02	DRB1*11:01	DQB1*03:03
HC0213	CONTIG1	A*03:01	B*18:01	C*04:01	DRB1*01:01	DQB1*03:01
	CONTIG2	A*25:01	B*35:01	C*07:01	DRB1*11:04	DQB1*05:01
HC0214	CONTIG1	A*02:01	B*08:01	C*07:01	DRB1*03:01	DQB1*02:01
	CONTIG2	-	B*39:24	-	DRB1*13:03	DQB1*03:01
HC0215	CONTIG1	A*03:01	B*07:02	C*04:01	DRB1*01:01	DQB1*05:01
	CONTIG2	-	B*35:01	C*07:02	DRB1*15:01	DQB1*06:02
HC0216	CONTIG1	A*03:01	B*18:01	C*04:01	DRB1*01:01	DQB1*02:02
	CONTIG2	A*25:01	B*35:01	C*12:03	DRB1*04:04	DQB1*05:01
HC0217	CONTIG1	A*02:01	B*15:01	C*01:02	DRB1*01:01	DQB1*05:01
	CONTIG2	-	B*27:05	C*03:03	DRB1*13:01	DQB1*06:03
HC0218	CONTIG1	A*03:01	B*13:02	C*04:01	DRB1*01:01	DQB1*02:02
	CONTIG2	A*11:01	B*35:01	C*06:02	DRB1*07:01	DQB1*05:01
HC0219	CONTIG1	A*02:01	B*13:02	C*05:01	DRB1*01:01	DQB1*05:01
	CONTIG2	-	B*44:02	C*06:02	DRB1*15:01	DQB1*06:02
HC0220	CONTIG1	A*03:01	B*35:01	C*04:01	DRB1*01:01	DQB1*05:01
	CONTIG2	A*66:01	B*38:01	C*12:03	DRB1*13:01	DQB1*06:03
HC0221	CONTIG1	A*24:02	B*07:02	C*07:02	DRB1*13:03	DQB1*03:01
	CONTIG2	A*66:01	B*41:02	C*17:03	DRB1*15:01	DQB1*06:02
HC0222	CONTIG1	A*01:01	B*27:05	C*01:02	DRB1*04:04	DQB1*03:01
	CONTIG2	A*24:02	B*35:03	C*04:01	DRB1*11:12	DQB1*03:02
HC0223	CONTIG1	A*02:01	B*44:27	C*07:04	DRB1*11:03	DQB1*03:01
	CONTIG2	-	B*51:01	C*15:04	DRB1*16:01	DQB1*05:02
HC0224	CONTIG1	A*02:01	B*14:02	C*08:02	DRB1*04:08	DQB1*02:02
	CONTIG2	A*68:01	-	-	DRB1*07:01	DQB1*03:01
HC0225	CONTIG1	A*01:01	B*07:02	C*07:01	DRB1*03:01	DQB1*02:01

	CONTIG2	A*24:02	B*08:01	C*07:02	DRB1*11:04	DQB1*03:01
HC0226	CONTIG1	A*32:01	B*15:17	C*07:01	DRB1*04:04	DQB1*03:02
	CONTIG2	A*69:01	B*51:01	C*15:02	DRB1*13:02	DQB1*06:04
HC0227	CONTIG1	A*02:01	B*51:01	C*02:02	DRB1*07:01	DQB1*03:01
	CONTIG2	A*31:01	-	C*15:02	DRB1*12:01	DQB1*03:03
HC0228	CONTIG1	A*02:01	B*07:02	C*04:01	DRB1*01:01	DQB1*05:01
	CONTIG2	A*03:01	B*35:01	C*07:02	DRB1*15:01	DQB1*06:02
HC0229	CONTIG1	A*02:01	B*40:02	C*03:04	DRB1*08:04	DQB1*04:02
	CONTIG2	A*68:01	B*58:01	C*07:18	DRB1*14:54	DQB1*05:03
HC0230	CONTIG1	A*01:01	B*27:05	C*05:01	DRB1*01:01	DQB1*05:01
	CONTIG2	-	B*38:01	C*12:03	DRB1*13:01	DQB1*06:03
HC0231	CONTIG1	A*02:01	B*40:01	C*01:02	DRB1*01:01	DQB1*05:01
	CONTIG2	A*11:01	B*56:01	C*03:04	DRB1*13:02	DQB1*06:04
HC0232	CONTIG1	A*01:01	B*08:01	C*06:02	DRB1*03:01	DQB1*02:01
	CONTIG2	A*02:01	B*35:02	C*07:01	DRB1*11:04	DQB1*03:01
HC0233	CONTIG1	A*01:01	B*08:01	C*06:02	DRB1*03:01	DQB1*02:01
	CONTIG2	A*02:01	B*13:02	C*07:01	DRB1*09:01	DQB1*03:03
HC0234	CONTIG1	A*02:01	B*15:01	C*02:02	DRB1*04:01	DQB1*03:02
	CONTIG2	-	B*27:02	C*03:04	DRB1*16:01	DQB1*05:02
HC0235	CONTIG1	A*01:01	B*35:01	C*04:01	DRB1*01:01	DQB1*03:03
	CONTIG2	A*25:01	B*57:01	C*06:02	DRB1*07:01	DQB1*05:01
HC0236	CONTIG1	A*01:01	B*44:03	C*06:02	DRB1*04:01	DQB1*03:01
	CONTIG2	A*02:01	B*50:01	C*16:02	DRB1*04:02	DQB1*03:02
HC0237	CONTIG1	A*02:01	B*35:08	C*04:01	DRB1*04:03	DQB1*03:02
	CONTIG2	A*03:02	B*57:01	C*06:02	DRB1*16:01	DQB1*05:02
HC0238	CONTIG1	A*11:01	B*38:01	C*03:03	DRB1*13:01	DQB1*05:02
	CONTIG2	A*24:02	B*55:01	C*12:03	DRB1*16:01	DQB1*06:03
HC0239	CONTIG1	A*03:01	B*44:05	C*02:02	DRB1*12:01	DQB1*03:01
	CONTIG2	-	B*51:01	C*03:04	DRB1*16:01	DQB1*05:02
HC0240	CONTIG1	A*01:01	B*08:01	C*07:01	DRB1*03:01	DQB1*02:01
	CONTIG2	-	B*51:07	C*14:02	DRB1*11:01	DQB1*03:01
HC0241	CONTIG1	A*03:01	B*15:01	C*03:03	DRB1*04:04	DQB1*02:02
	CONTIG2	A*23:01	B*44:03	C*04:01	DRB1*07:01	DQB1*03:02
HC0242	CONTIG1	A*03:01	B*07:02	C*06:02	DRB1*07:01	DQB1*02:02
	CONTIG2	-	B*13:02	C*07:02	DRB1*15:01	DQB1*06:02
HC0243	CONTIG1	A*03:01	B*35:03	C*04:01	DRB1*01:01	DQB1*05:01
	CONTIG2	A*24:02	B*51:01	C*12:03	DRB1*15:01	DQB1*06:02
HC0244	CONTIG1	A*01:03	B*35:03	C*04:01	DRB1*11:01	DQB1*03:01
	CONTIG2	A*32:01	B*73:01	C*15:05	-	-
HC0245	CONTIG1	A*01:01	B*08:01	C*04:01	DRB1*01:01	DQB1*02:01
	CONTIG2	A*03:01	B*35:01	C*07:01	DRB1*03:01	DQB1*05:01
HC0246	CONTIG1	A*01:01	B*08:01	C*03:03	DRB1*03:01	DQB1*02:01
	CONTIG2	A*24:02	B*44:02	C*07:01	DRB1*11:03	DQB1*03:01
HC0247	CONTIG1	A*02:01	B*15:01	C*01:02	DRB1*01:01	DQB1*05:01
	CONTIG2	A*26:01	B*27:05	C*03:03	DRB1*15:01	DQB1*06:02
HC0248	CONTIG1	A*23:01	B*18:01	C*04:01	DRB1*11:01	DQB1*03:01
	CONTIG2	A*25:01	B*44:03	C*12:03	DRB1*15:01	DQB1*06:02
HC0249	CONTIG1	A*02:01	B*07:02	C*02:02	DRB1*01:01	DQB1*03:03
	CONTIG2	A*24:02	B*27:05	C*07:02	DRB1*09:01	DQB1*05:01
HC0250	CONTIG1	A*01:01	B*08:01	C*07:01	DRB1*03:01	DQB1*02:01

	CONTIG2	A*31:01	B*39:01	C*12:03	DRB1*12:01	DQB1*03:01
HC0251	CONTIG1	A*03:01	B*35:02	C*04:01	DRB1*08:01	DQB1*03:01
	CONTIG2	A*24:02	B*35:03	-	DRB1*11:04	DQB1*04:02
HC0252	CONTIG1	A*01:01	B*07:02	C*07:01	DRB1*13:01	DQB1*06:03
	CONTIG2	A*11:01	B*08:01	C*07:02	DRB1*15:01	DQB1*06:13
HC0253	CONTIG1	A*02:01	B*18:01	C*02:02	DRB1*01:01	DQB1*05:01
	CONTIG2	A*25:01	B*27:05	C*12:03	DRB1*15:01	DQB1*06:02
HC0254	CONTIG1	A*29:01	B*07:02	C*07:02	DRB1*10:01	DQB1*05:01
	CONTIG2	A*68:01	B*07:05	C*15:05	DRB1*15:01	DQB1*06:02
HC0255	CONTIG1	A*02:01	B*18:01	C*02:02	DRB1*01:01	DQB1*02:01
	CONTIG2	A*03:01	B*27:05	C*05:01	DRB1*03:01	DQB1*05:01
HC0256	CONTIG1	A*02:01	B*13:02	C*06:02	DRB1*03:01	DQB1*02:01
	CONTIG2	A*32:01	B*50:01	-	DRB1*07:01	DQB1*02:02
HC0257	CONTIG1	A*01:01	B*08:01	C*07:01	DRB1*03:01	DQB1*02:01
	CONTIG2	A*25:01	B*18:01	C*12:03	DRB1*13:01	DQB1*06:03
HC0258	CONTIG1	A*02:01	B*41:02	C*04:01	DRB1*07:01	DQB1*02:02
	CONTIG2	A*24:02	B*44:03	C*17:03	DRB1*13:03	DQB1*03:01
HC0259	CONTIG1	A*24:02	B*07:02	C*07:02	DRB1*01:01	DQB1*05:01
	CONTIG2	A*25:01	B*18:01	C*12:03	DRB1*15:01	DQB1*06:02
HC0260	CONTIG1	A*24:02	B*07:02	C*01:02	DRB1*01:01	DQB1*05:01
	CONTIG2	A*25:01	B*18:01	C*12:03	DRB1*15:01	DQB1*06:02
HC0261	CONTIG1	A*02:01	B*40:01	C*03:04	DRB1*03:01	DQB1*02:01
	CONTIG2	A*03:01	B*44:03	C*04:01	DRB1*07:01	DQB1*02:02
HC0262	CONTIG1	A*11:01	B*41:01	C*15:02	DRB1*03:01	DQB1*02:01
	CONTIG2	A*29:01	B*51:01	C*17:01	DRB1*04:04	DQB1*03:02
HC0263	CONTIG1	A*02:01	B*35:01	C*03:04	DRB1*01:01	DQB1*05:01
	CONTIG2	A*03:01	B*40:01	C*04:01	DRB1*13:02	DQB1*06:04
HC0264	CONTIG1	A*03:01	B*44:03	C*04:01	DRB1*03:01	DQB1*02:01
	CONTIG2	A*23:01	B*51:01	C*16:01	DRB1*15:01	DQB1*06:02
HC0265	CONTIG1	A*11:01	B*18:01	C*12:03	DRB1*13:01	DQB1*06:02
	CONTIG2	A*25:01	B*38:01	-	DRB1*15:01	DQB1*06:03
HC0266	CONTIG1	A*02:01	B*13:02	C*03:04	DRB1*01:01	DQB1*02:02
	CONTIG2	-	B*27:05	C*06:02	DRB1*07:01	DQB1*05:01
HC0267	CONTIG1	A*01:01	B*08:01	C*02:02	DRB1*01:02	DQB1*02:01
	CONTIG2	A*33:01	B*44:03	C*07:01	DRB1*03:01	DQB1*05:01
HC0268	CONTIG1	A*02:01	B*07:02	C*03:04	DRB1*04:01	DQB1*03:01
	CONTIG2	A*03:01	B*15:01	C*07:02	DRB1*11:01	DQB1*03:02
HC0269	CONTIG1	A*25:01	B*14:02	C*02:02	DRB1*01:01	DQB1*05:01
	CONTIG2	A*32:01	B*27:05	C*08:02	DRB1*01:02	-
HC0270	CONTIG1	A*01:01	B*08:01	C*07:01	DRB1*01:01	DQB1*05:01
	CONTIG2	A*02:01	B*51:01	C*14:02	DRB1*15:01	DQB1*06:02
HC0271	CONTIG1	A*02:01	B*07:02	C*03:04	DRB1*13:02	DQB1*06:02
	CONTIG2	-	B*40:01	C*07:02	DRB1*15:01	DQB1*06:04
HC0272	CONTIG1	A*01:01	B*08:01	C*07:01	DRB1*03:01	DQB1*02:01
	CONTIG2	A*25:01	B*18:01	C*12:03	DRB1*12:01	DQB1*03:01
HC0273	CONTIG1	A*02:83N	B*07:02	C*07:02	DRB1*04:01	DQB1*03:02
	CONTIG2	A*68:01	B*44:02	C*07:04	DRB1*10:01	DQB1*05:01
HC0274	CONTIG1	A*01:01	B*13:02	C*06:02	DRB1*04:01	DQB1*02:02
	CONTIG2	A*24:02	B*37:01	-	DRB1*07:01	DQB1*03:02
HC0275	CONTIG1	A*11:01	B*13:02	C*05:01	DRB1*07:01	DQB1*02:02

	CONTIG2	A*24:02	B*44:02	C*06:02	DRB1*12:01	DQB1*03:01
HC0276	CONTIG1	A*02:01	B*07:02	C*07:02	DRB1*13:01	DQB1*06:03
	CONTIG2	A*26:01	B*38:01	C*12:03	DRB1*15:01	-
HC0277	CONTIG1	A*24:02	B*07:02	C*07:02	DRB1*01:01	DQB1*05:01
	CONTIG2	A*25:01	B*18:01	C*12:03	DRB1*15:01	DQB1*06:02
HC0278	CONTIG1	A*01:01	B*08:01	C*02:02	DRB1*04:01	DQB1*03:01
	CONTIG2	A*32:01	B*40:02	C*07:01	DRB1*11:01	DQB1*03:02
HC0279	CONTIG1	A*02:01	B*18:01	C*02:02	DRB1*07:01	DQB1*02:02
	CONTIG2	A*25:01	B*27:02	C*12:03	DRB1*08:01	DQB1*04:02
HC0280	CONTIG1	A*02:01	B*35:01	C*04:01	DRB1*04:01	DQB1*03:01
	CONTIG2	A*03:01	B*44:02	C*05:01	DRB1*16:01	DQB1*05:02
HC0281	CONTIG1	A*03:01	B*07:02	C*04:01	DRB1*11:01	DQB1*03:01
	CONTIG2	-	B*35:03	C*07:02	DRB1*15:01	DQB1*06:02
HC0282	CONTIG1	A*03:01	B*35:03	C*04:01	DRB1*08:01	DQB1*03:01
	CONTIG2	A*23:01	B*44:03	-	DRB1*11:01	DQB1*04:02
HC0283	CONTIG1	A*03:01	B*40:02	C*02:02	DRB1*11:01	DQB1*03:01
	CONTIG2	A*11:01	B*51:01	C*16:02	-	-
HC0284	CONTIG1	A*03:01	B*07:02	C*06:02	DRB1*07:01	DQB1*02:02
	CONTIG2	A*24:02	B*13:02	C*07:02	DRB1*15:01	DQB1*06:02
HC0285	CONTIG1	A*01:01	B*37:01	C*06:02	DRB1*07:01	DQB1*03:01
	CONTIG2	A*02:01	B*57:01	-	DRB1*11:04	DQB1*03:03
HC0286	CONTIG1	A*02:01	B*13:02	C*06:02	DRB1*07:01	DQB1*02:02
	CONTIG2	A*24:02	B*18:01	C*07:01	DRB1*11:04	DQB1*03:01
HC0287	CONTIG1	A*02:01	B*07:02	C*06:02	DRB1*07:01	DQB1*03:03
	CONTIG2	A*24:02	B*57:01	C*07:02	DRB1*15:01	DQB1*06:02
HC0288	CONTIG1	A*02:01	B*47:02	C*06:02	DRB1*01:01	DQB1*05:01
	CONTIG2	A*03:01	B*57:01	-	DRB1*15:01	DQB1*06:02
HC0289	CONTIG1	A*01:01	B*18:01	C*07:01	DRB1*07:01	DQB1*02:02
	CONTIG2	A*02:01	B*38:01	C*12:03	DRB1*11:04	DQB1*03:01
HC0290	CONTIG1	A*02:05	B*18:01	C*04:01	DRB1*11:01	DQB1*03:01
	CONTIG2	A*25:01	B*35:03	C*12:03	DRB1*15:01	DQB1*06:02
HC0291	CONTIG1	A*01:01	B*08:01	C*07:01	DRB1*03:01	DQB1*02:01
	CONTIG2	A*02:01	B*39:01	C*12:03	DRB1*12:01	DQB1*03:01
HC0292	CONTIG1	A*02:01	B*18:01	C*12:03	DRB1*04:01	DQB1*03:01
	CONTIG2	A*02:17	-	-	DRB1*11:04	DQB1*03:02
HC0293	CONTIG1	A*03:01	B*07:02	C*01:02	DRB1*01:01	DQB1*05:01
	CONTIG2	-	B*51:01	C*07:02	DRB1*15:01	DQB1*06:02
HC0294	CONTIG1	A*01:01	B*08:01	C*01:02	DRB1*03:01	DQB1*02:01
	CONTIG2	A*24:02	B*51:01	C*07:01	DRB1*15:01	DQB1*06:02
HC0295	CONTIG1	A*24:03	B*27:05	C*02:02	DRB1*03:01	DQB1*02:01
	CONTIG2	A*32:01	B*50:01	C*15:04	DRB1*09:01	DQB1*03:03
HC0296	CONTIG1	A*24:02	B*18:01	C*07:01	DRB1*01:01	DQB1*03:01
	CONTIG2	A*25:01	B*18:01	C*12:03	DRB1*11:04	DQB1*05:01
HC0297	CONTIG1	A*02:01	B*27:05	C*02:02	DRB1*04:08	DQB1*03:01
	CONTIG2	A*24:02	B*35:03	C*12:03	DRB1*11:01	DQB1*03:04
HC0298	CONTIG1	A*02:01	B*27:05	C*02:02	DRB1*01:01	DQB1*03:01
	CONTIG2	A*03:01	B*35:03	C*04:01	DRB1*11:01	DQB1*05:01
HC0299	CONTIG1	A*02:01	B*44:02	C*05:01	DRB1*01:01	DQB1*05:01
	CONTIG2	A*02:17	B*51:01	C*15:02	DRB1*15:01	DQB1*06:02
HC0300	CONTIG1	A*02:01	B*15:09	C*07:04	DRB1*11:01	DQB1*03:01

	CONTIG2	A*24:02	B*41:02	C*17:03	DRB1*13:03	-
HC0301	CONTIG1	A*02:01	B*18:01	C*02:02	DRB1*15:01	DQB1*05:02
	CONTIG2	A*25:01	B*27:02	C*12:03	DRB1*16:01	DQB1*06:02
HC0302	CONTIG1	A*02:01	B*40:02	C*02:02	DRB1*07:01	DQB1*02:02
	CONTIG2	A*23:01	B*58:01	C*07:18	DRB1*11:01	DQB1*03:01
HC0303	CONTIG1	A*01:01	B*07:02	C*02:02	DRB1*15:01	DQB1*06:02
	CONTIG2	A*24:02	B*27:05	C*07:02	-	-
HC0304	CONTIG1	A*02:01	B*38:01	C*12:03	DRB1*04:02	DQB1*03:02
	CONTIG2	A*26:01	B*44:03	C*16:02	DRB1*15:01	DQB1*06:03
HC0305	CONTIG1	A*02:01	B*07:02	C*07:01	DRB1*11:01	DQB1*03:01
	CONTIG2	A*03:01	B*18:01	C*07:02	-	-
HC0306	CONTIG1	A*03:01	B*07:02	C*07:02	DRB1*13:02	DQB1*06:02
	CONTIG2	A*26:01	B*14:01	C*08:02	DRB1*15:01	DQB1*06:04
HC0307	CONTIG1	A*02:09	B*18:01	C*04:01	DRB1*11:04	DQB1*03:01
	CONTIG2	A*24:02	B*35:03	C*12:03	DRB1*12:01	DQB1*03:01
HC0308	CONTIG1	A*02:01	B*14:02	C*03:04	DRB1*01:02	DQB1*03:02
	CONTIG2	-	B*15:01	C*08:02	DRB1*04:01	DQB1*05:01
HC0309	CONTIG1	A*02:01	B*15:01	C*02:02	DRB1*11:03	DQB1*03:01
	CONTIG2	A*11:01	B*27:02	C*03:03	DRB1*16:01	DQB1*05:02
HC0310	CONTIG1	A*02:01	B*07:02	C*07:01	DRB1*03:01	DQB1*02:01
	CONTIG2	A*23:01	B*18:01	C*07:02	DRB1*12:01	DQB1*03:01
HC0311	CONTIG1	A*01:01	B*13:02	C*03:04	DRB1*07:01	DQB1*02:02
	CONTIG2	A*66:01	B*40:01	C*06:02	DRB1*13:02	DQB1*06:04
HC0312	CONTIG1	A*02:01	B*07:02	C*07:02	DRB1*13:03	DQB1*03:01
	CONTIG2	-	B*41:02	C*17:03	DRB1*15:01	DQB1*06:02
HC0313	CONTIG1	A*02:01	B*18:01	C*04:01	DRB1*04:01	DQB1*03:02
	CONTIG2	A*25:01	B*35:03	C*12:03	DRB1*15:01	DQB1*06:02
HC0314	CONTIG1	A*01:01	B*07:02	C*04:01	DRB1*11:04	DQB1*03:01
	CONTIG2	A*03:01	B*35:02	C*07:02	DRB1*15:01	DQB1*06:02
HC0315	CONTIG1	A*02:01	B*07:02	C*07:02	DRB1*04:08	DQB1*03:01
	CONTIG2	A*03:01	B*38:01	C*12:03	DRB1*11:01	DQB1*03:04
HC0316	CONTIG1	A*01:01	B*08:01	C*07:01	DRB1*03:01	DQB1*02:01
	CONTIG2	A*02:17	B*51:01	C*14:02	DRB1*07:01	DQB1*02:02
HC0317	CONTIG1	A*24:02	B*07:02	C*03:04	DRB1*14:03	DQB1*03:01
	CONTIG2	-	B*40:02	C*07:02	DRB1*15:01	DQB1*06:02
HC0318	CONTIG1	A*02:01	B*44:02	C*05:01	DRB1*04:01	DQB1*03:01
	CONTIG2	-	B*52:01	C*12:02	DRB1*15:02	DQB1*06:01
HC0319	CONTIG1	A*01:01	B*08:01	C*02:02	DRB1*03:01	DQB1*02:01
	CONTIG2	A*24:02	B*44:05	C*07:01	DRB1*11:04	DQB1*03:01
HC0320	CONTIG1	A*02:01	B*15:01	C*03:03	DRB1*04:01	DQB1*03:02
	CONTIG2	A*11:01	B*55:01	C*03:04	DRB1*13:01	DQB1*06:03
HC0321	CONTIG1	A*01:03	B*14:02	C*08:02	DRB1*01:02	DQB1*05:01
	CONTIG2	A*33:01	B*73:01	C*15:05	DRB1*13:02	DQB1*06:04
HC0322	CONTIG1	A*01:01	B*08:01	C*07:01	DRB1*03:01	DQB1*02:01
	CONTIG2	A*25:01	B*39:01	C*12:03	DRB1*11:01	DQB1*03:01
HC0323	CONTIG1	A*03:01	B*08:01	C*07:01	DRB1*03:01	DQB1*02:01
	CONTIG2	A*25:01	B*18:01	C*12:03	DRB1*15:01	DQB1*06:02
HC0324	CONTIG1	A*02:01	B*08:01	C*03:03	DRB1*01:01	DQB1*05:01
	CONTIG2	-	B*35:01	C*07:01	-	-
HC0325	CONTIG1	A*24:02	B*18:01	C*04:01	DRB1*08:01	DQB1*03:01

	CONTIG2	A*25:01	B*35:02	C*12:03	DRB1*11:04	DQB1*04:02
HC0326	CONTIG1	A*01:01	B*08:01	C*05:01	DRB1*03:01	DQB1*02:01
	CONTIG2	A*02:01	B*44:02	C*07:01	DRB1*12:01	DQB1*03:01
HC0327	CONTIG1	A*01:01	B*13:02	C*06:02	DRB1*11:02	DQB1*03:01
	CONTIG2	A*02:01	B*39:01	C*07:02	DRB1*16:01	DQB1*05:02
HC0328	CONTIG1	A*01:01	B*08:01	C*03:04	DRB1*03:01	DQB1*02:01
	CONTIG2	A*30:01	B*40:01	C*07:01	DRB1*11:01	DQB1*03:01
HC0329	CONTIG1	A*02:01	B*14:02	C*07:01	DRB1*01:02	DQB1*03:01
	CONTIG2	A*23:01	B*49:01	C*08:02	DRB1*11:04	DQB1*05:01
HC0330	CONTIG1	A*01:01	B*51:01	C*12:02	DRB1*04:01	DQB1*03:02
	CONTIG2	A*25:01	B*52:01	C*12:03	DRB1*15:02	DQB1*06:01
HC0331	CONTIG1	A*01:01	B*08:01	C*04:01	DRB1*03:01	DQB1*02:01
	CONTIG2	A*23:01	B*44:03	C*07:01	DRB1*04:01	DQB1*03:02
HC0332	CONTIG1	A*01:01	B*08:01	C*07:01	DRB1*03:01	DQB1*02:01
	CONTIG2	-	B*52:01	C*12:02	DRB1*15:02	DQB1*06:01
HC0333	CONTIG1	A*24:02	B*40:02	C*03:03	DRB1*11:01	DQB1*03:01
	CONTIG2	A*26:01	B*44:02	C*03:04	DRB1*11:03	-
HC0334	CONTIG1	A*24:02	B*13:02	C*06:02	DRB1*01:01	DQB1*02:02
	CONTIG2	A*26:01	B*38:01	C*12:03	DRB1*07:01	DQB1*05:04
HC0335	CONTIG1	A*01:01	B*08:01	C*04:01	DRB1*03:01	DQB1*02:01
	CONTIG2	A*03:01	B*35:03	C*07:01	DRB1*08:01	DQB1*04:02
HC0336	CONTIG1	A*02:01	B*27:05	C*02:02	DRB1*01:01	DQB1*03:01
	CONTIG2	A*26:01	B*55:01	C*03:03	DRB1*11:03	DQB1*05:01
HC0337	CONTIG1	A*02:01	B*07:02	C*02:02	DRB1*01:01	DQB1*05:01
	CONTIG2	A*24:02	B*27:05	C*07:02	DRB1*15:01	DQB1*06:02
HC0338	CONTIG1	A*24:02	B*13:02	C*06:02	DRB1*01:02	DQB1*05:01
	CONTIG2	A*68:01	B*14:02	C*08:02	DRB1*10:01	-
HC0339	CONTIG1	A*03:01	B*07:02	C*07:02	DRB1*04:04	DQB1*03:02
	CONTIG2	-	B*39:06	C*07:02	DRB1*15:01	DQB1*06:02
HC0340	CONTIG1	A*24:02	B*18:01	C*04:01	DRB1*11:04	DQB1*03:01
	CONTIG2	A*29:02	B*44:03	C*12:03	DRB1*13:02	DQB1*06:09
HC0341	CONTIG1	A*01:01	B*08:01	C*07:01	DRB1*03:01	DQB1*02:01
	CONTIG2	A*02:01	B*44:27	C*07:04	DRB1*16:01	DQB1*05:02
HC0342	CONTIG1	A*03:01	B*35:03	C*04:01	DRB1*08:01	DQB1*03:01
	CONTIG2	A*66:01	B*41:02	C*17:03	DRB1*13:03	DQB1*04:02
HC0343	CONTIG1	A*03:01	B*51:01	C*01:02	DRB1*15:01	DQB1*05:02
	CONTIG2	A*25:01	-	C*14:02	DRB1*16:01	DQB1*06:02
HC0344	CONTIG1	A*03:01	B*07:02	C*07:02	DRB1*08:01	DQB1*04:02
	CONTIG2	A*24:02	-	-	DRB1*15:01	DQB1*06:02
HC0345	CONTIG1	A*02:01	B*14:02	C*08:02	DRB1*13:03	DQB1*03:01
	CONTIG2	A*68:02	B*18:01	C*12:03	DRB1*15:01	DQB1*06:02
HC0346	CONTIG1	A*03:01	B*35:01	C*04:01	DRB1*01:01	DQB1*05:01
	CONTIG2	A*11:01	B*35:03	-	-	-
HC0347	CONTIG1	A*01:01	B*08:01	C*03:04	DRB1*04:01	DQB1*03:02
	CONTIG2	A*25:01	B*15:01	C*07:01	DRB1*13:01	DQB1*06:03
HC0348	CONTIG1	A*01:01	B*35:02	C*01:02	DRB1*11:04	DQB1*03:01
	CONTIG2	A*32:01	B*51:01	C*04:01	DRB1*12:01	-
HC0349	CONTIG1	A*02:01	B*27:05	C*02:02	DRB1*08:01	DQB1*03:03
	CONTIG2	A*32:01	B*27:05	-	DRB1*09:01	DQB1*04:02
HC0350	CONTIG1	A*24:02	B*18:01	C*04:01	DRB1*07:01	DQB1*02:02

	CONTIG2	A*24:03	B*44:03	C*12:03	DRB1*11:04	DQB1*03:01
HC0351	CONTIG1	A*01:01	B*44:02	C*04:01	DRB1*11:01	DQB1*03:01
	CONTIG2	A*23:01	B*44:03	C*05:01	DRB1*11:04	-
HC0352	CONTIG1	A*01:01	B*08:01	C*07:01	DRB1*01:02	DQB1*02:01
	CONTIG2	A*03:01	B*14:02	C*08:02	DRB1*03:01	DQB1*05:01
HC0353	CONTIG1	A*02:01	B*40:01	C*03:04	DRB1*13:01	DQB1*06:02
	CONTIG2	A*03:01	B*51:01	C*15:02	DRB1*15:01	DQB1*06:03
HC0354	CONTIG1	A*01:01	B*08:01	C*07:01	DRB1*03:01	DQB1*02:01
	CONTIG2	A*02:01	B*41:02	C*17:03	DRB1*13:03	DQB1*03:01
HC0355	CONTIG1	A*02:01	B*27:05	C*02:02	DRB1*11:01	DQB1*03:01
	CONTIG2	A*29:01	B*51:01	C*15:02	DRB1*13:01	DQB1*06:03
HC0356	CONTIG1	A*02:01	B*07:02	C*05:01	DRB1*04:01	DQB1*03:01
	CONTIG2	A*03:01	B*44:02	C*07:02	DRB1*15:01	DQB1*06:03
HC0357	CONTIG1	A*02:01	B*35:01	C*04:01	DRB1*11:01	DQB1*03:01
	CONTIG2	A*24:02	B*44:03	-	DRB1*14:54	DQB1*05:03
HC0358	CONTIG1	A*02:01	B*18:01	C*12:03	DRB1*09:01	DQB1*03:03
	CONTIG2	A*25:01	B*51:01	C*14:02	DRB1*15:01	DQB1*06:02
HC0359	CONTIG1	A*01:01	B*08:01	C*07:01	DRB1*01:01	DQB1*02:01
	CONTIG2	A*24:02	B*51:01	C*15:02	DRB1*03:01	DQB1*05:01
HC0360	CONTIG1	A*11:01	B*18:01	C*12:03	DRB1*11:04	DQB1*03:01
	CONTIG2	A*25:01	B*51:01	-	DRB1*15:01	DQB1*06:02
HC0361	CONTIG1	A*01:01	B*08:01	C*03:03	DRB1*03:01	DQB1*02:01
	CONTIG2	A*26:01	B*55:01	C*07:01	DRB1*11:03	DQB1*03:01
HC0362	CONTIG1	A*01:01	B*13:02	C*02:02	DRB1*07:01	DQB1*02:02
	CONTIG2	A*02:01	B*40:02	C*06:02	DRB1*11:01	DQB1*03:01
HC0363	CONTIG1	A*02:01	B*07:02	C*04:01	DRB1*01:01	DQB1*05:01
	CONTIG2	A*23:01	B*35:01	C*07:02	DRB1*15:01	DQB1*06:02
HC0364	CONTIG1	A*02:01	B*07:02	C*02:02	DRB1*11:01	DQB1*03:01
	CONTIG2	A*03:01	B*51:01	C*07:02	DRB1*15:01	DQB1*06:02
HC0365	CONTIG1	A*02:01	B*18:01	C*07:01	DRB1*11:04	DQB1*03:01
	CONTIG2	A*24:02	-	C*07:01	DRB1*13:03	-
HC0366	CONTIG1	A*03:01	B*07:02	C*07:02	DRB1*01:01	DQB1*02:02
	CONTIG2	A*26:01	B*15:220	C*12:03	DRB1*07:01	DQB1*05:01
HC0367	CONTIG1	A*11:01	B*35:01	C*04:01	DRB1*01:01	DQB1*05:01
	CONTIG2	-	-	-	-	-
HC0368	CONTIG1	A*03:01	B*07:02	C*07:02	DRB1*07:01	DQB1*03:01
	CONTIG2	-	-	-	DRB1*11:01	DQB1*03:03
HC0369	CONTIG1	A*02:01	B*15:01	C*02:02	DRB1*11:03	DQB1*03:01
	CONTIG2	A*11:01	B*40:02	C*04:01	DRB1*13:02	DQB1*06:04
HC0370	CONTIG1	A*01:01	B*08:01	C*03:04	DRB1*03:01	DQB1*02:01
	CONTIG2	A*02:01	B*40:01	C*07:01	DRB1*13:02	DQB1*06:04
HC0371	CONTIG1	A*01:01	B*08:01	C*05:01	DRB1*03:01	DQB1*02:01
	CONTIG2	A*02:01	B*44:02	C*07:01	DRB1*04:01	DQB1*03:01
HC0372	CONTIG1	A*02:06	B*27:05	C*03:03	DRB1*04:01	DQB1*03:02
	CONTIG2	A*11:01	B*35:01	C*04:01	DRB1*14:54	DQB1*05:03
HC0373	CONTIG1	A*24:02	B*07:02	C*07:02	DRB1*15:01	DQB1*05:02
	CONTIG2	A*26:01	B*38:01	C*12:03	DRB1*16:01	DQB1*06:02
HC0374	CONTIG1	A*01:01	B*08:01	C*07:01	DRB1*07:01	DQB1*03:01
	CONTIG2	A*24:02	B*18:01	-	DRB1*11:04	DQB1*03:03
HC0375	CONTIG1	A*32:01	B*35:01	C*05:01	DRB1*10:01	DQB1*03:01

	CONTIG2	A*68:01	B*44:02	C*15:02	DRB1*11:03	DQB1*05:01
HC0376	CONTIG1	A*02:01	B*35:03	C*04:01	DRB1*04:03	DQB1*03:02
	CONTIG2	A*68:01	B*44:27	C*07:04	DRB1*16:01	DQB1*05:02
HC0377	CONTIG1	A*02:01	B*40:01	C*03:04	DRB1*11:01	DQB1*03:01
	CONTIG2	A*03:01	B*44:02	C*05:01	DRB1*13:01	DQB1*06:03
HC0378	CONTIG1	A*01:01	B*08:01	C*06:02	DRB1*07:01	DQB1*03:03
	CONTIG2	-	B*57:01	C*07:01	DRB1*15:01	DQB1*06:02
HC0379	CONTIG1	A*02:01	B*18:01	C*07:01	DRB1*11:01	DQB1*03:01
	CONTIG2	-	B*38:01	C*12:03	DRB1*11:03	-
HC0380	CONTIG1	A*11:01	B*18:01	C*02:02	DRB1*04:01	DQB1*02:02
	CONTIG2	A*25:01	B*40:02	C*12:03	DRB1*07:01	DQB1*03:02
HC0381	CONTIG1	A*23:01	B*18:01	C*04:01	DRB1*07:01	DQB1*02:02
	CONTIG2	A*24:02	B*44:03	C*07:01	DRB1*12:01	DQB1*03:01
HC0382	CONTIG1	A*01:01	B*15:01	C*03:03	DRB1*04:01	DQB1*03:02
	CONTIG2	A*25:01	B*55:01	-	-	-
HC0383	CONTIG1	A*02:01	B*13:02	C*04:01	DRB1*04:02	DQB1*02:02
	CONTIG2	A*30:01	B*44:03	C*06:02	DRB1*07:01	DQB1*03:02
HC0384	CONTIG1	A*01:01	B*44:03	C*03:03	DRB1*07:01	DQB1*02:02
	CONTIG2	A*02:01	B*55:01	C*04:01	DRB1*16:01	DQB1*05:02
HC0385	CONTIG1	A*02:01	B*18:01	C*02:02	DRB1*10:01	DQB1*05:01
	CONTIG2	A*25:01	B*27:05	C*12:03	DRB1*14:54	DQB1*05:03
HC0386	CONTIG1	A*01:01	B*08:01	C*01:02	DRB1*03:01	DQB1*02:01
	CONTIG2	A*02:01	B*56:01	C*07:01	DRB1*07:01	DQB1*02:02
HC0387	CONTIG1	A*02:01	B*15:01	C*02:02	DRB1*04:01	DQB1*03:01
	CONTIG2	A*32:01	B*40:02	C*03:03	DRB1*11:01	DQB1*03:02
HC0388	CONTIG1	A*30:01	B*13:02	C*06:02	DRB1*01:01	DQB1*03:01
	CONTIG2	A*68:01	B*44:02	C*07:04	DRB1*11:01	DQB1*05:01
HC0389	CONTIG1	A*03:01	B*07:02	C*07:02	DRB1*03:01	DQB1*02:01
	CONTIG2	A*25:01	B*18:01	C*12:03	DRB1*04:07	DQB1*03:01
HC0390	CONTIG1	A*03:01	B*35:01	C*04:01	DRB1*11:01	DQB1*03:01
	CONTIG2	A*11:01	B*35:03	-	DRB1*14:54	DQB1*05:03
HC0391	CONTIG1	A*02:01	B*15:01	C*03:03	DRB1*04:04	DQB1*03:02
	CONTIG2	A*26:01	B*44:02	C*03:04	DRB1*13:01	DQB1*06:03
HC0392	CONTIG1	A*24:02	B*27:05	C*01:02	DRB1*01:01	DQB1*03:01
	CONTIG2	A*32:01	B*44:03	C*04:01	DRB1*11:01	DQB1*05:01
HC0393	CONTIG1	A*25:01	B*38:01	C*03:03	DRB1*11:03	DQB1*03:01
	CONTIG2	A*30:01	B*55:01	C*12:03	DRB1*13:01	DQB1*06:03
HC0394	CONTIG1	A*02:01	B*14:01	C*07:02	DRB1*01:01	DQB1*02:02
	CONTIG2	A*24:02	B*39:06	C*08:02	DRB1*07:01	DQB1*05:01
HC0395	CONTIG1	A*25:01	B*18:01	C*07:01	DRB1*13:01	DQB1*06:03
	CONTIG2	A*26:01	B*49:01	C*12:03	DRB1*13:02	DQB1*06:04
HC0396	CONTIG1	A*24:02	B*07:02	C*07:01	DRB1*01:01	DQB1*05:04
	CONTIG2	A*26:01	B*49:01	C*07:02	DRB1*15:01	DQB1*06:02
HC0397	CONTIG1	A*03:01	B*07:02	C*07:02	DRB1*15:01	DQB1*06:02
	CONTIG2	A*25:01	B*18:01	C*12:03	-	-
HC0398	CONTIG1	A*01:01	B*13:02	C*06:02	DRB1*07:01	DQB1*02:02
	CONTIG2	A*02:01	B*44:03	C*16:02	DRB1*07:01	-
HC0399	CONTIG1	A*01:01	B*27:05	C*02:02	DRB1*13:02	DQB1*05:02
	CONTIG2	A*24:02	B*58:01	C*03:02	DRB1*16:01	DQB1*06:09
HC0400	CONTIG1	A*11:01	B*18:01	C*07:01	DRB1*04:04	DQB1*03:02

	CONTIG2	A*24:02	B*51:01	C*16:02	DRB1*07:01	DQB1*03:03
HC0401	CONTIG1	A*03:01	B*07:02	C*05:01	DRB1*07:01	DQB1*02:02
	CONTIG2	-	B*44:02	C*07:02	DRB1*15:01	DQB1*06:02
HC0402	CONTIG1	A*03:01	B*15:01	C*04:01	DRB1*01:01	DQB1*05:01
	CONTIG2	A*29:01	B*35:01	C*04:01	DRB1*15:01	DQB1*06:02
HC0403	CONTIG1	A*02:01	B*07:02	C*05:01	DRB1*15:01	DQB1*06:02
	CONTIG2	A*24:02	B*44:02	C*07:02	-	-
HC0404	CONTIG1	A*01:01	B*08:01	C*02:02	DRB1*01:01	DQB1*02:01
	CONTIG2	A*11:01	B*27:05	C*07:01	DRB1*03:01	DQB1*05:01
HC0405	CONTIG1	A*02:01	B*07:02	C*07:01	DRB1*13:01	DQB1*03:01
	CONTIG2	A*26:01	B*18:01	C*07:02	DRB1*13:15	DQB1*06:03
HC0406	CONTIG1	A*02:01	B*35:03	C*04:01	DRB1*11:01	DQB1*03:01
	CONTIG2	A*24:02	B*57:03	C*07:01	DRB1*15:01	DQB1*06:02
HC0407	CONTIG1	A*03:01	B*07:02	C*07:01	DRB1*03:01	DQB1*02:01
	CONTIG2	A*11:01	B*08:01	C*07:02	DRB1*15:01	DQB1*06:02
HC0408	CONTIG1	A*24:02	B*18:01	C*06:02	DRB1*07:01	DQB1*03:03
	CONTIG2	-	B*57:01	C*07:01	DRB1*13:02	DQB1*06:04
HC0409	CONTIG1	A*01:01	B*14:02	C*08:02	DRB1*01:02	DQB1*04:02
	CONTIG2	A*24:02	B*52:01	C*12:02	DRB1*08:01	DQB1*05:01
HC0410	CONTIG1	A*25:01	B*18:01	C*12:03	DRB1*15:01	DQB1*06:02
	CONTIG2	A*26:01	B*38:01	-	-	DQB1*06:03
HC0411	CONTIG1	A*03:01	B*07:02	C*04:01	DRB1*01:01	DQB1*05:01
	CONTIG2	A*11:01	B*35:01	C*07:02	-	-
HC0412	CONTIG1	A*01:01	B*08:01	C*01:02	DRB1*01:01	DQB1*02:01
	CONTIG2	A*03:01	B*51:01	C*07:01	DRB1*03:01	DQB1*05:01
HC0413	CONTIG1	A*03:01	B*35:01	C*04:01	DRB1*08:01	DQB1*03:02
	CONTIG2	A*68:01	B*35:03	C*04:01	DRB1*15:01	DQB1*06:02
HC0414	CONTIG1	A*02:01	B*15:220	C*05:01	DRB1*07:01	DQB1*02:02
	CONTIG2	A*25:01	B*44:27	C*07:04	DRB1*16:01	DQB1*05:02
HC0415	CONTIG1	A*01:01	B*08:01	C*07:01	DRB1*03:01	DQB1*02:01
	CONTIG2	A*24:02	B*38:01	C*12:03	DRB1*13:01	DQB1*06:03
HC0416	CONTIG1	A*02:01	B*27:02	C*02:02	DRB1*01:01	DQB1*05:01
	CONTIG2	A*68:01	B*40:01	C*03:04	DRB1*16:01	DQB1*05:02
HC0417	CONTIG1	A*02:01	B*27:05	C*01:02	DRB1*01:01	DQB1*05:01
	CONTIG2	A*24:02	B*44:02	C*05:01	DRB1*16:01	DQB1*05:02
HC0418	CONTIG1	A*01:01	B*07:02	C*06:02	DRB1*01:01	DQB1*02:02
	CONTIG2	A*03:01	B*13:02	C*07:02	DRB1*07:01	DQB1*05:01
HC0419	CONTIG1	A*01:01	B*08:01	C*02:02	DRB1*03:01	DQB1*02:01
	CONTIG2	-	B*27:02	C*07:01	DRB1*11:01	DQB1*03:01
HC0420	CONTIG1	A*02:01	B*13:02	C*04:01	DRB1*01:01	DQB1*02:02
	CONTIG2	A*24:02	B*15:01	C*06:02	DRB1*07:01	DQB1*05:01
HC0421	CONTIG1	A*01:01	B*07:02	C*04:01	DRB1*14:01	DQB1*05:03
	CONTIG2	A*30:01	B*35:01	C*07:02	DRB1*15:01	DQB1*06:02
HC0422	CONTIG1	A*02:01	B*18:01	C*01:02	DRB1*11:04	DQB1*03:01
	CONTIG2	A*30:01	B*56:01	C*07:01	-	-
HC0423	CONTIG1	A*03:01	B*35:01	C*01:02	DRB1*01:01	DQB1*05:01
	CONTIG2	A*11:01	B*51:01	C*04:01	DRB1*14:54	DQB1*05:03
HC0424	CONTIG1	A*02:01	B*07:02	C*02:02	DRB1*11:04	DQB1*03:01
	CONTIG2	A*24:02	B*27:05	C*07:02	DRB1*12:01	-
HC0425	CONTIG1	A*02:01	B*08:01	C*02:02	DRB1*01:01	DQB1*03:01

	CONTIG2	A*24:02	B*27:02	C*07:01	DRB1*11:04	DQB1*05:02
HC0426	CONTIG1	A*02:01	B*13:02	C*06:02	DRB1*07:01	DQB1*02:02
	CONTIG2	A*25:01	B*18:01	C*12:03	DRB1*16:01	DQB1*05:02
HC0427	CONTIG1	A*01:01	B*08:01	C*07:01	DRB1*03:01	DQB1*02:01
	CONTIG2	A*32:01	B*41:02	C*17:03	DRB1*13:03	DQB1*03:01
HC0428	CONTIG1	A*02:01	B*18:01	C*05:01	DRB1*11:04	DQB1*03:01
	CONTIG2	A*03:01	B*44:02	C*06:02	DRB1*12:01	DQB1*03:01
HC0429	CONTIG1	A*01:01	B*08:01	C*03:04	DRB1*03:01	DQB1*02:01
	CONTIG2	A*31:01	B*40:01	C*07:01	DRB1*15:01	DQB1*06:02
HC0430	CONTIG1	A*01:01	B*15:01	C*03:03	DRB1*08:01	DQB1*04:02
	CONTIG2	A*25:01	B*39:06	C*07:02	DRB1*15:01	DQB1*06:02
HC0431	CONTIG1	A*02:01	B*07:02	C*07:02	DRB1*07:01	DQB1*02:02
	CONTIG2	-	B*51:01	C*15:02	DRB1*15:01	DQB1*06:02
HC0432	CONTIG1	A*01:01	B*08:01	C*07:01	DRB1*03:01	DQB1*02:01
	CONTIG2	A*02:01	B*18:01	-	DRB1*11:04	DQB1*03:01
HC0433	CONTIG1	A*02:01	B*13:02	C*06:02	DRB1*03:01	DQB1*02:01
	CONTIG2	A*24:02	B*51:01	C*15:02	DRB1*09:01	DQB1*03:03
HC0434	CONTIG1	A*01:01	B*08:01	C*06:02	DRB1*03:01	DQB1*02:01
	CONTIG2	A*02:01	B*13:02	C*07:01	DRB1*07:01	DQB1*02:02
HC0435	CONTIG1	A*26:01	B*27:05	C*01:02	DRB1*07:01	DQB1*02:02
	CONTIG2	A*32:01	B*44:03	C*04:01	-	DQB1*03:03
HC0436	CONTIG1	A*11:01	B*35:01	C*04:01	DRB1*07:01	DQB1*02:02
	CONTIG2	A*25:01	B*52:01	C*12:02	DRB1*16:01	DQB1*05:02
HC0437	CONTIG1	A*23:01	B*15:01	C*03:04	DRB1*07:01	DQB1*02:02
	CONTIG2	A*33:03	B*44:03	C*04:01	DRB1*13:01	DQB1*06:03
HC0438	CONTIG1	A*01:01	B*08:01	C*06:02	DRB1*03:01	DQB1*02:01
	CONTIG2	-	B*37:01	C*07:01	DRB1*07:01	DQB1*02:02
HC0439	CONTIG1	A*02:01	B*15:01	C*03:03	DRB1*01:01	DQB1*03:02
	CONTIG2	-	-	C*04:01	DRB1*04:01	DQB1*05:01
HC0440	CONTIG1	A*01:01	B*08:01	C*03:03	DRB1*03:01	DQB1*02:01
	CONTIG2	A*68:24	B*55:01	C*07:01	DRB1*13:01	DQB1*06:03
HC0441	CONTIG1	A*02:01	B*13:02	C*02:02	DRB1*07:01	DQB1*02:02
	CONTIG2	A*03:01	B*51:01	C*06:02	DRB1*09:01	DQB1*03:03
HC0442	CONTIG1	A*01:01	B*08:01	C*04:01	DRB1*01:01	DQB1*02:01
	CONTIG2	A*11:01	B*35:01	C*07:01	DRB1*03:01	DQB1*05:01
HC0443	CONTIG1	A*02:01	B*15:01	C*03:03	DRB1*10:01	DQB1*05:01
	CONTIG2	A*24:02	B*44:27	C*05:01	DRB1*16:01	DQB1*05:02
HC0444	CONTIG1	A*01:01	B*08:01	C*06:02	DRB1*03:01	DQB1*02:01
	CONTIG2	A*26:01	B*57:01	C*07:01	DRB1*07:01	DQB1*03:03
HC0445	CONTIG1	A*02:05	B*35:01	C*04:01	DRB1*01:01	DQB1*02:02
	CONTIG2	A*03:01	B*49:01	C*07:01	DRB1*07:01	DQB1*05:01
HC0446	CONTIG1	A*03:01	B*35:02	C*01:02	DRB1*11:01	DQB1*03:01
	CONTIG2	A*24:03	B*51:01	C*04:01	DRB1*11:04	-
HC0447	CONTIG1	A*01:01	B*08:01	C*03:03	DRB1*13:02	DQB1*06:02
	CONTIG2	A*24:02	B*55:01	C*07:01	DRB1*15:01	DQB1*06:04
HC0448	CONTIG1	A*31:01	B*27:05	C*02:02	DRB1*09:01	DQB1*03:01
	CONTIG2	A*32:01	B*39:01	C*12:03	DRB1*12:01	DQB1*03:03
HC0449	CONTIG1	A*24:02	B*38:01	C*12:03	DRB1*09:01	DQB1*03:03
	CONTIG2	A*32:01	B*51:01	C*15:02	DRB1*13:01	DQB1*06:03
HC0450	CONTIG1	A*02:01	B*13:02	C*02:02	DRB1*07:01	DQB1*02:02

	CONTIG2	A*24:02	B*27:02	C*06:02	DRB1*16:01	DQB1*05:02
HC0451	CONTIG1	A*03:01	B*35:01	C*04:01	DRB1*01:01	DQB1*03:01
	CONTIG2	A*31:01	B*44:02	C*05:01	DRB1*11:04	DQB1*05:01
HC0452	CONTIG1	A*01:01	B*08:01	C*07:01	DRB1*03:01	DQB1*02:01
	CONTIG2	A*24:02	B*42:01	C*17:01	DRB1*03:02	DQB1*04:02
HC0453	CONTIG1	A*03:01	B*18:01	C*12:03	DRB1*07:01	DQB1*02:02
	CONTIG2	A*23:01	B*39:01	-	DRB1*13:01	DQB1*06:03
HC0454	CONTIG1	A*23:01	B*14:01	C*04:01	DRB1*07:01	DQB1*02:02
	CONTIG2	A*24:02	B*35:01	C*08:02	DRB1*13:03	DQB1*03:01
HC0455	CONTIG1	A*24:02	B*27:05	C*02:02	DRB1*04:01	DQB1*03:02
	CONTIG2	A*32:01	B*53:01	C*04:01	DRB1*13:02	DQB1*06:04
HC0456	CONTIG1	A*25:01	B*15:01	C*03:03	DRB1*07:01	DQB1*03:03
	CONTIG2	A*68:01	B*41:02	C*17:03	DRB1*15:01	DQB1*06:02
HC0457	CONTIG1	A*25:01	B*14:01	C*08:02	DRB1*04:04	DQB1*02:02
	CONTIG2	A*26:01	B*18:01	C*12:03	DRB1*07:01	-
HC0458	CONTIG1	A*03:01	B*13:02	C*04:01	DRB1*01:01	DQB1*02:02
	CONTIG2	A*25:01	B*35:01	C*06:02	DRB1*07:01	DQB1*05:01
HC0459	CONTIG1	A*02:01	B*15:01	C*03:04	DRB1*03:01	DQB1*02:01
	CONTIG2	A*24:02	B*48:01	C*08:03	DRB1*16:01	DQB1*05:02
HC0460	CONTIG1	A*02:01	B*07:02	C*06:02	DRB1*07:01	DQB1*02:02
	CONTIG2	A*03:01	B*13:02	C*07:02	DRB1*15:01	DQB1*06:02
HC0461	CONTIG1	A*02:01	B*07:02	C*07:01	DRB1*11:01	DQB1*03:01
	CONTIG2	A*03:01	B*18:01	C*07:02	DRB1*15:01	DQB1*06:02
HC0462	CONTIG1	A*01:01	B*44:27	C*07:04	DRB1*11:04	DQB1*03:01
	CONTIG2	A*31:01	B*51:01	C*12:03	DRB1*16:01	DQB1*05:02
HC0463	CONTIG1	A*03:01	B*37:01	C*06:02	DRB1*07:01	DQB1*02:02
	CONTIG2	A*25:01	B*47:01	C*12:03	DRB1*10:01	DQB1*05:01
HC0464	CONTIG1	A*23:01	B*08:01	C*03:03	DRB1*03:01	DQB1*02:01
	CONTIG2	A*26:08	B*55:01	C*07:01	DRB1*15:01	DQB1*05:02
HC0465	CONTIG1	A*01:01	B*08:01	C*07:01	DRB1*03:01	DQB1*02:01
	CONTIG2	A*30:01	B*38:01	C*12:03	DRB1*13:01	DQB1*06:03
HC0466	CONTIG1	A*03:01	B*13:02	C*01:02	DRB1*07:01	DQB1*02:02
	CONTIG2	A*68:01	B*27:05	C*06:02	-	-
HC0467	CONTIG1	A*01:01	B*07:02	C*07:01	DRB1*01:01	DQB1*02:01
	CONTIG2	A*03:01	B*08:01	C*07:02	DRB1*03:01	DQB1*05:01
HC0468	CONTIG1	A*01:01	B*08:01	C*07:01	DRB1*01:01	DQB1*02:01
	CONTIG2	A*02:01	B*52:01	C*12:02	DRB1*03:01	DQB1*05:01
HC0469	CONTIG1	A*02:01	B*18:01	C*12:03	DRB1*07:01	-
	CONTIG2	A*25:01	B*39:01	-	DRB1*11:01	-
HC0470	CONTIG1	A*01:01	B*08:01	C*04:01	DRB1*03:01	DQB1*02:01
	CONTIG2	A*03:01	B*35:01	C*07:01	DRB1*07:01	DQB1*02:02
HC0471	CONTIG1	A*02:01	B*08:01	C*02:02	DRB1*03:01	DQB1*02:01
	CONTIG2	-	B*27:05	C*07:01	DRB1*07:01	DQB1*03:03
HC0472	CONTIG1	A*02:01	B*07:02	C*07:02	DRB1*07:01	DQB1*03:03
	CONTIG2	A*03:01	-	-	DRB1*15:01	DQB1*06:02
HC0473	CONTIG1	A*01:01	B*08:01	C*07:01	DRB1*03:01	DQB1*02:01
	CONTIG2	A*29:02	B*44:03	C*16:01	DRB1*07:01	DQB1*02:02
HC0474	CONTIG1	A*11:01	B*07:02	C*07:02	DRB1*01:01	DQB1*03:01
	CONTIG2	A*24:02	B*52:01	C*12:02	DRB1*11:01	DQB1*05:01
HC0475	CONTIG1	A*11:01	B*35:01	C*04:01	DRB1*01:01	DQB1*03:02

	CONTIG2	-	B*51:01	C*15:02	DRB1*04:04	DQB1*05:01
HC0476	CONTIG1	A*02:01	B*27:05	C*02:02	DRB1*01:01	DQB1*04:02
	CONTIG2	A*24:02	B*42:01	C*17:01	DRB1*03:02	DQB1*05:01
HC0477	CONTIG1	A*24:02	B*27:02	C*05:01	DRB1*11:01	DQB1*03:01
	CONTIG2	A*32:01	B*44:02	-	DRB1*15:01	DQB1*06:02
HC0478	CONTIG1	A*02:01	B*14:02	C*08:02	DRB1*03:01	DQB1*02:01
	CONTIG2	A*26:01	B*41:01	C*17:01	DRB1*13:02	DQB1*06:09
HC0479	CONTIG1	A*24:02	B*13:02	C*02:02	DRB1*01:01	DQB1*02:02
	CONTIG2	A*25:01	B*27:05	C*06:02	DRB1*07:01	DQB1*05:01
HC0480	CONTIG1	A*11:01	B*44:05	C*02:02	DRB1*11:01	DQB1*03:01
	CONTIG2	A*24:02	B*52:01	C*12:02	DRB1*15:02	DQB1*06:01
HC0481	CONTIG1	A*03:01	B*07:02	C*07:01	DRB1*07:01	DQB1*03:03
	CONTIG2	-	B*08:01	C*07:02	DRB1*16:01	DQB1*05:02
HC0482	CONTIG1	A*02:01	B*13:02	C*03:04	DRB1*07:01	DQB1*02:02
	CONTIG2	A*24:02	B*40:01	C*06:02	DRB1*15:01	DQB1*06:02
HC0483	CONTIG1	A*01:01	B*14:02	C*06:02	DRB1*01:01	DQB1*02:01
	CONTIG2	A*33:01	B*57:01	C*08:02	DRB1*03:01	DQB1*05:01
HC0484	CONTIG1	A*02:01	B*15:01	C*03:04	DRB1*04:01	DQB1*03:02
	CONTIG2	A*03:01	B*44:02	C*05:01	DRB1*13:01	DQB1*06:03
HC0485	CONTIG1	A*02:01	B*18:01	C*01:02	DRB1*07:01	DQB1*02:02
	CONTIG2	A*26:01	B*56:01	C*12:03	DRB1*16:01	DQB1*05:02
HC0486	CONTIG1	A*02:01	B*18:01	C*02:02	DRB1*03:01	DQB1*02:01
	CONTIG2	A*30:02	B*27:05	C*05:01	DRB1*08:01	DQB1*04:02
HC0487	CONTIG1	A*02:01	B*15:01	C*03:04	DRB1*04:01	DQB1*03:02
	CONTIG2	A*31:01	B*35:01	C*04:01	DRB1*14:54	DQB1*05:03
HC0488	CONTIG1	A*02:01	B*07:02	C*03:03	DRB1*15:01	DQB1*05:02
	CONTIG2	A*03:01	B*15:01	C*07:02	DRB1*16:01	DQB1*06:02
HC0489	CONTIG1	A*02:01	B*27:05	C*01:02	DRB1*01:01	DQB1*02:01
	CONTIG2	A*25:01	B*35:01	C*03:03	DRB1*03:01	DQB1*05:01
HC0490	CONTIG1	A*03:02	B*15:01	C*03:04	DRB1*04:01	DQB1*03:02
	CONTIG2	A*24:02	B*57:01	C*06:02	DRB1*07:01	DQB1*03:03
HC0491	CONTIG1	A*11:01	B*13:02	C*04:01	DRB1*01:01	DQB1*03:02
	CONTIG2	A*30:01	B*35:01	C*06:02	DRB1*04:04	DQB1*05:01
HC0492	CONTIG1	A*24:02	B*13:02	C*05:01	DRB1*01:01	DQB1*02:02
	CONTIG2	A*31:01	B*27:05	C*06:02	DRB1*07:01	DQB1*05:01
HC0493	CONTIG1	A*03:01	B*07:02	C*07:02	DRB1*04:01	DQB1*03:02
	CONTIG2	A*25:01	B*18:01	C*12:03	DRB1*15:01	DQB1*06:02
HC0494	CONTIG1	A*02:01	B*07:02	C*07:02	DRB1*01:01	DQB1*05:01
	CONTIG2	A*31:01	B*39:01	C*12:03	DRB1*13:01	DQB1*06:03
HC0495	CONTIG1	A*02:01	B*07:02	C*02:02	DRB1*15:01	DQB1*05:02
	CONTIG2	A*24:02	B*40:02	C*07:02	DRB1*16:02	DQB1*06:02
HC0496	CONTIG1	A*01:01	B*07:02	C*07:01	DRB1*03:01	DQB1*02:01
	CONTIG2	A*03:01	B*08:01	C*07:02	DRB1*15:01	DQB1*06:02
HC0497	CONTIG1	A*02:01	B*07:02	C*15:02	DRB1*07:01	DQB1*02:02
	CONTIG2	-	B*41:01	C*17:01	DRB1*15:01	DQB1*06:02
HC0498	CONTIG1	A*26:01	B*13:02	C*06:02	DRB1*04:01	DQB1*02:02
	CONTIG2	A*30:01	B*38:01	C*12:03	DRB1*07:01	DQB1*03:01
HC0499	CONTIG1	A*02:01	B*14:02	C*04:01	DRB1*01:02	DQB1*05:01
	CONTIG2	A*11:01	B*35:01	C*08:02	DRB1*16:01	DQB1*05:02
HC0500	CONTIG1	A*01:01	B*15:01	C*03:03	DRB1*11:03	DQB1*03:01

	CONTIG2	A*24:02	B*41:02	C*17:03	DRB1*13:03	-
HC0501	CONTIG1	A*02:05	B*49:01	C*06:02	DRB1*03:01	DQB1*02:01
	CONTIG2	A*03:01	B*50:01	C*07:01	DRB1*07:01	DQB1*03:03
HC0502	CONTIG1	A*68:01	B*13:02	C*06:02	DRB1*07:01	DQB1*02:02
	CONTIG2	-	B*18:01	C*07:01	DRB1*16:01	DQB1*05:02
HC0503	CONTIG1	A*02:01	B*15:01	C*03:03	DRB1*01:01	DQB1*05:01
	CONTIG2	A*25:01	B*18:01	C*12:03	DRB1*15:01	DQB1*06:02
HC0504	CONTIG1	A*01:01	B*57:01	C*06:02	DRB1*07:01	DQB1*03:01
	CONTIG2	A*02:01	B*58:01	C*16:02	DRB1*11:01	DQB1*03:03
HC0505	CONTIG1	A*02:01	B*38:01	C*03:04	DRB1*04:01	DQB1*03:01
	CONTIG2	A*03:01	B*40:01	C*12:03	DRB1*13:01	DQB1*06:03
HC0506	CONTIG1	A*03:01	B*07:02	C*04:01	DRB1*01:01	DQB1*05:01
	CONTIG2	A*68:01	B*35:03	C*07:02	DRB1*15:01	DQB1*06:02
HC0507	CONTIG1	A*02:01	B*15:01	C*04:01	DRB1*08:01	DQB1*04:02
	CONTIG2	A*31:01	B*18:01	C*07:01	-	-
HC0508	CONTIG1	A*02:01	B*07:02	C*07:02	DRB1*15:01	DQB1*05:02
	CONTIG2	A*26:01	-	-	DRB1*16:01	DQB1*06:02
HC0509	CONTIG1	A*02:01	B*07:02	C*02:02	DRB1*15:01	DQB1*05:02
	CONTIG2	A*03:01	B*27:02	C*07:02	DRB1*16:01	DQB1*06:02
HC0510	CONTIG1	A*03:01	B*15:01	C*03:03	DRB1*04:01	DQB1*03:02
	CONTIG2	A*11:01	B*55:01	C*03:04	DRB1*14:54	DQB1*05:03
HC0511	CONTIG1	A*02:01	B*15:01	C*04:01	DRB1*01:01	DQB1*04:02
	CONTIG2	A*11:01	B*52:01	C*12:02	DRB1*08:01	DQB1*05:01
HC0512	CONTIG1	A*02:01	B*18:03	C*05:01	DRB1*04:01	DQB1*03:01
	CONTIG2	A*32:01	B*44:02	C*07:01	DRB1*11:01	DQB1*03:01
HC0513	CONTIG1	A*01:01	B*07:02	C*07:01	DRB1*03:01	DQB1*02:01
	CONTIG2	A*68:01	B*08:01	C*07:02	DRB1*12:01	DQB1*03:01
HC0514	CONTIG1	A*02:01	B*13:02	C*01:02	DRB1*01:01	DQB1*02:02
	CONTIG2	A*11:01	B*56:01	C*06:02	DRB1*07:01	DQB1*05:01
HC0515	CONTIG1	A*02:01	B*14:01	C*08:02	DRB1*07:01	DQB1*02:02
	CONTIG2	A*11:01	B*52:01	C*12:02	DRB1*15:02	DQB1*06:01
HC0516	CONTIG1	A*01:01	B*08:01	C*04:01	DRB1*03:01	DQB1*02:01
	CONTIG2	A*23:01	B*44:03	C*07:01	DRB1*07:01	DQB1*02:02
HC0517	CONTIG1	A*01:01	B*15:18	C*07:04	DRB1*11:01	DQB1*03:01
	CONTIG2	A*32:01	B*18:01	-	DRB1*13:01	DQB1*06:03
HC0518	CONTIG1	A*01:01	B*07:02	C*06:02	DRB1*07:01	DQB1*03:03
	CONTIG2	A*03:01	B*57:01	C*07:02	DRB1*15:01	DQB1*06:02
HC0519	CONTIG1	A*02:01	B*35:01	C*02:02	DRB1*01:01	DQB1*05:01
	CONTIG2	A*11:01	B*44:05	C*04:01	DRB1*16:01	DQB1*05:02
HC0520	CONTIG1	A*03:01	B*40:01	C*03:04	DRB1*12:01	DQB1*03:01
	CONTIG2	A*31:01	B*51:01	C*15:02	DRB1*15:01	DQB1*06:02
HC0521	CONTIG1	A*23:01	B*44:03	C*03:02	DRB1*07:01	DQB1*02:02
	CONTIG2	A*33:03	B*58:01	C*04:01	DRB1*15:01	DQB1*06:02
HC0522	CONTIG1	A*03:01	B*07:02	C*07:02	DRB1*15:01	DQB1*06:02
	CONTIG2	-	-	-	-	-
HC0523	CONTIG1	A*11:01	B*08:01	C*04:01	DRB1*01:01	DQB1*02:01
	CONTIG2	A*25:01	B*35:01	C*07:01	DRB1*03:01	DQB1*05:01
HC0524	CONTIG1	A*25:01	B*18:01	C*12:03	DRB1*01:01	DQB1*05:01
	CONTIG2	-	B*39:01	-	DRB1*15:01	DQB1*06:02
HC0525	CONTIG1	A*01:01	B*08:01	C*04:01	DRB1*03:01	DQB1*02:01

	CONTIG2	A*02:01	B*35:03	C*07:01	DRB1*10:38Q	DQB1*05:01
HC0526	CONTIG1	A*01:01	B*08:01	C*01:02	DRB1*01:01	DQB1*02:01
	CONTIG2	A*02:01	B*27:05	C*07:01	DRB1*03:01	DQB1*05:01
HC0527	CONTIG1	A*02:01	B*13:02	C*02:02	DRB1*07:01	DQB1*02:02
	CONTIG2	A*26:01	B*27:05	C*06:02	DRB1*08:01	DQB1*04:02
HC0528	CONTIG1	A*02:01	B*27:05	C*02:02	DRB1*08:01	DQB1*04:02
	CONTIG2	-	B*37:01	C*03:03	DRB1*15:01	DQB1*05:02
HC0529	CONTIG1	A*11:01	B*18:01	C*06:02	DRB1*11:01	DQB1*03:01
	CONTIG2	A*24:02	B*44:02	C*07:01	DRB1*14:01	DQB1*05:03
HC0530	CONTIG1	A*03:01	B*44:02	C*06:02	DRB1*07:01	DQB1*03:01
	CONTIG2	A*68:01	B*57:01	C*07:04	DRB1*11:01	DQB1*03:03
HC0531	CONTIG1	A*02:01	B*39:01	C*02:02	DRB1*13:01	DQB1*05:02
	CONTIG2	-	B*45:01	C*16:01	DRB1*16:01	DQB1*06:03
HC0532	CONTIG1	A*02:01	B*15:01	C*04:01	DRB1*01:01	DQB1*05:01
	CONTIG2	-	B*44:27	C*07:04	DRB1*16:01	DQB1*05:02
HC0533	CONTIG1	A*01:01	B*07:02	C*07:01	DRB1*03:01	DQB1*02:01
	CONTIG2	A*03:01	B*08:01	C*07:02	DRB1*15:01	DQB1*06:02
HC0534	CONTIG1	A*33:03	B*41:02	C*03:02	DRB1*04:06	DQB1*04:02
	CONTIG2	A*66:01	B*58:01	C*17:03	DRB1*10:01	DQB1*05:01
HC0535	CONTIG1	A*02:01	B*44:05	C*02:02	DRB1*08:04	DQB1*04:02
	CONTIG2	A*24:02	-	-	DRB1*16:01	DQB1*05:02
HC0536	CONTIG1	A*01:01	B*08:01	C*07:01	DRB1*03:01	DQB1*02:01
	CONTIG2	-	-	-	-	-
HC0537	CONTIG1	A*02:01	B*35:01	C*03:04	DRB1*01:01	DQB1*05:01
	CONTIG2	A*03:01	B*40:01	C*04:01	DRB1*13:02	DQB1*06:04
HC0538	CONTIG1	A*02:01	B*39:01	C*12:03	DRB1*07:01	DQB1*02:02
	CONTIG2	A*29:02	B*44:03	C*16:01	DRB1*16:01	DQB1*05:02
HC0539	CONTIG1	A*02:01	B*15:01	C*04:01	DRB1*07:01	DQB1*03:03
	CONTIG2	-	B*44:02	C*07:04	DRB1*15:01	DQB1*06:02
HC0540	CONTIG1	A*02:01	B*13:02	C*06:02	DRB1*07:01	DQB1*02:02
	CONTIG2	A*30:01	-	-	-	-
HC0541	CONTIG1	A*02:01	B*14:02	C*03:03	DRB1*01:02	DQB1*03:01
	CONTIG2	A*03:01	B*15:01	C*08:02	DRB1*13:03	DQB1*05:01
HC0542	CONTIG1	A*02:01	B*07:02	C*07:02	DRB1*01:01	DQB1*03:02
	CONTIG2	A*25:01	B*15:01	C*12:03	DRB1*04:01	DQB1*05:01
HC0543	CONTIG1	A*03:01	B*18:01	C*06:02	DRB1*11:04	DQB1*03:01
	CONTIG2	A*26:01	B*38:01	C*12:03	DRB1*13:01	DQB1*06:03
HC0544	CONTIG1	A*03:02	B*44:02	C*05:01	DRB1*01:01	DQB1*02:02
	CONTIG2	A*68:01	B*44:03	C*16:01	DRB1*07:01	DQB1*05:01
HC0545	CONTIG1	A*23:01	B*35:01	C*03:04	DRB1*01:01	DQB1*02:02
	CONTIG2	A*30:02	B*44:03	C*04:01	DRB1*07:01	DQB1*05:01
HC0546	CONTIG1	A*03:01	B*35:01	C*04:01	DRB1*12:01	DQB1*03:01
	CONTIG2	A*11:01	B*51:01	C*16:02	DRB1*15:01	DQB1*06:02
HC0547	CONTIG1	A*02:01	B*37:01	C*06:02	DRB1*03:01	DQB1*02:01
	CONTIG2	A*32:01	B*39:01	C*12:03	-	-
HC0548	CONTIG1	A*02:01	B*07:02	C*03:359	DRB1*07:01	DQB1*03:03
	CONTIG2	A*03:01	B*40:01	C*07:02	DRB1*15:01	DQB1*06:02
HC0549	CONTIG1	A*02:01	B*41:01	C*05:01	DRB1*03:01	DQB1*02:01
	CONTIG2	A*23:01	B*44:02	C*17:01	DRB1*07:01	DQB1*02:02
HC0550	CONTIG1	A*02:01	B*07:02	C*03:04	DRB1*04:01	DQB1*03:02

	CONTIG2	A*03:01	B*15:01	C*07:02	DRB1*15:01	DQB1*06:02
HC0551	CONTIG1	A*02:01	B*44:03	C*03:03	DRB1*07:01	DQB1*02:02
	CONTIG2	A*26:01	B*55:01	C*16:01	DRB1*11:03	DQB1*03:01
HC0552	CONTIG1	A*03:01	B*35:01	C*01:02	DRB1*14:54	DQB1*05:03
	CONTIG2	A*03:01	B*56:01	C*04:01	DRB1*15:01	DQB1*06:02
HC0553	CONTIG1	A*24:02	B*27:05	C*01:02	DRB1*01:01	DQB1*03:03
	CONTIG2	A*31:01	B*57:01	C*06:02	DRB1*07:01	DQB1*05:01
HC0554	CONTIG1	A*11:01	B*44:03	C*04:01	DRB1*07:01	DQB1*03:01
	CONTIG2	A*24:02	B*57:01	C*06:02	DRB1*11:01	DQB1*03:03
HC0555	CONTIG1	A*02:01	B*44:02	C*02:02	DRB1*11:01	DQB1*03:01
	CONTIG2	A*03:01	B*44:05	C*05:01	-	-
HC0556	CONTIG1	A*24:02	B*08:01	C*03:03	DRB1*03:01	DQB1*02:01
	CONTIG2	A*32:01	B*15:135	C*07:02	DRB1*13:01	DQB1*06:03
HC0557	CONTIG1	A*03:01	B*07:02	C*07:01	DRB1*11:04	DQB1*03:01
	CONTIG2	-	B*18:01	C*07:02	DRB1*13:01	DQB1*06:03
HC0558	CONTIG1	A*01:01	B*44:02	C*05:01	DRB1*01:01	DQB1*05:01
	CONTIG2	A*02:01	B*57:01	C*06:02	DRB1*13:01	DQB1*06:03
HC0559	CONTIG1	A*23:01	B*07:02	C*07:01	DRB1*11:01	DQB1*03:01
	CONTIG2	A*26:01	B*49:01	C*07:02	DRB1*15:01	DQB1*06:02
HC0560	CONTIG1	A*01:01	B*08:01	C*06:02	DRB1*03:01	DQB1*02:01
	CONTIG2	A*30:01	B*57:01	C*07:01	DRB1*07:01	DQB1*03:03
HC0561	CONTIG1	A*30:02	B*08:01	C*02:02	DRB1*03:01	DQB1*02:01
	CONTIG2	A*31:01	B*44:05	C*07:01	DRB1*16:01	DQB1*05:02
HC0562	CONTIG1	A*01:01	B*27:05	C*02:02	DRB1*01:01	DQB1*03:01
	CONTIG2	A*03:01	B*57:01	C*06:02	DRB1*12:01	DQB1*05:01
HC0563	CONTIG1	A*02:01	B*38:01	C*12:03	DRB1*15:01	DQB1*06:01
	CONTIG2	A*31:01	B*41:02	C*17:03	DRB1*15:02	DQB1*06:02
HC0564	CONTIG1	A*02:01	B*27:02	C*02:02	DRB1*14:01	DQB1*05:02
	CONTIG2	A*26:01	B*38:01	C*12:03	DRB1*16:01	DQB1*05:03
HC0565	CONTIG1	A*02:01	B*27:05	C*02:02	DRB1*07:01	DQB1*02:02
	CONTIG2	A*24:02	B*44:03	C*04:01	DRB1*16:01	DQB1*05:02
HC0566	CONTIG1	A*02:01	B*07:02	C*07:02	DRB1*13:02	DQB1*06:02
	CONTIG2	A*29:01	B*07:05	C*15:05	DRB1*15:01	DQB1*06:04
HC0567	CONTIG1	A*02:01	B*51:01	C*12:02	DRB1*15:01	DQB1*05:02
	CONTIG2	-	B*52:01	C*14:02	DRB1*15:02	DQB1*06:01
HC0568	CONTIG1	A*02:01	B*44:02	C*05:01	DRB1*07:01	DQB1*02:02
	CONTIG2	A*23:01	B*51:01	C*15:02	DRB1*15:01	DQB1*06:02
HC0569	CONTIG1	A*01:01	B*13:02	C*06:02	DRB1*04:08	DQB1*02:02
	CONTIG2	A*25:01	B*35:03	C*12:03	DRB1*07:01	DQB1*03:04
HC0570	CONTIG1	A*02:01	B*13:02	C*02:02	DRB1*01:01	DQB1*02:02
	CONTIG2	A*24:02	B*27:05	C*06:02	DRB1*07:01	DQB1*05:01
HC0571	CONTIG1	A*03:01	B*07:02	C*07:02	DRB1*07:01	DQB1*03:03
	CONTIG2	A*32:01	B*58:01	-	DRB1*15:02	DQB1*06:01
HC0572	CONTIG1	A*02:01	B*15:01	C*03:04	DRB1*04:01	DQB1*03:01
	CONTIG2	A*68:01	B*57:01	C*06:02	DRB1*11:03	DQB1*03:02
HC0573	CONTIG1	A*01:01	B*18:01	C*07:01	DRB1*07:01	DQB1*02:02
	CONTIG2	A*26:01	B*58:01	C*07:18	DRB1*11:04	DQB1*03:01
HC0574	CONTIG1	A*01:01	B*08:01	C*03:03	DRB1*09:01	DQB1*03:03
	CONTIG2	A*02:01	B*15:01	C*07:01	DRB1*13:01	DQB1*06:03
HC0575	CONTIG1	A*24:02	B*15:01	C*03:03	DRB1*13:01	DQB1*05:03

	CONTIG2	-	B*18:01	C*07:01	DRB1*14:54	DQB1*06:03
HC0576	CONTIG1	A*29:02	B*18:01	C*03:02	DRB1*13:02	DQB1*05:03
	CONTIG2	A*33:03	B*58:01	C*07:01	DRB1*14:54	DQB1*06:09
HC0577	CONTIG1	A*01:01	B*40:01	C*03:03	DRB1*11:04	DQB1*03:01
	CONTIG2	A*11:01	B*55:01	C*03:19	DRB1*15:01	DQB1*06:02
HC0578	CONTIG1	A*11:01	B*14:02	C*04:01	DRB1*01:02	DQB1*02:01
	CONTIG2	A*30:02	B*35:01	C*08:02	DRB1*03:01	DQB1*05:01
HC0579	CONTIG1	A*02:01	B*27:05	C*02:02	DRB1*01:01	DQB1*05:01
	CONTIG2	A*26:01	-	-	-	-
HC0580	CONTIG1	A*24:02	B*38:01	C*03:03	DRB1*11:03	DQB1*03:01
	CONTIG2	A*26:01	B*55:01	C*12:03	DRB1*13:01	DQB1*06:03
HC0581	CONTIG1	A*03:01	B*07:02	C*03:03	DRB1*11:03	DQB1*03:01
	CONTIG2	A*24:02	B*15:01	C*07:02	DRB1*15:01	DQB1*06:02
HC0582	CONTIG1	A*03:01	B*35:01	C*03:04	DRB1*10:01	DQB1*03:01
	CONTIG2	A*25:01	B*40:01	C*04:01	DRB1*11:01	DQB1*05:01
HC0583	CONTIG1	A*29:01	B*15:01	C*03:03	DRB1*13:01	DQB1*02:01
	CONTIG2	A*31:01	B*44:02	C*05:01	-	DQB1*06:03
HC0584	CONTIG1	A*01:01	B*13:02	C*06:02	DRB1*07:01	DQB1*02:02
	CONTIG2	A*68:01	B*15:17	C*07:01	DRB1*15:01	DQB1*06:02
HC0585	CONTIG1	A*02:01	B*27:05	C*02:02	DRB1*01:01	DQB1*03:01
	CONTIG2	A*24:02	B*48:01	C*08:03	DRB1*12:01	DQB1*05:01
HC0586	CONTIG1	A*02:01	B*38:01	C*12:03	DRB1*04:02	DQB1*03:02
	CONTIG2	A*29:02	B*44:03	C*16:01	DRB1*15:01	DQB1*06:01
HC0587	CONTIG1	A*03:01	B*07:02	C*07:02	DRB1*15:01	DQB1*06:02
	CONTIG2	-	-	-	-	-
HC0588	CONTIG1	A*01:01	B*08:01	C*04:01	DRB1*03:01	DQB1*02:01
	CONTIG2	A*02:01	B*35:01	C*07:01	DRB1*04:02	DQB1*03:02
HC0589	CONTIG1	A*11:01	B*15:01	C*02:02	DRB1*07:01	DQB1*03:03
	CONTIG2	A*26:01	B*27:05	C*03:03	DRB1*13:01	DQB1*06:03
HC0590	CONTIG1	A*02:01	B*27:02	C*02:02	DRB1*16:01	DQB1*05:02
	CONTIG2	-	B*44:05	C*02:02	-	-
HC0591	CONTIG1	A*01:01	B*37:01	C*03:04	DRB1*03:01	DQB1*02:01
	CONTIG2	A*24:02	B*40:01	C*06:02	DRB1*15:01	DQB1*06:02
HC0592	CONTIG1	A*02:01	B*15:01	C*04:01	DRB1*04:02	DQB1*03:02
	CONTIG2	A*26:01	B*38:01	C*12:03	DRB1*08:01	DQB1*04:02
HC0593	CONTIG1	A*01:01	B*08:01	C*07:01	DRB1*03:01	DQB1*02:01
	CONTIG2	A*02:01	B*41:02	C*17:03	DRB1*13:03	DQB1*03:01
HC0594	CONTIG1	A*02:01	B*18:01	C*06:02	DRB1*03:01	DQB1*02:01
	CONTIG2	-	B*50:01	C*12:03	DRB1*16:01	DQB1*05:02
HC0595	CONTIG1	A*01:01	B*07:02	C*07:01	DRB1*03:01	DQB1*02:01
	CONTIG2	A*24:02	B*08:01	C*07:02	DRB1*12:01	DQB1*03:01
HC0596	CONTIG1	A*01:01	B*08:01	C*04:01	DRB1*01:01	DQB1*02:01
	CONTIG2	A*31:01	B*35:01	C*07:01	DRB1*03:01	DQB1*05:01
HC0597	CONTIG1	A*02:01	B*13:02	C*06:02	DRB1*07:01	DQB1*02:02
	CONTIG2	A*24:02	B*51:01	C*15:02	DRB1*11:01	DQB1*03:01
HC0598	CONTIG1	A*26:01	B*07:02	C*01:02	DRB1*07:01	DQB1*02:02
	CONTIG2	A*31:01	B*56:01	C*07:02	DRB1*08:01	DQB1*04:02
HC0599	CONTIG1	A*02:01	B*35:01	C*02:02	DRB1*14:54	DQB1*05:02
	CONTIG2	A*11:01	B*44:05	C*04:01	DRB1*16:01	DQB1*05:03
HC0600	CONTIG1	A*01:01	B*44:03	C*04:01	DRB1*07:01	DQB1*02:02

	CONTIG2	A*23:01	B*57:01	C*06:02	DRB1*11:01	DQB1*03:01
HC0601	CONTIG1	A*01:01	B*08:01	C*02:02	DRB1*12:01	DQB1*03:01
	CONTIG2	A*02:01	B*27:05	C*07:01	DRB1*15:01	DQB1*06:02
HC0602	CONTIG1	A*02:01	B*27:02	C*02:02	DRB1*13:02	DQB1*05:02
	CONTIG2	-	B*40:01	C*03:04	DRB1*16:01	DQB1*06:04
HC0603	CONTIG1	A*02:01	B*27:02	C*02:02	DRB1*11:01	DQB1*03:01
	CONTIG2	-	B*35:01	C*04:01	DRB1*16:01	DQB1*05:02
HC0604	CONTIG1	A*02:01	B*27:02	C*02:02	DRB1*11:01	DQB1*03:01
	CONTIG2	-	B*40:02	C*02:02	DRB1*16:01	DQB1*05:02
HC0605	CONTIG1	A*02:01	B*27:05	C*01:02	DRB1*09:01	DQB1*03:03
	CONTIG2	A*03:01	-	C*02:02	DRB1*15:01	DQB1*06:02
HC0606	CONTIG1	A*02:01	B*07:02	C*07:02	DRB1*03:01	DQB1*02:01
	CONTIG2	A*25:01	B*18:01	C*12:03	DRB1*14:54	DQB1*05:03
HC0607	CONTIG1	A*03:01	B*07:02	C*07:02	DRB1*11:01	DQB1*03:01
	CONTIG2	A*25:01	B*18:01	C*12:03	DRB1*15:01	DQB1*06:02
HC0608	CONTIG1	A*02:01	B*35:01	C*03:03	DRB1*08:01	DQB1*02:02
	CONTIG2	A*03:01	B*47:01	C*06:02	DRB1*13:03	DQB1*04:02
HC0609	CONTIG1	A*01:01	B*08:01	C*07:01	DRB1*03:01	DQB1*02:01
	CONTIG2	-	-	C*07:01	-	-
HC0610	CONTIG1	A*23:01	B*37:01	C*01:02	DRB1*13:01	DQB1*06:03
	CONTIG2	A*24:02	B*55:01	C*06:02	DRB1*13:01	-
HC0611	CONTIG1	A*02:01	B*35:01	C*04:01	DRB1*01:01	DQB1*03:01
	CONTIG2	A*24:02	B*44:02	C*16:04	DRB1*11:04	DQB1*05:01
HC0612	CONTIG1	A*01:01	B*40:01	C*03:04	DRB1*13:01	DQB1*06:03
	CONTIG2	A*02:01	B*44:29	C*05:01	DRB1*13:02	DQB1*06:04
HC0613	CONTIG1	A*25:01	B*14:02	C*08:02	DRB1*13:01	DQB1*06:03
	CONTIG2	A*33:01	B*18:01	C*12:03	DRB1*13:01	-
HC0614	CONTIG1	A*11:01	B*35:01	C*03:04	DRB1*01:01	DQB1*03:02
	CONTIG2	A*31:01	B*40:01	C*04:01	DRB1*04:04	DQB1*05:01
HC0615	CONTIG1	A*02:01	B*40:01	C*03:04	DRB1*09:01	DQB1*03:03
	CONTIG2	A*26:01	B*40:02	-	DRB1*13:02	DQB1*06:04
HC0616	CONTIG1	A*03:01	B*07:02	C*07:02	DRB1*13:03	DQB1*03:01
	CONTIG2	A*68:02	B*14:02	C*08:02	DRB1*14:01	DQB1*05:03
HC0617	CONTIG1	A*02:01	B*14:02	C*08:02	DRB1*01:02	DQB1*05:01
	CONTIG2	A*33:01	B*39:01	C*12:03	DRB1*16:01	DQB1*05:02
HC0618	CONTIG1	A*01:01	B*18:01	C*02:02	DRB1*11:04	DQB1*03:01
	CONTIG2	A*24:02	B*27:05	C*07:01	DRB1*12:01	DQB1*03:01
HC0619	CONTIG1	A*24:02	B*15:17	C*04:01	DRB1*01:01	DQB1*05:01
	CONTIG2	-	B*44:03	C*07:01	DRB1*15:02	DQB1*06:01
HC0620	CONTIG1	A*02:01	B*27:05	C*01:02	DRB1*01:01	DQB1*04:02
	CONTIG2	A*31:01	-	C*02:02	DRB1*08:01	DQB1*05:01
HC0621	CONTIG1	A*02:01	B*44:03	C*12:02	DRB1*07:01	DQB1*02:02
	CONTIG2	A*11:01	B*52:01	C*16:01	DRB1*14:54	DQB1*05:03
HC0622	CONTIG1	A*01:01	B*08:01	C*07:01	DRB1*03:01	DQB1*02:01
	CONTIG2	A*02:01	B*35:03	C*12:03	DRB1*04:08	DQB1*03:04
HC0623	CONTIG1	A*02:01	B*15:01	C*03:03	DRB1*12:01	DQB1*03:01
	CONTIG2	A*31:01	B*39:01	C*12:03	DRB1*13:01	DQB1*06:03
HC0624	CONTIG1	A*02:01	B*15:01	C*01:02	DRB1*01:03	DQB1*03:01
	CONTIG2	-	B*27:05	C*04:01	DRB1*07:01	DQB1*03:03
HC0625	CONTIG1	A*11:01	B*44:03	C*02:02	DRB1*07:01	DQB1*02:02

	CONTIG2	A*25:01	B*44:05	C*04:01	DRB1*11:04	DQB1*03:01
HC0626	CONTIG1	A*02:01	B*18:01	C*07:01	DRB1*11:04	DQB1*03:01
	CONTIG2	A*25:01	-	C*12:03	DRB1*15:01	DQB1*06:02
HC0627	CONTIG1	A*03:01	B*13:02	C*06:02	DRB1*07:01	DQB1*02:02
	CONTIG2	A*03:02	B*18:01	C*15:02	DRB1*11:01	DQB1*03:01
HC0628	CONTIG1	A*02:01	B*18:01	C*01:02	DRB1*01:01	DQB1*03:02
	CONTIG2	A*25:01	B*27:05	C*12:03	DRB1*04:01	DQB1*05:01
HC0629	CONTIG1	A*01:01	B*08:01	C*03:02	DRB1*13:02	DQB1*05:03
	CONTIG2	A*33:03	B*58:01	C*07:01	DRB1*14:54	DQB1*06:09
HC0630	CONTIG1	A*24:02	B*40:02	C*02:02	DRB1*11:01	DQB1*03:01
	CONTIG2	A*25:01	B*44:02	C*05:01	DRB1*14:54	DQB1*05:03
HC0631	CONTIG1	A*01:01	B*08:01	C*01:02	DRB1*01:01	DQB1*03:01
	CONTIG2	A*03:01	B*56:01	C*07:01	DRB1*11:01	DQB1*05:01
HC0632	CONTIG1	A*02:01	B*07:02	C*07:01	DRB1*11:04	DQB1*03:01
	CONTIG2	A*03:01	B*18:01	C*07:02	-	-
HC0633	CONTIG1	A*02:01	B*07:04	C*07:02	DRB1*15:01	DQB1*06:02
	CONTIG2	A*26:01	B*14:02	C*08:02	-	-
HC0634	CONTIG1	A*02:01	B*38:01	C*05:01	DRB1*11:01	DQB1*03:01
	CONTIG2	A*03:01	B*44:02	C*12:03	DRB1*13:01	DQB1*06:03
HC0635	CONTIG1	A*02:01	B*15:01	C*03:03	DRB1*11:04	DQB1*03:01
	CONTIG2	A*24:02	B*18:01	C*07:01	DRB1*14:04	DQB1*05:01
HC0636	CONTIG1	A*24:02	B*15:01	C*03:03	DRB1*07:01	DQB1*02:02
	CONTIG2	A*26:01	B*49:01	C*07:01	DRB1*07:01	-
HC0637	CONTIG1	A*02:01	B*35:03	C*03:04	DRB1*01:01	DQB1*04:02
	CONTIG2	A*68:01	B*40:01	C*04:01	DRB1*08:02	DQB1*05:01
HC0638	CONTIG1	A*01:01	B*07:02	C*06:02	DRB1*01:01	DQB1*05:01
	CONTIG2	A*24:02	B*13:02	C*07:02	DRB1*13:02	DQB1*06:04
HC0639	CONTIG1	A*26:01	B*38:01	C*06:02	DRB1*09:01	DQB1*02:02
	CONTIG2	A*29:02	B*45:01	C*12:03	DRB1*15:01	DQB1*06:03
HC0640	CONTIG1	A*26:01	B*38:01	C*04:01	DRB1*01:02	DQB1*05:01
	CONTIG2	A*32:01	B*44:03	C*12:03	DRB1*13:01	DQB1*06:03
HC0641	CONTIG1	A*02:01	B*15:01	C*04:01	DRB1*01:01	DQB1*04:02
	CONTIG2	A*26:01	B*49:01	C*07:01	DRB1*08:01	DQB1*05:04
HC0642	CONTIG1	A*01:01	B*08:01	C*06:02	DRB1*03:01	DQB1*02:01
	CONTIG2	A*11:01	B*57:01	C*07:01	DRB1*11:04	DQB1*03:01
HC0643	CONTIG1	A*03:01	B*07:02	C*04:01	DRB1*13:03	DQB1*03:01
	CONTIG2	A*24:02	B*49:01	C*07:02	DRB1*15:01	DQB1*06:02
HC0644	CONTIG1	A*11:01	B*14:02	C*06:02	DRB1*04:01	DQB1*02:02
	CONTIG2	A*23:01	B*50:01	C*08:02	DRB1*07:01	DQB1*03:02
HC0645	CONTIG1	A*01:01	B*08:01	C*06:02	DRB1*01:01	DQB1*02:02
	CONTIG2	A*24:02	B*13:02	C*07:01	DRB1*07:01	DQB1*05:01
HC0646	CONTIG1	A*23:01	B*27:05	C*03:04	DRB1*04:01	DQB1*02:02
	CONTIG2	A*66:01	B*44:03	C*04:01	DRB1*07:01	DQB1*03:02
HC0647	CONTIG1	A*26:01	B*27:05	C*02:02	DRB1*04:04	DQB1*03:01
	CONTIG2	A*32:01	B*40:01	C*03:04	DRB1*11:01	DQB1*03:02
HC0648	CONTIG1	A*03:01	B*13:02	C*03:04	DRB1*04:01	DQB1*03:01
	CONTIG2	A*24:02	B*40:02	C*06:02	DRB1*11:01	DQB1*03:02
HC0649	CONTIG1	A*02:01	B*15:01	C*03:03	DRB1*11:03	DQB1*03:01
	CONTIG2	A*24:02	B*44:27	C*07:04	DRB1*16:01	DQB1*05:02
HC0650	CONTIG1	A*11:01	B*35:01	C*04:01	DRB1*04:02	DQB1*03:02

	CONTIG2	A*26:01	B*38:01	C*12:03	DRB1*04:04	-
HC0651	CONTIG1	A*02:01	B*15:01	C*01:02	DRB1*09:01	DQB1*03:01
	CONTIG2	A*32:01	B*27:05	C*03:03	DRB1*11:01	DQB1*03:03
HC0652	CONTIG1	A*01:01	B*08:01	C*01:02	DRB1*01:01	DQB1*02:02
	CONTIG2	A*26:01	B*27:05	C*07:01	DRB1*07:01	DQB1*05:01
HC0653	CONTIG1	A*02:01	B*38:01	C*12:02	DRB1*07:01	DQB1*02:02
	CONTIG2	A*11:01	B*52:79	C*12:03	DRB1*13:01	DQB1*06:03
HC0654	CONTIG1	A*01:01	B*07:02	C*04:01	DRB1*13:02	DQB1*06:02
	CONTIG2	A*03:01	B*35:03	C*07:02	DRB1*15:01	DQB1*06:04
HC0655	CONTIG1	A*23:01	B*35:02	C*04:01	DRB1*07:01	DQB1*02:02
	CONTIG2	A*24:02	B*44:03	C*04:01	DRB1*11:04	DQB1*03:01
HC0656	CONTIG1	A*01:01	B*08:01	C*04:01	DRB1*03:01	DQB1*02:01
	CONTIG2	A*03:01	B*35:03	C*07:01	DRB1*04:03	DQB1*03:02
HC0657	CONTIG1	A*02:01	B*18:01	C*07:01	DRB1*04:01	DQB1*03:01
	CONTIG2	A*23:01	B*49:01	C*12:03	DRB1*11:01	DQB1*03:02
HC0658	CONTIG1	A*11:01	B*18:01	C*12:03	DRB1*04:08	DQB1*03:04
	CONTIG2	A*31:01	B*35:03	-	DRB1*15:01	DQB1*06:02
HC0659	CONTIG1	A*02:01	B*07:04	C*06:02	DRB1*07:01	DQB1*02:02
	CONTIG2	A*30:01	B*13:02	C*07:02	DRB1*15:01	DQB1*06:02
HC0660	CONTIG1	A*02:01	B*15:01	C*03:03	DRB1*04:08	DQB1*03:04
	CONTIG2	-	B*35:03	C*12:03	DRB1*08:01	DQB1*04:02
HC0661	CONTIG1	A*02:05	B*35:01	C*04:01	DRB1*01:01	DQB1*02:02
	CONTIG2	A*11:01	B*50:01	C*06:02	DRB1*07:01	DQB1*05:01
HC0662	CONTIG1	A*02:05	B*14:02	C*03:03	DRB1*01:02	DQB1*05:01
	CONTIG2	A*24:02	B*15:01	C*08:02	DRB1*13:01	DQB1*06:03
HC0663	CONTIG1	A*03:01	B*07:02	C*07:02	DRB1*07:01	DQB1*02:02
	CONTIG2	A*31:01	B*39:01	C*12:03	DRB1*11:01	DQB1*03:01
HC0664	CONTIG1	A*02:01	B*14:02	C*08:02	DRB1*01:02	DQB1*05:01
	CONTIG2	A*33:01	B*38:01	C*12:03	DRB1*13:01	DQB1*06:03
HC0665	CONTIG1	A*02:01	B*35:01	C*04:01	DRB1*01:01	DQB1*03:03
	CONTIG2	A*03:01	B*44:02	C*05:01	DRB1*07:01	DQB1*05:01
HC0666	CONTIG1	A*24:02	B*07:02	C*07:02	DRB1*11:04	DQB1*03:01
	CONTIG2	A*25:01	B*18:01	C*12:03	DRB1*15:01	DQB1*06:02
HC0667	CONTIG1	A*02:01	B*40:01	C*03:04	DRB1*07:01	DQB1*02:02
	CONTIG2	-	B*50:01	C*06:02	DRB1*07:01	-
HC0668	CONTIG1	A*03:01	B*38:01	C*02:02	DRB1*04:08	DQB1*03:04
	CONTIG2	A*26:01	B*40:02	C*12:03	DRB1*13:01	DQB1*06:03
HC0669	CONTIG1	A*02:01	B*08:01	C*02:02	DRB1*01:01	DQB1*02:01
	CONTIG2	A*26:01	B*27:05	C*07:01	DRB1*03:01	DQB1*05:01
HC0670	CONTIG1	A*02:06	B*07:02	C*07:02	DRB1*15:01	DQB1*06:02
	CONTIG2	A*11:01	-	-	-	-
HC0671	CONTIG1	A*01:01	B*08:01	C*07:01	DRB1*03:01	DQB1*02:01
	CONTIG2	A*02:01	B*18:01	C*07:01	DRB1*14:54	DQB1*05:03
HC0672	CONTIG1	A*01:01	B*18:01	C*04:01	DRB1*01:01	DQB1*03:02
	CONTIG2	A*25:01	B*39:01	C*12:03	DRB1*04:01	DQB1*05:01
HC0673	CONTIG1	A*11:01	B*35:01	C*04:01	DRB1*11:01	DQB1*03:01
	CONTIG2	A*31:01	B*38:01	C*12:03	DRB1*11:03	-
HC0674	CONTIG1	A*01:01	B*07:02	C*07:01	DRB1*03:01	DQB1*02:01
	CONTIG2	A*24:02	B*08:01	C*07:02	DRB1*07:01	DQB1*02:02
HC0675	CONTIG1	A*01:01	B*07:05	C*07:01	DRB1*03:01	DQB1*02:01

	CONTIG2	A*29:01	B*08:01	C*15:05	DRB1*10:01	DQB1*05:01
HC0676	CONTIG1	A*01:01	B*35:02	C*01:02	DRB1*11:04	DQB1*03:01
	CONTIG2	A*24:02	-	C*04:01	-	DQB1*03:01
HC0677	CONTIG1	A*02:01	B*44:03	C*04:01	DRB1*04:04	DQB1*03:01
	CONTIG2	A*11:01	B*51:01	C*15:02	DRB1*11:04	DQB1*03:02
HC0678	CONTIG1	A*03:01	B*15:01	C*03:04	DRB1*04:01	DQB1*03:02
	CONTIG2	A*24:02	B*49:01	C*07:01	-	-
HC0679	CONTIG1	A*02:01	B*18:01	C*05:01	DRB1*03:01	DQB1*02:01
	CONTIG2	A*68:01	-	C*07:01	DRB1*11:04	DQB1*03:01
HC0680	CONTIG1	A*02:01	B*07:02	C*07:02	DRB1*08:01	DQB1*04:02
	CONTIG2	A*03:01	B*51:01	C*14:02	DRB1*15:01	DQB1*06:02
HC0681	CONTIG1	A*02:01	B*18:01	C*07:01	DRB1*11:04	DQB1*03:01
	CONTIG2	A*03:01	B*51:01	C*14:02	DRB1*13:01	DQB1*06:03
HC0682	CONTIG1	A*02:01	B*08:01	C*06:02	DRB1*03:01	DQB1*02:01
	CONTIG2	A*24:02	B*57:01	C*07:01	DRB1*13:02	DQB1*06:04
HC0683	CONTIG1	A*01:01	B*35:02	C*06:02	DRB1*07:01	DQB1*02:02
	CONTIG2	A*02:01	B*51:01	C*14:02	DRB1*11:04	DQB1*03:01
HC0684	CONTIG1	A*02:01	B*18:01	C*05:01	DRB1*07:01	DQB1*02:02
	CONTIG2	A*32:01	B*44:02	C*12:03	DRB1*15:01	DQB1*06:02
HC0685	CONTIG1	A*01:01	B*07:02	C*03:03	DRB1*04:03	DQB1*03:01
	CONTIG2	A*02:01	B*35:01	C*07:02	DRB1*11:01	DQB1*03:02
HC0686	CONTIG1	A*01:01	B*35:02	C*04:01	DRB1*07:01	DQB1*03:01
	CONTIG2	A*02:01	B*57:01	C*06:02	DRB1*11:04	DQB1*03:03
HC0687	CONTIG1	A*01:01	B*08:01	C*07:01	DRB1*03:01	DQB1*02:01
	CONTIG2	A*26:01	B*38:01	C*12:03	DRB1*15:01	DQB1*06:03
HC0688	CONTIG1	A*68:01	B*07:02	C*04:01	DRB1*01:01	DQB1*05:01
	CONTIG2	A*68:01	B*35:03	C*07:02	DRB1*13:01	DQB1*06:03
HC0689	CONTIG1	A*03:01	B*07:02	C*07:02	DRB1*01:02	DQB1*05:01
	CONTIG2	A*24:02	B*14:02	C*08:02	DRB1*13:01	DQB1*06:03
HC0690	CONTIG1	A*02:01	B*15:01	C*03:03	DRB1*13:01	DQB1*03:01
	CONTIG2	A*02:06	B*41:02	C*17:03	DRB1*13:03	DQB1*06:03
HC0691	CONTIG1	A*01:01	B*38:01	C*07:01	DRB1*04:04	DQB1*03:02
	CONTIG2	A*24:02	B*51:01	C*12:03	DRB1*14:01	DQB1*05:03
HC0692	CONTIG1	A*01:01	B*08:01	C*07:01	DRB1*03:01	DQB1*02:01
	CONTIG2	A*02:01	B*38:01	C*12:03	DRB1*13:01	DQB1*06:03
HC0693	CONTIG1	A*24:02	B*44:02	C*07:04	DRB1*11:01	DQB1*03:01
	CONTIG2	A*25:01	B*44:27	C*12:03	DRB1*13:01	DQB1*06:03
HC0694	CONTIG1	A*02:01	B*13:02	C*06:02	DRB1*07:01	DQB1*02:02
	CONTIG2	-	B*18:01	C*07:01	DRB1*11:04	DQB1*03:01
HC0695	CONTIG1	A*03:01	B*27:05	C*01:02	DRB1*01:01	DQB1*05:01
	CONTIG2	-	B*56:01	-	DRB1*15:01	DQB1*06:02
HC0696	CONTIG1	A*02:05	B*07:02	C*07:01	DRB1*08:04	DQB1*03:01
	CONTIG2	A*24:02	B*41:01	C*07:02	DRB1*15:01	DQB1*06:02
HC0697	CONTIG1	A*02:01	B*27:05	C*02:02	DRB1*07:01	DQB1*03:03
	CONTIG2	A*11:01	B*57:01	C*06:02	DRB1*15:01	DQB1*06:02
HC0698	CONTIG1	A*03:01	B*40:01	C*03:04	DRB1*04:04	DQB1*02:02
	CONTIG2	A*31:01	B*44:03	C*16:01	DRB1*07:01	DQB1*03:02
HC0699	CONTIG1	A*01:01	B*07:02	C*06:02	DRB1*04:04	DQB1*03:01
	CONTIG2	A*11:01	B*57:01	C*07:02	DRB1*13:05	DQB1*03:02
HC0700	CONTIG1	A*02:01	B*07:02	C*07:01	DRB1*11:01	DQB1*03:01

	CONTIG2	A*03:01	B*18:01	C*07:02	DRB1*15:01	DQB1*06:02
HC0701	CONTIG1	A*01:01	B*08:01	C*02:02	DRB1*03:01	DQB1*02:01
	CONTIG2	A*02:01	B*27:02	C*07:01	DRB1*16:01	DQB1*05:02
HC0702	CONTIG1	A*03:01	B*07:02	C*04:01	DRB1*01:01	DQB1*02:02
	CONTIG2	A*24:02	B*35:01	C*07:02	DRB1*07:01	DQB1*05:01
HC0703	CONTIG1	A*11:01	B*07:02	C*12:03	DRB1*11:04	DQB1*03:01
	CONTIG2	A*25:01	B*18:01	C*15:02	DRB1*15:01	DQB1*06:02
HC0704	CONTIG1	A*02:05	B*35:03	C*04:01	DRB1*11:04	DQB1*03:01
	CONTIG2	A*03:01	B*50:01	C*06:02	DRB1*12:01	DQB1*03:01
HC0705	CONTIG1	A*24:02	B*08:01	C*04:01	DRB1*03:01	DQB1*02:01
	CONTIG2	A*26:01	B*44:03	C*07:02	DRB1*11:01	DQB1*03:01
HC0706	CONTIG1	A*03:01	B*07:02	C*07:01	DRB1*13:01	DQB1*06:02
	CONTIG2	A*11:01	B*51:01	C*07:02	DRB1*15:01	DQB1*06:03
HC0707	CONTIG1	A*23:01	B*07:02	C*02:02	DRB1*04:04	DQB1*03:02
	CONTIG2	A*31:01	B*44:02	C*07:02	DRB1*07:01	DQB1*03:03
HC0708	CONTIG1	A*01:01	B*08:01	C*04:01	DRB1*03:01	DQB1*02:01
	CONTIG2	A*24:02	B*44:03	C*07:01	DRB1*11:01	DQB1*03:01
HC0709	CONTIG1	A*01:01	B*08:01	C*07:01	DRB1*03:01	DQB1*02:01
	CONTIG2	A*30:01	B*18:01	-	DRB1*11:04	DQB1*03:01
HC0710	CONTIG1	A*24:02	B*18:01	C*02:02	DRB1*08:01	DQB1*03:01
	CONTIG2	A*25:01	-	C*12:03	DRB1*11:04	DQB1*04:02
HC0711	CONTIG1	A*01:01	B*44:02	C*05:01	DRB1*15:01	DQB1*06:01
	CONTIG2	A*02:01	B*52:01	C*12:02	DRB1*15:02	DQB1*06:02
HC0712	CONTIG1	A*02:01	B*08:01	C*06:02	DRB1*07:01	DQB1*02:02
	CONTIG2	-	B*13:02	C*07:01	DRB1*13:01	DQB1*06:03
HC0713	CONTIG1	A*02:01	B*15:01	C*01:02	DRB1*04:01	DQB1*03:01
	CONTIG2	A*31:01	B*40:02	C*03:04	DRB1*11:01	DQB1*03:02
HC0714	CONTIG1	A*02:01	B*49:01	C*07:01	DRB1*01:01	DQB1*03:01
	CONTIG2	A*26:01	B*51:01	C*15:02	DRB1*12:01	DQB1*05:04
HC0715	CONTIG1	A*03:01	B*07:02	C*07:02	DRB1*04:01	DQB1*03:02
	CONTIG2	A*68:01	B*18:01	-	DRB1*15:01	DQB1*06:02
HC0716	CONTIG1	A*01:01	B*08:01	C*07:01	DRB1*03:01	DQB1*02:01
	CONTIG2	A*25:01	B*18:01	C*12:03	DRB1*04:01	DQB1*03:02
HC0717	CONTIG1	A*11:01	B*07:02	C*03:04	DRB1*03:01	DQB1*02:01
	CONTIG2	A*68:01	B*40:01	C*07:02	DRB1*07:01	DQB1*02:02
HC0718	CONTIG1	A*24:02	B*07:02	C*04:01	DRB1*03:01	DQB1*02:01
	CONTIG2	A*68:01	B*35:03	C*07:02	DRB1*08:01	DQB1*03:02
HC0719	CONTIG1	A*01:01	B*40:02	C*02:02	DRB1*11:01	DQB1*03:01
	CONTIG2	A*26:01	B*52:01	C*12:02	DRB1*15:02	DQB1*06:01
HC0720	CONTIG1	A*02:01	B*07:02	C*07:02	DRB1*07:01	DQB1*02:02
	CONTIG2	A*03:01	B*44:03	C*16:01	DRB1*15:01	DQB1*06:02
HC0721	CONTIG1	A*02:01	B*51:01	C*02:02	DRB1*13:01	DQB1*06:02
	CONTIG2	-	B*55:01	C*03:03	DRB1*15:01	DQB1*06:03
HC0722	CONTIG1	A*24:02	B*18:01	C*07:01	DRB1*11:04	DQB1*03:01
	CONTIG2	A*31:01	B*41:02	C*17:03	DRB1*13:03	-
HC0723	CONTIG1	A*01:01	B*07:02	C*07:02	DRB1*12:01	DQB1*03:01
	CONTIG2	A*24:02	B*39:01	C*12:03	DRB1*15:01	DQB1*06:02
HC0724	CONTIG1	A*02:01	B*13:02	C*06:02	DRB1*07:01	DQB1*02:02
	CONTIG2	A*31:01	B*39:01	C*12:03	DRB1*14:54	DQB1*05:03
HC0725	CONTIG1	A*01:01	B*08:01	C*07:01	DRB1*03:01	DQB1*02:01

	CONTIG2	A*25:01	B*18:01	C*12:03	DRB1*15:01	DQB1*06:02
HC0726	CONTIG1	A*01:01	B*08:01	C*04:01	DRB1*01:01	DQB1*02:01
	CONTIG2	A*11:01	B*35:01	C*07:01	DRB1*03:01	DQB1*05:01
HC0727	CONTIG1	A*03:01	B*07:02	C*02:02	DRB1*11:01	DQB1*03:01
	CONTIG2	A*03:01	B*27:02	C*07:02	DRB1*15:01	DQB1*06:02
HC0728	CONTIG1	A*01:01	B*18:01	C*04:01	DRB1*13:02	DQB1*06:02
	CONTIG2	A*25:01	B*35:02	C*12:03	DRB1*15:01	DQB1*06:04
HC0729	CONTIG1	A*03:01	B*07:02	C*02:02	DRB1*13:01	DQB1*06:03
	CONTIG2	-	B*27:05	C*03:04	DRB1*13:02	DQB1*06:04
HC0730	CONTIG1	A*23:01	B*27:51	C*02:02	DRB1*01:01	DQB1*02:02
	CONTIG2	A*32:01	B*44:03	C*04:01	DRB1*07:01	DQB1*05:01
HC0731	CONTIG1	A*02:01	B*07:02	C*07:02	DRB1*04:02	DQB1*03:02
	CONTIG2	A*11:01	B*38:01	C*12:03	DRB1*08:01	DQB1*04:02
HC0732	CONTIG1	A*01:01	B*13:02	C*06:02	DRB1*07:01	DQB1*02:02
	CONTIG2	A*32:01	B*18:01	C*12:03	DRB1*15:01	DQB1*06:02
HC0733	CONTIG1	A*03:01	B*07:02	C*07:02	DRB1*15:01	DQB1*06:02
	CONTIG2	A*24:02	-	-	-	-
HC0734	CONTIG1	A*02:01	B*27:05	C*01:02	DRB1*01:01	DQB1*05:01
	CONTIG2	-	B*35:03	C*12:03	DRB1*14:54	DQB1*05:03
HC0735	CONTIG1	A*03:01	B*18:01	C*01:02	DRB1*11:04	DQB1*03:01
	CONTIG2	A*11:01	B*52:01	C*12:02	DRB1*15:02	DQB1*06:01
HC0736	CONTIG1	A*30:01	B*13:02	C*06:02	DRB1*07:01	DQB1*02:02
	CONTIG2	A*32:01	B*51:01	C*14:02	DRB1*13:02	DQB1*06:04
HC0737	CONTIG1	A*02:01	B*07:02	C*04:01	DRB1*01:03	DQB1*02:02
	CONTIG2	A*23:01	B*44:03	C*07:02	DRB1*07:01	DQB1*05:01
HC0738	CONTIG1	A*03:01	B*14:02	C*06:02	DRB1*03:01	DQB1*02:01
	CONTIG2	A*11:01	B*37:01	C*08:02	DRB1*11:04	DQB1*03:01
HC0739	CONTIG1	A*03:01	B*07:02	C*07:02	DRB1*07:01	DQB1*02:02
	CONTIG2	A*33:01	B*14:02	C*08:02	DRB1*11:01	DQB1*03:01
HC0740	CONTIG1	A*11:01	B*35:01	C*02:02	DRB1*01:01	DQB1*05:01
	CONTIG2	A*24:02	B*44:05	C*04:01	DRB1*11:03	-
HC0741	CONTIG1	A*02:01	B*44:02	C*08:02	DRB1*12:01	DQB1*03:01
	CONTIG2	A*32:01	B*44:03	C*16:01	DRB1*13:05	DQB1*05:01
HC0742	CONTIG1	A*01:01	B*08:01	C*01:02	DRB1*03:01	DQB1*02:01
	CONTIG2	A*02:01	B*27:05	C*07:01	DRB1*15:01	DQB1*06:02
HC0743	CONTIG1	A*03:01	B*18:01	C*07:01	DRB1*12:01	DQB1*03:01
	CONTIG2	A*31:01	B*38:01	C*12:03	DRB1*13:01	DQB1*06:03
HC0744	CONTIG1	A*02:01	B*38:01	C*06:02	DRB1*07:01	DQB1*02:02
	CONTIG2	A*03:01	B*50:01	C*12:03	DRB1*13:01	DQB1*06:03
HC0745	CONTIG1	A*02:01	B*35:02	C*04:01	DRB1*11:04	DQB1*03:01
	CONTIG2	A*32:01	B*39:06	C*12:03	DRB1*16:01	DQB1*05:02
HC0746	CONTIG1	A*23:01	B*14:01	C*03:02	DRB1*04:04	DQB1*03:02
	CONTIG2	A*30:04	B*58:01	C*08:02	DRB1*04:05	DQB1*04:02
HC0747	CONTIG1	A*02:01	B*08:01	C*07:02	DRB1*03:01	DQB1*02:01
	CONTIG2	A*26:01	B*39:01	-	DRB1*09:01	DQB1*03:03
HC0748	CONTIG1	A*02:01	B*27:02	C*02:02	DRB1*16:01	DQB1*05:02
	CONTIG2	A*26:01	B*55:01	C*03:03	-	-
HC0749	CONTIG1	A*03:02	B*40:01	C*03:04	DRB1*04:04	DQB1*02:02
	CONTIG2	A*68:01	B*50:01	C*06:02	DRB1*07:01	DQB1*03:02
HC0750	CONTIG1	A*02:01	B*08:01	C*07:01	DRB1*03:01	DQB1*02:01

	CONTIG2	A*25:01	B*18:01	C*12:03	DRB1*15:01	DQB1*06:02
HC0751	CONTIG1	A*02:01	B*07:02	C*05:01	DRB1*04:01	DQB1*03:02
	CONTIG2	A*03:01	B*44:02	C*07:02	DRB1*15:01	DQB1*06:02
HC0752	CONTIG1	A*02:01	B*40:01	C*03:04	DRB1*03:01	DQB1*02:01
	CONTIG2	A*68:01	B*49:01	C*07:01	DRB1*11:04	DQB1*03:01
HC0753	CONTIG1	A*01:01	B*27:05	C*01:02	DRB1*13:01	DQB1*06:02
	CONTIG2	A*24:02	B*57:01	C*06:02	DRB1*15:01	DQB1*06:03
HC0754	CONTIG1	A*02:01	B*07:02	C*07:02	DRB1*01:01	DQB1*05:01
	CONTIG2	A*03:01	B*07:06	C*15:05	DRB1*13:01	DQB1*06:03
HC0755	CONTIG1	A*02:01	B*07:02	C*07:02	DRB1*15:01	DQB1*06:02
	CONTIG2	A*25:01	B*18:01	C*12:03	-	-
HC0756	CONTIG1	A*01:01	B*08:01	C*07:01	DRB1*03:01	DQB1*02:01
	CONTIG2	-	-	-	DRB1*03:01	-
HC0757	CONTIG1	A*02:01	B*27:05	C*02:02	DRB1*04:01	DQB1*03:02
	CONTIG2	A*31:01	B*35:01	C*04:01	DRB1*14:01	DQB1*05:03
HC0758	CONTIG1	A*02:01	B*13:02	C*06:02	DRB1*07:01	DQB1*02:02
	CONTIG2	A*30:01	-	-	-	-
HC0759	CONTIG1	A*02:01	B*37:01	C*04:01	DRB1*07:01	DQB1*02:02
	CONTIG2	A*23:01	B*44:03	C*06:02	DRB1*15:01	DQB1*06:02
HC0760	CONTIG1	A*02:01	B*38:01	C*07:04	DRB1*04:02	DQB1*03:01
	CONTIG2	A*26:01	B*44:02	C*12:03	DRB1*11:01	DQB1*03:02
HC0761	CONTIG1	A*33:01	B*14:02	C*04:01	DRB1*01:02	DQB1*03:01
	CONTIG2	A*68:01	B*35:03	C*08:02	DRB1*12:01	DQB1*05:01
HC0762	CONTIG1	A*03:01	B*07:02	C*04:01	DRB1*11:01	DQB1*03:01
	CONTIG2	A*24:02	B*44:03	C*07:02	DRB1*15:01	DQB1*06:02
HC0763	CONTIG1	A*02:01	B*35:02	C*04:01	DRB1*01:01	DQB1*03:01
	CONTIG2	A*24:02	B*44:27	C*07:04	DRB1*11:04	DQB1*05:01
HC0764	CONTIG1	A*02:01	B*45:01	C*01:02	DRB1*08:01	DQB1*04:02
	CONTIG2	A*26:01	B*56:01	C*06:02	DRB1*10:01	DQB1*05:01
HC0765	CONTIG1	A*01:01	B*37:01	C*06:02	DRB1*11:01	DQB1*03:01
	CONTIG2	A*02:01	B*41:02	C*17:03	DRB1*13:03	-
HC0766	CONTIG1	A*03:01	B*35:01	C*04:01	DRB1*01:01	DQB1*02:01
	CONTIG2	A*11:01	B*44:02	C*05:01	DRB1*03:01	DQB1*05:104
HC0767	CONTIG1	A*03:02	B*18:01	C*06:02	DRB1*04:01	DQB1*02:02
	CONTIG2	A*25:01	B*50:01	C*12:03	DRB1*07:01	DQB1*03:02
HC0768	CONTIG1	A*02:01	B*18:01	C*07:01	DRB1*04:01	DQB1*03:01
	CONTIG2	A*25:01	B*18:01	C*12:03	DRB1*11:01	DQB1*03:02
HC0769	CONTIG1	A*01:01	B*18:01	C*06:02	DRB1*04:05	DQB1*03:01
	CONTIG2	A*31:01	B*47:01	C*07:01	DRB1*11:01	DQB1*03:02
HC0770	CONTIG1	A*01:01	B*08:01	C*04:01	DRB1*03:01	DQB1*02:01
	CONTIG2	A*24:02	B*44:03	C*07:01	DRB1*11:01	DQB1*03:01
HC0771	CONTIG1	A*02:01	B*07:02	C*06:02	DRB1*07:01	DQB1*02:02
	CONTIG2	-	B*13:02	C*07:02	DRB1*12:01	DQB1*03:01
HC0772	CONTIG1	A*11:01	B*13:02	C*04:01	DRB1*01:01	DQB1*04:02
	CONTIG2	A*30:01	B*35:01	C*06:02	DRB1*08:01	DQB1*05:01
HC0773	CONTIG1	A*01:01	B*35:02	C*05:32	DRB1*11:04	DQB1*03:01
	CONTIG2	A*24:02	B*44:02	C*06:02	DRB1*13:01	-
HC0774	CONTIG1	A*02:01	B*13:02	C*06:02	DRB1*07:01	DQB1*02:02
	CONTIG2	-	B*41:02	C*17:03	DRB1*15:01	DQB1*06:02
HC0775	CONTIG1	A*24:02	B*13:02	C*02:02	DRB1*01:01	DQB1*02:02

	CONTIG2	A*30:01	B*44:05	C*06:02	DRB1*07:01	DQB1*05:01
HC0776	CONTIG1	A*02:01	B*07:02	C*03:03	DRB1*11:01	DQB1*03:01
	CONTIG2	A*25:01	B*35:01	C*07:02	DRB1*15:01	DQB1*06:02
HC0777	CONTIG1	A*24:02	B*27:05	C*01:02	DRB1*03:01	DQB1*02:01
	CONTIG2	A*68:01	B*44:02	C*07:04	DRB1*11:01	DQB1*03:01
HC0778	CONTIG1	A*02:01	B*07:02	C*07:02	DRB1*08:01	DQB1*04:02
	CONTIG2	A*26:01	B*38:01	C*12:03	DRB1*13:01	DQB1*06:03
HC0779	CONTIG1	A*01:01	B*07:02	C*07:01	DRB1*03:01	DQB1*02:01
	CONTIG2	A*26:01	B*08:01	C*07:02	DRB1*14:54	DQB1*05:03
HC0780	CONTIG1	A*03:01	B*35:03	C*04:01	DRB1*03:01	DQB1*02:01
	CONTIG2	-	B*49:01	C*07:01	DRB1*04:03	DQB1*03:02
HC0781	CONTIG1	A*02:01	B*18:01	C*07:01	DRB1*03:01	DQB1*02:01
	CONTIG2	A*25:01	B*49:01	C*12:03	DRB1*11:01	DQB1*03:01
HC0782	CONTIG1	A*01:01	B*08:01	C*07:01	DRB1*03:01	DQB1*02:01
	CONTIG2	A*23:01	B*49:01	C*07:01	DRB1*11:04	DQB1*03:01
HC0783	CONTIG1	A*01:01	B*15:08	C*01:02	DRB1*03:01	DQB1*02:01
	CONTIG2	A*03:01	B*35:03	C*04:01	DRB1*04:03	DQB1*03:02
HC0784	CONTIG1	A*11:01	B*35:01	C*04:01	DRB1*07:01	DQB1*02:02
	CONTIG2	A*23:01	B*44:03	C*04:01	DRB1*11:03	DQB1*03:01
HC0785	CONTIG1	A*02:01	B*40:02	C*02:02	DRB1*01:01	DQB1*03:02
	CONTIG2	-	B*44:02	C*05:01	DRB1*04:03	DQB1*05:01
HC0786	CONTIG1	A*03:01	B*07:02	C*04:01	DRB1*13:02	DQB1*06:02
	CONTIG2	-	B*35:01	C*07:02	DRB1*15:01	DQB1*06:04
HC0787	CONTIG1	A*01:01	B*08:01	C*06:02	DRB1*03:01	DQB1*02:01
	CONTIG2	A*24:02	B*13:02	C*07:01	DRB1*11:01	DQB1*03:01
HC0788	CONTIG1	A*02:01	B*18:01	C*12:03	DRB1*15:01	DQB1*05:02
	CONTIG2	A*25:01	B*38:01	-	DRB1*16:01	DQB1*06:02
HC0789	CONTIG1	A*02:01	B*07:02	C*07:01	DRB1*01:01	DQB1*05:01
	CONTIG2	A*03:01	B*18:01	C*07:02	DRB1*14:54	DQB1*05:03
HC0790	CONTIG1	A*24:02	B*44:05	C*01:02	DRB1*01:01	DQB1*03:01
	CONTIG2	A*68:01	B*56:01	C*02:02	DRB1*08:01	DQB1*05:01
HC0791	CONTIG1	A*02:01	B*15:01	C*01:02	DRB1*08:01	DQB1*03:01
	CONTIG2	-	B*51:01	C*15:02	DRB1*11:01	DQB1*04:02
HC0792	CONTIG1	A*01:01	B*08:01	C*04:01	DRB1*04:03	DQB1*03:01
	CONTIG2	A*02:01	B*15:01	C*07:01	DRB1*12:01	DQB1*03:02
HC0793	CONTIG1	A*03:01	B*08:01	C*03:04	DRB1*03:01	DQB1*02:01
	CONTIG2	A*33:03	B*40:01	C*07:01	DRB1*04:04	DQB1*03:02
HC0794	CONTIG1	A*02:01	B*13:02	C*06:02	DRB1*07:01	DQB1*02:02
	CONTIG2	A*24:02	B*44:27	C*07:04	DRB1*16:01	DQB1*05:02
HC0795	CONTIG1	A*03:01	B*07:02	C*04:01	DRB1*01:01	DQB1*05:01
	CONTIG2	-	B*35:01	C*07:02	DRB1*15:01	DQB1*06:02
HC0796	CONTIG1	A*02:01	B*07:02	C*07:02	DRB1*08:01	DQB1*04:02
	CONTIG2	A*03:01	B*51:08	C*16:02	DRB1*13:02	DQB1*06:04
HC0797	CONTIG1	A*24:02	B*39:01	C*01:02	DRB1*01:01	DQB1*03:01
	CONTIG2	A*26:01	B*51:01	C*07:02	DRB1*11:01	DQB1*05:01
HC0798	CONTIG1	A*02:01	B*27:05	C*02:02	DRB1*01:01	DQB1*03:01
	CONTIG2	A*32:01	B*50:01	C*06:02	DRB1*11:04	DQB1*05:01
HC0799	CONTIG1	A*24:02	B*07:02	C*06:02	DRB1*01:01	DQB1*03:03
	CONTIG2	-	B*57:01	C*07:02	DRB1*07:01	DQB1*05:01
HC0800	CONTIG1	A*26:01	B*15:01	C*03:03	DRB1*13:01	DQB1*06:03

	CONTIG2	A*29:02	B*38:01	C*12:03	DRB1*13:01	-
HC0801	CONTIG1	A*24:02	B*44:02	C*01:02	DRB1*11:01	DQB1*03:01
	CONTIG2	A*26:01	B*51:01	C*03:03	DRB1*11:03	-
HC0802	CONTIG1	A*03:01	B*44:03	C*01:02	DRB1*07:01	DQB1*02:02
	CONTIG2	A*11:01	B*56:01	C*04:01	DRB1*15:01	DQB1*06:02
HC0803	CONTIG1	A*02:01	B*27:05	C*04:01	DRB1*01:01	DQB1*03:01
	CONTIG2	A*25:01	B*35:01	C*05:01	DRB1*11:01	DQB1*05:01
HC0804	CONTIG1	A*25:01	B*18:01	C*12:03	DRB1*12:01	DQB1*03:01
	CONTIG2	A*68:01	B*51:01	C*15:02	DRB1*13:01	DQB1*06:03
HC0805	CONTIG1	A*01:01	B*08:01	C*07:01	DRB1*03:01	DQB1*02:01
	CONTIG2	A*31:01	B*39:01	C*12:03	DRB1*15:01	DQB1*06:02
HC0806	CONTIG1	A*02:01	B*07:02	C*04:01	DRB1*01:01	DQB1*04:02
	CONTIG2	A*03:01	B*35:01	C*07:02	DRB1*08:01	DQB1*05:01
HC0807	CONTIG1	A*11:01	B*35:01	C*04:01	DRB1*01:01	DQB1*05:01
	CONTIG2	A*31:01	B*52:01	C*12:02	-	-
HC0808	CONTIG1	A*23:01	B*44:02	C*05:01	DRB1*03:01	DQB1*02:01
	CONTIG2	A*24:02	B*50:01	C*06:02	DRB1*07:01	DQB1*02:02
HC0809	CONTIG1	A*02:01	B*07:02	C*07:01	DRB1*11:02	DQB1*03:19
	CONTIG2	A*03:01	B*49:01	C*07:02	DRB1*15:01	DQB1*06:02
HC0810	CONTIG1	A*01:01	B*08:01	C*03:03	DRB1*03:01	DQB1*02:01
	CONTIG2	A*02:01	B*35:01	C*07:01	DRB1*08:01	DQB1*04:02
HC0811	CONTIG1	A*02:01	B*13:02	C*06:02	DRB1*07:01	DQB1*02:02
	CONTIG2	A*24:02	B*37:01	-	-	-
HC0812	CONTIG1	A*03:01	B*18:01	C*02:02	DRB1*01:01	DQB1*03:03
	CONTIG2	A*25:01	B*27:05	C*07:01	DRB1*09:01	DQB1*05:01
HC0813	CONTIG1	A*02:01	B*55:01	C*03:03	DRB1*14:54	DQB1*05:03
	CONTIG2	A*11:01	B*57:01	C*06:02	DRB1*15:01	DQB1*06:02
HC0814	CONTIG1	A*02:01	B*07:02	C*03:04	DRB1*01:01	DQB1*03:03
	CONTIG2	A*24:02	B*40:01	C*07:02	DRB1*07:01	DQB1*05:01
HC0815	CONTIG1	A*02:01	B*15:01	C*03:04	DRB1*04:01	DQB1*02:02
	CONTIG2	-	B*44:03	C*04:01	DRB1*07:01	DQB1*03:02
HC0816	CONTIG1	A*02:06	B*15:01	C*03:04	DRB1*04:01	DQB1*02:02
	CONTIG2	A*24:02	B*57:01	C*07:01	DRB1*04:05	DQB1*03:02
HC0817	CONTIG1	A*03:01	B*35:03	C*04:01	DRB1*07:01	DQB1*02:02
	CONTIG2	-	B*44:03	-	DRB1*12:01	DQB1*03:01
HC0818	CONTIG1	A*01:01	B*08:01	C*07:01	DRB1*03:01	DQB1*02:01
	CONTIG2	A*31:01	B*52:01	C*12:02	DRB1*15:02	DQB1*06:01
HC0819	CONTIG1	A*02:01	B*08:01	C*02:02	DRB1*01:01	DQB1*05:01
	CONTIG2	A*25:01	B*44:05	C*07:01	DRB1*16:01	DQB1*05:02
HC0820	CONTIG1	A*01:01	B*08:01	C*07:01	DRB1*03:01	DQB1*02:01
	CONTIG2	A*02:01	B*39:05	C*12:03	DRB1*16:01	DQB1*05:02
HC0821	CONTIG1	A*01:01	B*44:03	C*03:03	DRB1*07:01	DQB1*02:02
	CONTIG2	A*02:01	B*50:01	C*16:01	DRB1*07:01	-
HC0822	CONTIG1	A*02:01	B*35:08	C*03:04	DRB1*11:01	DQB1*03:01
	CONTIG2	A*24:07	B*40:01	C*04:01	DRB1*11:04	-
HC0823	CONTIG1	A*02:01	B*41:01	C*07:18	DRB1*03:01	DQB1*02:01
	CONTIG2	A*02:05	B*58:01	C*17:01	DRB1*03:01	-
HC0824	CONTIG1	A*01:01	B*08:01	C*07:01	DRB1*03:01	DQB1*02:01
	CONTIG2	A*02:01	B*41:01	C*17:01	DRB1*07:01	DQB1*03:03
HC0825	CONTIG1	A*03:01	B*07:02	C*07:01	DRB1*03:01	DQB1*02:01

	CONTIG2	A*32:01	B*08:01	C*07:02	DRB1*08:01	DQB1*04:02
HC0826	CONTIG1	A*02:01	B*07:02	C*07:02	DRB1*13:01	DQB1*06:02
	CONTIG2	-	B*38:01	C*12:03	DRB1*15:01	DQB1*06:03
HC0827	CONTIG1	A*02:01	B*18:01	C*02:02	DRB1*03:01	DQB1*02:01
	CONTIG2	A*11:01	B*27:05	C*07:01	DRB1*11:01	DQB1*03:01
HC0828	CONTIG1	A*24:02	B*07:02	C*01:02	DRB1*15:01	DQB1*06:02
	CONTIG2	A*31:01	-	C*07:02	-	-
HC0829	CONTIG1	A*03:01	B*07:02	C*07:02	DRB1*11:01	DQB1*03:01
	CONTIG2	A*25:01	B*39:01	C*12:03	DRB1*15:01	DQB1*06:02
HC0830	CONTIG1	A*02:01	B*39:06	C*04:01	DRB1*08:01	DQB1*03:01
	CONTIG2	A*24:02	B*44:03	C*07:02	DRB1*11:04	DQB1*04:02
HC0831	CONTIG1	A*02:01	B*40:02	C*03:04	DRB1*11:04	DQB1*03:01
	CONTIG2	-	B*51:01	C*14:02	DRB1*14:54	DQB1*05:03
HC0832	CONTIG1	A*01:01	B*08:01	C*07:01	DRB1*03:01	DQB1*02:01
	CONTIG2	A*26:01	B*38:01	C*12:03	DRB1*16:01	DQB1*05:02
HC0833	CONTIG1	A*01:01	B*08:01	C*02:02	DRB1*01:01	DQB1*02:02
	CONTIG2	A*32:01	B*40:02	C*07:01	DRB1*07:01	DQB1*05:01
HC0834	CONTIG1	A*03:01	B*08:01	C*02:02	DRB1*03:01	DQB1*02:01
	CONTIG2	A*24:02	B*27:02	C*07:01	DRB1*16:01	DQB1*05:02
HC0835	CONTIG1	A*02:01	B*18:01	C*07:02	DRB1*04:02	DQB1*02:02
	CONTIG2	A*33:01	B*45:01	C*12:03	DRB1*07:01	DQB1*03:02
HC0836	CONTIG1	A*02:01	B*07:02	C*07:02	DRB1*15:01	DQB1*05:02
	CONTIG2	A*03:01	B*51:01	C*16:02	DRB1*16:02	DQB1*06:02
HC0837	CONTIG1	A*01:01	B*08:01	C*03:04	DRB1*04:01	DQB1*03:02
	CONTIG2	A*25:01	B*15:01	C*07:01	-	-
HC0838	CONTIG1	A*01:01	B*08:01	C*07:01	DRB1*03:01	DQB1*02:01
	CONTIG2	A*25:01	B*18:01	C*12:03	DRB1*15:01	DQB1*06:02
HC0839	CONTIG1	A*02:01	B*07:02	C*07:02	DRB1*04:02	DQB1*03:02
	CONTIG2	A*24:56	B*51:01	C*15:13	DRB1*15:01	DQB1*06:02
HC0840	CONTIG1	A*03:02	B*44:03	C*04:01	DRB1*07:01	DQB1*02:02
	CONTIG2	A*23:01	B*52:01	C*12:02	DRB1*07:01	-
HC0841	CONTIG1	A*01:01	B*35:01	C*04:01	DRB1*03:01	DQB1*02:01
	CONTIG2	A*02:01	B*57:01	C*06:02	DRB1*14:54	DQB1*05:03
HC0842	CONTIG1	A*02:01	B*07:02	C*03:04	DRB1*01:01	DQB1*04:02
	CONTIG2	A*25:01	B*15:01	C*04:01	DRB1*08:01	DQB1*05:01
HC0843	CONTIG1	A*11:01	B*35:01	C*01:02	DRB1*01:01	DQB1*05:01
	CONTIG2	-	B*56:01	C*04:01	DRB1*15:01	DQB1*06:02
HC0844	CONTIG1	A*02:01	B*15:01	C*03:03	DRB1*11:01	DQB1*03:01
	CONTIG2	A*24:02	-	-	DRB1*13:01	DQB1*06:03
HC0845	CONTIG1	A*25:01	B*18:01	C*07:01	DRB1*01:01	DQB1*03:01
	CONTIG2	A*66:01	B*49:01	C*12:03	DRB1*11:01	DQB1*05:01
HC0846	CONTIG1	A*03:01	B*44:27	C*06:02	DRB1*11:01	DQB1*03:01
	CONTIG2	-	B*47:01	C*07:04	DRB1*13:01	DQB1*06:03
HC0847	CONTIG1	A*02:01	B*08:01	C*01:02	DRB1*03:01	DQB1*02:01
	CONTIG2	A*26:01	B*13:02	C*06:02	DRB1*07:01	DQB1*02:02
HC0848	CONTIG1	A*02:01	B*13:02	C*06:02	DRB1*04:01	DQB1*03:02
	CONTIG2	A*31:01	B*39:01	C*12:03	DRB1*04:04	-
HC0849	CONTIG1	A*02:01	B*15:01	C*01:02	DRB1*04:01	DQB1*02:02
	CONTIG2	A*26:01	B*56:01	C*03:04	DRB1*07:01	DQB1*03:02
HC0850	CONTIG1	A*01:01	B*08:01	C*07:01	DRB1*03:01	DQB1*02:01

	CONTIG2	A*30:01	B*38:01	C*12:03	DRB1*16:01	DQB1*05:02
HC0851	CONTIG1	A*02:01	B*27:05	C*02:02	DRB1*04:01	DQB1*03:01
	CONTIG2	A*02:188	B*45:01	C*06:02	DRB1*04:06	DQB1*04:02
HC0852	CONTIG1	A*01:01	B*14:01	C*06:02	DRB1*07:01	DQB1*02:02
	CONTIG2	A*02:01	B*57:01	C*08:02	DRB1*13:01	DQB1*06:03
HC0853	CONTIG1	A*01:01	B*35:01	C*04:01	DRB1*08:01	DQB1*04:02
	CONTIG2	A*25:01	B*57:01	C*06:02	DRB1*14:54	DQB1*05:03
HC0854	CONTIG1	A*03:01	B*15:01	C*03:03	DRB1*04:01	DQB1*03:01
	CONTIG2	A*31:01	B*44:02	C*05:01	DRB1*13:01	DQB1*06:03
HC0855	CONTIG1	A*02:01	B*15:01	C*03:03	DRB1*07:01	DQB1*03:03
	CONTIG2	-	B*57:01	C*06:02	DRB1*13:01	DQB1*06:03
HC0856	CONTIG1	A*01:01	B*08:01	C*05:01	DRB1*03:01	DQB1*02:01
	CONTIG2	A*02:01	B*18:01	C*07:01	-	-
HC0857	CONTIG1	A*01:01	B*08:01	C*07:01	DRB1*03:01	DQB1*02:01
	CONTIG2	A*25:01	B*18:01	C*12:03	DRB1*15:01	DQB1*06:02
HC0858	CONTIG1	A*24:02	B*55:01	C*03:02	DRB1*01:01	DQB1*05:01
	CONTIG2	A*26:01	B*58:01	C*03:03	DRB1*15:01	DQB1*06:02
HC0859	CONTIG1	A*01:01	B*27:02	C*01:02	DRB1*01:01	DQB1*05:01
	CONTIG2	A*02:01	B*56:01	C*02:02	DRB1*16:01	DQB1*05:02
HC0860	CONTIG1	A*02:01	B*41:01	C*07:01	DRB1*01:01	DQB1*05:01
	CONTIG2	A*26:01	B*49:01	C*17:01	DRB1*10:01	DQB1*05:04
HC0861	CONTIG1	A*01:01	B*37:01	C*06:02	DRB1*10:01	DQB1*03:01
	CONTIG2	A*02:35	B*44:27	C*07:04	DRB1*11:01	DQB1*05:01
HC0862	CONTIG1	A*03:01	B*07:02	C*07:01	DRB1*11:04	DQB1*03:01
	CONTIG2	A*26:01	B*18:01	C*07:02	DRB1*15:01	DQB1*06:02
HC0863	CONTIG1	A*01:01	B*18:01	C*04:01	DRB1*14:01	DQB1*05:03
	CONTIG2	A*24:02	B*35:01	C*07:01	DRB1*15:01	DQB1*06:02
HC0864	CONTIG1	A*68:01	B*40:01	C*01:02	DRB1*04:01	DQB1*03:01
	CONTIG2	-	B*56:01	C*03:04	DRB1*08:01	DQB1*03:01
HC0865	CONTIG1	A*24:02	B*40:01	C*03:04	DRB1*04:04	DQB1*02:02
	CONTIG2	A*31:01	B*44:03	C*04:01	DRB1*07:01	DQB1*03:02
HC0866	CONTIG1	A*02:01	B*13:02	C*06:02	DRB1*07:01	DQB1*02:02
	CONTIG2	-	B*52:01	C*12:02	DRB1*15:02	DQB1*06:01
HC0867	CONTIG1	A*01:01	B*08:01	C*07:01	DRB1*03:01	DQB1*02:01
	CONTIG2	A*68:01	B*15:17	C*07:01	DRB1*07:01	DQB1*02:02
HC0868	CONTIG1	A*02:01	B*38:01	C*12:02	DRB1*01:01	DQB1*05:01
	CONTIG2	A*68:01	B*52:01	C*12:03	-	-
HC0869	CONTIG1	A*01:01	B*08:01	C*07:01	DRB1*03:01	DQB1*02:01
	CONTIG2	A*68:02	B*39:10	C*12:03	DRB1*11:03	DQB1*03:01
HC0870	CONTIG1	A*01:01	B*49:01	C*06:02	DRB1*01:01	DQB1*03:03
	CONTIG2	A*26:01	B*57:01	C*07:01	DRB1*07:01	DQB1*05:04
HC0871	CONTIG1	A*01:01	B*51:01	C*02:02	DRB1*07:01	DQB1*02:02
	CONTIG2	A*03:01	B*57:01	C*06:02	DRB1*11:01	DQB1*03:01
HC0872	CONTIG1	A*03:01	B*35:03	C*01:02	DRB1*01:01	DQB1*05:01
	CONTIG2	A*11:01	B*56:01	C*04:01	DRB1*14:54	DQB1*05:03
HC0873	CONTIG1	A*01:01	B*08:01	C*04:01	DRB1*03:01	DQB1*02:01
	CONTIG2	A*24:03	B*35:02	C*07:01	DRB1*11:04	DQB1*03:01
HC0874	CONTIG1	A*02:01	B*18:03	C*07:01	DRB1*11:01	DQB1*03:01
	CONTIG2	A*24:14	B*38:01	C*12:03	DRB1*11:04	-
HC0875	CONTIG1	A*02:01	B*15:01	C*03:03	DRB1*03:01	DQB1*02:01

	CONTIG2	A*11:01	B*49:01	C*07:01	DRB1*11:02	DQB1*03:19
HC0876	CONTIG1	A*03:01	B*07:02	C*07:02	DRB1*01:01	DQB1*05:01
	CONTIG2	-	-	-	DRB1*15:01	DQB1*06:02
HC0877	CONTIG1	A*01:01	B*44:03	C*04:01	DRB1*07:01	DQB1*02:02
	CONTIG2	A*03:01	-	-	-	-
HC0878	CONTIG1	A*01:01	B*08:01	C*07:01	DRB1*03:01	DQB1*02:01
	CONTIG2	-	B*52:01	C*12:02	DRB1*15:02	DQB1*06:01
HC0879	CONTIG1	A*02:01	B*07:02	C*07:02	DRB1*13:03	DQB1*03:01
	CONTIG2	A*03:01	B*41:02	C*17:03	DRB1*13:03	DQB1*03:02
HC0880	CONTIG1	A*11:01	B*41:02	C*01:02	DRB1*04:03	DQB1*03:01
	CONTIG2	A*66:01	B*56:01	C*17:03	DRB1*11:01	DQB1*03:02
HC0881	CONTIG1	A*02:01	B*07:02	C*04:01	DRB1*07:01	DQB1*02:02
	CONTIG2	A*03:01	B*44:03	C*07:02	DRB1*15:01	DQB1*06:02
HC0882	CONTIG1	A*02:01	B*07:02	C*05:01	DRB1*04:07	DQB1*03:01
	CONTIG2	A*03:01	B*44:02	C*07:02	DRB1*12:01	DQB1*03:01
HC0883	CONTIG1	A*02:01	B*07:02	C*04:01	DRB1*07:01	DQB1*03:03
	CONTIG2	A*68:01	B*15:01	C*07:02	DRB1*15:01	DQB1*06:02
HC0884	CONTIG1	A*01:01	B*08:01	C*03:03	DRB1*03:01	DQB1*02:01
	CONTIG2	A*24:02	B*15:01	C*07:01	DRB1*11:03	DQB1*03:01
HC0885	CONTIG1	A*03:01	B*35:01	C*04:01	DRB1*07:01	DQB1*02:02
	CONTIG2	A*29:02	B*44:03	C*16:01	DRB1*12:01	DQB1*03:01
HC0886	CONTIG1	A*03:01	B*35:03	C*04:01	DRB1*03:01	DQB1*02:01
	CONTIG2	A*30:01	B*44:02	C*07:04	DRB1*08:01	DQB1*04:02
HC0887	CONTIG1	A*03:01	B*27:05	C*01:02	DRB1*01:01	DQB1*04:02
	CONTIG2	A*30:01	B*35:01	C*03:03	DRB1*08:01	DQB1*05:01
HC0888	CONTIG1	A*02:01	B*27:05	C*02:02	DRB1*01:01	DQB1*03:01
	CONTIG2	A*24:02	B*44:02	C*05:01	-	DQB1*05:01
HC0889	CONTIG1	A*24:02	B*15:01	C*03:03	DRB1*01:01	DQB1*05:01
	CONTIG2	-	B*39:06	C*07:02	DRB1*13:01	DQB1*06:03
HC0890	CONTIG1	A*02:01	B*44:02	C*01:02	DRB1*12:01	DQB1*03:01
	CONTIG2	A*29:02	B*49:01	C*07:01	DRB1*13:03	DQB1*05:01
HC0891	CONTIG1	A*02:01	B*27:02	C*02:02	DRB1*04:04	DQB1*03:02
	CONTIG2	A*68:01	B*44:02	C*07:04	DRB1*16:01	DQB1*05:02
HC0892	CONTIG1	A*02:01	B*15:01	C*03:04	DRB1*04:01	DQB1*03:02
	CONTIG2	A*26:01	B*35:01	C*04:01	DRB1*14:54	DQB1*05:03
HC0893	CONTIG1	A*01:01	B*18:01	C*06:02	DRB1*03:01	DQB1*02:01
	CONTIG2	A*03:01	B*57:01	C*07:01	DRB1*07:01	DQB1*03:03
HC0894	CONTIG1	A*01:01	B*08:01	C*03:02	DRB1*03:01	DQB1*02:01
	CONTIG2	A*24:02	B*58:01	C*07:01	-	-
HC0895	CONTIG1	A*25:01	B*18:01	C*12:03	DRB1*03:01	DQB1*02:01
	CONTIG2	-	-	-	DRB1*15:01	DQB1*06:02
HC0896	CONTIG1	A*02:01	B*27:02	C*01:02	DRB1*04:01	DQB1*03:02
	CONTIG2	A*24:02	B*55:01	C*02:02	DRB1*16:01	DQB1*05:02
HC0897	CONTIG1	A*02:01	B*44:05	C*01:02	DRB1*01:01	DQB1*03:02
	CONTIG2	A*26:01	B*51:01	C*02:02	DRB1*04:01	DQB1*05:01
HC0898	CONTIG1	A*24:02	B*38:01	C*12:03	DRB1*13:01	DQB1*05:02
	CONTIG2	A*26:01	B*39:01	-	DRB1*16:01	DQB1*06:03
HC0899	CONTIG1	A*02:01	B*15:01	C*03:03	DRB1*07:01	DQB1*02:02
	CONTIG2	A*02:05	B*50:01	C*06:02	DRB1*11:01	DQB1*03:01
HC0900	CONTIG1	A*02:01	B*07:02	C*02:02	DRB1*01:01	DQB1*05:01

	CONTIG2	A*32:01	B*27:05	C*07:46	DRB1*15:01	DQB1*06:02
HC0901	CONTIG1	A*01:01	B*08:01	C*01:02	DRB1*03:01	DQB1*02:01
	CONTIG2	A*02:01	B*56:01	C*07:01	DRB1*04:01	DQB1*03:01
HC0902	CONTIG1	A*02:01	B*07:02	C*02:02	DRB1*08:01	DQB1*03:01
	CONTIG2	A*26:01	B*40:02	C*07:02	DRB1*11:01	DQB1*04:02
HC0903	CONTIG1	A*02:01	B*07:02	C*07:02	DRB1*13:03	DQB1*03:01
	CONTIG2	A*24:02	B*41:02	C*17:03	DRB1*15:01	DQB1*06:02
HC0904	CONTIG1	A*01:01	B*44:02	C*05:01	DRB1*07:01	DQB1*03:03
	CONTIG2	A*02:01	B*57:01	C*06:02	DRB1*13:01	DQB1*06:03
HC0905	CONTIG1	A*03:01	B*07:02	C*04:01	DRB1*07:01	DQB1*02:02
	CONTIG2	A*23:01	B*44:03	C*07:02	DRB1*15:01	DQB1*06:02
HC0906	CONTIG1	A*03:01	B*07:02	C*07:01	DRB1*03:01	DQB1*02:01
	CONTIG2	A*23:01	B*08:01	C*07:02	DRB1*15:01	DQB1*06:02
HC0907	CONTIG1	A*01:01	B*08:01	C*03:03	DRB1*03:01	DQB1*02:01
	CONTIG2	A*24:02	B*15:01	C*07:01	DRB1*13:02	DQB1*06:04
HC0908	CONTIG1	A*02:01	B*38:01	C*12:03	DRB1*13:01	DQB1*06:03
	CONTIG2	A*02:629	-	-	-	-
HC0909	CONTIG1	A*02:01	B*51:01	C*01:02	DRB1*01:01	DQB1*05:01
	CONTIG2	-	B*56:01	C*14:02	DRB1*14:54	DQB1*05:03
HC0910	CONTIG1	A*03:01	B*07:02	C*07:02	DRB1*01:01	DQB1*05:01
	CONTIG2	A*24:02	B*39:06	C*07:02	DRB1*15:01	DQB1*06:02
HC0911	CONTIG1	A*02:01	B*18:01	C*03:03	DRB1*03:01	DQB1*02:01
	CONTIG2	A*30:02	B*55:01	C*05:01	DRB1*12:01	DQB1*03:01
HC0912	CONTIG1	A*02:01	B*35:01	C*04:01	DRB1*01:01	DQB1*03:03
	CONTIG2	A*11:01	B*51:01	C*14:02	DRB1*09:01	DQB1*05:01
HC0913	CONTIG1	A*02:01	B*07:02	C*01:02	DRB1*07:01	DQB1*03:03
	CONTIG2	A*03:01	B*51:01	C*07:02	DRB1*15:01	DQB1*06:03
HC0914	CONTIG1	A*23:01	B*07:05	C*04:01	DRB1*07:01	DQB1*02:02
	CONTIG2	A*30:01	B*44:03	C*15:05	DRB1*11:01	DQB1*03:01
HC0915	CONTIG1	A*02:01	B*27:05	C*01:02	DRB1*01:03	DQB1*02:02
	CONTIG2	A*23:01	B*44:03	C*04:01	DRB1*07:01	DQB1*03:01
HC0916	CONTIG1	A*01:01	B*08:01	C*02:02	DRB1*03:01	DQB1*02:01
	CONTIG2	A*24:02	B*40:02	C*07:01	DRB1*11:01	DQB1*03:01
HC0917	CONTIG1	A*02:01	B*14:02	C*01:02	DRB1*01:02	DQB1*03:01
	CONTIG2	A*33:01	B*27:05	C*08:02	DRB1*01:02	DQB1*05:01
HC0918	CONTIG1	A*01:01	B*08:01	C*02:02	DRB1*03:01	DQB1*02:01
	CONTIG2	A*32:01	B*40:02	C*07:01	DRB1*11:01	DQB1*03:01
HC0919	CONTIG1	A*02:01	B*07:02	C*06:02	DRB1*07:01	DQB1*03:03
	CONTIG2	A*03:01	B*57:01	C*07:02	DRB1*15:01	DQB1*06:02
HC0920	CONTIG1	A*02:01	B*07:02	C*03:04	DRB1*13:02	DQB1*03:01
	CONTIG2	A*31:01	B*40:01	C*07:02	DRB1*13:03	DQB1*06:09
HC0921	CONTIG1	A*02:01	B*15:01	C*03:03	DRB1*04:07	DQB1*03:01
	CONTIG2	A*24:02	B*49:01	C*07:01	DRB1*11:01	DQB1*03:01
HC0922	CONTIG1	A*02:01	B*15:01	C*03:04	DRB1*04:01	DQB1*03:02
	CONTIG2	A*31:01	B*47:01	C*06:02	DRB1*04:05	-
HC0923	CONTIG1	A*02:01	B*18:01	C*12:03	DRB1*11:01	DQB1*03:01
	CONTIG2	A*25:01	B*51:01	C*15:02	DRB1*15:01	DQB1*06:02
HC0924	CONTIG1	A*03:01	B*07:02	C*07:02	DRB1*04:04	DQB1*03:02
	CONTIG2	A*11:01	B*51:01	C*15:02	DRB1*15:01	DQB1*06:02
HC0925	CONTIG1	A*02:01	B*35:03	C*04:01	DRB1*04:03	DQB1*02:02

	CONTIG2	A*31:01	B*48:01	C*08:03	DRB1*07:01	DQB1*03:02
HC0926	CONTIG1	A*01:01	B*18:01	C*04:01	DRB1*01:01	DQB1*03:02
	CONTIG2	A*25:01	B*35:01	C*12:03	DRB1*04:01	DQB1*05:01
HC0927	CONTIG1	A*24:02	B*14:02	C*02:02	DRB1*01:01	DQB1*03:01
	CONTIG2	-	B*18:01	C*12:03	DRB1*11:04	DQB1*05:01
HC0928	CONTIG1	A*02:05	B*39:01	C*01:02	DRB1*07:01	DQB1*03:03
	CONTIG2	A*11:01	B*56:01	C*12:03	DRB1*13:01	DQB1*06:03
HC0929	CONTIG1	A*02:01	B*38:01	C*02:02	DRB1*13:01	DQB1*05:02
	CONTIG2	A*26:01	B*44:05	C*12:03	DRB1*15:01	DQB1*06:03
HC0930	CONTIG1	A*02:01	B*47:01	C*06:02	DRB1*01:01	DQB1*05:01
	CONTIG2	A*03:01	B*52:01	C*12:02	-	-
HC0931	CONTIG1	A*25:01	B*18:01	C*12:03	DRB1*01:01	DQB1*05:01
	CONTIG2	-	B*18:01	-	DRB1*15:01	DQB1*06:02
HC0932	CONTIG1	A*01:01	B*35:03	C*12:03	DRB1*13:01	DQB1*05:03
	CONTIG2	A*26:01	B*51:01	-	DRB1*14:54	DQB1*06:03
HC0933	CONTIG1	A*02:01	B*15:01	C*04:01	DRB1*08:01	DQB1*03:01
	CONTIG2	A*23:01	B*49:01	C*07:01	DRB1*11:01	DQB1*04:02
HC0934	CONTIG1	A*02:01	B*40:01	C*03:04	DRB1*04:04	DQB1*03:02
	CONTIG2	A*32:01	-	-	DRB1*04:04	DQB1*03:02
HC0935	CONTIG1	A*02:01	B*27:05	C*02:02	DRB1*11:01	DQB1*03:01
	CONTIG2	A*03:01	B*44:02	C*07:04	-	-
HC0936	CONTIG1	A*03:01	B*27:02	C*02:02	DRB1*11:01	DQB1*03:01
	CONTIG2	A*32:01	B*35:03	C*04:01	DRB1*15:01	DQB1*06:02
HC0937	CONTIG1	A*01:01	B*08:01	C*07:01	DRB1*03:01	DQB1*02:01
	CONTIG2	A*02:01	B*44:27	C*07:04	DRB1*16:01	DQB1*05:02
HC0938	CONTIG1	A*01:01	B*08:01	C*05:01	DRB1*03:01	DQB1*02:01
	CONTIG2	A*02:01	B*44:02	C*07:01	DRB1*04:01	DQB1*03:01
HC0939	CONTIG1	A*24:02	B*15:01	C*03:04	DRB1*01:01	DQB1*03:02
	CONTIG2	A*33:03	B*44:03	C*16:02	DRB1*04:01	DQB1*05:01
HC0940	CONTIG1	A*24:02	B*13:02	C*01:02	DRB1*15:01	DQB1*05:02
	CONTIG2	A*29:01	B*51:01	C*06:02	DRB1*16:01	DQB1*06:02
HC0941	CONTIG1	A*01:01	B*51:01	C*01:02	DRB1*01:01	DQB1*05:01
	CONTIG2	A*02:01	B*57:01	C*06:02	DRB1*16:01	DQB1*05:02
HC0942	CONTIG1	A*02:01	B*07:02	C*07:02	DRB1*01:03	DQB1*03:01
	CONTIG2	A*31:01	B*51:01	C*15:02	DRB1*11:01	DQB1*05:01
HC0943	CONTIG1	A*02:01	B*13:02	C*06:02	DRB1*04:01	DQB1*03:01
	CONTIG2	-	B*14:01	C*08:02	DRB1*13:02	DQB1*06:04
HC0944	CONTIG1	A*30:01	B*38:01	C*03:04	DRB1*04:08	DQB1*03:01
	CONTIG2	A*31:01	B*40:01	C*12:03	DRB1*13:03	DQB1*03:01
HC0945	CONTIG1	A*02:01	B*38:01	C*02:02	DRB1*11:01	DQB1*03:01
	CONTIG2	A*24:02	B*40:02	C*12:03	DRB1*13:01	DQB1*06:03
HC0946	CONTIG1	A*02:01	B*13:02	C*06:02	DRB1*07:01	DQB1*02:02
	CONTIG2	A*29:02	B*44:03	C*16:01	DRB1*15:01	DQB1*06:02
HC0947	CONTIG1	A*26:01	B*35:03	C*04:01	DRB1*01:01	DQB1*03:02
	CONTIG2	A*68:01	B*38:01	C*12:03	DRB1*04:02	DQB1*05:01
HC0948	CONTIG1	A*25:01	B*18:01	C*07:01	DRB1*11:01	DQB1*03:01
	CONTIG2	A*32:01	B*49:01	C*12:03	DRB1*16:01	DQB1*05:02
HC0949	CONTIG1	A*02:01	B*35:03	C*06:02	DRB1*10:01	DQB1*05:01
	CONTIG2	A*03:01	B*37:01	C*12:03	DRB1*15:01	DQB1*06:02
HC0950	CONTIG1	A*01:01	B*07:02	C*03:02	DRB1*03:01	DQB1*02:01

	CONTIG2	A*02:01	B*08:01	C*07:01	DRB1*15:01	DQB1*06:02
HC0951	CONTIG1	A*02:01	B*18:01	C*07:04	DRB1*11:01	DQB1*03:01
	CONTIG2	A*25:01	-	C*12:03	DRB1*16:01	DQB1*05:02
HC0952	CONTIG1	A*03:01	B*18:01	C*04:01	DRB1*11:04	DQB1*03:01
	CONTIG2	A*32:01	B*35:02	C*12:03	-	DQB1*03:01
HC0953	CONTIG1	A*01:01	B*08:01	C*06:02	DRB1*03:01	DQB1*02:01
	CONTIG2	A*02:05	B*50:01	C*07:01	DRB1*07:01	DQB1*02:02
HC0954	CONTIG1	A*24:02	B*18:01	C*02:02	DRB1*11:01	DQB1*03:01
	CONTIG2	A*24:02	B*40:02	C*12:03	-	-
HC0955	CONTIG1	A*01:01	B*07:02	C*06:02	DRB1*04:01	DQB1*03:01
	CONTIG2	A*02:01	B*57:01	C*07:02	DRB1*11:01	DQB1*03:02
HC0956	CONTIG1	A*02:05	B*15:01	C*04:01	DRB1*07:01	DQB1*02:02
	CONTIG2	A*03:01	B*50:01	C*06:02	DRB1*13:01	DQB1*06:03
HC0957	CONTIG1	A*26:01	B*07:02	C*07:01	DRB1*13:02	DQB1*06:02
	CONTIG2	A*32:01	B*49:01	C*07:02	DRB1*15:01	DQB1*06:04
HC0958	CONTIG1	A*02:01	B*13:02	C*05:01	DRB1*12:01	DQB1*03:01
	CONTIG2	A*30:01	B*44:02	C*06:02	DRB1*13:01	DQB1*06:03
HC0959	CONTIG1	A*23:01	B*44:03	C*04:01	DRB1*01:01	DQB1*02:01
	CONTIG2	-	B*49:01	C*07:01	DRB1*03:01	DQB1*05:01
HC0960	CONTIG1	A*03:01	B*07:02	C*05:01	DRB1*13:01	DQB1*06:03
	CONTIG2	A*31:01	B*44:02	C*07:02	DRB1*15:01	DQB1*06:39
HC0961	CONTIG1	A*02:01	B*18:01	C*12:03	DRB1*04:01	DQB1*03:01
	CONTIG2	A*25:01	B*18:01	-	DRB1*04:08	DQB1*03:02
HC0962	CONTIG1	A*02:01	B*18:01	C*04:01	DRB1*11:01	DQB1*03:01
	CONTIG2	A*03:01	B*35:02	C*07:01	DRB1*11:04	DQB1*03:01
HC0963	CONTIG1	A*02:01	B*13:02	C*05:01	DRB1*07:01	DQB1*02:02
	CONTIG2	A*24:14	B*44:02	C*06:02	DRB1*08:01	DQB1*04:02
HC0964	CONTIG1	A*02:01	B*27:02	C*02:02	DRB1*11:01	DQB1*03:01
	CONTIG2	A*24:02	B*39:01	C*12:03	DRB1*13:01	DQB1*06:03
HC0965	CONTIG1	A*25:01	B*18:01	C*03:02	DRB1*03:01	DQB1*02:01
	CONTIG2	A*68:01	B*58:01	C*07:01	DRB1*13:01	DQB1*06:03
HC0966	CONTIG1	A*01:01	B*07:02	C*07:01	DRB1*01:01	DQB1*03:01
	CONTIG2	A*29:02	B*08:01	C*07:02	DRB1*11:01	DQB1*05:01
HC0967	CONTIG1	A*03:01	B*35:01	C*12:03	DRB1*01:01	DQB1*03:02
	CONTIG2	A*26:01	B*38:01	C*15:02	DRB1*04:02	DQB1*05:01
HC0968	CONTIG1	A*03:01	B*15:01	C*02:02	DRB1*04:04	DQB1*03:02
	CONTIG2	A*32:01	B*40:02	C*04:01	DRB1*14:54	DQB1*05:03
HC0969	CONTIG1	A*01:01	B*08:01	C*03:03	DRB1*03:01	DQB1*05:01
	CONTIG2	A*02:01	B*15:01	C*07:01	DRB1*13:01	DQB1*06:03
HC0970	CONTIG1	A*01:01	B*08:01	C*07:01	DRB1*03:01	DQB1*02:01
	CONTIG2	A*02:01	B*39:24	-	DRB1*13:03	DQB1*03:01
HC0971	CONTIG1	A*24:02	B*27:05	C*01:02	DRB1*01:01	DQB1*03:02
	CONTIG2	A*31:01	B*35:01	C*04:01	DRB1*04:02	DQB1*05:01
HC0972	CONTIG1	A*01:01	B*08:01	C*01:02	DRB1*03:01	DQB1*02:01
	CONTIG2	A*02:01	B*56:01	C*07:01	DRB1*04:01	DQB1*03:01
HC0973	CONTIG1	A*02:01	B*15:01	C*03:03	DRB1*11:01	DQB1*03:01
	CONTIG2	A*32:01	B*44:27	C*07:04	DRB1*16:01	DQB1*06:03
HC0974	CONTIG1	A*01:01	B*08:01	C*04:01	DRB1*03:01	DQB1*02:53Q
	CONTIG2	A*02:01	B*15:01	C*07:01	DRB1*04:03	DQB1*03:02
HC0975	CONTIG1	A*02:01	B*27:02	C*02:02	DRB1*13:03	DQB1*03:01

	CONTIG2	-	B*41:02	C*17:03	DRB1*16:01	DQB1*05:02
HC0976	CONTIG1	A*01:01	B*07:02	C*06:02	DRB1*13:05	DQB1*03:01
	CONTIG2	A*03:01	B*57:01	C*07:02	DRB1*15:01	DQB1*06:02
HC0977	CONTIG1	A*01:01	B*08:01	C*07:01	DRB1*03:01	DQB1*02:01
	CONTIG2	A*02:01	B*41:02	C*17:03	DRB1*11:01	-
HC0978	CONTIG1	A*02:01	B*15:01	C*02:02	DRB1*16:01	DQB1*05:02
	CONTIG2	A*31:01	B*27:05	C*03:04	-	-
HC0979	CONTIG1	A*01:01	B*07:02	C*07:02	DRB1*12:01	DQB1*03:01
	CONTIG2	A*24:02	B*39:01	C*12:03	DRB1*15:01	DQB1*06:02
HC0980	CONTIG1	A*02:01	B*35:01	C*03:04	DRB1*01:01	DQB1*05:01
	CONTIG2	A*11:01	B*40:01	C*04:01	DRB1*13:02	DQB1*06:04
HC0981	CONTIG1	A*03:01	B*14:02	C*01:02	DRB1*01:02	DQB1*05:01
	CONTIG2	A*33:01	B*56:01	C*08:02	DRB1*13:02	DQB1*06:04
HC0982	CONTIG1	A*01:01	B*08:01	C*04:01	DRB1*03:01	DQB1*02:01
	CONTIG2	A*26:01	B*35:01	C*07:01	-	-
HC0983	CONTIG1	A*03:01	B*18:01	C*04:01	DRB1*01:01	DQB1*03:01
	CONTIG2	A*11:01	B*35:01	C*07:01	DRB1*11:04	DQB1*05:01
HC0984	CONTIG1	A*25:01	B*13:02	C*06:02	DRB1*15:01	DQB1*05:02
	CONTIG2	A*30:01	B*18:01	C*12:03	DRB1*16:01	DQB1*06:02
HC0985	CONTIG1	A*02:12	B*35:01	C*04:01	DRB1*01:01	DQB1*05:01
	CONTIG2	A*03:01	B*52:01	C*12:02	DRB1*15:02	DQB1*06:01
HC0986	CONTIG1	A*02:01	B*49:01	C*07:01	DRB1*03:01	DQB1*02:01
	CONTIG2	A*23:01	B*51:01	C*15:02	DRB1*13:01	DQB1*06:03
HC0987	CONTIG1	A*24:02	B*35:03	C*04:01	DRB1*11:04	DQB1*03:01
	CONTIG2	A*26:01	B*44:02	C*05:01	DRB1*16:01	DQB1*05:02
HC0988	CONTIG1	A*01:01	B*39:01	C*12:03	DRB1*11:01	DQB1*03:01
	CONTIG2	A*31:01	B*41:02	C*17:03	DRB1*13:03	-
HC0989	CONTIG1	A*01:01	B*35:02	C*04:01	DRB1*11:04	DQB1*03:01
	CONTIG2	A*11:01	B*51:01	C*15:02	DRB1*12:01	DQB1*06:03
HC0990	CONTIG1	A*03:01	B*35:03	C*04:01	DRB1*07:01	DQB1*02:02
	CONTIG2	A*23:01	B*44:03	C*07:02	DRB1*13:01	DQB1*06:03
HC0991	CONTIG1	A*02:01	B*44:02	C*05:01	DRB1*11:01	DQB1*03:01
	CONTIG2	A*29:02	B*44:04	C*16:01	DRB1*13:01	DQB1*06:03
HC0992	CONTIG1	A*02:01	B*27:05	C*02:02	DRB1*04:04	DQB1*03:01
	CONTIG2	-	B*44:02	C*07:04	DRB1*11:01	DQB1*03:02
HC0993	CONTIG1	A*02:01	B*35:03	C*03:04	DRB1*11:01	DQB1*03:01
	CONTIG2	A*32:01	B*40:01	C*04:01	DRB1*11:04	-
HC0994	CONTIG1	A*11:01	B*51:01	C*15:02	DRB1*04:04	DQB1*03:02
	CONTIG2	A*24:02	B*51:01	-	DRB1*13:01	DQB1*06:03
HC0995	CONTIG1	A*02:01	B*27:05	C*02:02	DRB1*01:01	DQB1*03:01
	CONTIG2	A*29:02	B*45:01	C*06:02	DRB1*04:01	DQB1*05:01
HC0996	CONTIG1	A*02:01	B*18:01	C*03:04	DRB1*13:02	DQB1*06:02
	CONTIG2	A*25:01	B*40:01	C*12:03	DRB1*15:01	DQB1*06:04
HC0997	CONTIG1	A*02:01	B*15:01	C*03:03	DRB1*11:01	DQB1*03:01
	CONTIG2	A*25:01	B*18:01	C*12:03	DRB1*13:01	DQB1*06:03
HC0998	CONTIG1	A*24:02	B*13:02	C*02:02	DRB1*07:01	DQB1*02:02
	CONTIG2	A*32:01	B*27:05	C*06:02	DRB1*12:01	DQB1*03:01
HC0999	CONTIG1	A*02:01	B*13:02	C*06:02	DRB1*04:01	DQB1*02:02
	CONTIG2	-	B*45:01	C*06:02	DRB1*07:01	DQB1*03:01
HC1000	CONTIG1	A*02:01	B*15:01	C*04:01	DRB1*11:03	DQB1*03:01

	CONTIG2	A*32:01	B*18:01	C*07:01	DRB1*16:02	DQB1*05:02
HC1001	CONTIG1	A*01:01	B*08:01	C*01:02	DRB1*03:01	DQB1*02:01
	CONTIG2	A*02:01	B*27:05	C*07:01	DRB1*15:01	DQB1*06:02
HC1002	CONTIG1	A*01:01	B*35:03	C*04:01	DRB1*07:01	DQB1*03:01
	CONTIG2	A*02:01	B*57:01	C*06:02	DRB1*11:04	DQB1*03:03
HC1003	CONTIG1	A*02:01	B*45:01	C*04:01	DRB1*11:02	DQB1*03:19
	CONTIG2	A*68:02	B*53:01	C*06:02	DRB1*13:02	DQB1*06:04
HC1004	CONTIG1	A*01:01	B*08:01	C*04:01	DRB1*03:01	DQB1*02:01
	CONTIG2	A*24:02	B*44:03	C*07:01	DRB1*07:01	DQB1*02:02
HC1005	CONTIG1	A*01:01	B*13:02	C*06:02	DRB1*07:01	DQB1*02:02
	CONTIG2	-	B*44:02	C*07:04	DRB1*11:01	DQB1*03:01
HC1006	CONTIG1	A*03:01	B*35:03	C*04:01	DRB1*08:01	DQB1*03:02
	CONTIG2	A*68:01	B*44:02	C*07:04	DRB1*15:01	DQB1*06:02
HC1007	CONTIG1	A*03:01	B*07:02	C*07:02	DRB1*04:01	DQB1*03:02
	CONTIG2	A*25:01	-	-	DRB1*08:01	DQB1*04:02
HC1008	CONTIG1	A*02:01	B*27:02	C*02:02	DRB1*11:01	DQB1*03:01
	CONTIG2	-	B*40:02	-	DRB1*13:01	DQB1*06:03
HC1009	CONTIG1	A*02:01	B*15:01	C*03:04	DRB1*04:01	DQB1*03:01
	CONTIG2	-	B*51:01	C*12:03	DRB1*11:04	DQB1*03:02
HC1010	CONTIG1	A*01:01	B*08:01	C*02:02	DRB1*13:01	DQB1*05:02
	CONTIG2	A*02:01	B*27:02	C*07:01	DRB1*16:01	DQB1*06:03
HC1011	CONTIG1	A*03:01	B*08:01	C*07:01	DRB1*01:01	DQB1*03:02
	CONTIG2	A*25:01	B*18:01	C*12:03	DRB1*04:01	DQB1*05:01
HC1012	CONTIG1	A*01:01	B*08:01	C*02:02	DRB1*03:01	DQB1*02:01
	CONTIG2	A*32:01	B*40:02	C*07:01	DRB1*11:01	DQB1*03:01
HC1013	CONTIG1	A*02:01	B*07:02	C*06:02	DRB1*13:03	DQB1*03:01
	CONTIG2	A*24:02	B*57:01	C*07:02	DRB1*15:01	DQB1*06:02
HC1014	CONTIG1	A*11:01	B*44:03	C*01:02	DRB1*04:02	DQB1*02:02
	CONTIG2	A*24:02	B*56:01	C*04:01	DRB1*07:01	DQB1*03:02
HC1015	CONTIG1	A*01:01	B*57:01	C*06:02	DRB1*07:01	DQB1*03:03
	CONTIG2	-	-	-	DRB1*07:01	-
HC1016	CONTIG1	A*01:01	B*08:01	C*07:01	DRB1*03:01	DQB1*02:01
	CONTIG2	A*02:01	B*51:01	C*15:02	DRB1*04:01	DQB1*03:02
HC1017	CONTIG1	A*01:01	B*35:02	C*04:01	DRB1*11:04	DQB1*03:01
	CONTIG2	A*68:01	B*44:02	C*05:01	DRB1*13:01	DQB1*06:03
HC1018	CONTIG1	A*02:01	B*07:05	C*15:05	DRB1*07:01	DQB1*02:02
	CONTIG2	A*29:01	B*44:03	C*16:01	DRB1*11:01	DQB1*03:01
HC1019	CONTIG1	A*24:02	B*07:02	C*03:04	DRB1*04:03	DQB1*03:02
	CONTIG2	A*31:01	B*40:01	C*07:02	DRB1*15:01	DQB1*06:02
HC1020	CONTIG1	A*03:01	B*18:01	C*03:04	DRB1*01:01	DQB1*05:01
	CONTIG2	A*25:01	B*40:01	C*12:03	DRB1*10:01	DQB1*05:01
HC1021	CONTIG1	A*01:01	B*35:08	C*03:04	DRB1*07:01	DQB1*02:02
	CONTIG2	A*02:01	B*40:01	C*04:01	DRB1*13:01	DQB1*03:01
HC1022	CONTIG1	A*03:01	B*14:02	C*05:01	DRB1*01:01	DQB1*05:01
	CONTIG2	A*33:01	B*44:02	C*08:02	-	-
HC1023	CONTIG1	A*01:01	B*35:03	C*12:02	DRB1*04:08	DQB1*03:04
	CONTIG2	A*11:01	B*52:01	C*12:03	DRB1*15:02	DQB1*06:01
HC1024	CONTIG1	A*03:01	B*15:01	C*03:03	DRB1*07:01	DQB1*02:02
	CONTIG2	A*26:01	B*50:01	C*03:04	DRB1*15:01	DQB1*06:02
HC1025	CONTIG1	A*01:01	B*08:01	C*07:01	DRB1*03:01	DQB1*02:01

	CONTIG2	A*03:01	B*18:01	C*12:03	DRB1*11:04	DQB1*03:01
HC1026	CONTIG1	A*02:01	B*18:01	C*05:01	DRB1*03:01	DQB1*02:01
	CONTIG2	A*30:02	B*41:01	C*17:01	-	-
HC1027	CONTIG1	A*02:01	B*07:02	C*02:02	DRB1*08:01	DQB1*04:02
	CONTIG2	A*32:01	B*44:05	C*07:02	DRB1*16:01	DQB1*05:02
HC1028	CONTIG1	A*01:01	B*08:01	C*06:02	DRB1*03:01	DQB1*02:01
	CONTIG2	-	B*35:02	C*07:01	DRB1*11:04	DQB1*03:01
HC1029	CONTIG1	A*32:01	B*07:02	C*02:02	DRB1*11:01	DQB1*03:01
	CONTIG2	-	B*40:02	C*07:02	-	-
HC1030	CONTIG1	A*02:01	B*08:01	C*06:02	DRB1*03:01	DQB1*02:01
	CONTIG2	-	B*57:01	C*07:01	DRB1*07:01	DQB1*03:03
HC1031	CONTIG1	A*02:01	B*40:01	C*03:04	DRB1*04:01	DQB1*03:01
	CONTIG2	A*23:01	B*50:01	C*06:02	DRB1*04:06	DQB1*04:02
HC1032	CONTIG1	A*01:01	B*08:01	C*02:02	DRB1*01:01	DQB1*02:01
	CONTIG2	A*02:01	B*27:05	C*07:01	DRB1*03:01	DQB1*05:01
HC1033	CONTIG1	A*02:01	B*41:02	C*03:02	DRB1*13:02	DQB1*03:01
	CONTIG2	A*66:01	B*58:01	C*17:03	DRB1*13:03	DQB1*06:09
HC1034	CONTIG1	A*02:01	B*35:03	C*01:02	DRB1*04:08	DQB1*03:04
	CONTIG2	A*11:01	B*51:01	C*12:03	DRB1*08:01	DQB1*04:02
HC1035	CONTIG1	A*03:02	B*38:01	C*07:18	DRB1*01:01	DQB1*03:01
	CONTIG2	A*11:01	B*58:01	C*12:03	DRB1*13:03	DQB1*05:01
HC1036	CONTIG1	A*02:01	B*15:01	C*03:04	DRB1*04:01	DQB1*03:02
	CONTIG2	A*32:01	B*57:01	C*06:02	DRB1*07:01	DQB1*03:03
HC1037	CONTIG1	A*02:01	B*27:02	C*02:02	DRB1*03:01	DQB1*02:01
	CONTIG2	A*02:06	B*27:05	C*03:03	DRB1*16:01	DQB1*05:02
HC1038	CONTIG1	A*02:01	B*13:02	C*01:02	DRB1*03:01	DQB1*02:01
	CONTIG2	A*24:02	B*56:01	C*06:02	DRB1*13:02	DQB1*06:04
HC1039	CONTIG1	A*02:01	B*27:05	C*01:02	DRB1*01:01	DQB1*04:02
	CONTIG2	A*03:01	B*51:01	-	DRB1*08:01	DQB1*05:01
HC1040	CONTIG1	A*26:01	B*08:01	C*07:02	DRB1*03:01	DQB1*02:01
	CONTIG2	A*29:02	B*44:03	C*16:01	DRB1*15:01	DQB1*06:02
HC1041	CONTIG1	A*02:01	B*07:04	C*02:02	DRB1*15:01	DQB1*05:02
	CONTIG2	-	B*51:01	C*07:02	DRB1*16:01	DQB1*06:02
HC1042	CONTIG1	A*02:01	B*40:01	C*03:04	DRB1*04:01	DQB1*02:02
	CONTIG2	A*25:01	B*44:02	C*05:01	DRB1*07:01	DQB1*03:01
HC1043	CONTIG1	A*01:01	B*07:02	C*07:01	DRB1*04:01	DQB1*03:01
	CONTIG2	A*24:02	B*18:01	C*07:02	DRB1*15:01	DQB1*06:02
HC1044	CONTIG1	A*26:01	B*38:01	C*12:03	DRB1*04:02	DQB1*02:02
	CONTIG2	A*29:02	B*44:03	C*16:01	DRB1*07:01	DQB1*03:02
HC1045	CONTIG1	A*02:01	B*44:02	C*02:02	DRB1*13:01	DQB1*05:02
	CONTIG2	A*31:01	B*51:01	C*05:01	DRB1*16:01	DQB1*06:03
HC1046	CONTIG1	A*02:01	B*35:08	C*04:01	DRB1*04:01	DQB1*03:02
	CONTIG2	-	B*37:01	C*06:02	DRB1*13:02	DQB1*06:04
HC1047	CONTIG1	A*11:01	B*27:05	C*01:02	DRB1*04:01	DQB1*03:02
	CONTIG2	A*68:01	B*38:01	C*12:03	DRB1*13:01	DQB1*06:03
HC1048	CONTIG1	A*26:01	B*27:05	C*02:02	DRB1*01:01	DQB1*03:01
	CONTIG2	-	B*35:02	C*04:01	DRB1*11:04	DQB1*05:01
HC1049	CONTIG1	A*03:01	B*39:01	C*01:02	DRB1*08:01	DQB1*04:02
	CONTIG2	A*32:01	B*56:01	C*12:03	DRB1*13:01	DQB1*06:03
HC1050	CONTIG1	A*01:01	B*37:01	C*03:04	DRB1*10:01	DQB1*05:01

	CONTIG2	A*03:01	B*40:01	C*06:02	DRB1*15:01	DQB1*06:02
HC1051	CONTIG1	A*24:02	B*27:05	C*02:02	DRB1*01:01	DQB1*03:02
	CONTIG2	A*25:01	B*27:05	-	DRB1*04:01	DQB1*05:01
HC1052	CONTIG1	A*01:01	B*18:01	C*04:01	DRB1*01:01	DQB1*05:01
	CONTIG2	A*24:02	B*35:08	C*12:03	DRB1*15:01	DQB1*06:02
HC1053	CONTIG1	A*03:01	B*15:01	C*03:04	DRB1*13:01	DQB1*06:02
	CONTIG2	A*24:02	B*57:01	C*06:02	DRB1*15:01	DQB1*06:03
HC1054	CONTIG1	A*02:01	B*14:02	C*02:02	DRB1*03:01	DQB1*02:01
	CONTIG2	A*33:01	B*27:02	C*08:02	DRB1*04:04	DQB1*04:02
HC1055	CONTIG1	A*01:01	B*08:01	C*02:02	DRB1*03:01	DQB1*02:01
	CONTIG2	A*03:01	B*44:05	C*07:01	DRB1*04:04	DQB1*03:02
HC1056	CONTIG1	A*02:01	B*08:01	C*03:04	DRB1*03:01	DQB1*02:01
	CONTIG2	A*03:01	B*40:01	C*07:01	DRB1*04:03	DQB1*03:02
HC1057	CONTIG1	A*31:01	B*35:03	C*03:02	DRB1*03:01	DQB1*02:01
	CONTIG2	A*33:03	B*58:01	C*04:01	DRB1*16:01	DQB1*05:02
HC1058	CONTIG1	A*02:01	B*44:27	C*07:04	DRB1*07:01	DQB1*02:02
	CONTIG2	A*03:02	B*51:01	C*15:02	DRB1*16:01	DQB1*05:02
HC1059	CONTIG1	A*03:01	B*18:01	C*06:02	DRB1*01:01	DQB1*03:01
	CONTIG2	A*03:01	B*47:01	C*12:03	DRB1*11:04	DQB1*05:01
HC1060	CONTIG1	A*03:01	B*18:01	C*07:01	DRB1*03:01	DQB1*02:01
	CONTIG2	A*68:01	B*38:01	C*12:03	DRB1*13:01	DQB1*06:03
HC1061	CONTIG1	A*03:01	B*35:01	C*04:01	DRB1*04:01	DQB1*03:02
	CONTIG2	-	-	C*04:01	DRB1*13:02	DQB1*06:04
HC1062	CONTIG1	A*02:01	B*15:01	C*02:02	DRB1*04:01	DQB1*03:02
	CONTIG2	A*24:02	B*27:05	C*03:03	DRB1*07:01	DQB1*03:03
HC1063	CONTIG1	A*02:06	B*35:01	C*03:03	DRB1*13:01	DQB1*05:02
	CONTIG2	A*03:01	B*44:27	C*07:04	DRB1*16:01	DQB1*06:03
HC1064	CONTIG1	A*02:01	B*18:01	C*03:04	DRB1*11:01	DQB1*03:01
	CONTIG2	A*25:01	B*40:01	C*12:03	DRB1*16:01	DQB1*05:02
HC1065	CONTIG1	A*03:01	B*07:02	C*07:01	DRB1*13:01	DQB1*06:02
	CONTIG2	A*26:01	B*08:01	C*07:02	DRB1*15:01	DQB1*06:03
HC1066	CONTIG1	A*01:01	B*27:05	C*02:02	DRB1*01:01	DQB1*03:01
	CONTIG2	A*11:01	B*35:02	C*06:02	DRB1*11:04	DQB1*05:01
HC1067	CONTIG1	A*02:01	B*18:01	C*02:02	DRB1*04:03	DQB1*03:02
	CONTIG2	A*03:01	B*37:01	C*12:03	DRB1*15:01	DQB1*05:02
HC1068	CONTIG1	A*02:01	B*15:01	C*02:02	DRB1*07:01	DQB1*02:02
	CONTIG2	-	B*27:02	C*03:04	DRB1*16:01	DQB1*05:02
HC1069	CONTIG1	A*02:01	B*07:02	C*07:02	DRB1*13:02	DQB1*06:02
	CONTIG2	A*26:01	B*51:07	C*14:02	DRB1*15:01	DQB1*06:09
HC1070	CONTIG1	A*01:01	B*08:01	C*05:01	DRB1*03:01	DQB1*02:01
	CONTIG2	A*02:01	B*44:02	C*07:01	DRB1*04:01	DQB1*03:01
HC1071	CONTIG1	A*02:01	B*07:02	C*06:02	DRB1*01:01	DQB1*02:02
	CONTIG2	A*03:01	B*13:02	C*07:02	DRB1*07:01	DQB1*05:01
HC1072	CONTIG1	A*03:01	B*18:01	C*01:02	DRB1*13:01	DQB1*06:02
	CONTIG2	A*25:01	B*51:01	C*12:03	DRB1*15:01	DQB1*06:03
HC1073	CONTIG1	A*01:01	B*08:01	C*07:01	DRB1*03:01	DQB1*02:01
	CONTIG2	A*30:02	-	C*07:18	-	-
HC1074	CONTIG1	A*11:01	B*07:02	C*07:01	DRB1*01:01	DQB1*03:05
	CONTIG2	A*29:02	B*49:01	C*07:02	DRB1*04:03	DQB1*05:01
HC1075	CONTIG1	A*29:02	B*18:01	C*05:01	DRB1*03:01	DQB1*02:01

	CONTIG2	A*30:02	B*44:03	C*16:01	DRB1*10:01	DQB1*05:01
HC1076	CONTIG1	A*25:01	B*15:01	C*03:04	DRB1*04:01	DQB1*03:01
	CONTIG2	A*29:01	B*35:02	C*04:01	DRB1*11:04	DQB1*03:02
HC1077	CONTIG1	A*03:01	B*39:01	C*12:03	DRB1*11:01	DQB1*03:01
	CONTIG2	A*32:01	B*51:01	C*14:02	DRB1*16:01	DQB1*05:02
HC1078	CONTIG1	A*01:01	B*07:02	C*02:02	DRB1*11:04	DQB1*03:01
	CONTIG2	A*03:01	B*44:05	C*07:02	DRB1*15:01	DQB1*06:02
HC1079	CONTIG1	A*01:01	B*08:01	C*06:02	DRB1*03:01	DQB1*02:01
	CONTIG2	A*24:02	B*13:02	C*07:01	DRB1*07:01	DQB1*02:02
HC1080	CONTIG1	A*02:01	B*07:02	C*05:01	DRB1*04:01	DQB1*03:01
	CONTIG2	A*31:01	B*44:02	C*07:02	DRB1*15:01	DQB1*06:02
HC1081	CONTIG1	A*24:02	B*07:02	C*07:02	DRB1*01:02	DQB1*03:01
	CONTIG2	A*33:01	B*14:02	C*08:02	DRB1*11:04	DQB1*05:01
HC1082	CONTIG1	A*03:01	B*18:01	C*01:02	DRB1*01:01	DQB1*03:03
	CONTIG2	A*11:01	B*56:01	C*07:01	DRB1*09:01	DQB1*05:01
HC1083	CONTIG1	A*01:01	B*07:02	C*07:01	DRB1*03:01	DQB1*02:01
	CONTIG2	A*03:01	B*08:01	C*07:02	DRB1*11:03	DQB1*03:01
HC1084	CONTIG1	A*01:01	B*08:01	C*07:01	DRB1*03:01	DQB1*02:01
	CONTIG2	A*02:01	B*18:01	C*12:03	DRB1*04:01	DQB1*03:01
HC1085	CONTIG1	A*03:02	B*08:01	C*07:01	DRB1*03:01	DQB1*02:01
	CONTIG2	A*68:01	B*44:02	C*07:04	DRB1*11:01	DQB1*03:01
HC1086	CONTIG1	A*03:01	B*15:01	C*04:01	DRB1*08:01	DQB1*03:01
	CONTIG2	-	B*35:01	-	DRB1*11:01	DQB1*04:02
HC1087	CONTIG1	A*02:01	B*13:02	C*06:02	DRB1*13:01	DQB1*06:03
	CONTIG2	A*30:01	B*38:01	C*12:03	DRB1*13:02	DQB1*06:04
HC1088	CONTIG1	A*03:01	B*35:01	C*02:02	DRB1*01:01	DQB1*05:01
	CONTIG2	A*29:02	B*39:01	C*04:01	DRB1*15:01	DQB1*06:02
HC1089	CONTIG1	A*01:01	B*44:03	C*04:01	DRB1*07:01	DQB1*03:03
	CONTIG2	A*23:01	B*57:01	C*06:02	DRB1*13:01	DQB1*06:03
HC1090	CONTIG1	A*01:01	B*37:01	C*06:02	DRB1*04:02	DQB1*03:02
	CONTIG2	A*26:01	B*38:01	C*12:03	DRB1*13:02	DQB1*06:04
HC1091	CONTIG1	A*01:01	B*08:01	C*06:02	DRB1*03:01	DQB1*02:01
	CONTIG2	-	B*27:02	C*07:01	DRB1*11:01	DQB1*03:01
HC1092	CONTIG1	A*01:01	B*08:01	C*07:01	DRB1*03:01	DQB1*02:01
	CONTIG2	A*68:01	B*35:03	C*12:03	DRB1*04:08	DQB1*03:04
HC1093	CONTIG1	A*03:01	B*27:02	C*02:02	DRB1*01:01	DQB1*05:01
	CONTIG2	A*24:02	B*35:03	C*04:01	DRB1*13:02	DQB1*06:04
HC1094	CONTIG1	A*01:01	B*49:01	C*03:03	DRB1*11:01	DQB1*03:01
	CONTIG2	A*02:01	B*55:01	C*07:01	DRB1*16:02	DQB1*05:02
HC1095	CONTIG1	A*03:01	B*07:02	C*01:02	DRB1*07:01	DQB1*03:03
	CONTIG2	-	B*51:01	C*07:02	DRB1*15:01	DQB1*06:02
HC1096	CONTIG1	A*11:01	B*38:01	C*05:01	DRB1*04:01	DQB1*03:01
	CONTIG2	A*26:01	B*44:02	C*12:03	DRB1*13:01	DQB1*06:03
HC1097	CONTIG1	A*11:01	B*08:01	C*07:01	DRB1*03:01	DQB1*02:01
	CONTIG2	A*31:01	B*18:01	-	DRB1*15:01	DQB1*06:02
HC1098	CONTIG1	A*02:01	B*15:01	C*03:03	DRB1*11:04	DQB1*03:01
	CONTIG2	A*25:01	B*35:08	C*04:01	DRB1*13:01	DQB1*06:03
HC1099	CONTIG1	A*02:01	B*35:01	C*04:01	DRB1*04:08	DQB1*03:04
	CONTIG2	A*24:02	B*39:06	C*07:02	DRB1*08:01	DQB1*04:02
HC1100	CONTIG1	A*01:01	B*08:01	C*06:02	DRB1*03:01	DQB1*02:01

	CONTIG2	-	B*37:01	C*07:01	DRB1*16:01	DQB1*05:02
HC1101	CONTIG1	A*01:01	B*08:01	C*02:02	DRB1*03:01	DQB1*02:01
	CONTIG2	A*11:01	B*40:02	C*07:01	DRB1*11:01	DQB1*03:01
HC1102	CONTIG1	A*01:01	B*40:02	C*02:02	DRB1*08:01	DQB1*03:01
	CONTIG2	A*02:01	B*51:01	C*14:02	DRB1*11:01	DQB1*04:02
HC1103	CONTIG1	A*02:01	B*07:02	C*06:02	DRB1*07:01	DQB1*02:02
	CONTIG2	A*25:01	B*13:02	C*07:02	DRB1*15:01	DQB1*06:02
HC1104	CONTIG1	A*01:01	B*08:01	C*04:01	DRB1*03:01	DQB1*02:01
	CONTIG2	A*03:01	B*35:03	C*07:01	DRB1*04:03	DQB1*03:02
HC1105	CONTIG1	A*02:01	B*15:01	C*04:01	DRB1*01:01	DQB1*03:03
	CONTIG2	A*11:01	B*35:01	-	DRB1*07:01	DQB1*05:01
HC1106	CONTIG1	A*24:02	B*35:03	C*03:04	DRB1*11:01	DQB1*03:01
	CONTIG2	A*32:01	B*40:02	C*12:03	DRB1*15:03	DQB1*06:03
HC1107	CONTIG1	A*25:01	B*18:01	C*12:03	DRB1*15:01	DQB1*05:02
	CONTIG2	-	-	-	DRB1*16:01	DQB1*06:02
HC1108	CONTIG1	A*25:01	B*18:01	C*12:03	DRB1*11:01	DQB1*03:01
	CONTIG2	A*68:01	B*51:01	C*15:02	DRB1*14:54	DQB1*05:03
HC1109	CONTIG1	A*02:01	B*07:02	C*04:01	DRB1*11:01	DQB1*03:01
	CONTIG2	A*03:01	B*35:03	C*07:02	DRB1*15:01	DQB1*06:02
HC1110	CONTIG1	A*02:01	B*44:02	C*05:01	DRB1*04:04	DQB1*02:02
	CONTIG2	A*29:02	B*44:03	C*16:01	DRB1*07:01	DQB1*03:02
HC1111	CONTIG1	A*24:02	B*15:01	C*03:04	DRB1*04:01	DQB1*03:02
	CONTIG2	A*25:01	B*18:01	C*12:03	DRB1*15:01	DQB1*05:01
HC1112	CONTIG1	A*01:01	B*07:02	C*07:01	DRB1*03:01	DQB1*02:01
	CONTIG2	A*03:01	B*08:01	C*07:02	DRB1*15:01	DQB1*06:02
HC1113	CONTIG1	A*11:01	B*18:03	C*05:01	DRB1*11:01	DQB1*03:01
	CONTIG2	A*32:01	B*44:02	C*07:01	DRB1*12:01	-
HC1114	CONTIG1	A*01:01	B*27:02	C*02:02	DRB1*11:01	DQB1*03:01
	CONTIG2	A*24:02	B*38:01	C*06:02	DRB1*13:05	-
HC1115	CONTIG1	A*01:01	B*08:01	C*07:01	DRB1*01:01	DQB1*02:01
	CONTIG2	A*26:01	B*49:01	-	DRB1*03:01	DQB1*05:04
HC1116	CONTIG1	A*02:01	B*13:02	C*01:02	DRB1*01:01	DQB1*02:02
	CONTIG2	A*25:01	B*27:05	C*06:02	DRB1*07:01	DQB1*05:01
HC1117	CONTIG1	A*01:01	B*08:01	C*06:02	DRB1*03:01	DQB1*02:01
	CONTIG2	A*29:02	B*45:01	C*07:01	DRB1*04:01	DQB1*03:01
HC1118	CONTIG1	A*01:01	B*08:01	C*03:03	DRB1*03:01	DQB1*02:01
	CONTIG2	A*02:01	B*15:01	C*07:01	DRB1*13:01	DQB1*06:03
HC1119	CONTIG1	A*01:02	B*27:05	C*01:02	DRB1*01:01	DQB1*05:01
	CONTIG2	A*26:01	B*38:01	C*12:03	DRB1*13:01	DQB1*06:03
HC1120	CONTIG1	A*01:01	B*08:01	C*07:01	DRB1*01:01	DQB1*05:01
	CONTIG2	A*26:01	B*38:01	C*12:03	DRB1*13:01	DQB1*06:03
HC1121	CONTIG1	A*03:01	B*44:03	C*04:01	DRB1*07:01	DQB1*02:02
	CONTIG2	A*24:02	B*49:01	C*07:01	DRB1*11:01	DQB1*03:01
HC1122	CONTIG1	A*03:02	B*08:01	C*07:02	DRB1*01:01	DQB1*05:01
	CONTIG2	A*11:01	B*52:01	C*12:02	DRB1*15:01	DQB1*06:02
HC1123	CONTIG1	A*23:01	B*08:01	C*04:01	DRB1*01:01	DQB1*03:01
	CONTIG2	A*31:01	B*44:03	C*07:01	DRB1*11:01	DQB1*05:01
HC1124	CONTIG1	A*02:01	B*15:01	C*03:03	DRB1*11:03	DQB1*03:01
	CONTIG2	A*03:01	-	-	DRB1*15:02	DQB1*06:01
HC1125	CONTIG1	A*02:01	B*39:01	C*03:04	DRB1*07:01	DQB1*02:02

	CONTIG2	A*24:02	B*40:01	C*12:03	DRB1*13:02	DQB1*06:04
HC1126	CONTIG1	A*25:01	B*35:03	C*04:01	DRB1*11:04	DQB1*03:01
	CONTIG2	A*26:01	B*39:01	C*12:03	DRB1*12:01	-
HC1127	CONTIG1	A*01:01	B*18:01	C*01:02	DRB1*12:01	DQB1*03:01
	CONTIG2	A*02:01	B*51:01	C*16:02	DRB1*14:54	DQB1*05:03
HC1128	CONTIG1	A*24:02	B*27:05	C*01:02	DRB1*07:01	DQB1*03:01
	CONTIG2	A*26:01	B*40:02	C*02:02	DRB1*11:01	DQB1*03:03
HC1129	CONTIG1	A*01:01	B*08:01	C*06:02	DRB1*03:01	DQB1*02:01
	CONTIG2	A*29:02	B*45:01	C*07:01	DRB1*04:01	DQB1*03:01
HC1130	CONTIG1	A*26:01	B*07:02	C*05:01	DRB1*13:01	DQB1*06:02
	CONTIG2	A*32:01	B*44:02	C*07:02	DRB1*15:01	DQB1*06:03
HC1131	CONTIG1	A*01:01	B*07:02	C*07:02	DRB1*15:01	DQB1*06:02
	CONTIG2	A*03:01	-	-	-	-
HC1132	CONTIG1	A*02:01	B*44:02	C*03:03	DRB1*01:01	DQB1*03:01
	CONTIG2	A*11:01	B*52:01	C*12:02	DRB1*12:01	DQB1*05:01
HC1133	CONTIG1	A*23:01	B*40:02	C*02:02	DRB1*13:01	DQB1*05:02
	CONTIG2	A*31:01	B*57:01	C*06:02	DRB1*16:01	DQB1*06:03
HC1134	CONTIG1	A*02:01	B*07:02	C*07:02	DRB1*09:20	DQB1*03:03
	CONTIG2	A*26:01	-	-	DRB1*15:01	DQB1*06:02
HC1135	CONTIG1	A*24:02	B*27:05	C*02:02	DRB1*13:01	DQB1*06:03
	CONTIG2	A*33:03	B*58:01	C*03:02	DRB1*13:02	DQB1*06:09
HC1136	CONTIG1	A*24:02	B*15:01	C*03:03	DRB1*13:01	DQB1*05:03
	CONTIG2	A*32:01	B*18:01	C*07:01	DRB1*14:54	DQB1*06:03
HC1137	CONTIG1	A*11:01	B*38:01	C*12:03	DRB1*04:04	DQB1*03:02
	CONTIG2	A*26:01	B*51:01	C*15:02	DRB1*13:01	DQB1*06:03
HC1138	CONTIG1	A*03:01	B*07:02	C*05:01	DRB1*04:01	DQB1*03:01
	CONTIG2	A*24:02	B*44:02	C*07:02	DRB1*15:01	DQB1*06:02
HC1139	CONTIG1	A*01:01	B*08:01	C*07:01	DRB1*03:01	DQB1*02:01
	CONTIG2	A*02:01	B*39:01	C*12:03	DRB1*16:01	DQB1*05:02
HC1140	CONTIG1	A*02:01	B*07:02	C*03:04	DRB1*04:01	DQB1*03:02
	CONTIG2	A*26:01	B*15:01	C*07:02	DRB1*15:01	DQB1*06:02
HC1141	CONTIG1	A*01:01	B*08:01	C*03:03	DRB1*03:01	DQB1*02:01
	CONTIG2	A*25:01	B*35:01	C*07:01	DRB1*08:01	DQB1*04:02
HC1142	CONTIG1	A*03:01	B*07:02	C*04:01	DRB1*04:01	DQB1*03:01
	CONTIG2	A*26:01	B*35:01	C*07:02	DRB1*11:04	DQB1*03:02
HC1143	CONTIG1	A*02:01	B*41:02	C*07:01	DRB1*07:01	DQB1*02:02
	CONTIG2	A*02:05	B*49:01	C*17:03	DRB1*13:03	DQB1*03:01
HC1144	CONTIG1	A*02:01	B*38:01	C*03:03	DRB1*11:03	DQB1*03:01
	CONTIG2	A*26:01	B*44:02	C*12:03	DRB1*13:01	DQB1*06:03
HC1145	CONTIG1	A*11:01	B*35:02	C*04:01	DRB1*01:01	DQB1*05:01
	CONTIG2	A*24:02	B*44:02	C*05:01	DRB1*13:02	DQB1*06:04
HC1146	CONTIG1	A*01:01	B*15:01	C*04:01	DRB1*07:01	DQB1*03:01
	CONTIG2	A*02:01	B*35:02	C*06:02	DRB1*11:03	DQB1*03:03
HC1147	CONTIG1	A*02:01	B*40:01	C*03:04	DRB1*08:01	DQB1*04:02
	CONTIG2	A*31:01	B*51:01	C*14:02	DRB1*13:01	DQB1*06:03
HC1148	CONTIG1	A*02:01	B*07:02	C*06:02	DRB1*07:01	DQB1*02:02
	CONTIG2	A*32:01	B*13:02	C*07:02	DRB1*15:01	DQB1*06:02
HC1149	CONTIG1	A*03:01	B*07:02	C*07:02	DRB1*15:01	DQB1*06:02
	CONTIG2	A*11:01	-	-	-	-
HC1150	CONTIG1	A*02:01	B*35:03	C*07:04	DRB1*04:01	DQB1*03:01

	CONTIG2	A*03:01	B*44:27	C*12:03	DRB1*04:08	DQB1*03:04
HC1151	CONTIG1	A*02:01	B*15:01	C*02:02	DRB1*04:01	DQB1*03:02
	CONTIG2	-	B*51:01	C*03:04	DRB1*13:02	DQB1*06:04
HC1152	CONTIG1	A*25:01	B*18:01	C*02:02	DRB1*01:01	DQB1*05:01
	CONTIG2	A*32:01	B*40:02	C*12:03	DRB1*13:01	DQB1*06:03
HC1153	CONTIG1	A*03:01	B*27:05	C*02:02	DRB1*04:07	DQB1*03:01
	CONTIG2	A*24:02	B*35:01	C*04:01	DRB1*11:01	-
HC1154	CONTIG1	A*01:01	B*07:02	C*01:02	DRB1*10:01	DQB1*05:01
	CONTIG2	A*11:01	B*55:01	C*07:02	DRB1*15:01	DQB1*06:02
HC1155	CONTIG1	A*02:01	B*07:02	C*03:04	DRB1*04:01	DQB1*03:02
	CONTIG2	-	B*27:05	C*07:02	DRB1*15:01	DQB1*06:02
HC1156	CONTIG1	A*31:01	B*14:02	C*01:02	DRB1*01:02	DQB1*05:01
	CONTIG2	A*33:01	B*51:01	C*08:02	DRB1*16:01	DQB1*05:02
HC1157	CONTIG1	A*02:01	B*07:02	C*04:01	DRB1*09:01	DQB1*03:01
	CONTIG2	A*24:02	B*35:03	C*07:02	DRB1*11:01	DQB1*03:03
HC1158	CONTIG1	A*03:01	B*07:02	C*07:01	DRB1*03:01	DQB1*02:01
	CONTIG2	A*32:01	B*08:01	C*07:02	DRB1*04:07	DQB1*03:01
HC1159	CONTIG1	A*02:01	B*18:01	C*02:02	DRB1*16:01	DQB1*05:02
	CONTIG2	A*24:02	B*27:02	C*12:03	-	-
HC1160	CONTIG1	A*03:01	B*35:01	C*04:01	DRB1*07:01	DQB1*02:02
	CONTIG2	A*11:01	B*47:02	C*06:02	DRB1*14:54	DQB1*05:03
HC1161	CONTIG1	A*02:06	B*07:02	C*03:03	DRB1*01:01	DQB1*05:01
	CONTIG2	A*24:02	B*55:01	C*07:02	DRB1*15:01	DQB1*06:02
HC1162	CONTIG1	A*11:01	B*35:01	C*02:02	DRB1*11:01	DQB1*03:01
	CONTIG2	A*24:02	B*40:02	C*04:01	DRB1*14:54	DQB1*05:03
HC1163	CONTIG1	A*02:686	B*07:02	C*07:02	DRB1*04:01	DQB1*03:02
	CONTIG2	A*25:01	B*18:01	C*12:03	DRB1*15:01	DQB1*06:02
HC1164	CONTIG1	A*02:01	B*27:05	C*01:02	DRB1*01:01	DQB1*03:03
	CONTIG2	A*26:01	B*35:01	C*04:01	DRB1*07:01	DQB1*05:01
HC1165	CONTIG1	A*11:01	B*35:01	C*01:02	DRB1*01:01	DQB1*05:01
	CONTIG2	A*26:01	B*56:01	C*04:01	DRB1*13:02	DQB1*06:04
HC1166	CONTIG1	A*02:01	B*39:01	C*04:01	DRB1*07:01	DQB1*02:02
	CONTIG2	-	B*44:03	C*12:03	DRB1*13:01	DQB1*06:03
HC1167	CONTIG1	A*11:01	B*35:01	C*03:03	DRB1*01:01	DQB1*03:01
	CONTIG2	A*25:01	B*55:01	C*04:01	DRB1*11:03	DQB1*05:01
HC1168	CONTIG1	A*02:01	B*07:02	C*07:02	DRB1*01:01	DQB1*05:01
	CONTIG2	A*68:01	-	-	DRB1*15:01	DQB1*06:02
HC1169	CONTIG1	A*01:01	B*08:01	C*07:01	DRB1*13:01	DQB1*06:02
	CONTIG2	A*24:02	B*39:06	C*07:02	DRB1*15:01	DQB1*06:03
HC1170	CONTIG1	A*01:01	B*15:17	C*04:01	DRB1*13:02	DQB1*03:01
	CONTIG2	A*33:03	B*35:08	C*07:01	DRB1*13:03	DQB1*06:04
HC1171	CONTIG1	A*11:01	B*35:03	C*07:02	DRB1*04:08	DQB1*03:04
	CONTIG2	A*24:02	B*39:06	C*12:03	DRB1*08:01	DQB1*04:02
HC1172	CONTIG1	A*01:01	B*08:01	C*06:02	DRB1*03:01	DQB1*02:01
	CONTIG2	A*31:01	B*13:02	C*07:01	DRB1*15:01	DQB1*06:02
HC1173	CONTIG1	A*02:01	B*40:01	C*03:04	DRB1*03:01	DQB1*02:01
	CONTIG2	A*68:01	-	-	DRB1*15:02	DQB1*06:01
HC1174	CONTIG1	A*01:01	B*15:01	C*03:03	DRB1*11:04	DQB1*03:01
	CONTIG2	A*11:01	B*35:02	C*06:02	DRB1*13:01	DQB1*06:03
HC1175	CONTIG1	A*25:01	B*18:01	C*02:02	DRB1*01:01	DQB1*05:01

	CONTIG2	A*32:01	B*27:05	C*12:03	DRB1*15:01	DQB1*06:02
HC1176	CONTIG1	A*03:01	B*39:01	C*02:02	DRB1*11:01	DQB1*03:01
	CONTIG2	-	B*51:01	C*12:03	-	-
HC1177	CONTIG1	A*03:01	B*07:02	C*04:01	DRB1*01:01	DQB1*02:02
	CONTIG2	A*31:01	B*35:01	C*07:02	DRB1*07:01	DQB1*05:01
HC1178	CONTIG1	A*11:01	B*35:01	C*04:01	DRB1*03:01	DQB1*02:01
	CONTIG2	-	B*35:03	-	DRB1*15:01	DQB1*06:02
HC1179	CONTIG1	A*25:01	B*15:01	C*04:01	DRB1*01:01	DQB1*04:02
	CONTIG2	A*26:01	B*18:01	C*12:03	DRB1*08:01	DQB1*05:01
HC1180	CONTIG1	A*01:01	B*07:04	C*07:01	DRB1*03:01	DQB1*02:01
	CONTIG2	A*02:01	B*08:01	C*07:02	DRB1*15:01	DQB1*06:02
HC1181	CONTIG1	A*01:01	B*08:01	C*07:01	DRB1*03:01	DQB1*02:01
	CONTIG2	A*02:01	B*39:01	C*12:03	DRB1*13:01	DQB1*06:03
HC1182	CONTIG1	A*03:01	B*38:01	C*03:03	DRB1*14:54	DQB1*05:03
	CONTIG2	A*26:01	B*55:01	C*12:03	DRB1*15:01	DQB1*06:03
HC1183	CONTIG1	A*02:01	B*07:02	C*05:01	DRB1*11:01	DQB1*03:01
	CONTIG2	A*24:02	B*44:02	C*07:02	DRB1*12:01	-
HC1184	CONTIG1	A*03:01	B*44:03	C*04:01	DRB1*07:01	DQB1*02:02
	CONTIG2	A*23:01	B*44:27	C*07:04	DRB1*16:01	DQB1*05:02
HC1185	CONTIG1	A*02:01	B*13:02	C*06:02	DRB1*07:01	DQB1*02:02
	CONTIG2	-	B*18:01	C*07:01	DRB1*11:04	DQB1*03:01
HC1186	CONTIG1	A*01:01	B*44:03	C*06:02	DRB1*07:01	DQB1*02:02
	CONTIG2	A*24:02	B*57:01	C*16:01	-	DQB1*03:03
HC1187	CONTIG1	A*02:01	B*14:02	C*08:02	DRB1*01:02	DQB1*05:01
	CONTIG2	A*25:01	B*39:01	C*12:03	DRB1*16:01	DQB1*05:02
HC1188	CONTIG1	A*02:01	B*41:01	C*05:01	DRB1*07:01	DQB1*02:02
	CONTIG2	-	B*44:02	C*17:01	DRB1*11:01	DQB1*03:01
HC1189	CONTIG1	A*02:01	B*35:03	C*04:01	DRB1*04:01	DQB1*03:01
	CONTIG2	-	B*44:02	C*05:01	DRB1*04:03	DQB1*03:02
HC1190	CONTIG1	A*02:01	B*35:01	C*03:02	DRB1*13:02	DQB1*05:03
	CONTIG2	A*11:01	B*58:01	C*04:01	DRB1*14:54	DQB1*06:09
HC1191	CONTIG1	A*24:02	B*07:05	C*02:02	DRB1*01:01	DQB1*05:01
	CONTIG2	A*29:01	B*44:05	C*15:05	DRB1*10:01	-
HC1192	CONTIG1	A*02:01	B*15:01	C*04:01	DRB1*07:01	DQB1*03:03
	CONTIG2	-	B*38:01	C*12:03	DRB1*13:01	DQB1*06:03
HC1193	CONTIG1	A*01:01	B*35:01	C*04:01	DRB1*01:03	DQB1*03:01
	CONTIG2	A*32:01	B*44:02	C*05:01	DRB1*14:54	DQB1*05:03
HC1194	CONTIG1	A*02:01	B*13:02	C*02:02	DRB1*07:01	DQB1*02:02
	CONTIG2	A*03:01	B*27:02	C*06:02	DRB1*16:01	DQB1*05:02
HC1195	CONTIG1	A*03:01	B*35:01	C*04:01	DRB1*01:01	DQB1*03:02
	CONTIG2	A*68:01	B*35:03	-	DRB1*08:01	DQB1*05:01
HC1196	CONTIG1	A*02:01	B*13:02	C*02:02	DRB1*16:01	DQB1*05:02
	CONTIG2	A*24:02	B*27:02	C*06:02	-	-
HC1197	CONTIG1	A*03:01	B*07:02	C*02:02	DRB1*01:01	DQB1*03:02
	CONTIG2	A*24:02	B*40:02	C*07:02	DRB1*04:04	DQB1*05:01
HC1198	CONTIG1	A*02:01	B*27:05	C*02:02	DRB1*04:02	DQB1*03:02
	CONTIG2	A*11:01	B*44:03	C*16:02	DRB1*16:01	DQB1*05:02
HC1199	CONTIG1	A*01:01	B*35:02	C*01:02	DRB1*11:04	DQB1*03:01
	CONTIG2	A*02:01	B*51:01	C*06:02	DRB1*15:01	DQB1*06:02
HC1200	CONTIG1	A*02:06	B*15:01	C*03:03	DRB1*11:01	DQB1*03:01

	CONTIG2	A*25:01	B*35:01	-	DRB1*13:01	DQB1*06:03
HC1201	CONTIG1	A*01:01	B*35:01	C*04:01	DRB1*11:01	DQB1*03:01
	CONTIG2	A*02:01	B*39:01	C*12:03	DRB1*13:03	-
HC1202	CONTIG1	A*01:01	B*08:01	C*04:01	DRB1*13:02	DQB1*06:03
	CONTIG2	A*02:01	B*35:02	C*07:01	DRB1*15:01	DQB1*06:04
HC1203	CONTIG1	A*24:02	B*18:01	C*07:01	DRB1*11:04	DQB1*03:01
	CONTIG2	A*68:02	B*40:06	C*12:02	DRB1*12:02	-
HC1204	CONTIG1	A*24:02	B*35:02	C*04:01	DRB1*11:04	DQB1*03:01
	CONTIG2	A*31:01	B*39:01	C*12:03	DRB1*12:01	-
HC1205	CONTIG1	A*03:01	B*35:01	C*04:01	DRB1*01:01	DQB1*05:01
	CONTIG2	A*23:01	B*50:01	C*06:02	DRB1*15:01	DQB1*05:02
HC1206	CONTIG1	A*03:01	B*07:02	C*02:02	DRB1*15:01	DQB1*05:02
	CONTIG2	A*11:01	B*27:02	C*07:02	DRB1*16:01	DQB1*06:02
HC1207	CONTIG1	A*01:01	B*18:01	C*06:02	DRB1*07:01	DQB1*03:03
	CONTIG2	A*25:01	B*57:01	C*12:03	DRB1*13:01	DQB1*06:03
HC1208	CONTIG1	A*11:01	B*27:05	C*01:02	DRB1*11:01	DQB1*03:01
	CONTIG2	A*26:01	B*41:02	C*17:03	DRB1*13:03	-
HC1209	CONTIG1	A*03:01	B*44:02	C*03:03	DRB1*11:01	DQB1*03:01
	CONTIG2	A*25:01	B*55:01	C*07:04	DRB1*14:54	DQB1*05:03
HC1210	CONTIG1	A*03:01	B*07:02	C*07:02	DRB1*04:01	DQB1*03:02
	CONTIG2	A*24:02	B*52:01	C*12:02	DRB1*15:01	DQB1*06:02
HC1211	CONTIG1	A*02:01	B*44:02	C*05:01	DRB1*07:01	DQB1*03:01
	CONTIG2	A*03:01	B*57:01	C*06:02	DRB1*12:01	DQB1*03:03
HC1212	CONTIG1	A*29:02	B*40:02	C*02:02	DRB1*07:01	DQB1*02:02
	CONTIG2	A*32:01	B*44:03	C*16:01	DRB1*11:01	DQB1*03:01
HC1213	CONTIG1	A*01:01	B*08:01	C*06:02	DRB1*03:01	DQB1*02:01
	CONTIG2	A*29:02	B*45:01	C*07:01	DRB1*09:01	DQB1*02:02
HC1214	CONTIG1	A*01:01	B*08:01	C*05:01	DRB1*03:01	DQB1*02:01
	CONTIG2	A*32:01	B*44:02	C*07:01	DRB1*12:01	DQB1*03:01
HC1215	CONTIG1	A*03:01	B*07:02	C*06:02	DRB1*14:54	DQB1*05:03
	CONTIG2	A*30:01	B*13:02	C*07:02	DRB1*15:01	DQB1*06:02
HC1216	CONTIG1	A*02:01	B*13:02	C*04:01	DRB1*04:02	DQB1*02:02
	CONTIG2	A*24:02	B*35:01	C*06:02	DRB1*07:01	DQB1*03:02
HC1217	CONTIG1	A*01:01	B*08:01	C*03:03	DRB1*07:01	DQB1*02:02
	CONTIG2	A*26:01	B*55:01	C*07:01	DRB1*11:03	DQB1*03:01
HC1218	CONTIG1	A*02:01	B*13:02	C*05:01	DRB1*03:01	DQB1*02:01
	CONTIG2	A*30:01	B*44:02	C*06:02	DRB1*12:01	DQB1*03:01
HC1219	CONTIG1	A*01:01	B*08:01	C*07:01	DRB1*01:01	DQB1*02:01
	CONTIG2	A*25:01	B*18:01	C*12:03	DRB1*03:01	DQB1*05:01
HC1220	CONTIG1	A*03:01	B*18:01	C*07:01	DRB1*13:01	DQB1*05:03
	CONTIG2	A*11:01	B*38:01	C*12:03	DRB1*14:54	DQB1*06:03
HC1221	CONTIG1	A*01:01	B*18:01	C*07:01	DRB1*09:01	DQB1*03:01
	CONTIG2	A*02:01	B*48:01	C*08:03	DRB1*11:01	DQB1*03:03
HC1222	CONTIG1	A*01:01	B*08:01	C*04:01	DRB1*03:01	DQB1*02:01
	CONTIG2	A*23:01	B*44:03	C*07:01	DRB1*07:01	DQB1*02:02
HC1223	CONTIG1	A*25:01	B*18:01	C*03:04	DRB1*01:01	DQB1*03:03
	CONTIG2	A*68:01	B*40:01	C*12:03	DRB1*07:01	DQB1*05:01
HC1224	CONTIG1	A*02:01	B*07:02	C*05:01	DRB1*13:01	DQB1*06:02
	CONTIG2	A*03:01	B*44:02	C*07:02	DRB1*15:01	DQB1*06:03
HC1225	CONTIG1	A*03:01	B*35:01	C*06:02	DRB1*01:01	DQB1*02:02

	CONTIG2	A*11:01	B*50:01	C*15:02	DRB1*07:01	DQB1*05:01
HC1226	CONTIG1	A*01:01	B*40:02	C*02:02	DRB1*15:02	DQB1*05:02
	CONTIG2	A*11:01	B*52:01	C*12:02	DRB1*16:02	DQB1*06:01
HC1227	CONTIG1	A*01:01	B*07:02	C*07:01	DRB1*03:01	DQB1*02:01
	CONTIG2	A*03:01	B*08:01	C*07:02	DRB1*15:01	DQB1*06:02
HC1228	CONTIG1	A*11:01	B*15:01	C*03:03	DRB1*13:01	DQB1*06:03
	CONTIG2	A*24:02	B*35:01	C*04:01	DRB1*13:02	DQB1*06:04
HC1229	CONTIG1	A*02:01	B*39:01	C*05:01	DRB1*04:01	DQB1*03:01
	CONTIG2	-	B*44:02	C*07:02	DRB1*07:01	DQB1*03:03
HC1230	CONTIG1	A*02:01	B*18:01	C*04:01	DRB1*01:01	DQB1*03:01
	CONTIG2	A*23:01	B*44:03	C*07:01	DRB1*11:01	DQB1*05:01
HC1231	CONTIG1	A*01:01	B*07:02	C*06:02	DRB1*07:01	DQB1*03:03
	CONTIG2	A*03:01	B*57:01	C*07:02	DRB1*15:01	DQB1*06:02
HC1232	CONTIG1	A*02:01	B*07:02	C*07:02	DRB1*08:04	DQB1*04:02
	CONTIG2	A*26:01	-	-	DRB1*15:01	DQB1*06:02
HC1233	CONTIG1	A*25:01	B*07:02	C*07:02	DRB1*08:01	DQB1*04:02
	CONTIG2	A*26:01	B*18:01	C*12:03	DRB1*13:01	DQB1*06:03
HC1234	CONTIG1	A*02:01	B*08:01	C*05:01	DRB1*03:01	DQB1*02:01
	CONTIG2	A*11:01	B*44:02	C*07:01	DRB1*04:02	DQB1*03:02
HC1235	CONTIG1	A*01:01	B*08:01	C*03:04	DRB1*07:01	DQB1*02:02
	CONTIG2	A*68:01	B*40:01	C*07:01	DRB1*13:03	DQB1*03:01
HC1236	CONTIG1	A*01:01	B*35:02	C*04:01	DRB1*07:01	DQB1*02:02
	CONTIG2	A*26:01	B*49:01	C*07:01	DRB1*11:04	DQB1*03:01
HC1237	CONTIG1	A*02:01	B*27:02	C*02:02	DRB1*04:05	DQB1*02:02
	CONTIG2	-	B*41:01	C*17:38	DRB1*16:01	DQB1*05:02
HC1238	CONTIG1	A*02:01	B*35:01	C*04:01	DRB1*04:01	DQB1*02:02
	CONTIG2	A*03:01	B*44:02	C*05:01	DRB1*07:01	DQB1*03:01
HC1239	CONTIG1	A*26:01	B*27:05	C*02:02	DRB1*13:01	DQB1*05:02
	CONTIG2	A*31:01	B*38:01	C*12:03	DRB1*16:01	DQB1*06:03
HC1240	CONTIG1	A*02:01	B*27:05	C*01:02	DRB1*01:01	DQB1*04:02
	CONTIG2	-	B*35:01	C*04:01	DRB1*08:01	DQB1*05:01
HC1241	CONTIG1	A*01:01	B*38:01	C*08:03	DRB1*04:02	DQB1*03:01
	CONTIG2	A*03:01	B*48:01	C*12:03	DRB1*12:01	DQB1*03:02
HC1242	CONTIG1	A*01:01	B*15:07	C*03:03	DRB1*04:04	DQB1*03:02
	CONTIG2	A*24:02	B*52:01	C*12:02	DRB1*15:02	DQB1*06:01
HC1243	CONTIG1	A*02:01	B*18:01	C*03:04	DRB1*11:01	DQB1*03:01
	CONTIG2	A*25:01	B*40:01	C*12:03	DRB1*12:01	-
HC1244	CONTIG1	A*01:01	B*08:01	C*07:01	DRB1*03:01	DQB1*02:01
	CONTIG2	-	-	-	-	-
HC1245	CONTIG1	A*01:01	B*08:01	C*01:02	DRB1*01:01	DQB1*02:01
	CONTIG2	A*02:01	B*56:01	C*07:01	DRB1*03:01	DQB1*05:01
HC1246	CONTIG1	A*01:01	B*08:01	C*07:01	DRB1*01:01	DQB1*02:01
	CONTIG2	A*11:01	B*52:01	C*12:02	DRB1*03:01	DQB1*05:01
HC1247	CONTIG1	A*01:01	B*08:01	C*02:02	DRB1*01:03	DQB1*02:01
	CONTIG2	A*11:01	B*27:05	C*07:01	DRB1*03:01	DQB1*03:01
HC1248	CONTIG1	A*02:01	B*07:02	C*03:04	DRB1*04:01	DQB1*03:02
	CONTIG2	-	B*15:01	C*07:02	DRB1*04:03	-
HC1249	CONTIG1	A*25:01	B*38:01	C*06:02	DRB1*07:01	DQB1*03:03
	CONTIG2	A*26:01	B*57:01	C*12:03	DRB1*08:01	DQB1*04:02
HC1250	CONTIG1	A*02:01	B*07:02	C*05:01	DRB1*01:01	DQB1*04:02

	CONTIG2	A*25:01	B*44:02	C*07:02	DRB1*08:01	DQB1*05:01
HC1251	CONTIG1	A*02:01	B*51:01	C*04:01	DRB1*07:01	DQB1*03:03
	CONTIG2	A*24:02	B*51:05	C*05:01	DRB1*14:04	DQB1*05:03
HC1252	CONTIG1	A*01:01	B*08:01	C*03:03	DRB1*01:01	DQB1*02:01
	CONTIG2	A*25:01	B*15:01	C*07:01	DRB1*03:01	DQB1*05:01
HC1253	CONTIG1	A*03:01	B*07:02	C*07:02	DRB1*15:01	DQB1*06:02
	CONTIG2	A*26:01	-	-	-	-
HC1254	CONTIG1	A*02:01	B*15:01	C*03:04	DRB1*04:01	DQB1*03:02
	CONTIG2	A*03:01	B*35:03	C*04:01	DRB1*15:01	DQB1*06:02
HC1255	CONTIG1	A*02:01	B*35:08	C*04:01	DRB1*04:01	DQB1*03:01
	CONTIG2	A*24:02	B*57:01	C*06:02	DRB1*11:01	DQB1*03:02
HC1256	CONTIG1	A*02:01	B*13:02	C*03:03	DRB1*07:01	DQB1*02:02
	CONTIG2	-	B*35:01	C*06:02	DRB1*08:01	DQB1*04:02
HC1257	CONTIG1	A*02:01	B*41:01	C*04:01	DRB1*07:01	DQB1*02:02
	CONTIG2	A*23:01	B*44:03	C*17:01	DRB1*11:04	DQB1*03:01
HC1258	CONTIG1	A*32:01	B*14:02	C*04:01	DRB1*01:02	DQB1*03:01
	CONTIG2	A*33:01	B*35:01	C*08:02	DRB1*11:03	DQB1*05:01
HC1259	CONTIG1	A*11:01	B*15:01	C*01:02	DRB1*11:01	DQB1*03:01
	CONTIG2	A*68:01	B*27:05	C*03:03	DRB1*13:01	DQB1*06:03
HC1260	CONTIG1	A*02:01	B*13:02	C*06:02	DRB1*07:01	DQB1*02:02
	CONTIG2	A*30:01	-	-	DRB1*11:03	DQB1*03:01
HC1261	CONTIG1	A*03:01	B*07:02	C*03:04	DRB1*01:01	DQB1*02:01
	CONTIG2	A*68:01	B*40:01	C*07:02	DRB1*03:01	DQB1*05:01
HC1262	CONTIG1	A*24:02	B*27:05	C*02:02	DRB1*13:01	DQB1*06:03
	CONTIG2	A*25:01	B*40:01	C*03:04	-	-
HC1263	CONTIG1	A*02:01	B*07:02	C*07:02	DRB1*08:01	DQB1*03:01
	CONTIG2	A*03:01	B*51:01	C*15:02	DRB1*12:01	DQB1*04:02
HC1264	CONTIG1	A*01:01	B*35:01	C*04:01	DRB1*01:03	DQB1*03:01
	CONTIG2	A*32:01	B*44:02	C*05:01	DRB1*14:54	DQB1*05:03
HC1265	CONTIG1	A*03:02	B*18:01	C*03:04	DRB1*03:01	DQB1*02:01
	CONTIG2	A*68:01	B*40:01	C*15:02	DRB1*11:01	DQB1*03:01
HC1266	CONTIG1	A*01:01	B*27:05	C*02:02	DRB1*08:03	DQB1*03:01
	CONTIG2	A*02:01	B*51:01	C*16:02	DRB1*11:01	-
HC1267	CONTIG1	A*03:01	B*15:01	C*03:04	DRB1*04:01	DQB1*03:02
	CONTIG2	A*25:01	B*18:01	C*12:03	DRB1*15:01	DQB1*06:02
HC1268	CONTIG1	A*01:01	B*08:01	C*07:01	DRB1*04:01	DQB1*03:01
	CONTIG2	A*11:01	B*52:01	C*12:02	DRB1*04:04	DQB1*03:02
HC1269	CONTIG1	A*02:01	B*40:01	C*02:02	DRB1*11:01	DQB1*03:01
	CONTIG2	A*11:01	B*40:02	C*03:04	DRB1*13:01	DQB1*06:03
HC1270	CONTIG1	A*26:01	B*08:01	C*03:03	DRB1*03:01	DQB1*02:01
	CONTIG2	A*30:04	B*55:01	C*07:01	DRB1*14:54	DQB1*05:03
HC1271	CONTIG1	A*02:01	B*07:02	C*02:02	DRB1*11:01	DQB1*03:01
	CONTIG2	A*32:01	B*40:02	C*07:02	DRB1*15:01	DQB1*06:02
HC1272	CONTIG1	A*01:01	B*08:01	C*07:01	DRB1*03:01	DQB1*02:01
	CONTIG2	-	-	-	-	-
HC1273	CONTIG1	A*29:01	B*40:01	C*03:04	DRB1*01:01	DQB1*03:03
	CONTIG2	A*68:01	B*44:27	C*07:04	DRB1*07:01	DQB1*05:01
HC1274	CONTIG1	A*01:01	B*08:01	C*07:01	DRB1*03:01	DQB1*02:01
	CONTIG2	A*25:01	B*18:01	C*12:03	DRB1*15:01	DQB1*06:02
HC1275	CONTIG1	A*24:02	B*18:01	C*07:01	DRB1*11:01	DQB1*03:01

	CONTIG2	A*68:01	B*44:02	C*07:68	-	-
HC1276	CONTIG1	A*11:01	B*35:01	C*02:02	DRB1*01:01	DQB1*03:02
	CONTIG2	A*26:01	B*40:02	C*04:01	DRB1*04:04	DQB1*05:01
HC1277	CONTIG1	A*11:01	B*08:01	C*04:01	DRB1*03:01	DQB1*02:01
	CONTIG2	A*23:01	B*44:03	C*07:01	DRB1*09:01	DQB1*03:03
HC1278	CONTIG1	A*01:01	B*08:01	C*06:02	DRB1*03:01	DQB1*02:01
	CONTIG2	A*03:01	B*47:01	C*07:01	DRB1*07:01	DQB1*02:02
HC1279	CONTIG1	A*01:01	B*08:01	C*06:02	DRB1*07:01	DQB1*02:02
	CONTIG2	A*02:01	B*13:02	C*07:01	DRB1*13:01	DQB1*06:03
HC1280	CONTIG1	A*01:01	B*08:01	C*04:01	DRB1*03:01	DQB1*02:01
	CONTIG2	A*02:01	B*35:02	C*07:01	DRB1*08:04	DQB1*03:01
HC1281	CONTIG1	A*01:01	B*18:01	C*05:01	DRB1*04:01	DQB1*03:01
	CONTIG2	A*25:01	B*44:02	C*12:03	DRB1*15:01	DQB1*06:02
HC1282	CONTIG1	A*03:01	B*07:02	C*06:02	DRB1*07:01	DQB1*02:02
	CONTIG2	A*23:01	B*13:02	C*07:02	DRB1*11:04	DQB1*03:01
HC1283	CONTIG1	A*26:01	B*07:02	C*02:02	DRB1*08:01	DQB1*04:02
	CONTIG2	A*31:01	B*40:02	C*07:01	DRB1*14:01	DQB1*05:03
HC1284	CONTIG1	A*01:01	B*44:03	C*04:01	DRB1*03:01	DQB1*02:01
	CONTIG2	-	B*57:01	C*06:02	DRB1*07:01	DQB1*02:02
HC1285	CONTIG1	A*01:01	B*08:01	C*03:03	DRB1*03:01	DQB1*02:01
	CONTIG2	A*24:02	B*15:01	C*07:01	DRB1*11:03	DQB1*03:01
HC1286	CONTIG1	A*01:01	B*44:02	C*05:01	DRB1*14:54	DQB1*05:02
	CONTIG2	A*03:01	B*44:27	C*07:04	DRB1*16:01	DQB1*05:03
HC1287	CONTIG1	A*02:01	B*13:02	C*03:04	DRB1*07:01	DQB1*02:02
	CONTIG2	-	B*40:01	C*06:02	DRB1*13:02	DQB1*06:04
HC1288	CONTIG1	A*02:01	B*07:02	C*02:02	DRB1*12:01	DQB1*03:01
	CONTIG2	A*68:01	B*27:05	C*07:02	DRB1*15:01	DQB1*06:02
HC1289	CONTIG1	A*02:01	B*07:02	C*02:02	DRB1*04:04	DQB1*03:02
	CONTIG2	A*03:01	B*27:05	C*07:02	DRB1*15:01	DQB1*06:02
HC1290	CONTIG1	A*02:01	B*15:01	C*03:03	DRB1*13:01	DQB1*06:03
	CONTIG2	A*11:01	B*40:01	C*03:04	DRB1*13:02	DQB1*06:04
HC1291	CONTIG1	A*01:01	B*08:01	C*03:04	DRB1*03:01	DQB1*02:01
	CONTIG2	A*02:01	B*15:01	C*07:01	DRB1*11:04	DQB1*03:01
HC1292	CONTIG1	A*02:01	B*51:01	C*02:02	DRB1*11:01	DQB1*03:01
	CONTIG2	A*26:01	-	C*14:02	DRB1*15:01	DQB1*06:02
HC1293	CONTIG1	A*01:01	B*39:01	C*06:02	DRB1*07:01	DQB1*02:02
	CONTIG2	A*02:05	B*50:01	-	DRB1*11:01	DQB1*03:01
HC1294	CONTIG1	A*02:01	B*35:03	C*02:29	DRB1*08:01	DQB1*03:01
	CONTIG2	A*68:01	B*51:01	C*04:01	DRB1*11:01	DQB1*04:02
HC1295	CONTIG1	A*02:01	B*07:02	C*05:01	DRB1*04:01	DQB1*03:01
	CONTIG2	A*31:01	B*44:02	C*07:02	DRB1*15:01	DQB1*06:02
HC1296	CONTIG1	A*02:01	B*07:02	C*07:02	DRB1*01:01	DQB1*05:02
	CONTIG2	-	-	-	DRB1*15:01	DQB1*05:160
HC1297	CONTIG1	A*02:01	B*27:05	C*03:04	DRB1*04:01	DQB1*03:02
	CONTIG2	-	B*38:01	C*12:03	DRB1*13:01	DQB1*06:03
HC1298	CONTIG1	A*02:01	B*15:17	C*07:01	DRB1*01:01	DQB1*03:02
	CONTIG2	A*68:01	B*44:02	C*07:04	DRB1*04:02	DQB1*05:01
HC1299	CONTIG1	A*03:01	B*15:01	C*03:03	DRB1*01:01	DQB1*05:01
	CONTIG2	A*24:02	B*35:08	C*04:01	DRB1*13:01	DQB1*06:03
HC1300	CONTIG1	A*02:01	B*27:05	C*01:02	DRB1*11:01	DQB1*03:01

	CONTIG2	-	B*35:03	C*03:03	DRB1*13:01	DQB1*06:03
HC1301	CONTIG1	A*02:01	B*13:02	C*03:04	DRB1*07:01	DQB1*02:02
	CONTIG2	A*31:01	B*15:01	C*06:02	DRB1*15:01	DQB1*06:02
HC1302	CONTIG1	A*01:01	B*08:01	C*03:03	DRB1*03:01	DQB1*02:01
	CONTIG2	A*25:01	B*15:01	C*07:01	DRB1*04:04	DQB1*03:02
HC1303	CONTIG1	A*25:01	B*40:01	C*03:04	DRB1*07:01	DQB1*02:02
	CONTIG2	A*29:02	B*44:03	C*16:01	DRB1*08:01	DQB1*04:02
HC1304	CONTIG1	A*02:01	B*41:02	C*02:02	DRB1*13:01	DQB1*03:01
	CONTIG2	A*66:01	B*51:01	C*17:03	DRB1*13:03	DQB1*06:03
HC1305	CONTIG1	A*02:01	B*08:01	C*07:01	DRB1*03:01	DQB1*02:01
	CONTIG2	A*26:01	B*38:01	C*12:03	DRB1*15:01	DQB1*06:02
HC1306	CONTIG1	A*01:01	B*07:02	C*07:01	DRB1*03:01	DQB1*03:02
	CONTIG2	A*03:01	B*08:01	C*07:02	DRB1*07:01	DQB1*03:03
HC1307	CONTIG1	A*25:01	B*18:01	C*04:01	DRB1*04:01	DQB1*03:02
	CONTIG2	A*68:01	B*35:03	C*12:03	DRB1*15:01	DQB1*06:03
HC1308	CONTIG1	A*02:01	B*35:01	C*04:01	DRB1*01:01	DQB1*05:01
	CONTIG2	A*03:01	B*44:02	C*14:02	DRB1*13:01	DQB1*06:03
HC1309	CONTIG1	A*03:01	B*07:02	C*07:02	DRB1*01:01	DQB1*05:01
	CONTIG2	A*30:01	B*38:01	C*12:03	DRB1*16:01	DQB1*05:02
HC1310	CONTIG1	A*23:01	B*44:03	C*04:01	DRB1*07:01	DQB1*02:02
	CONTIG2	A*24:02	B*51:01	C*14:02	DRB1*15:01	DQB1*06:02
HC1311	CONTIG1	A*02:01	B*07:02	C*07:02	DRB1*11:01	DQB1*03:01
	CONTIG2	A*03:01	B*44:02	C*07:04	DRB1*15:01	DQB1*06:02
HC1312	CONTIG1	A*02:01	B*18:01	C*05:01	DRB1*03:01	DQB1*02:01
	CONTIG2	A*24:02	B*38:01	C*12:03	DRB1*04:02	DQB1*03:02
HC1313	CONTIG1	A*01:01	B*07:02	C*07:01	DRB1*13:01	DQB1*06:02
	CONTIG2	-	B*27:05	C*07:02	DRB1*15:01	DQB1*06:03
HC1314	CONTIG1	A*02:01	B*18:03	C*07:01	DRB1*04:04	DQB1*03:01
	CONTIG2	A*11:01	B*51:01	C*15:02	DRB1*11:04	DQB1*03:02
HC1315	CONTIG1	A*01:01	B*07:02	C*04:01	DRB1*13:03	DQB1*03:01
	CONTIG2	A*03:01	B*35:01	C*07:02	DRB1*16:01	DQB1*05:02
HC1316	CONTIG1	A*02:01	B*35:01	C*04:01	DRB1*01:01	DQB1*03:01
	CONTIG2	A*11:01	B*44:02	C*05:01	DRB1*11:03	DQB1*05:01
HC1317	CONTIG1	A*30:02	B*27:02	C*02:02	DRB1*11:01	DQB1*03:01
	CONTIG2	A*68:01	B*40:01	C*03:04	DRB1*13:02	DQB1*06:04
HC1318	CONTIG1	A*02:01	B*27:05	C*01:02	DRB1*08:01	DQB1*03:01
	CONTIG2	-	B*44:02	C*05:01	DRB1*11:04	DQB1*04:02
HC1319	CONTIG1	A*02:01	B*35:01	C*02:02	DRB1*12:01	DQB1*03:01
	CONTIG2	A*11:01	B*44:05	C*04:01	DRB1*16:01	DQB1*05:02
HC1320	CONTIG1	A*25:01	B*18:01	C*12:03	DRB1*04:01	DQB1*03:02
	CONTIG2	A*26:01	B*38:01	-	DRB1*15:01	DQB1*06:02
HC1321	CONTIG1	A*01:01	B*40:01	C*03:04	DRB1*01:01	DQB1*05:01
	CONTIG2	-	B*52:01	C*12:02	DRB1*15:02	DQB1*06:01
HC1322	CONTIG1	A*03:01	B*35:01	C*04:01	DRB1*01:01	DQB1*05:01
	CONTIG2	A*11:01	-	-	DRB1*15:01	DQB1*06:02
HC1323	CONTIG1	A*03:01	B*07:02	C*07:01	DRB1*11:04	DQB1*03:01
	CONTIG2	A*30:01	B*18:01	C*07:02	DRB1*15:01	DQB1*06:02
HC1324	CONTIG1	A*03:01	B*35:01	C*04:01	DRB1*07:01	DQB1*02:02
	CONTIG2	A*11:01	B*45:01	C*06:02	DRB1*14:54	DQB1*05:03
HC1325	CONTIG1	A*24:02	B*35:01	C*04:01	DRB1*07:01	DQB1*03:01

	CONTIG2	A*31:01	B*51:01	C*15:02	DRB1*11:01	DQB1*03:03
HC1326	CONTIG1	A*02:01	B*15:01	C*03:04	DRB1*04:01	DQB1*02:02
	CONTIG2	A*24:02	B*18:01	C*07:01	DRB1*07:01	DQB1*03:02
HC1327	CONTIG1	A*01:01	B*13:02	C*06:02	DRB1*07:01	DQB1*02:02
	CONTIG2	A*24:02	B*57:01	-	-	DQB1*03:03
HC1328	CONTIG1	A*11:01	B*07:02	C*07:02	DRB1*01:01	DQB1*05:01
	CONTIG2	A*33:01	B*14:02	C*08:02	DRB1*01:02	-
HC1329	CONTIG1	A*02:01	B*15:01	C*03:03	DRB1*01:01	DQB1*05:01
	CONTIG2	A*24:02	B*38:01	C*12:03	DRB1*13:01	DQB1*06:03
HC1330	CONTIG1	A*25:01	B*18:01	C*07:01	DRB1*08:01	DQB1*04:02
	CONTIG2	-	B*49:01	C*12:03	DRB1*13:02	DQB1*06:04
HC1331	CONTIG1	A*01:01	B*08:01	C*07:01	DRB1*13:01	DQB1*06:03
	CONTIG2	A*29:02	B*38:01	C*12:03	-	-
HC1332	CONTIG1	A*02:01	B*07:04	C*07:02	DRB1*15:01	DQB1*06:02
	CONTIG2	A*24:02	B*18:01	C*12:03	-	-
HC1333	CONTIG1	A*02:01	B*13:02	C*06:02	DRB1*07:01	DQB1*02:02
	CONTIG2	A*11:01	B*38:01	C*12:03	DRB1*11:04	DQB1*03:01
HC1334	CONTIG1	A*02:01	B*51:01	C*01:02	DRB1*01:01	DQB1*03:03
	CONTIG2	A*31:01	B*57:01	C*06:02	DRB1*07:01	DQB1*05:01
HC1335	CONTIG1	A*03:01	B*07:02	C*04:01	DRB1*01:01	DQB1*05:01
	CONTIG2	A*11:01	B*35:01	C*07:02	DRB1*15:01	DQB1*06:02
HC1336	CONTIG1	A*01:01	B*18:01	C*07:01	DRB1*11:04	DQB1*03:01
	CONTIG2	A*02:01	B*52:01	C*12:02	DRB1*15:02	DQB1*06:01
HC1337	CONTIG1	A*03:01	B*51:01	C*01:02	DRB1*01:01	DQB1*05:01
	CONTIG2	A*24:02	B*56:01	C*14:02	DRB1*16:01	DQB1*05:02
HC1338	CONTIG1	A*02:01	B*13:02	C*04:01	DRB1*01:01	DQB1*05:01
	CONTIG2	A*24:02	B*35:01	C*06:02	-	-
HC1339	CONTIG1	A*24:02	B*18:01	C*07:01	DRB1*07:01	DQB1*02:02
	CONTIG2	A*68:01	B*44:03	C*16:01	DRB1*13:15	DQB1*03:01
HC1340	CONTIG1	A*02:01	B*07:02	C*07:02	DRB1*01:01	DQB1*03:02
	CONTIG2	A*03:01	B*44:03	C*16:02	DRB1*04:02	DQB1*05:01
HC1341	CONTIG1	A*02:01	B*07:02	C*03:04	DRB1*04:01	DQB1*03:02
	CONTIG2	A*03:01	B*15:01	C*07:02	DRB1*15:01	DQB1*06:02
HC1342	CONTIG1	A*03:01	B*07:02	C*07:02	DRB1*13:03	DQB1*03:01
	CONTIG2	A*25:01	B*41:02	C*17:03	DRB1*15:01	DQB1*06:02
HC1343	CONTIG1	A*03:01	B*35:01	C*04:01	DRB1*01:01	DQB1*05:01
	CONTIG2	-	B*44:27	C*07:04	DRB1*16:01	DQB1*05:02
HC1344	CONTIG1	A*02:01	B*15:01	C*03:04	DRB1*04:07	DQB1*02:02
	CONTIG2	A*29:02	B*44:03	C*16:01	DRB1*07:01	DQB1*03:01
HC1345	CONTIG1	A*03:01	B*35:03	C*04:01	DRB1*01:01	DQB1*05:01
	CONTIG2	A*24:02	B*44:02	C*05:01	DRB1*16:01	DQB1*05:02
HC1346	CONTIG1	A*02:01	B*18:01	C*03:03	DRB1*01:01	DQB1*03:02
	CONTIG2	A*03:01	B*35:01	C*07:01	DRB1*04:03	DQB1*05:01
HC1347	CONTIG1	A*01:01	B*08:01	C*07:01	DRB1*03:01	DQB1*02:01
	CONTIG2	-	-	-	-	-
HC1348	CONTIG1	A*02:01	B*07:02	C*03:03	DRB1*01:01	DQB1*05:01
	CONTIG2	A*32:01	B*15:01	C*07:02	DRB1*13:01	DQB1*06:03
HC1349	CONTIG1	A*24:02	B*07:02	C*07:02	DRB1*07:01	DQB1*02:02
	CONTIG2	-	B*51:01	C*14:02	DRB1*16:01	DQB1*05:02
HC1350	CONTIG1	A*03:01	B*27:05	C*02:02	DRB1*11:01	DQB1*03:01

	CONTIG2	-	B*35:01	C*04:01	DRB1*15:01	DQB1*06:02
HC1351	CONTIG1	A*03:01	B*35:01	C*04:01	DRB1*01:01	DQB1*05:01
	CONTIG2	A*26:01	B*38:01	C*12:03	DRB1*15:01	DQB1*06:03
HC1352	CONTIG1	A*02:01	B*44:03	C*04:01	DRB1*04:02	DQB1*02:02
	CONTIG2	A*23:01	-	C*16:02	DRB1*07:01	DQB1*03:02
HC1353	CONTIG1	A*11:01	B*07:02	C*07:01	DRB1*03:01	DQB1*02:01
	CONTIG2	A*26:01	B*08:01	C*07:02	DRB1*14:54	DQB1*05:03
HC1354	CONTIG1	A*02:01	B*44:02	C*03:03	DRB1*11:01	DQB1*03:01
	CONTIG2	A*11:01	B*55:01	C*05:01	DRB1*15:01	DQB1*06:02
HC1355	CONTIG1	A*01:01	B*07:02	C*07:02	DRB1*13:01	DQB1*05:03
	CONTIG2	A*02:01	B*38:01	C*12:03	DRB1*14:54	DQB1*06:88
HC1356	CONTIG1	A*01:01	B*08:01	C*03:03	DRB1*03:01	DQB1*02:01
	CONTIG2	A*03:01	B*15:01	C*07:01	DRB1*04:01	DQB1*03:02
HC1357	CONTIG1	A*02:01	B*15:01	C*03:03	DRB1*14:54	DQB1*05:03
	CONTIG2	-	B*51:01	C*15:02	DRB1*15:01	DQB1*06:02
HC1358	CONTIG1	A*02:01	B*14:02	C*05:01	DRB1*03:01	DQB1*02:01
	CONTIG2	A*34:02	B*44:02	C*08:02	DRB1*13:01	DQB1*06:03
HC1359	CONTIG1	A*02:01	B*13:02	C*06:02	DRB1*07:01	DQB1*03:03
	CONTIG2	A*24:02	B*57:01	-	DRB1*13:01	DQB1*06:03
HC1360	CONTIG1	A*02:01	B*07:02	C*03:04	DRB1*04:01	DQB1*03:02
	CONTIG2	A*03:01	B*15:01	C*07:02	DRB1*15:01	DQB1*06:02
HC1361	CONTIG1	A*01:01	B*08:01	C*01:02	DRB1*03:01	DQB1*02:01
	CONTIG2	A*11:01	B*56:01	C*07:01	DRB1*04:04	DQB1*03:02
HC1362	CONTIG1	A*02:01	B*07:02	C*07:02	DRB1*04:01	DQB1*03:02
	CONTIG2	A*26:01	B*38:01	C*12:03	DRB1*13:02	DQB1*06:07
HC1363	CONTIG1	A*02:09	B*07:02	C*07:02	DRB1*11:04	DQB1*03:01
	CONTIG2	A*11:01	B*38:01	C*12:03	DRB1*15:01	DQB1*06:03
HC1364	CONTIG1	A*02:01	B*07:02	C*03:03	DRB1*04:04	DQB1*03:02
	CONTIG2	A*31:01	B*15:01	C*07:02	DRB1*13:02	DQB1*06:09
HC1365	CONTIG1	A*02:01	B*15:01	C*03:03	DRB1*13:01	DQB1*05:03
	CONTIG2	-	B*44:02	C*05:01	DRB1*14:54	DQB1*06:03
HC1366	CONTIG1	A*01:01	B*35:08	C*04:01	DRB1*01:01	DQB1*05:01
	CONTIG2	A*24:02	B*52:01	C*12:02	DRB1*15:01	DQB1*06:02
HC1367	CONTIG1	A*03:01	B*07:02	C*07:02	DRB1*11:04	DQB1*03:01
	CONTIG2	A*33:03	B*18:01	C*12:03	DRB1*15:01	DQB1*06:02
HC1368	CONTIG1	A*01:01	B*35:03	C*01:02	DRB1*12:01	DQB1*03:01
	CONTIG2	A*03:01	B*51:01	C*04:01	DRB1*15:01	DQB1*06:02
HC1369	CONTIG1	A*02:01	B*07:02	C*04:01	DRB1*11:04	DQB1*03:01
	CONTIG2	A*03:01	B*44:03	C*07:02	DRB1*15:01	DQB1*06:02
HC1370	CONTIG1	A*01:01	B*07:02	C*05:01	DRB1*07:01	DQB1*02:02
	CONTIG2	A*03:01	B*44:02	C*07:02	DRB1*11:04	DQB1*03:01
HC1371	CONTIG1	A*02:01	B*40:01	C*03:04	DRB1*04:04	DQB1*03:02
	CONTIG2	-	B*44:27	C*07:04	DRB1*07:01	DQB1*03:03
HC1372	CONTIG1	A*01:01	B*08:01	C*04:01	DRB1*03:01	DQB1*02:01
	CONTIG2	-	B*35:03	C*07:01	DRB1*13:02	DQB1*06:04
HC1373	CONTIG1	A*02:01	B*27:02	C*02:02	DRB1*04:01	DQB1*03:02
	CONTIG2	-	B*37:01	C*06:02	DRB1*16:01	DQB1*05:02
HC1374	CONTIG1	A*02:01	B*13:02	C*06:02	DRB1*07:01	DQB1*02:02
	CONTIG2	-	B*38:01	C*12:03	DRB1*13:01	DQB1*06:03
HC1375	CONTIG1	A*02:01	B*07:02	C*04:01	DRB1*15:01	DQB1*06:02

	CONTIG2	A*03:01	B*35:01	C*07:02	-	-
HC1376	CONTIG1	A*24:02	B*18:01	C*07:01	DRB1*11:04	DQB1*03:01
	CONTIG2	A*26:01	-	-	DRB1*14:54	DQB1*05:03
HC1377	CONTIG1	A*24:02	B*18:01	C*12:03	DRB1*08:03	DQB1*03:01
	CONTIG2	A*25:01	B*51:01	C*14:02	DRB1*13:01	DQB1*06:03
HC1378	CONTIG1	A*11:01	B*18:01	C*02:02	DRB1*07:01	DQB1*03:03
	CONTIG2	A*25:01	B*27:05	C*12:03	DRB1*15:01	DQB1*06:02
HC1379	CONTIG1	A*01:01	B*51:01	C*07:02	DRB1*08:03	DQB1*03:01
	CONTIG2	A*24:02	B*52:01	C*12:02	DRB1*15:02	DQB1*06:01
HC1380	CONTIG1	A*23:01	B*38:01	C*04:01	DRB1*04:01	DQB1*02:02
	CONTIG2	A*66:01	B*44:03	C*12:03	DRB1*07:01	DQB1*03:02
HC1381	CONTIG1	A*26:01	B*07:02	C*06:02	DRB1*07:01	DQB1*02:02
	CONTIG2	A*30:01	B*13:02	C*07:02	DRB1*15:01	DQB1*06:02
HC1382	CONTIG1	A*02:05	B*08:01	C*03:03	DRB1*03:01	DQB1*02:01
	CONTIG2	A*26:01	B*44:02	C*07:01	DRB1*11:03	DQB1*03:01
HC1383	CONTIG1	A*02:01	B*15:01	C*03:03	DRB1*01:01	DQB1*05:01
	CONTIG2	A*26:01	B*35:01	C*07:01	DRB1*10:01	-
HC1384	CONTIG1	A*01:01	B*27:05	C*01:02	DRB1*08:01	DQB1*03:01
	CONTIG2	A*68:01	B*35:03	C*04:01	DRB1*12:01	DQB1*03:02
HC1385	CONTIG1	A*01:01	B*08:01	C*02:02	DRB1*03:01	DQB1*02:01
	CONTIG2	A*32:01	B*40:02	C*07:01	DRB1*15:01	DQB1*06:02
HC1386	CONTIG1	A*25:01	B*18:01	C*04:01	DRB1*04:01	DQB1*03:01
	CONTIG2	A*29:02	B*35:03	C*12:03	DRB1*11:01	DQB1*03:02
HC1387	CONTIG1	A*01:01	B*07:02	C*04:01	DRB1*11:01	DQB1*03:01
	CONTIG2	A*68:01	B*35:03	C*07:02	DRB1*15:01	DQB1*06:02
HC1388	CONTIG1	A*24:02	B*44:27	C*06:02	DRB1*11:02	DQB1*03:01
	CONTIG2	A*66:01	B*57:01	C*07:04	DRB1*16:01	DQB1*05:02
HC1389	CONTIG1	A*02:01	B*13:02	C*06:02	DRB1*07:01	DQB1*02:02
	CONTIG2	A*30:01	B*18:01	C*07:01	DRB1*11:04	DQB1*03:01
HC1390	CONTIG1	A*02:01	B*13:02	C*06:02	DRB1*07:01	DQB1*02:02
	CONTIG2	A*24:02	-	-	-	-
HC1391	CONTIG1	A*01:01	B*27:05	C*02:02	DRB1*04:02	DQB1*02:02
	CONTIG2	A*02:01	B*51:01	C*15:13	DRB1*07:01	DQB1*03:02
HC1392	CONTIG1	A*02:01	B*18:01	C*02:02	DRB1*04:01	DQB1*03:01
	CONTIG2	-	B*27:05	C*07:01	DRB1*04:03	DQB1*03:05
HC1393	CONTIG1	A*02:01	B*08:01	C*07:01	DRB1*03:01	DQB1*02:01
	CONTIG2	A*23:01	B*14:01	C*08:02	DRB1*07:01	DQB1*02:02
HC1394	CONTIG1	A*02:01	B*15:01	C*03:03	DRB1*04:04	DQB1*02:02
	CONTIG2	A*03:01	B*50:01	C*06:02	DRB1*07:01	DQB1*03:02
HC1395	CONTIG1	A*23:01	B*35:01	C*03:03	DRB1*04:08i	DQB1*03:04
	CONTIG2	A*24:02	B*35:03	C*04:01	DRB1*08:01	DQB1*04:02
HC1396	CONTIG1	A*02:01	B*07:02	C*02:02	DRB1*07:01	DQB1*03:01
	CONTIG2	-	B*27:05	C*07:02	DRB1*11:04	DQB1*03:03
HC1397	CONTIG1	A*03:01	B*18:01	C*04:01	DRB1*13:01	DQB1*06:02
	CONTIG2	A*25:01	B*35:01	C*12:03	DRB1*15:01	DQB1*06:03
HC1398	CONTIG1	A*03:01	B*14:02	C*08:02	DRB1*01:02	DQB1*04:02
	CONTIG2	A*25:01	B*41:01	C*17:01	DRB1*08:04	DQB1*05:01
HC1399	CONTIG1	A*01:01	B*35:02	C*04:01	DRB1*07:01	DQB1*02:02
	CONTIG2	A*02:01	B*50:01	C*06:02	DRB1*11:04	DQB1*03:01
HC1400	CONTIG1	A*03:01	B*08:01	C*07:02	DRB1*01:01	DQB1*03:01

	CONTIG2	A*24:02	B*35:03	C*12:03	DRB1*11:01	DQB1*05:01
HC1401	CONTIG1	A*02:01	B*07:02	C*07:02	DRB1*15:01	DQB1*06:02
	CONTIG2	A*25:01	B*18:01	C*12:03	-	-
HC1402	CONTIG1	A*01:01	B*08:01	C*07:01	DRB1*14:54	DQB1*05:02
	CONTIG2	A*02:01	B*44:27	C*07:04	DRB1*16:01	DQB1*05:03
HC1403	CONTIG1	A*01:01	B*35:01	C*03:03	DRB1*11:01	DQB1*03:01
	CONTIG2	A*66:01	B*35:02	C*04:01	DRB1*11:04	-
HC1404	CONTIG1	A*02:01	B*44:02	C*05:01	DRB1*03:01	DQB1*02:01
	CONTIG2	-	B*52:01	C*12:02	DRB1*15:02	DQB1*06:01
HC1405	CONTIG1	A*01:01	B*07:02	C*07:01	DRB1*03:01	DQB1*02:01
	CONTIG2	A*03:01	B*08:01	C*07:02	DRB1*15:01	DQB1*06:02
HC1406	CONTIG1	A*24:02	B*07:02	C*07:02	DRB1*13:01	DQB1*06:02
	CONTIG2	A*30:01	B*38:01	C*12:03	DRB1*15:01	DQB1*06:03
HC1407	CONTIG1	A*01:01	B*08:01	C*01:02	DRB1*01:01	DQB1*02:01
	CONTIG2	A*11:01	B*56:01	C*07:01	DRB1*03:01	DQB1*05:01
HC1408	CONTIG1	A*02:01	B*38:01	C*07:04	DRB1*13:01	DQB1*05:02
	CONTIG2	-	B*44:27	C*12:03	DRB1*16:01	DQB1*06:03
HC1409	CONTIG1	A*30:01	B*13:02	C*03:02	DRB1*07:01	DQB1*02:02
	CONTIG2	A*33:03	B*58:01	C*06:02	DRB1*13:02	DQB1*06:09
HC1410	CONTIG1	A*02:01	B*38:01	C*03:04	DRB1*01:01	DQB1*05:01
	CONTIG2	A*11:01	B*40:01	C*12:03	-	-
HC1411	CONTIG1	A*02:01	B*44:03	C*04:01	DRB1*07:01	DQB1*02:02
	CONTIG2	A*23:01	B*57:01	C*06:02	DRB1*16:01	DQB1*05:02
HC1412	CONTIG1	A*02:01	B*27:05	C*02:02	DRB1*01:01	DQB1*03:01
	CONTIG2	A*25:01	B*44:02	C*05:01	DRB1*11:01	DQB1*05:01
HC1413	CONTIG1	A*02:01	B*41:01	C*06:02	DRB1*03:01	DQB1*02:01
	CONTIG2	A*68:01	B*50:01	C*17:01	DRB1*07:01	DQB1*02:02
HC1414	CONTIG1	A*02:01	B*44:03	C*02:02	DRB1*07:01	DQB1*02:02
	CONTIG2	A*23:01	B*55:01	C*04:01	DRB1*11:01	DQB1*03:01
HC1415	CONTIG1	A*02:01	B*40:01	C*03:04	DRB1*13:02	DQB1*03:01
	CONTIG2	-	B*41:02	C*17:03	DRB1*13:03	DQB1*06:04
HC1416	CONTIG1	A*02:01	B*27:02	C*02:02	DRB1*04:01	DQB1*03:01
	CONTIG2	-	B*44:02	C*05:01	DRB1*13:01	DQB1*06:03
HC1417	CONTIG1	A*02:01	B*27:02	C*01:02	DRB1*01:01	DQB1*05:01
	CONTIG2	A*24:02	B*27:05	C*02:02	DRB1*16:01	DQB1*05:02
HC1418	CONTIG1	A*24:02	B*07:02	C*07:02	DRB1*04:02	DQB1*03:02
	CONTIG2	A*26:01	B*38:01	C*12:03	DRB1*15:01	DQB1*06:02
HC1419	CONTIG1	A*01:01	B*08:01	C*07:01	DRB1*03:01	DQB1*02:01
	CONTIG2	A*24:02	B*51:01	C*15:02	DRB1*11:01	DQB1*03:01
HC1420	CONTIG1	A*01:01	B*07:02	C*07:02	DRB1*04:04	DQB1*03:01
	CONTIG2	A*03:01	B*51:01	C*15:02	DRB1*11:04	DQB1*03:02
HC1421	CONTIG1	A*01:01	B*35:08	C*04:01	DRB1*07:01	DQB1*03:01
	CONTIG2	A*24:02	B*57:01	C*06:02	DRB1*11:01	DQB1*03:03
HC1422	CONTIG1	A*24:02	B*15:01	C*03:03	DRB1*01:01	DQB1*02:02
	CONTIG2	A*25:01	B*18:01	C*12:03	DRB1*07:01	DQB1*05:01
HC1423	CONTIG1	A*03:01	B*13:02	C*06:02	DRB1*07:01	DQB1*02:02
	CONTIG2	A*30:01	B*18:01	C*12:03	DRB1*15:01	DQB1*06:02
HC1424	CONTIG1	A*02:01	B*40:01	C*03:04	DRB1*04:01	DQB1*03:02
	CONTIG2	-	B*44:27	C*07:04	DRB1*09:01	DQB1*03:03
HC1425	CONTIG1	A*02:01	B*35:01	C*03:02	DRB1*01:01	DQB1*05:01

	CONTIG2	A*33:03	B*58:01	C*04:01	DRB1*13:02	DQB1*06:09
HC1426	CONTIG1	A*03:01	B*40:01	C*02:02	DRB1*11:01	DQB1*03:01
	CONTIG2	A*24:02	B*40:02	C*03:04	DRB1*15:01	DQB1*06:02
HC1427	CONTIG1	A*02:01	B*41:02	C*07:04	DRB1*13:03	DQB1*03:01
	CONTIG2	A*03:01	B*44:27	C*17:03	DRB1*15:01	DQB1*06:03
HC1428	CONTIG1	A*11:01	B*13:02	C*06:02	DRB1*07:01	DQB1*02:02
	CONTIG2	A*24:02	B*18:01	C*07:01	DRB1*14:54	DQB1*05:03
HC1429	CONTIG1	A*02:01	B*08:01	C*06:02	DRB1*03:01	DQB1*02:01
	CONTIG2	A*03:01	B*57:01	C*07:01	DRB1*07:01	DQB1*03:03
HC1430	CONTIG1	A*02:01	B*13:02	C*06:02	DRB1*07:01	DQB1*02:02
	CONTIG2	A*02:05	B*50:01	-	-	-
HC1431	CONTIG1	A*03:01	B*15:01	C*03:03	DRB1*01:01	DQB1*05:01
	CONTIG2	A*25:01	B*18:01	C*12:03	DRB1*13:01	DQB1*06:03
HC1432	CONTIG1	A*01:01	B*08:01	C*07:01	DRB1*08:01	DQB1*03:01
	CONTIG2	A*02:01	B*18:01	C*07:04	DRB1*11:01	DQB1*04:02
HC1433	CONTIG1	A*01:01	B*08:01	C*07:01	DRB1*03:01	DQB1*02:01
	CONTIG2	-	-	-	DRB1*16:01	DQB1*05:02
HC1434	CONTIG1	A*03:01	B*08:01	C*04:01	DRB1*01:01	DQB1*02:01
	CONTIG2	A*31:01	B*35:01	C*07:01	DRB1*03:01	DQB1*05:01
HC1435	CONTIG1	A*01:01	B*13:02	C*06:02	DRB1*07:01	DQB1*02:02
	CONTIG2	A*24:02	-	-	-	-
HC1436	CONTIG1	A*01:01	B*14:02	C*08:02	DRB1*07:01	DQB1*02:02
	CONTIG2	A*26:01	B*38:01	C*12:03	DRB1*15:01	DQB1*06:03
HC1437	CONTIG1	A*03:01	B*18:01	C*04:01	DRB1*03:01	DQB1*02:01
	CONTIG2	A*30:04	B*35:02	C*05:01	DRB1*11:04	DQB1*03:01
HC1438	CONTIG1	A*02:01	B*18:03	C*07:01	DRB1*11:04	DQB1*03:01
	CONTIG2	A*66:01	B*41:02	C*17:03	DRB1*13:03	-
HC1439	CONTIG1	A*01:01	B*35:03	C*04:01	DRB1*01:01	DQB1*05:01
	CONTIG2	A*03:01	B*57:01	C*06:02	DRB1*15:01	DQB1*06:02
HC1440	CONTIG1	A*24:02	B*35:03	C*03:04	DRB1*15:01	DQB1*06:01
	CONTIG2	A*68:01	B*40:01	C*04:01	DRB1*15:02	DQB1*06:02
HC1441	CONTIG1	A*23:01	B*27:02	C*02:02	DRB1*13:05	DQB1*03:01
	CONTIG2	A*32:01	B*35:01	C*04:01	DRB1*16:01	DQB1*05:02
HC1442	CONTIG1	A*23:01	B*44:02	C*07:01	DRB1*11:01	DQB1*03:01
	CONTIG2	A*68:01	B*49:01	C*07:04	DRB1*15:01	DQB1*06:02
HC1443	CONTIG1	A*02:01	B*07:02	C*08:02	DRB1*01:02	DQB1*02:01
	CONTIG2	A*11:01	B*14:02	C*15:02	DRB1*03:01	DQB1*05:01
HC1444	CONTIG1	A*02:01	B*15:01	C*03:03	DRB1*08:01	DQB1*03:02
	CONTIG2	A*68:01	B*35:03	C*04:01	DRB1*13:01	DQB1*06:03
HC1445	CONTIG1	A*24:02	B*40:01	C*03:04	DRB1*11:01	DQB1*03:01
	CONTIG2	A*26:01	B*44:03	C*04:01	-	-
HC1446	CONTIG1	A*24:02	B*07:02	C*07:02	DRB1*15:01	DQB1*06:02
	CONTIG2	-	-	-	-	-
HC1447	CONTIG1	A*02:05	B*44:03	C*06:02	DRB1*01:01	DQB1*02:02
	CONTIG2	A*29:02	B*50:01	C*16:01	DRB1*07:01	DQB1*05:01
HC1448	CONTIG1	A*23:01	B*38:01	C*06:02	DRB1*03:01	DQB1*02:01
	CONTIG2	A*26:01	B*50:01	C*12:03	DRB1*04:02	DQB1*03:02
HC1449	CONTIG1	A*03:01	B*35:01	C*04:01	DRB1*01:01	DQB1*05:01
	CONTIG2	A*11:01	B*52:01	C*12:02	-	-
HC1450	CONTIG1	A*01:01	B*08:01	C*07:01	DRB1*07:01	DQB1*02:02

	CONTIG2	A*02:01	B*38:01	C*12:03	DRB1*13:01	DQB1*06:03
HC1451	CONTIG1	A*01:01	B*07:02	C*07:02	DRB1*04:07	DQB1*03:01
	CONTIG2	A*03:01	B*44:03	C*07:06	DRB1*16:01	DQB1*05:02
HC1452	CONTIG1	A*02:01	B*35:01	C*04:01	DRB1*07:01	DQB1*03:01
	CONTIG2	A*24:02	B*57:01	C*06:02	DRB1*11:01	DQB1*03:03
HC1453	CONTIG1	A*01:01	B*07:02	C*03:04	DRB1*11:01	DQB1*03:01
	CONTIG2	A*31:01	B*40:01	C*07:02	DRB1*15:01	DQB1*06:02
HC1454	CONTIG1	A*02:01	B*18:01	C*04:01	DRB1*03:01	DQB1*02:01
	CONTIG2	A*11:01	B*35:01	C*07:01	DRB1*04:04	DQB1*03:02
HC1455	CONTIG1	A*25:01	B*18:01	C*01:02	DRB1*04:04	DQB1*03:02
	CONTIG2	A*26:01	B*56:01	C*12:03	DRB1*15:01	DQB1*06:02
HC1456	CONTIG1	A*02:01	B*08:01	C*05:01	DRB1*03:01	DQB1*02:01
	CONTIG2	-	B*44:02	C*07:01	DRB1*04:01	DQB1*03:01
HC1457	CONTIG1	A*03:01	B*35:01	C*04:01	DRB1*01:01	DQB1*03:02
	CONTIG2	A*29:02	B*44:03	C*16:01	DRB1*04:04	DQB1*05:01
HC1458	CONTIG1	A*02:01	B*56:01	C*01:02	DRB1*07:01	DQB1*03:03
	CONTIG2	A*11:01	B*57:01	C*06:02	DRB1*08:01	DQB1*04:02
HC1459	CONTIG1	A*02:01	B*13:02	C*03:04	DRB1*07:01	DQB1*02:02
	CONTIG2	A*30:01	B*40:01	C*06:02	DRB1*08:01	DQB1*04:02
HC1460	CONTIG1	A*02:01	B*07:02	C*03:04	DRB1*01:01	DQB1*03:03
	CONTIG2	A*03:01	B*40:01	C*07:02	DRB1*07:01	DQB1*05:01
HC1461	CONTIG1	A*24:02	B*07:02	C*07:02	DRB1*01:01	DQB1*05:01
	CONTIG2	A*26:01	-	-	-	-
HC1462	CONTIG1	A*03:01	B*37:01	C*06:02	DRB1*10:01	DQB1*05:01
	CONTIG2	A*68:01	B*57:01	C*07:04	DRB1*13:01	DQB1*06:03
HC1463	CONTIG1	A*23:01	B*13:02	C*04:01	DRB1*07:01	DQB1*02:02
	CONTIG2	A*24:02	B*44:03	C*06:02	DRB1*13:01	DQB1*06:03
HC1464	CONTIG1	A*25:01	B*35:01	C*02:02	DRB1*01:01	DQB1*03:01
	CONTIG2	A*26:01	B*44:05	C*04:01	DRB1*11:04	DQB1*05:01
HC1465	CONTIG1	A*01:01	B*08:01	C*07:01	DRB1*03:01	DQB1*02:01
	CONTIG2	A*29:02	B*44:03	C*16:01	DRB1*07:01	DQB1*02:02
HC1466	CONTIG1	A*02:01	B*27:05	C*01:02	DRB1*01:01	DQB1*05:01
	CONTIG2	A*11:01	B*44:27	C*07:04	DRB1*15:02	DQB1*06:01
HC1467	CONTIG1	A*01:01	B*08:01	C*07:01	DRB1*03:01	DQB1*02:01
	CONTIG2	A*02:01	B*44:27	C*07:04	DRB1*16:01	DQB1*05:02
HC1468	CONTIG1	A*02:01	B*18:01	C*03:03	DRB1*08:01	DQB1*03:01
	CONTIG2	-	B*35:01	C*07:01	DRB1*11:04	DQB1*04:02
HC1469	CONTIG1	A*02:01	B*13:02	C*06:02	DRB1*03:01	DQB1*02:01
	CONTIG2	A*32:01	B*57:01	-	DRB1*07:01	DQB1*03:03
HC1470	CONTIG1	A*02:01	B*13:02	C*05:01	DRB1*07:01	DQB1*02:02
	CONTIG2	A*31:01	B*27:05	C*06:02	DRB1*13:01	DQB1*06:03
HC1471	CONTIG1	A*01:01	B*37:01	C*02:02	DRB1*01:01	DQB1*03:02
	CONTIG2	A*02:01	B*40:02	C*06:02	DRB1*08:01	DQB1*05:01
HC1472	CONTIG1	A*01:01	B*27:05	C*06:02	DRB1*01:01	DQB1*03:05
	CONTIG2	A*02:01	B*57:01	C*15:11	DRB1*04:03	DQB1*05:01
HC1473	CONTIG1	A*01:01	B*08:01	C*07:01	DRB1*03:01	DQB1*02:01
	CONTIG2	-	B*35:03	C*12:03	DRB1*14:54	DQB1*05:03
HC1474	CONTIG1	A*02:01	B*38:01	C*05:01	DRB1*11:04	DQB1*03:01
	CONTIG2	A*26:01	B*44:02	C*12:03	DRB1*13:01	DQB1*06:03
HC1475	CONTIG1	A*01:01	B*08:01	C*07:01	DRB1*01:02	DQB1*02:01

	CONTIG2	A*24:02	B*14:02	C*08:02	DRB1*03:01	DQB1*05:01
HC1476	CONTIG1	A*02:01	B*07:02	C*06:02	DRB1*07:01	DQB1*02:02
	CONTIG2	A*03:01	B*13:02	C*07:02	DRB1*15:01	DQB1*06:02
HC1477	CONTIG1	A*25:01	B*14:02	C*08:02	DRB1*04:01	DQB1*02:02
	CONTIG2	A*68:02	B*18:01	C*12:03	DRB1*07:01	DQB1*03:02
HC1478	CONTIG1	A*02:01	B*14:02	C*04:01	DRB1*11:04	DQB1*03:01
	CONTIG2	A*24:02	B*35:02	C*08:02	DRB1*13:03	-
HC1479	CONTIG1	A*03:01	B*07:02	C*02:02	DRB1*01:01	DQB1*05:01
	CONTIG2	A*26:01	B*27:05	C*07:02	DRB1*15:01	DQB1*06:02
HC1480	CONTIG1	A*03:01	B*07:02	C*07:02	DRB1*04:02	DQB1*03:02
	CONTIG2	A*26:01	B*38:01	C*12:03	DRB1*10:01	DQB1*05:01
HC1481	CONTIG1	A*02:01	B*44:02	C*05:01	DRB1*04:01	DQB1*03:01
	CONTIG2	A*24:02	B*57:01	C*06:02	DRB1*16:01	DQB1*05:02
HC1482	CONTIG1	A*30:01	B*07:02	C*06:02	DRB1*04:01	DQB1*02:02
	CONTIG2	A*31:01	B*13:02	C*07:02	DRB1*07:01	DQB1*03:02
HC1483	CONTIG1	A*03:01	B*35:02	C*04:01	DRB1*01:01	DQB1*03:02
	CONTIG2	A*32:01	B*51:01	C*15:02	DRB1*04:04	DQB1*05:01
HC1484	CONTIG1	A*03:01	B*35:01	C*04:01	DRB1*01:01	DQB1*05:01
	CONTIG2	A*26:01	-	-	-	-
HC1485	CONTIG1	A*01:01	B*08:01	C*07:01	DRB1*12:01	DQB1*03:01
	CONTIG2	A*03:01	B*38:01	C*12:03	DRB1*15:01	DQB1*06:02
HC1486	CONTIG1	A*02:01	B*07:02	C*07:02	DRB1*15:01	DQB1*06:01
	CONTIG2	A*11:01	B*52:01	C*12:02	DRB1*15:02	DQB1*06:02
HC1487	CONTIG1	A*02:01	B*07:02	C*07:02	DRB1*01:02	DQB1*05:01
	CONTIG2	A*33:01	B*14:02	C*08:02	DRB1*14:54	DQB1*05:03
HC1488	CONTIG1	A*01:01	B*07:04	C*01:02	DRB1*04:01	DQB1*03:02
	CONTIG2	A*31:01	B*56:01	C*07:02	DRB1*15:01	DQB1*06:02
HC1489	CONTIG1	A*03:01	B*18:01	C*04:01	DRB1*03:01	DQB1*02:01
	CONTIG2	A*32:01	B*35:03	C*05:01	DRB1*12:01	DQB1*03:01
HC1490	CONTIG1	A*02:01	B*27:05	C*01:02	DRB1*01:01	DQB1*03:03
	CONTIG2	A*11:01	B*56:01	C*05:01	DRB1*09:01	DQB1*05:01
HC1491	CONTIG1	A*03:01	B*07:02	C*06:02	DRB1*13:02	DQB1*06:02
	CONTIG2	A*29:02	B*45:01	C*07:02	DRB1*15:01	DQB1*06:09
HC1492	CONTIG1	A*02:01	B*07:02	C*07:01	DRB1*09:01	DQB1*03:03
	CONTIG2	A*24:02	B*18:01	C*07:02	DRB1*13:01	DQB1*06:03
HC1493	CONTIG1	A*01:01	B*13:02	C*01:02	DRB1*07:01	DQB1*02:02
	CONTIG2	A*02:01	B*58:01	C*03:02	DRB1*11:01	DQB1*03:01
HC1494	CONTIG1	A*02:01	B*07:02	C*07:02	DRB1*11:04	DQB1*03:01
	CONTIG2	A*68:01	-	-	DRB1*15:01	DQB1*06:02
HC1495	CONTIG1	A*02:01	B*27:05	C*02:02	DRB1*04:04	DQB1*02:02
	CONTIG2	A*11:01	B*35:01	C*04:01	DRB1*07:01	DQB1*03:02
HC1496	CONTIG1	A*01:01	B*08:01	C*04:01	DRB1*07:01	DQB1*02:02
	CONTIG2	A*02:01	B*35:01	C*07:01	DRB1*13:05	DQB1*03:01
HC1497	CONTIG1	A*02:01	B*15:01	C*04:01	DRB1*03:01	DQB1*02:01
	CONTIG2	-	B*44:03	-	DRB1*08:01	DQB1*04:02
HC1498	CONTIG1	A*02:35	B*08:01	C*02:02	DRB1*03:01	DQB1*02:01
	CONTIG2	A*11:01	B*40:02	C*07:01	DRB1*11:01	DQB1*03:01
HC1499	CONTIG1	A*01:01	B*08:01	C*04:01	DRB1*03:01	DQB1*02:01
	CONTIG2	A*11:01	B*35:01	C*07:01	DRB1*07:01	DQB1*02:02
HC1500	CONTIG1	A*01:01	B*07:02	C*07:02	DRB1*15:01	DQB1*06:02

	CONTIG2	A*24:02	-	-	-	-
HC1501	CONTIG1	A*01:01	B*07:02	C*07:02	DRB1*13:01	DQB1*06:02
	CONTIG2	A*02:01	B*51:01	C*15:02	DRB1*15:01	DQB1*06:03
HC1502	CONTIG1	A*01:01	B*08:01	C*02:02	DRB1*01:01	DQB1*02:01
	CONTIG2	A*02:01	B*27:05	C*07:01	DRB1*03:01	DQB1*05:01
HC1503	CONTIG1	A*03:01	B*15:01	C*01:02	DRB1*04:01	DQB1*03:02
	CONTIG2	-	B*56:01	C*03:04	DRB1*13:01	DQB1*06:03
HC1504	CONTIG1	A*02:01	B*13:02	C*03:04	DRB1*07:01	DQB1*02:02
	CONTIG2	A*30:01	B*40:01	C*06:02	DRB1*08:01	DQB1*06:02
HC1505	CONTIG1	A*01:01	B*07:02	C*07:01	DRB1*07:01	DQB1*02:02
	CONTIG2	A*03:01	B*08:01	C*07:02	DRB1*15:01	DQB1*06:02
HC1506	CONTIG1	A*02:01	B*39:01	C*07:04	DRB1*07:01	DQB1*03:03
	CONTIG2	A*31:01	B*44:27	C*12:03	DRB1*15:01	DQB1*06:02
HC1507	CONTIG1	A*25:01	B*18:01	C*12:03	DRB1*11:01	DQB1*03:01
	CONTIG2	A*32:01	B*38:01	-	DRB1*11:04	-
HC1508	CONTIG1	A*02:01	B*13:02	C*03:03	DRB1*01:01	DQB1*02:02
	CONTIG2	A*32:01	B*15:01	C*06:02	DRB1*07:01	DQB1*05:01
HC1509	CONTIG1	A*02:01	B*44:02	C*04:01	DRB1*04:01	DQB1*02:02
	CONTIG2	A*23:01	B*44:03	C*05:01	DRB1*07:01	DQB1*03:02
HC1510	CONTIG1	A*02:01	B*27:05	C*02:02	DRB1*01:01	DQB1*03:03
	CONTIG2	A*03:01	B*35:01	C*04:01	DRB1*07:01	DQB1*05:01
HC1511	CONTIG1	A*02:01	B*14:02	C*08:02	DRB1*13:03	DQB1*03:01
	CONTIG2	A*68:02	B*51:01	C*15:02	DRB1*15:01	DQB1*06:02
HC1512	CONTIG1	A*01:01	B*38:01	C*07:04	DRB1*07:01	DQB1*02:02
	CONTIG2	A*02:35	B*44:27	C*12:03	DRB1*11:01	DQB1*03:01
HC1513	CONTIG1	A*01:01	B*07:02	C*06:02	DRB1*07:01	DQB1*02:02
	CONTIG2	A*32:01	B*50:01	C*07:02	DRB1*15:01	DQB1*06:02
HC1514	CONTIG1	A*03:01	B*08:01	C*03:03	DRB1*03:01	DQB1*02:01
	CONTIG2	A*26:01	B*44:02	C*07:01	DRB1*11:03	DQB1*03:01
HC1515	CONTIG1	A*03:01	B*35:03	C*04:01	DRB1*07:01	DQB1*03:03
	CONTIG2	A*24:02	B*57:01	C*06:02	DRB1*08:01	DQB1*04:02
HC1516	CONTIG1	A*02:01	B*07:02	C*06:02	DRB1*09:01	DQB1*02:02
	CONTIG2	A*23:01	B*45:01	C*07:02	DRB1*15:01	DQB1*06:02
HC1517	CONTIG1	A*01:01	B*57:01	C*06:02	DRB1*07:01	DQB1*02:02
	CONTIG2	A*02:01	B*57:03	C*07:01	-	DQB1*03:03
HC1518	CONTIG1	A*02:01	B*13:02	C*06:02	DRB1*01:01	DQB1*03:01
	CONTIG2	A*03:01	B*49:01	C*07:01	DRB1*11:01	DQB1*05:01
HC1519	CONTIG1	A*24:02	B*40:01	C*03:04	DRB1*01:01	DQB1*05:01
	CONTIG2	A*68:01	B*44:02	C*05:01	DRB1*13:02	DQB1*06:04
HC1520	CONTIG1	A*01:01	B*08:01	C*03:02	DRB1*03:01	DQB1*02:01
	CONTIG2	A*03:01	B*58:01	C*07:01	DRB1*04:05	DQB1*03:02
HC1521	CONTIG1	A*01:01	B*08:01	C*02:02	DRB1*07:01	DQB1*02:02
	CONTIG2	A*02:01	B*27:05	C*07:01	DRB1*15:01	DQB1*06:02
HC1522	CONTIG1	A*01:01	B*08:01	C*06:02	DRB1*03:01	DQB1*02:01
	CONTIG2	A*02:01	B*47:01	C*07:01	DRB1*07:01	DQB1*02:02
HC1523	CONTIG1	A*01:01	B*35:02	C*02:02	DRB1*11:01	DQB1*03:01
	CONTIG2	A*24:02	B*40:02	C*06:02	DRB1*11:04	-
HC1524	CONTIG1	A*03:01	B*15:01	C*04:01	DRB1*04:01	DQB1*03:01
	CONTIG2	A*66:01	B*41:02	C*17:03	DRB1*13:03	DQB1*03:02
HC1525	CONTIG1	A*01:01	B*44:02	C*05:01	DRB1*04:01	DQB1*02:02

	CONTIG2	A*02:01	B*44:03	C*16:01	DRB1*07:01	DQB1*03:01
HC1526	CONTIG1	A*02:01	B*07:02	C*02:02	DRB1*07:01	DQB1*03:03
	CONTIG2	-	B*27:05	C*07:02	DRB1*16:01	DQB1*05:02
HC1527	CONTIG1	A*02:01	B*15:01	C*01:02	DRB1*07:01	DQB1*02:02
	CONTIG2	A*26:01	-	C*04:01	-	DQB1*03:03
HC1528	CONTIG1	A*02:01	B*07:02	C*06:02	DRB1*08:01	DQB1*04:02
	CONTIG2	-	B*57:01	C*07:02	DRB1*13:01	DQB1*06:03
HC1529	CONTIG1	A*02:01	B*13:02	C*06:02	DRB1*07:01	DQB1*02:02
	CONTIG2	-	-	-	-	-
HC1530	CONTIG1	A*02:01	B*07:02	C*07:02	DRB1*15:01	DQB1*05:02
	CONTIG2	A*03:01	B*39:06	C*12:03	DRB1*16:01	DQB1*06:02
HC1531	CONTIG1	A*11:01	B*07:02	C*07:02	DRB1*11:01	DQB1*03:01
	CONTIG2	A*24:02	B*52:01	C*12:02	DRB1*15:01	DQB1*06:03
HC1532	CONTIG1	A*02:01	B*08:01	C*03:04	DRB1*03:01	DQB1*02:01
	CONTIG2	A*68:01	B*40:01	C*04:01	DRB1*13:02	DQB1*06:04
HC1533	CONTIG1	A*03:01	B*14:02	C*05:01	DRB1*01:01	DQB1*05:01
	CONTIG2	A*68:02	B*44:02	C*08:02	DRB1*14:54	DQB1*05:03
HC1534	CONTIG1	A*02:01	B*07:02	C*07:01	DRB1*11:04	DQB1*03:01
	CONTIG2	A*03:01	B*18:01	C*07:02	DRB1*16:01	DQB1*05:02
HC1535	CONTIG1	A*24:02	B*18:01	C*07:01	DRB1*13:01	DQB1*05:02
	CONTIG2	A*26:01	B*38:01	C*12:03	DRB1*16:01	DQB1*06:03
HC1536	CONTIG1	A*02:01	B*27:05	C*02:02	DRB1*04:04	DQB1*02:02
	CONTIG2	-	B*44:03	C*04:01	DRB1*07:01	DQB1*03:02
HC1537	CONTIG1	A*01:01	B*08:01	C*02:02	DRB1*04:01	DQB1*03:01
	CONTIG2	A*24:02	B*27:05	C*07:01	DRB1*15:01	DQB1*06:02
HC1538	CONTIG1	A*02:01	B*13:02	C*06:02	DRB1*07:01	DQB1*02:02
	CONTIG2	A*03:01	B*18:01	C*12:03	DRB1*10:01	DQB1*05:01
HC1539	CONTIG1	A*02:01	B*44:27	C*06:02	DRB1*07:01	DQB1*03:03
	CONTIG2	A*24:02	B*57:01	C*07:04	DRB1*15:01	DQB1*06:02
HC1540	CONTIG1	A*24:02	B*35:03	C*04:01	DRB1*08:01	DQB1*03:01
	CONTIG2	A*68:01	B*51:01	C*14:02	DRB1*11:01	DQB1*03:02
HC1541	CONTIG1	A*01:01	B*08:01	C*07:01	DRB1*03:01	DQB1*02:01
	CONTIG2	A*25:01	B*38:01	C*12:03	DRB1*04:01	DQB1*03:02
HC1542	CONTIG1	A*03:01	B*07:02	C*02:02	DRB1*15:01	DQB1*05:02
	CONTIG2	-	B*27:02	C*07:02	DRB1*16:01	DQB1*06:02
HC1543	CONTIG1	A*01:01	B*07:02	C*07:01	DRB1*03:01	DQB1*02:01
	CONTIG2	A*03:01	B*08:01	C*07:02	DRB1*11:01	DQB1*03:01
HC1544	CONTIG1	A*02:01	B*18:01	C*02:02	DRB1*11:04	DQB1*03:01
	CONTIG2	-	B*44:05	C*07:04	DRB1*16:01	DQB1*05:02
HC1545	CONTIG1	A*03:01	B*18:01	C*03:04	DRB1*04:01	DQB1*03:01
	CONTIG2	A*25:01	B*40:01	C*12:03	DRB1*13:03	-
HC1546	CONTIG1	A*02:01	B*15:01	C*03:04	DRB1*04:03	DQB1*03:05
	CONTIG2	-	B*18:01	C*07:01	DRB1*13:02	DQB1*06:04
HC1547	CONTIG1	A*01:01	B*08:01	C*07:01	DRB1*01:01	DQB1*02:01
	CONTIG2	A*32:01	B*51:01	C*12:03	DRB1*03:01	DQB1*05:01
HC1548	CONTIG1	A*03:01	B*35:01	C*04:01	DRB1*01:01	DQB1*02:01
	CONTIG2	A*32:01	B*51:01	C*15:02	DRB1*03:01	DQB1*05:01
HC1549	CONTIG1	A*02:01	B*07:02	C*07:02	DRB1*15:01	DQB1*06:02
	CONTIG2	A*03:01	-	-	-	-
HC1550	CONTIG1	A*01:01	B*15:17	C*04:01	DRB1*07:01	DQB1*02:02

	CONTIG2	A*23:01	B*44:03	C*07:01	DRB1*13:02	DQB1*05:01
HC1551	CONTIG1	A*02:01	B*07:02	C*02:02	DRB1*01:01	DQB1*03:03
	CONTIG2	-	B*27:05	C*07:02	DRB1*07:01	DQB1*05:01
HC1552	CONTIG1	A*01:01	B*13:02	C*06:02	DRB1*07:01	DQB1*02:02
	CONTIG2	A*02:01	B*37:01	-	DRB1*11:03	DQB1*03:01
HC1553	CONTIG1	A*11:01	B*07:02	C*04:01	DRB1*01:01	DQB1*05:01
	CONTIG2	A*32:01	B*35:01	C*07:02	-	-
HC1554	CONTIG1	A*02:01	B*13:02	C*04:01	DRB1*01:01	DQB1*02:02
	CONTIG2	A*24:02	B*35:01	C*06:02	DRB1*07:01	DQB1*05:01
HC1555	CONTIG1	A*01:01	B*08:01	C*03:04	DRB1*03:01	DQB1*02:01
	CONTIG2	A*03:01	B*40:01	C*07:01	DRB1*15:01	DQB1*06:02
HC1556	CONTIG1	A*02:01	B*07:02	C*03:03	DRB1*08:01	DQB1*04:02
	CONTIG2	-	B*15:01	C*07:02	DRB1*13:01	DQB1*06:03
HC1557	CONTIG1	A*24:02	B*07:02	C*07:02	DRB1*15:01	DQB1*06:02
	CONTIG2	A*25:01	B*18:01	C*12:03	-	-
HC1558	CONTIG1	A*02:01	B*07:05	C*04:01	DRB1*10:01	DQB1*05:01
	CONTIG2	A*03:01	B*52:01	C*12:02	DRB1*15:02	DQB1*06:01
HC1559	CONTIG1	A*02:01	B*18:01	C*06:02	DRB1*11:01	DQB1*03:01
	CONTIG2	-	B*57:01	C*07:01	DRB1*11:04	-
HC1560	CONTIG1	A*02:01	B*07:02	C*07:02	DRB1*15:01	DQB1*05:02
	CONTIG2	A*03:01	B*44:27	C*07:04	DRB1*16:01	DQB1*06:02
HC1561	CONTIG1	A*01:01	B*44:27	C*06:02	DRB1*01:01	DQB1*02:01
	CONTIG2	A*31:01	B*50:01	C*07:04	DRB1*03:01	DQB1*05:01
HC1562	CONTIG1	A*03:01	B*15:01	C*03:04	DRB1*01:01	DQB1*03:02
	CONTIG2	A*26:01	B*49:01	C*07:01	DRB1*04:01	DQB1*05:04
HC1563	CONTIG1	A*01:01	B*18:01	C*06:02	DRB1*11:01	DQB1*03:01
	CONTIG2	A*25:01	B*50:01	C*12:03	DRB1*12:01	-
HC1564	CONTIG1	A*01:01	B*07:02	C*07:01	DRB1*03:01	DQB1*02:01
	CONTIG2	A*03:01	B*08:01	C*07:02	DRB1*04:01	DQB1*03:01
HC1565	CONTIG1	A*02:01	B*44:05	C*01:02	DRB1*04:01	DQB1*03:01
	CONTIG2	-	B*56:01	C*02:02	DRB1*16:01	DQB1*05:02
HC1566	CONTIG1	A*02:01	B*13:02	C*03:04	DRB1*04:02	DQB1*02:02
	CONTIG2	A*32:01	B*15:01	C*06:02	DRB1*07:01	DQB1*03:02
HC1567	CONTIG1	A*30:01	B*13:02	C*06:02	DRB1*13:01	DQB1*06:02
	CONTIG2	A*68:01	B*51:01	C*15:02	DRB1*15:01	DQB1*06:03
HC1568	CONTIG1	A*02:01	B*27:05	C*02:02	DRB1*01:01	DQB1*05:01
	CONTIG2	-	B*44:05	-	DRB1*16:01	DQB1*05:02
HC1569	CONTIG1	A*02:01	B*18:01	C*05:01	DRB1*04:03	DQB1*03:01
	CONTIG2	A*68:02	B*44:02	C*07:01	DRB1*11:04	DQB1*03:02
HC1570	CONTIG1	A*01:01	B*08:01	C*05:01	DRB1*03:01	DQB1*02:01
	CONTIG2	A*02:01	B*44:02	C*07:01	DRB1*07:01	DQB1*02:02
HC1571	CONTIG1	A*01:01	B*07:02	C*07:01	DRB1*12:01	DQB1*03:01
	CONTIG2	A*03:01	B*08:01	C*07:02	DRB1*15:01	DQB1*06:02
HC1572	CONTIG1	A*02:01	B*07:02	C*06:02	DRB1*04:01	DQB1*03:01
	CONTIG2	A*03:01	B*45:01	C*07:02	DRB1*13:01	DQB1*06:03
HC1573	CONTIG1	A*02:01	B*07:02	C*04:01	DRB1*07:01	DQB1*03:03
	CONTIG2	-	B*15:01	C*07:02	DRB1*15:01	DQB1*06:02
HC1574	CONTIG1	A*03:01	B*07:02	C*07:02	DRB1*01:01	DQB1*05:01
	CONTIG2	A*24:02	B*38:01	C*12:03	DRB1*13:01	DQB1*06:03
HC1575	CONTIG1	A*03:01	B*07:02	C*04:01	DRB1*11:01	DQB1*03:01

	CONTIG2	A*23:01	B*35:01	C*12:03	DRB1*15:01	DQB1*05:02
HC1576	CONTIG1	A*24:02	B*35:01	C*02:02	DRB1*01:01	DQB1*04:02
	CONTIG2	A*25:01	B*40:02	C*12:03	DRB1*08:01	DQB1*05:01
HC1577	CONTIG1	A*03:01	B*07:02	C*07:02	DRB1*08:01	DQB1*04:02
	CONTIG2	A*68:01	B*39:01	C*12:03	DRB1*16:01	DQB1*05:02
HC1578	CONTIG1	A*24:02	B*38:01	C*02:02	DRB1*11:04	DQB1*03:01
	CONTIG2	-	B*44:05	C*12:03	DRB1*15:01	DQB1*06:02
HC1579	CONTIG1	A*23:01	B*44:02	C*04:01	DRB1*07:01	DQB1*02:02
	CONTIG2	A*25:01	B*44:03	C*07:04	DRB1*11:01	DQB1*03:01
HC1580	CONTIG1	A*02:01	B*44:02	C*05:01	DRB1*04:04	DQB1*03:01
	CONTIG2	A*11:01	B*51:01	C*15:02	DRB1*11:04	DQB1*03:02
HC1581	CONTIG1	A*02:01	B*07:02	C*02:02	DRB1*15:01	DQB1*05:02
	CONTIG2	-	B*27:02	C*07:02	DRB1*16:01	DQB1*06:02
HC1582	CONTIG1	A*01:01	B*18:01	C*06:02	DRB1*04:02	DQB1*03:02
	CONTIG2	-	B*37:01	C*07:01	DRB1*15:01	DQB1*06:02
HC1583	CONTIG1	A*01:01	B*08:01	C*07:01	DRB1*01:01	DQB1*02:01
	CONTIG2	A*02:01	B*38:01	C*12:03	DRB1*03:01	DQB1*05:01
HC1584	CONTIG1	A*03:01	B*15:01	C*04:01	DRB1*04:01	DQB1*03:01
	CONTIG2	A*31:01	B*39:01	C*12:03	DRB1*11:01	DQB1*03:02
HC1585	CONTIG1	A*02:01	B*07:02	C*03:04	DRB1*04:04	DQB1*03:02
	CONTIG2	A*03:01	B*40:01	C*07:02	DRB1*15:01	DQB1*06:02
HC1586	CONTIG1	A*02:01	B*18:01	C*04:01	DRB1*07:01	DQB1*02:02
	CONTIG2	A*24:02	B*35:01	C*07:01	DRB1*11:04	DQB1*03:01
HC1587	CONTIG1	A*01:01	B*14:02	C*02:02	DRB1*07:01	DQB1*02:02
	CONTIG2	A*03:01	B*27:02	C*08:02	DRB1*11:01	DQB1*03:01
HC1588	CONTIG1	A*02:01	B*15:01	C*03:03	DRB1*04:01	DQB1*03:02
	CONTIG2	A*26:01	B*55:01	C*03:04	DRB1*04:02	-
HC1589	CONTIG1	A*02:01	B*44:03	C*04:01	DRB1*03:01	DQB1*02:01
	CONTIG2	A*23:01	-	C*16:02	DRB1*04:02	DQB1*03:02
HC1590	CONTIG1	A*02:01	B*15:01	C*03:04	DRB1*07:01	DQB1*02:02
	CONTIG2	A*03:01	B*44:03	C*04:01	DRB1*11:01	DQB1*03:01
HC1591	CONTIG1	A*03:01	B*15:01	C*03:03	DRB1*13:01	DQB1*06:02
	CONTIG2	A*33:03	B*18:01	C*12:03	DRB1*15:01	DQB1*06:03
HC1592	CONTIG1	A*02:01	B*15:01	C*01:02	DRB1*04:01	DQB1*02:02
	CONTIG2	A*03:01	B*56:01	C*04:01	DRB1*07:01	DQB1*03:02
HC1593	CONTIG1	A*24:02	B*18:01	C*07:02	DRB1*11:01	DQB1*03:01
	CONTIG2	A*25:01	B*39:06	C*12:03	DRB1*13:02	DQB1*06:04
HC1594	CONTIG1	A*02:01	B*07:02	C*03:03	DRB1*04:01	DQB1*03:01
	CONTIG2	-	B*55:01	C*07:02	DRB1*07:01	DQB1*03:03
HC1595	CONTIG1	A*02:01	B*15:01	C*03:03	DRB1*04:04	DQB1*03:01
	CONTIG2	A*34:02	B*51:08	C*16:02	DRB1*13:03	DQB1*03:02
HC1596	CONTIG1	A*01:01	B*13:02	C*06:02	DRB1*07:01	DQB1*02:02
	CONTIG2	A*02:01	B*52:01	C*12:02	DRB1*15:02	DQB1*06:01
HC1597	CONTIG1	A*25:01	B*18:01	C*07:01	DRB1*04:01	DQB1*03:02
	CONTIG2	A*68:01	-	C*12:03	DRB1*13:01	DQB1*06:03
HC1598	CONTIG1	A*03:01	B*07:02	C*03:03	DRB1*12:01	DQB1*03:01
	CONTIG2	A*24:02	B*15:01	C*07:02	DRB1*13:01	DQB1*06:03
HC1599	CONTIG1	A*02:01	B*07:02	C*01:02	DRB1*01:01	DQB1*05:01
	CONTIG2	A*32:01	B*40:02	C*07:02	DRB1*15:01	DQB1*06:02
HC1600	CONTIG1	A*02:01	B*27:02	C*02:02	DRB1*15:01	DQB1*05:02

	CONTIG2	A*26:01	B*44:02	C*05:01	DRB1*16:01	DQB1*06:02
HC1601	CONTIG1	A*02:01	B*07:02	C*02:02	DRB1*01:01	DQB1*05:01
	CONTIG2	A*32:01	B*27:51	C*07:02	DRB1*15:01	DQB1*06:02
HC1602	CONTIG1	A*24:02	B*18:01	C*07:01	DRB1*07:01	DQB1*02:02
	CONTIG2	A*68:01	B*38:01	C*12:03	DRB1*15:01	DQB1*06:02
HC1603	CONTIG1	A*02:01	B*40:01	C*03:04	DRB1*07:01	DQB1*02:02
	CONTIG2	-	B*44:03	C*16:02	DRB1*13:02	DQB1*06:04
HC1604	CONTIG1	A*02:01	B*07:02	C*02:02	DRB1*11:03	DQB1*03:01
	CONTIG2	A*24:02	B*44:05	C*07:02	DRB1*13:01	DQB1*06:03
HC1605	CONTIG1	A*02:01	B*07:02	C*02:02	DRB1*15:01	DQB1*05:02
	CONTIG2	A*11:01	B*27:05	C*07:02	DRB1*16:01	DQB1*06:02
HC1606	CONTIG1	A*29:01	B*08:01	C*02:02	DRB1*03:01	DQB1*02:01
	CONTIG2	A*32:01	B*40:02	C*07:02	DRB1*04:01	DQB1*03:01
HC1607	CONTIG1	A*01:01	B*38:01	C*06:02	DRB1*04:02	DQB1*03:02
	CONTIG2	A*26:01	B*57:01	C*12:03	-	-
HC1608	CONTIG1	A*02:01	B*27:05	C*02:56	DRB1*01:01	DQB1*03:01
	CONTIG2	A*03:01	B*35:01	C*04:01	DRB1*11:01	DQB1*05:01
HC1609	CONTIG1	A*03:01	B*13:02	C*03:04	DRB1*07:01	DQB1*02:02
	CONTIG2	A*24:02	B*15:01	C*06:02	-	DQB1*03:03
HC1610	CONTIG1	A*24:02	B*38:01	C*05:01	DRB1*13:01	DQB1*05:02
	CONTIG2	A*26:01	B*44:02	C*12:03	DRB1*16:01	DQB1*06:03
HC1611	CONTIG1	A*01:01	B*08:01	C*07:01	DRB1*03:01	DQB1*02:01
	CONTIG2	-	-	-	-	-
HC1612	CONTIG1	A*11:01	B*35:01	C*04:01	DRB1*04:07	DQB1*03:01
	CONTIG2	A*25:01	B*39:01	C*12:03	DRB1*14:54	DQB1*05:03
HC1613	CONTIG1	A*02:01	B*08:01	C*04:01	DRB1*01:01	DQB1*02:01
	CONTIG2	A*23:01	B*44:03	-	DRB1*03:01	DQB1*05:01
HC1614	CONTIG1	A*30:01	B*08:01	C*06:02	DRB1*04:04	DQB1*03:02
	CONTIG2	A*33:03	B*13:02	C*07:01	DRB1*15:01	DQB1*06:03
HC1615	CONTIG1	A*03:01	B*35:03	C*04:01	DRB1*08:01	DQB1*04:02
	CONTIG2	A*24:02	B*39:06	C*07:02	-	-
HC1616	CONTIG1	A*24:02	B*41:02	C*01:02	DRB1*03:01	DQB1*02:01
	CONTIG2	A*32:01	B*51:01	C*17:03	DRB1*11:01	DQB1*03:01
HC1617	CONTIG1	A*02:01	B*07:02	C*07:02	DRB1*13:01	DQB1*06:02
	CONTIG2	A*24:02	B*38:01	C*12:03	DRB1*15:01	DQB1*06:03
HC1618	CONTIG1	A*02:01	B*18:01	C*02:02	DRB1*01:01	DQB1*05:01
	CONTIG2	A*25:01	B*27:05	C*12:03	DRB1*15:01	DQB1*06:02
HC1619	CONTIG1	A*02:01	B*13:02	C*03:03	DRB1*04:01	DQB1*02:02
	CONTIG2	A*24:02	B*15:01	C*06:02	DRB1*07:01	DQB1*03:02
HC1620	CONTIG1	A*03:01	B*13:02	C*06:02	DRB1*07:01	DQB1*02:02
	CONTIG2	-	B*39:01	C*12:03	-	-
HC1621	CONTIG1	A*01:01	B*39:01	C*06:02	DRB1*07:01	DQB1*03:01
	CONTIG2	A*31:01	B*57:01	C*12:03	DRB1*12:01	DQB1*03:03
HC1622	CONTIG1	A*01:01	B*08:01	C*01:02	DRB1*01:01	DQB1*02:01
	CONTIG2	A*02:01	B*27:05	C*07:01	DRB1*03:01	DQB1*05:01
HC1623	CONTIG1	A*25:01	B*18:01	C*01:02	DRB1*11:01	DQB1*03:01
	CONTIG2	A*31:01	B*40:02	C*12:03	DRB1*15:01	DQB1*06:02
HC1624	CONTIG1	A*02:01	B*13:02	C*01:02	DRB1*07:01	DQB1*02:02
	CONTIG2	A*11:01	B*52:01	C*12:02	DRB1*15:02	DQB1*06:01
HC1625	CONTIG1	A*02:01	B*07:05	C*03:04	DRB1*10:01	DQB1*05:01

	CONTIG2	A*25:01	B*40:01	C*04:01	DRB1*15:01	DQB1*06:02
HC1626	CONTIG1	A*24:02	B*07:02	C*01:02	DRB1*13:02	DQB1*06:02
	CONTIG2	-	B*51:01	C*07:02	DRB1*15:01	DQB1*06:04
HC1627	CONTIG1	A*02:01	B*44:02	C*03:03	DRB1*03:01	DQB1*02:01
	CONTIG2	A*03:01	B*55:01	C*05:01	DRB1*14:54	DQB1*05:03
HC1628	CONTIG1	A*01:01	B*07:02	C*07:02	DRB1*04:01	DQB1*03:02
	CONTIG2	A*02:01	B*38:01	C*12:03	DRB1*15:01	DQB1*06:02
HC1629	CONTIG1	A*02:01	B*35:03	C*04:01	DRB1*01:01	DQB1*03:01
	CONTIG2	A*11:01	B*38:01	C*12:03	DRB1*11:04	DQB1*05:01
HC1630	CONTIG1	A*01:01	B*35:01	C*03:03	DRB1*08:01	DQB1*04:02
	CONTIG2	A*11:01	B*37:01	C*06:02	DRB1*10:01	DQB1*05:01
HC1631	CONTIG1	A*01:01	B*08:01	C*04:01	DRB1*03:01	DQB1*02:01
	CONTIG2	A*03:01	B*40:02	C*07:01	DRB1*11:01	DQB1*03:01
HC1632	CONTIG1	A*01:01	B*08:01	C*02:02	DRB1*04:01	DQB1*03:02
	CONTIG2	A*02:01	B*27:02	C*07:01	-	-
HC1633	CONTIG1	A*23:01	B*44:02	C*04:01	DRB1*07:01	DQB1*02:02
	CONTIG2	A*68:01	B*44:03	C*07:68	DRB1*11:01	DQB1*03:01
HC1634	CONTIG1	A*25:01	B*14:01	C*08:02	DRB1*07:01	DQB1*02:02
	CONTIG2	A*32:01	B*39:01	C*12:03	DRB1*12:01	DQB1*03:01
HC1635	CONTIG1	A*24:02	B*35:01	C*04:01	DRB1*04:04	DQB1*03:01
	CONTIG2	A*30:01	B*49:01	C*07:01	DRB1*13:03	DQB1*03:02
HC1636	CONTIG1	A*02:01	B*27:05	C*02:02	DRB1*11:01	DQB1*03:01
	CONTIG2	A*24:02	B*44:27	C*07:04	DRB1*16:01	DQB1*05:02
HC1637	CONTIG1	A*01:01	B*40:02	C*02:26	DRB1*09:01	DQB1*03:01
	CONTIG2	A*02:01	B*44:03	C*16:01	DRB1*11:01	DQB1*03:03
HC1638	CONTIG1	A*02:01	B*15:01	C*03:03	DRB1*13:01	DQB1*06:03
	CONTIG2	A*32:01	B*40:01	C*03:04	-	-
HC1639	CONTIG1	A*02:01	B*15:01	C*03:03	DRB1*07:01	DQB1*02:02
	CONTIG2	-	B*57:01	C*06:02	DRB1*13:01	DQB1*06:03
HC1640	CONTIG1	A*01:01	B*39:01	C*07:04	DRB1*16:01	DQB1*05:02
	CONTIG2	A*03:01	B*44:27	C*12:03	-	-
HC1641	CONTIG1	A*02:01	B*15:01	C*01:02	DRB1*08:01	DQB1*04:02
	CONTIG2	-	B*27:05	C*03:03	DRB1*13:01	DQB1*06:03
HC1642	CONTIG1	A*02:01	B*08:01	C*03:04	DRB1*03:01	DQB1*02:01
	CONTIG2	A*03:01	B*57:01	C*07:01	DRB1*07:01	DQB1*03:03
HC1643	CONTIG1	A*11:01	B*18:03	C*01:02	DRB1*04:04	DQB1*03:01
	CONTIG2	-	B*44:02	C*07:01	DRB1*12:01	DQB1*03:02
HC1644	CONTIG1	A*02:01	B*07:02	C*03:04	DRB1*11:03	DQB1*03:01
	CONTIG2	A*25:01	B*15:01	C*07:02	DRB1*15:01	DQB1*06:02
HC1645	CONTIG1	A*11:01	B*08:01	C*07:01	DRB1*03:01	DQB1*02:01
	CONTIG2	A*29:02	B*44:03	C*16:01	DRB1*10:01	DQB1*05:01
HC1646	CONTIG1	A*02:01	B*18:01	C*12:03	DRB1*11:01	DQB1*03:01
	CONTIG2	A*25:01	B*51:01	C*15:02	DRB1*15:01	DQB1*06:02
HC1647	CONTIG1	A*03:01	B*07:02	C*03:04	DRB1*01:01	DQB1*05:01
	CONTIG2	A*24:02	B*40:01	C*07:02	DRB1*16:01	DQB1*05:02
HC1648	CONTIG1	A*01:01	B*08:01	C*07:01	DRB1*13:03	DQB1*03:01
	CONTIG2	A*30:02	B*58:01	C*07:18	DRB1*15:02	DQB1*06:01
HC1649	CONTIG1	A*25:01	B*27:05	C*02:02	DRB1*07:01	DQB1*03:01
	CONTIG2	A*32:01	B*40:02	-	DRB1*11:01	DQB1*03:03
HC1650	CONTIG1	A*11:01	B*35:01	C*02:02	DRB1*01:01	DQB1*03:01

	CONTIG2	A*26:01	B*40:02	C*04:01	DRB1*11:01	DQB1*05:01
HC1651	CONTIG1	A*02:01	B*18:01	C*01:02	DRB1*01:01	DQB1*03:01
	CONTIG2	A*25:01	B*27:05	C*12:03	DRB1*11:01	DQB1*05:01
HC1652	CONTIG1	A*24:02	B*07:02	C*07:02	DRB1*07:01	DQB1*02:02
	CONTIG2	A*30:01	B*14:01	C*08:02	DRB1*15:02	DQB1*06:01
HC1653	CONTIG1	A*02:01	B*44:02	C*05:01	DRB1*11:01	DQB1*03:01
	CONTIG2	A*24:02	B*51:01	-	DRB1*13:01	DQB1*06:03
HC1654	CONTIG1	A*11:01	B*40:02	C*01:02	DRB1*01:01	DQB1*03:01
	CONTIG2	A*24:02	B*56:01	C*02:02	DRB1*11:01	DQB1*05:01
HC1655	CONTIG1	A*02:01	B*27:05	C*01:02	DRB1*13:01	DQB1*05:03
	CONTIG2	-	B*44:02	C*05:01	DRB1*14:54	DQB1*06:03
HC1656	CONTIG1	A*03:01	B*07:02	C*07:01	DRB1*03:01	DQB1*02:01
	CONTIG2	A*11:01	B*08:01	C*07:02	DRB1*15:01	DQB1*06:02
HC1657	CONTIG1	A*03:01	B*07:02	C*07:02	DRB1*07:01	DQB1*03:03
	CONTIG2	A*24:02	-	-	DRB1*15:01	DQB1*06:02
HC1658	CONTIG1	A*01:01	B*44:03	C*03:02	DRB1*04:02	DQB1*02:02
	CONTIG2	A*33:03	B*58:01	C*04:01	DRB1*07:01	DQB1*03:02
HC1659	CONTIG1	A*02:01	B*18:01	C*02:02	DRB1*01:01	DQB1*05:01
	CONTIG2	A*25:01	B*27:05	C*12:03	DRB1*15:01	DQB1*06:02
HC1660	CONTIG1	A*02:01	B*07:04	C*03:04	DRB1*07:01	DQB1*02:02
	CONTIG2	A*03:01	B*40:01	C*07:02	DRB1*13:02	DQB1*06:04
HC1661	CONTIG1	A*01:01	B*08:01	C*06:02	DRB1*03:01	DQB1*02:01
	CONTIG2	A*26:01	B*57:01	C*07:01	DRB1*07:01	DQB1*03:03
HC1662	CONTIG1	A*24:02	B*15:18	C*01:02	DRB1*09:01	DQB1*03:01
	CONTIG2	A*68:01	B*27:05	C*07:04	DRB1*11:03	DQB1*03:03
HC1663	CONTIG1	A*03:01	B*14:02	C*04:01	DRB1*01:02	DQB1*03:01
	CONTIG2	A*11:01	B*35:01	C*08:02	DRB1*11:03	DQB1*05:01
HC1664	CONTIG1	A*01:01	B*35:03	C*04:01	DRB1*07:01	DQB1*03:03
	CONTIG2	A*30:01	B*57:01	C*06:02	DRB1*15:01	DQB1*06:02
HC1665	CONTIG1	A*02:01	B*13:02	C*04:01	DRB1*07:01	DQB1*02:02
	CONTIG2	-	B*44:03	C*06:02	DRB1*12:01	DQB1*03:01
HC1666	CONTIG1	A*01:01	B*08:01	C*04:01	DRB1*03:01	DQB1*02:01
	CONTIG2	A*11:01	B*51:01	C*07:01	DRB1*04:04	DQB1*03:02
HC1667	CONTIG1	A*02:01	B*18:01	C*02:02	DRB1*11:01	DQB1*03:01
	CONTIG2	-	B*27:02	C*07:01	DRB1*16:01	DQB1*05:02
HC1668	CONTIG1	A*02:01	B*13:02	C*03:04	DRB1*07:01	DQB1*02:02
	CONTIG2	A*26:01	B*40:01	C*06:02	DRB1*13:02	DQB1*06:04
HC1669	CONTIG1	A*02:01	B*27:05	C*02:02	DRB1*01:01	DQB1*03:03
	CONTIG2	A*11:01	B*57:01	C*06:02	DRB1*07:01	DQB1*05:01
HC1670	CONTIG1	A*24:02	B*07:02	C*04:01	DRB1*01:01	DQB1*05:01
	CONTIG2	A*26:08	B*35:03	C*07:02	DRB1*15:01	DQB1*06:02
HC1671	CONTIG1	A*02:01	B*15:01	C*02:02	DRB1*04:01	DQB1*03:01
	CONTIG2	-	B*27:02	C*03:04	DRB1*11:01	DQB1*03:02
HC1672	CONTIG1	A*24:02	B*15:01	C*01:02	DRB1*11:01	DQB1*03:01
	CONTIG2	-	B*51:01	C*03:03	DRB1*11:03	-
HC1673	CONTIG1	A*23:01	B*27:05	C*01:02	DRB1*01:01	DQB1*02:02
	CONTIG2	A*26:01	B*44:03	C*04:01	DRB1*07:01	DQB1*05:01
HC1674	CONTIG1	A*01:01	B*08:01	C*07:01	DRB1*03:01	DQB1*02:01
	CONTIG2	A*33:03	-	-	-	-
HC1675	CONTIG1	A*03:01	B*18:01	C*12:03	DRB1*01:01	DQB1*05:01

	CONTIG2	A*25:01	-	-	DRB1*15:01	DQB1*06:02
HC1676	CONTIG1	A*11:01	B*18:01	C*04:01	DRB1*07:01	DQB1*02:02
	CONTIG2	A*23:01	B*44:03	C*07:01	DRB1*11:04	DQB1*03:01
HC1677	CONTIG1	A*02:01	B*07:02	C*05:01	DRB1*04:01	DQB1*03:01
	CONTIG2	A*31:01	B*44:02	C*07:02	DRB1*15:01	DQB1*06:02
HC1678	CONTIG1	A*02:01	B*13:02	C*06:02	DRB1*04:01	DQB1*02:02
	CONTIG2	A*23:01	B*49:01	C*07:01	DRB1*07:01	DQB1*03:01
HC1679	CONTIG1	A*25:01	B*14:02	C*08:02	DRB1*07:01	DQB1*02:02
	CONTIG2	A*68:01	B*39:01	C*12:03	DRB1*11:01	DQB1*03:01
HC1680	CONTIG1	A*24:02	B*38:01	C*05:01	DRB1*07:01	DQB1*02:02
	CONTIG2	A*26:01	B*44:02	C*12:03	DRB1*09:01	DQB1*03:03
HC1681	CONTIG1	A*24:02	B*07:02	C*05:01	DRB1*12:01	DQB1*03:01
	CONTIG2	A*32:01	B*44:02	C*07:02	DRB1*15:01	DQB1*06:02
HC1682	CONTIG1	A*02:01	B*27:02	C*02:02	DRB1*11:01	DQB1*03:01
	CONTIG2	-	B*44:05	-	DRB1*16:01	DQB1*05:02
HC1683	CONTIG1	A*02:01	B*44:02	C*05:01	DRB1*04:01	DQB1*02:02
	CONTIG2	A*03:01	B*50:01	C*06:02	DRB1*07:01	DQB1*03:01
HC1684	CONTIG1	A*03:01	B*18:01	C*03:04	DRB1*07:01	DQB1*03:03
	CONTIG2	A*25:01	B*40:01	C*12:03	DRB1*15:01	DQB1*06:02
HC1685	CONTIG1	A*01:01	B*08:01	C*02:02	DRB1*04:01	DQB1*03:02
	CONTIG2	A*02:01	B*27:05	C*07:01	DRB1*07:01	DQB1*03:03
HC1686	CONTIG1	A*03:01	B*07:02	C*07:01	DRB1*15:01	DQB1*06:02
	CONTIG2	A*31:01	B*18:01	C*07:02	-	-
HC1687	CONTIG1	A*02:01	B*07:02	C*06:02	DRB1*15:01	DQB1*06:02
	CONTIG2	-	B*13:02	C*07:02	-	-
HC1688	CONTIG1	A*02:01	B*07:02	C*04:01	DRB1*13:11	DQB1*03:01
	CONTIG2	A*24:02	B*35:02	C*07:02	DRB1*15:01	DQB1*06:02
HC1689	CONTIG1	A*11:01	B*35:08	C*04:01	DRB1*08:01	DQB1*04:02
	CONTIG2	A*24:02	B*39:01	C*12:03	DRB1*16:01	DQB1*05:02
HC1690	CONTIG1	A*02:05	B*49:01	C*07:01	DRB1*13:02	DQB1*06:02
	CONTIG2	A*11:01	B*51:01	C*15:02	DRB1*15:01	DQB1*06:09
HC1691	CONTIG1	A*25:01	B*18:01	C*01:02	DRB1*01:01	DQB1*05:01
	CONTIG2	A*31:01	B*27:05	C*12:03	DRB1*13:01	DQB1*06:03
HC1692	CONTIG1	A*02:01	B*57:01	C*03:02	DRB1*07:01	DQB1*03:03
	CONTIG2	A*33:03	B*58:01	C*06:02	DRB1*15:01	DQB1*06:02
HC1693	CONTIG1	A*01:01	B*08:01	C*04:01	DRB1*03:01	DQB1*02:01
	CONTIG2	A*02:01	B*35:02	C*07:01	DRB1*04:04	DQB1*03:02
HC1694	CONTIG1	A*02:01	B*44:02	C*04:01	DRB1*04:01	DQB1*02:02
	CONTIG2	A*23:01	B*44:03	C*05:01	DRB1*07:01	DQB1*03:01
HC1695	CONTIG1	A*02:30	B*38:01	C*04:01	DRB1*07:01	DQB1*02:02
	CONTIG2	A*24:02	B*44:03	C*12:03	DRB1*13:01	DQB1*06:03
HC1696	CONTIG1	A*01:01	B*35:03	C*04:01	DRB1*04:01	DQB1*03:02
	CONTIG2	A*02:01	B*44:02	C*05:01	DRB1*08:01	DQB1*04:02
HC1697	CONTIG1	A*02:01	B*15:01	C*04:01	DRB1*07:01	DQB1*02:02
	CONTIG2	A*24:02	B*35:02	-	-	DQB1*03:03
HC1698	CONTIG1	A*24:02	B*38:01	C*03:04	DRB1*01:01	DQB1*05:04
	CONTIG2	A*26:01	B*40:01	C*12:03	DRB1*15:01	DQB1*06:02
HC1699	CONTIG1	A*03:01	B*07:02	C*04:01	DRB1*07:01	DQB1*02:02
	CONTIG2	A*23:01	B*44:03	C*07:02	DRB1*15:01	DQB1*06:02
HC1700	CONTIG1	A*02:01	B*38:01	C*06:02	DRB1*07:01	DQB1*03:03

	CONTIG2	A*31:01	B*57:01	C*12:03	DRB1*10:01	DQB1*05:01
HC1701	CONTIG1	A*03:01	B*27:05	C*01:02	DRB1*01:01	DQB1*03:01
	CONTIG2	A*66:01	B*41:02	C*17:03	DRB1*13:03	DQB1*05:01
HC1702	CONTIG1	A*24:03	B*38:01	C*06:02	DRB1*11:04	DQB1*03:01
	CONTIG2	A*68:01	B*51:01	C*15:04	-	-
HC1703	CONTIG1	A*01:01	B*15:01	C*01:02	DRB1*01:01	DQB1*02:01
	CONTIG2	A*11:01	B*18:01	C*05:01	DRB1*03:01	DQB1*05:01
HC1704	CONTIG1	A*30:01	B*40:02	C*01:02	DRB1*11:01	DQB1*03:01
	CONTIG2	A*31:01	B*52:01	C*12:02	DRB1*15:02	DQB1*06:01
HC1705	CONTIG1	A*02:01	B*35:01	C*04:01	DRB1*01:01	DQB1*05:01
	CONTIG2	A*11:01	B*44:27	C*07:04	DRB1*16:01	DQB1*05:02
HC1706	CONTIG1	A*03:01	B*07:02	C*03:03	DRB1*11:03	DQB1*03:01
	CONTIG2	A*24:31	B*15:01	C*07:02	DRB1*15:01	DQB1*06:02
HC1707	CONTIG1	A*01:01	B*07:02	C*07:01	DRB1*03:01	DQB1*02:01
	CONTIG2	A*03:01	B*08:01	C*07:02	DRB1*16:01	DQB1*05:02
HC1708	CONTIG1	A*02:01	B*52:01	C*06:02	DRB1*07:01	DQB1*03:03
	CONTIG2	A*11:01	B*57:01	C*12:02	DRB1*14:04	DQB1*05:03
HC1709	CONTIG1	A*02:01	B*35:03	C*02:02	DRB1*04:01	DQB1*03:02
	CONTIG2	A*32:01	B*40:02	C*04:01	DRB1*15:01	DQB1*06:39
HC1710	CONTIG1	A*01:01	B*37:01	C*06:02	DRB1*13:03	DQB1*03:01
	CONTIG2	-	B*57:01	-	DRB1*15:01	DQB1*06:02
HC1711	CONTIG1	A*24:02	B*13:02	C*06:02	DRB1*07:01	DQB1*02:02
	CONTIG2	A*66:01	B*41:02	C*17:03	DRB1*13:03	DQB1*03:01
HC1712	CONTIG1	A*02:01	B*15:01	C*01:02	DRB1*07:01	DQB1*02:02
	CONTIG2	A*03:01	B*51:01	C*03:03	DRB1*13:01	DQB1*06:03
HC1713	CONTIG1	A*02:01	B*15:01	C*01:02	DRB1*07:01	DQB1*02:02
	CONTIG2	-	B*44:27	C*07:04	DRB1*16:01	DQB1*05:02
HC1714	CONTIG1	A*02:01	B*38:01	C*12:03	DRB1*07:01	DQB1*02:02
	CONTIG2	A*26:01	-	-	DRB1*13:01	DQB1*06:03
HC1715	CONTIG1	A*03:01	B*35:01	C*04:01	DRB1*11:03	DQB1*03:01
	CONTIG2	-	B*38:01	C*12:03	DRB1*15:01	DQB1*06:03
HC1716	CONTIG1	A*01:01	B*40:01	C*03:04	DRB1*04:04	DQB1*03:02
	CONTIG2	A*02:01	B*57:01	C*06:02	DRB1*07:01	DQB1*03:03
HC1717	CONTIG1	A*02:01	B*18:01	C*07:04	DRB1*15:01	DQB1*05:02
	CONTIG2	A*25:01	B*44:27	C*12:03	DRB1*16:01	DQB1*06:02
HC1718	CONTIG1	A*24:02	B*27:05	C*01:02	DRB1*08:01	DQB1*04:02
	CONTIG2	A*25:01	B*51:01	C*14:02	DRB1*13:01	DQB1*06:03
HC1719	CONTIG1	A*02:01	B*40:01	C*03:04	DRB1*13:02	DQB1*06:04
	CONTIG2	A*32:01	B*44:03	C*12:03	-	-
HC1720	CONTIG1	A*01:01	B*07:02	C*06:02	DRB1*13:05	DQB1*03:01
	CONTIG2	A*03:01	B*57:01	C*07:02	DRB1*15:01	DQB1*06:02
HC1721	CONTIG1	A*11:01	B*07:02	C*07:02	DRB1*01:01	DQB1*03:02
	CONTIG2	-	B*51:01	C*15:02	DRB1*04:01	DQB1*05:01
HC1722	CONTIG1	A*01:01	B*18:01	C*04:01	DRB1*03:01	DQB1*02:01
	CONTIG2	A*24:02	B*35:01	C*05:01	DRB1*13:01	DQB1*06:03
HC1723	CONTIG1	A*03:01	B*15:01	C*04:01	DRB1*13:03	DQB1*03:01
	CONTIG2	A*11:01	B*35:01	-	DRB1*14:54	DQB1*05:03
HC1724	CONTIG1	A*02:01	B*07:02	C*07:02	DRB1*07:01	DQB1*02:02
	CONTIG2	A*11:01	B*50:01	C*15:02	-	DQB1*03:03
HC1725	CONTIG1	A*02:01	B*15:01	C*03:03	DRB1*07:01	DQB1*03:01

	CONTIG2	-	B*51:01	C*15:02	DRB1*11:01	DQB1*03:03
HC1726	CONTIG1	A*02:01	B*44:02	C*01:02	DRB1*04:01	DQB1*03:02
	CONTIG2	A*26:01	B*56:01	C*05:01	DRB1*08:01	DQB1*04:02
HC1727	CONTIG1	A*01:01	B*44:02	C*05:01	DRB1*15:02	DQB1*05:02
	CONTIG2	-	B*52:01	C*12:02	DRB1*16:01	DQB1*06:01
HC1728	CONTIG1	A*11:01	B*14:02	C*08:02	DRB1*04:08	DQB1*03:01
	CONTIG2	A*68:02	B*35:03	C*12:03	DRB1*13:03	DQB1*03:04
HC1729	CONTIG1	A*01:01	B*08:01	C*01:02	DRB1*03:01	DQB1*02:01
	CONTIG2	A*02:01	B*56:01	C*07:01	DRB1*07:01	DQB1*02:02
HC1730	CONTIG1	A*02:01	B*07:02	C*05:01	DRB1*04:01	DQB1*03:01
	CONTIG2	-	B*44:02	C*07:02	DRB1*15:01	DQB1*06:02
HC1731	CONTIG1	A*24:02	B*35:03	C*04:01	DRB1*11:12	DQB1*03:01
	CONTIG2	A*68:01	B*41:01	C*17:01	DRB1*13:02	DQB1*06:04
HC1732	CONTIG1	A*01:01	B*27:05	C*02:02	DRB1*07:01	DQB1*03:03
	CONTIG2	A*02:01	B*37:01	C*06:02	DRB1*15:01	DQB1*06:02
HC1733	CONTIG1	A*02:01	B*51:01	C*06:02	DRB1*07:01	DQB1*03:01
	CONTIG2	A*03:01	B*57:01	C*16:02	DRB1*12:01	DQB1*03:03
HC1734	CONTIG1	A*01:01	B*08:01	C*07:01	DRB1*01:01	DQB1*02:01
	CONTIG2	A*23:01	B*49:01	-	DRB1*03:01	DQB1*05:04
HC1735	CONTIG1	A*02:01	B*44:27	C*06:02	DRB1*07:01	DQB1*02:02
	CONTIG2	A*02:05	B*50:01	C*07:04	DRB1*16:01	DQB1*05:02
HC1736	CONTIG1	A*02:01	B*18:01	C*04:01	DRB1*11:12	DQB1*03:01
	CONTIG2	A*68:01	B*35:03	C*12:03	DRB1*15:01	DQB1*06:02
HC1737	CONTIG1	A*03:01	B*15:01	C*03:04	DRB1*01:01	DQB1*05:01
	CONTIG2	A*26:01	B*38:01	C*12:03	DRB1*13:01	DQB1*06:03
HC1738	CONTIG1	A*24:02	B*07:02	C*03:04	DRB1*01:01	DQB1*05:01
	CONTIG2	A*68:01	B*40:01	C*07:02	DRB1*15:01	DQB1*06:02
HC1739	CONTIG1	A*02:01	B*44:27	C*07:04	DRB1*04:07	DQB1*03:01
	CONTIG2	A*03:01	B*51:01	C*14:02	DRB1*16:01	DQB1*05:02
HC1740	CONTIG1	A*01:01	B*07:02	C*07:02	DRB1*15:01	DQB1*06:01
	CONTIG2	A*24:02	B*52:01	C*12:02	DRB1*15:02	DQB1*06:02
HC1741	CONTIG1	A*02:01	B*35:01	C*04:01	DRB1*01:01	DQB1*03:01
	CONTIG2	A*11:01	B*41:02	C*17:03	DRB1*13:03	DQB1*05:01
HC1742	CONTIG1	A*03:01	B*27:05	C*02:02	DRB1*01:02	DQB1*03:03
	CONTIG2	-	B*50:01	C*06:02	DRB1*09:01	DQB1*05:01
HC1743	CONTIG1	A*11:01	B*27:05	C*01:02	DRB1*01:01	DQB1*03:02
	CONTIG2	A*24:02	B*35:01	C*04:01	DRB1*04:04	DQB1*05:01
HC1744	CONTIG1	A*02:01	B*08:01	C*04:01	DRB1*01:01	DQB1*02:01
	CONTIG2	A*03:01	B*35:01	C*07:01	DRB1*03:01	DQB1*05:01
HC1745	CONTIG1	A*02:01	B*38:01	C*06:02	DRB1*04:01	DQB1*03:01
	CONTIG2	A*26:01	B*57:01	C*12:03	DRB1*07:01	DQB1*03:03
HC1746	CONTIG1	A*11:01	B*27:05	C*01:02	DRB1*03:01	DQB1*02:01
	CONTIG2	A*31:01	B*56:01	C*02:02	DRB1*15:01	DQB1*06:02
HC1747	CONTIG1	A*01:01	B*39:01	C*07:01	DRB1*11:01	DQB1*03:01
	CONTIG2	A*02:01	B*49:01	C*12:03	-	-
HC1748	CONTIG1	A*01:01	B*57:01	C*06:02	DRB1*07:01	DQB1*03:01
	CONTIG2	A*02:01	-	-	DRB1*13:03	DQB1*03:03
HC1749	CONTIG1	A*03:01	B*07:02	C*03:03	DRB1*11:03	DQB1*03:01
	CONTIG2	A*26:01	B*44:02	C*07:02	DRB1*15:01	DQB1*06:02
HC1750	CONTIG1	A*02:01	B*15:01	C*04:01	DRB1*08:01	DQB1*03:02

	CONTIG2	-	B*35:03	-	-	DQB1*04:02
HC1751	CONTIG1	A*24:02	B*41:02	C*16:01	DRB1*13:03	DQB1*03:01
	CONTIG2	A*31:01	B*44:03	C*17:03	DRB1*15:01	DQB1*06:02
HC1752	CONTIG1	A*11:01	B*07:02	C*03:04	DRB1*15:01	DQB1*05:02
	CONTIG2	A*26:01	B*15:01	C*07:02	DRB1*16:01	DQB1*06:02
HC1753	CONTIG1	A*11:01	B*38:01	C*07:01	DRB1*01:01	DQB1*05:04
	CONTIG2	A*30:02	B*49:01	C*12:03	DRB1*13:02	DQB1*06:09
HC1754	CONTIG1	A*03:01	B*35:01	C*04:01	DRB1*08:01	DQB1*04:02
	CONTIG2	A*11:01	B*35:03	-	DRB1*14:54	DQB1*05:03
HC1755	CONTIG1	A*02:01	B*08:01	C*06:02	DRB1*03:01	DQB1*02:01
	CONTIG2	A*23:01	B*50:01	C*07:01	-	-
HC1756	CONTIG1	A*01:01	B*08:01	C*02:02	DRB1*03:01	DQB1*02:01
	CONTIG2	-	B*40:02	C*07:01	DRB1*11:01	DQB1*03:01
HC1757	CONTIG1	A*01:01	B*07:02	C*07:02	DRB1*01:01	DQB1*03:01
	CONTIG2	A*02:01	B*39:31	C*12:03	DRB1*04:07	DQB1*05:01
HC1758	CONTIG1	A*24:02	B*07:02	C*05:01	DRB1*11:04	DQB1*03:01
	CONTIG2	A*31:01	B*18:01	C*12:03	DRB1*15:01	DQB1*06:02
HC1759	CONTIG1	A*01:01	B*35:02	C*03:03	DRB1*11:04	DQB1*03:01
	CONTIG2	A*24:02	B*55:01	C*04:01	DRB1*16:01	DQB1*05:02
HC1760	CONTIG1	A*02:01	B*27:05	C*01:02	DRB1*01:01	DQB1*02:02
	CONTIG2	A*23:01	B*44:03	C*04:01	DRB1*07:01	DQB1*05:01
HC1761	CONTIG1	A*03:01	B*08:01	C*07:01	DRB1*03:01	DQB1*02:01
	CONTIG2	A*23:01	B*44:03	C*16:01	DRB1*07:01	DQB1*02:02
HC1762	CONTIG1	A*30:01	B*18:01	C*03:04	DRB1*03:01	DQB1*02:01
	CONTIG2	A*68:01	B*40:01	C*07:01	DRB1*11:04	DQB1*03:01
HC1763	CONTIG1	A*02:01	B*27:05	C*02:02	DRB1*15:01	DQB1*05:02
	CONTIG2	A*24:02	B*40:01	C*03:04	DRB1*16:01	DQB1*06:02
HC1764	CONTIG1	A*11:01	B*13:02	C*06:02	DRB1*07:01	DQB1*02:02
	CONTIG2	A*24:02	B*51:01	C*14:02	DRB1*11:01	DQB1*03:01
HC1765	CONTIG1	A*02:01	B*07:02	C*06:02	DRB1*13:01	DQB1*06:02
	CONTIG2	-	B*13:02	C*07:02	DRB1*15:01	DQB1*06:03
HC1766	CONTIG1	A*02:01	B*13:02	C*06:02	DRB1*04:01	DQB1*03:02
	CONTIG2	A*24:02	B*51:01	C*15:02	DRB1*10:01	DQB1*05:01
HC1767	CONTIG1	A*01:01	B*08:01	C*07:01	DRB1*03:01	DQB1*02:01
	CONTIG2	A*25:01	B*18:01	C*12:03	DRB1*15:01	DQB1*06:02
HC1768	CONTIG1	A*01:01	B*08:01	C*07:01	DRB1*09:01	DQB1*03:03
	CONTIG2	A*24:02	B*51:01	C*15:02	DRB1*16:01	DQB1*05:02
HC1769	CONTIG1	A*25:01	B*18:01	C*12:03	DRB1*04:01	DQB1*03:02
	CONTIG2	A*26:01	-	-	DRB1*15:01	DQB1*06:02
HC1770	CONTIG1	A*02:01	B*18:01	C*07:01	DRB1*11:04	DQB1*03:01
	CONTIG2	A*24:02	-	C*12:03	-	-
HC1771	CONTIG1	A*03:01	B*14:02	C*08:02	DRB1*11:03	DQB1*03:01
	CONTIG2	A*66:01	B*41:02	C*17:03	DRB1*13:03	-
HC1772	CONTIG1	A*01:01	B*18:01	C*05:01	DRB1*10:01	DQB1*05:01
	CONTIG2	A*25:01	B*44:02	C*12:03	DRB1*13:01	DQB1*06:03
HC1773	CONTIG1	A*26:01	B*38:01	C*12:03	DRB1*13:01	DQB1*06:02
	CONTIG2	A*30:01	B*51:01	C*15:02	DRB1*15:01	DQB1*06:03
HC1774	CONTIG1	A*02:01	B*27:05	C*02:02	DRB1*09:01	DQB1*03:01
	CONTIG2	A*68:01	B*41:02	C*17:03	DRB1*13:03	DQB1*03:03
HC1775	CONTIG1	A*03:01	B*18:01	C*02:02	DRB1*04:04	DQB1*03:02

	CONTIG2	A*25:01	B*27:05	C*12:03	DRB1*15:01	DQB1*06:02
HC1776	CONTIG1	A*01:01	B*13:02	C*06:02	DRB1*04:04	DQB1*02:02
	CONTIG2	A*11:01	B*51:01	C*15:02	DRB1*07:01	DQB1*03:02
HC1777	CONTIG1	A*01:01	B*18:01	C*12:03	DRB1*03:01	DQB1*02:01
	CONTIG2	A*25:01	B*51:01	-	DRB1*15:01	DQB1*06:02
HC1778	CONTIG1	A*02:01	B*07:02	C*07:02	DRB1*12:01	DQB1*03:01
	CONTIG2	A*11:01	B*39:06	C*12:03	DRB1*16:01	DQB1*05:02
HC1779	CONTIG1	A*03:01	B*07:02	C*04:01	DRB1*07:01	DQB1*02:02
	CONTIG2	A*23:01	B*44:03	C*07:02	DRB1*15:01	DQB1*06:02
HC1780	CONTIG1	A*02:01	B*07:02	C*03:04	DRB1*12:01	DQB1*03:01
	CONTIG2	A*03:01	B*40:01	C*07:02	DRB1*15:01	DQB1*06:02
HC1781	CONTIG1	A*01:01	B*40:01	C*01:02	DRB1*04:01	DQB1*03:01
	CONTIG2	A*02:01	B*51:01	C*03:04	DRB1*11:01	DQB1*03:02
HC1782	CONTIG1	A*02:01	B*07:02	C*07:02	DRB1*07:01	DQB1*02:02
	CONTIG2	A*23:01	B*44:03	C*16:01	DRB1*15:01	DQB1*06:02
HC1783	CONTIG1	A*02:01	B*08:01	C*06:02	DRB1*03:01	DQB1*02:01
	CONTIG2	A*11:01	B*13:02	C*07:01	DRB1*08:04	DQB1*04:02
HC1784	CONTIG1	A*02:01	B*18:01	C*07:01	DRB1*11:01	DQB1*03:01
	CONTIG2	-	B*51:01	C*12:03	DRB1*11:04	-
HC1785	CONTIG1	A*02:01	B*35:03	C*12:03	DRB1*13:01	DQB1*05:03
	CONTIG2	A*26:01	B*38:01	-	DRB1*14:54	DQB1*06:03
HC1786	CONTIG1	A*25:01	B*18:01	C*12:03	DRB1*04:01	DQB1*02:02
	CONTIG2	A*29:02	B*44:03	C*16:01	DRB1*07:01	DQB1*03:02
HC1787	CONTIG1	A*01:01	B*15:01	C*03:03	DRB1*04:01	DQB1*03:01
	CONTIG2	A*02:01	B*51:01	C*14:02	DRB1*08:03	DQB1*03:02
HC1788	CONTIG1	A*02:01	B*39:24	C*07:01	DRB1*13:01	DQB1*03:01
	CONTIG2	-	B*51:01	C*14:02	DRB1*13:03	DQB1*06:03
HC1789	CONTIG1	A*24:02	B*27:02	C*01:02	DRB1*07:01	DQB1*02:02
	CONTIG2	A*31:01	B*56:01	C*02:02	DRB1*16:01	DQB1*05:02
HC1790	CONTIG1	A*24:02	B*13:02	C*06:02	DRB1*07:01	DQB1*02:02
	CONTIG2	A*26:01	B*51:01	C*16:02	DRB1*16:01	DQB1*05:02
HC1791	CONTIG1	A*02:01	B*40:02	C*02:02	DRB1*01:02	DQB1*03:01
	CONTIG2	-	B*44:02	C*05:01	DRB1*12:01	DQB1*05:01
HC1792	CONTIG1	A*02:01	B*07:02	C*03:03	DRB1*13:01	DQB1*06:03
	CONTIG2	-	B*15:01	C*07:02	DRB1*13:02	DQB1*06:04
HC1793	CONTIG1	A*01:01	B*35:01	C*04:01	DRB1*01:01	DQB1*03:01
	CONTIG2	-	B*37:01	C*06:02	DRB1*12:01	DQB1*05:01
HC1794	CONTIG1	A*02:01	B*07:02	C*06:02	DRB1*13:05	DQB1*03:01
	CONTIG2	A*03:01	B*35:03	C*07:02	DRB1*15:01	DQB1*06:02
HC1795	CONTIG1	A*02:01	B*27:02	C*02:02	DRB1*01:01	DQB1*05:01
	CONTIG2	A*11:01	B*35:01	C*04:01	DRB1*16:01	DQB1*05:02
HC1796	CONTIG1	A*01:01	B*07:02	C*07:01	DRB1*13:01	DQB1*06:02
	CONTIG2	-	B*18:01	C*07:02	DRB1*15:01	DQB1*06:03
HC1797	CONTIG1	A*11:01	B*27:05	C*01:02	DRB1*01:01	DQB1*03:03
	CONTIG2	A*26:01	B*57:01	C*06:02	DRB1*07:01	DQB1*05:01
HC1798	CONTIG1	A*02:01	B*35:01	C*04:01	DRB1*13:02	DQB1*06:04
	CONTIG2	A*26:01	B*51:08	C*16:02	-	-
HC1799	CONTIG1	A*01:01	B*08:01	C*07:01	DRB1*03:01	DQB1*02:01
	CONTIG2	-	-	-	-	-
HC1800	CONTIG1	A*02:01	B*07:02	C*07:02	DRB1*01:01	DQB1*05:01

	CONTIG2	A*11:01	B*52:01	C*12:02	DRB1*15:01	DQB1*06:02
HC1801	CONTIG1	A*01:01	B*08:01	C*07:01	DRB1*03:01	DQB1*02:01
	CONTIG2	A*02:01	B*51:01	C*14:02	DRB1*13:01	DQB1*06:03
HC1802	CONTIG1	A*01:01	B*40:02	C*02:02	DRB1*01:02	DQB1*02:02
	CONTIG2	A*02:01	B*41:01	C*17:01	DRB1*07:01	DQB1*05:01
HC1803	CONTIG1	A*02:01	B*35:02	C*04:01	DRB1*07:01	DQB1*03:01
	CONTIG2	-	-	C*06:02	DRB1*11:04	DQB1*03:03
HC1804	CONTIG1	A*02:01	B*07:02	C*06:02	DRB1*07:01	DQB1*03:03
	CONTIG2	A*03:01	B*57:01	C*07:02	DRB1*15:01	DQB1*06:02
HC1805	CONTIG1	A*01:01	B*27:05	C*01:02	DRB1*01:01	DQB1*05:01
	CONTIG2	A*02:01	-	C*02:02	-	-
HC1806	CONTIG1	A*02:01	B*13:02	C*03:04	DRB1*07:01	DQB1*02:02
	CONTIG2	-	B*40:01	C*06:02	DRB1*11:01	DQB1*03:01
HC1807	CONTIG1	A*24:02	B*13:02	C*06:02	DRB1*10:01	DQB1*05:01
	CONTIG2	A*68:01	B*51:01	C*14:02	DRB1*13:01	DQB1*06:03
HC1808	CONTIG1	A*02:01	B*27:05	C*02:02	DRB1*09:01	DQB1*03:03
	CONTIG2	A*03:01	B*57:01	C*06:02	DRB1*16:01	DQB1*05:02
HC1809	CONTIG1	A*02:01	B*15:01	C*03:03	DRB1*08:01	DQB1*04:02
	CONTIG2	-	B*40:01	C*03:04	DRB1*13:01	DQB1*06:03
HC1810	CONTIG1	A*02:01	B*18:01	C*05:01	DRB1*13:02	DQB1*05:03
	CONTIG2	-	B*44:02	C*07:01	DRB1*14:54	DQB1*06:04
HC1811	CONTIG1	A*11:01	B*57:01	C*06:02	DRB1*07:01	DQB1*03:03
	CONTIG2	-	-	-	-	-
HC1812	CONTIG1	A*03:01	B*14:02	C*03:04	DRB1*01:02	DQB1*05:01
	CONTIG2	A*33:01	B*15:01	C*08:02	DRB1*15:01	DQB1*06:02
HC1813	CONTIG1	A*02:01	B*07:02	C*02:02	DRB1*11:03	DQB1*03:01
	CONTIG2	A*03:01	B*27:02	C*03:03	DRB1*16:01	DQB1*05:02
HC1814	CONTIG1	A*03:01	B*07:02	C*05:01	DRB1*03:01	DQB1*02:01
	CONTIG2	A*24:02	B*44:02	C*07:02	DRB1*13:01	DQB1*06:03
HC1815	CONTIG1	A*02:01	B*13:02	C*06:02	DRB1*04:01	DQB1*02:02
	CONTIG2	A*24:02	B*44:27	C*07:04	DRB1*07:01	DQB1*03:01
HC1816	CONTIG1	A*01:01	B*18:01	C*12:03	DRB1*12:01	DQB1*03:01
	CONTIG2	A*24:02	B*39:01	-	DRB1*14:54	DQB1*05:03
HC1817	CONTIG1	A*03:01	B*40:01	C*03:04	DRB1*04:07	DQB1*03:01
	CONTIG2	A*24:02	B*51:01	C*04:01	DRB1*13:02	DQB1*06:04
HC1818	CONTIG1	A*01:01	B*07:02	C*07:02	DRB1*15:01	DQB1*06:01
	CONTIG2	A*02:01	B*52:01	C*12:02	DRB1*15:02	DQB1*06:02
HC1819	CONTIG1	A*03:01	B*18:01	C*02:02	DRB1*01:01	DQB1*03:01
	CONTIG2	A*25:01	B*27:02	C*12:03	DRB1*11:01	DQB1*05:01
HC1820	CONTIG1	A*01:01	B*08:01	C*02:02	DRB1*01:01	DQB1*02:01
	CONTIG2	A*25:01	B*27:05	C*07:01	DRB1*03:01	DQB1*05:01
HC1821	CONTIG1	A*02:01	B*07:02	C*04:01	DRB1*14:54	DQB1*05:03
	CONTIG2	A*11:01	B*35:01	C*07:02	DRB1*15:01	DQB1*06:02
HC1822	CONTIG1	A*01:01	B*08:01	C*03:04	DRB1*03:01	DQB1*02:01
	CONTIG2	A*24:02	B*40:01	C*07:01	DRB1*15:01	DQB1*06:02
HC1823	CONTIG1	A*01:01	B*35:02	C*06:02	DRB1*04:02	DQB1*03:02
	CONTIG2	A*26:01	B*38:01	C*12:03	DRB1*07:01	DQB1*03:03
HC1824	CONTIG1	A*03:01	B*07:02	C*07:01	DRB1*12:01	DQB1*03:01
	CONTIG2	A*11:01	B*49:01	C*07:02	DRB1*15:01	DQB1*06:02
HC1825	CONTIG1	A*02:01	B*39:06	C*02:02	DRB1*04:04	DQB1*03:02

	CONTIG2	A*03:01	B*44:05	C*07:02	DRB1*15:01	DQB1*06:02
HC1826	CONTIG1	A*24:02	B*15:01	C*03:03	DRB1*11:01	DQB1*03:01
	CONTIG2	A*25:01	B*18:01	C*12:03	DRB1*13:22	DQB1*04:02
HC1827	CONTIG1	A*02:01	B*15:01	C*03:03	DRB1*03:01	DQB1*02:01
	CONTIG2	-	B*41:01	C*17:01	DRB1*11:03	DQB1*03:01
HC1828	CONTIG1	A*25:01	B*18:01	C*04:01	DRB1*01:01	DQB1*05:01
	CONTIG2	A*68:01	B*35:03	C*12:03	-	-
HC1829	CONTIG1	A*01:01	B*08:01	C*07:01	DRB1*03:01	DQB1*02:01
	CONTIG2	A*24:02	B*18:01	-	DRB1*13:15	DQB1*03:01
HC1830	CONTIG1	A*02:01	B*35:03	C*04:01	DRB1*01:01	DQB1*02:01
	CONTIG2	A*03:01	B*41:01	C*17:01	DRB1*03:01	DQB1*05:01
HC1831	CONTIG1	A*24:02	B*08:01	C*06:02	DRB1*03:01	DQB1*02:01
	CONTIG2	A*31:01	B*57:01	C*07:01	DRB1*11:04	DQB1*03:01
HC1832	CONTIG1	A*02:01	B*13:02	C*06:02	DRB1*03:01	DQB1*02:01
	CONTIG2	A*23:01	B*49:01	C*07:01	DRB1*07:01	DQB1*02:02
HC1833	CONTIG1	A*02:01	B*35:01	C*04:01	DRB1*14:01	DQB1*05:02
	CONTIG2	A*02:17	B*44:27	C*07:04	DRB1*16:01	DQB1*05:03
HC1834	CONTIG1	A*03:01	B*07:02	C*03:04	DRB1*11:01	DQB1*03:01
	CONTIG2	A*24:02	B*40:02	C*07:02	DRB1*15:01	DQB1*06:02
HC1835	CONTIG1	A*26:01	B*18:01	C*07:01	DRB1*11:04	DQB1*03:01
	CONTIG2	A*30:01	B*38:01	C*12:03	DRB1*16:01	DQB1*05:02
HC1836	CONTIG1	A*01:01	B*08:01	C*07:01	DRB1*03:01	DQB1*02:01
	CONTIG2	A*68:01	B*51:01	C*15:13	DRB1*04:04	DQB1*03:02
HC1837	CONTIG1	A*02:01	B*40:02	C*02:02	DRB1*11:03	DQB1*03:01
	CONTIG2	A*32:01	B*57:01	C*06:02	DRB1*16:01	DQB1*05:02
HC1838	CONTIG1	A*02:01	B*07:02	C*07:02	DRB1*04:01	DQB1*03:02
	CONTIG2	A*26:01	-	-	DRB1*15:01	DQB1*06:02
HC1839	CONTIG1	A*24:02	B*27:05	C*01:02	DRB1*01:01	DQB1*05:01
	CONTIG2	A*26:01	B*51:01	C*14:02	DRB1*16:01	DQB1*05:02
HC1840	CONTIG1	A*32:01	B*27:02	C*02:02	DRB1*03:01	DQB1*02:01
	CONTIG2	A*68:01	B*27:05	-	DRB1*11:01	DQB1*03:01
HC1841	CONTIG1	A*02:01	B*13:02	C*03:03	DRB1*04:01	DQB1*02:02
	CONTIG2	A*30:01	B*15:01	C*06:02	DRB1*07:01	DQB1*03:02
HC1842	CONTIG1	A*02:01	B*40:01	C*03:04	DRB1*08:01	DQB1*03:01
	CONTIG2	-	B*41:02	C*17:03	DRB1*13:03	DQB1*04:02
HC1843	CONTIG1	A*03:01	B*18:01	C*04:01	DRB1*01:01	DQB1*03:02
	CONTIG2	A*29:02	B*44:03	C*12:03	DRB1*04:01	DQB1*05:01
HC1844	CONTIG1	A*03:01	B*44:03	C*04:01	DRB1*11:04	DQB1*03:01
	CONTIG2	A*23:01	B*51:01	C*12:03	DRB1*13:02	DQB1*06:09
HC1845	CONTIG1	A*02:01	B*18:01	C*01:02	DRB1*01:01	DQB1*05:01
	CONTIG2	A*03:01	B*56:01	C*07:01	DRB1*16:01	DQB1*05:02
HC1846	CONTIG1	A*33:03	B*35:03	C*03:02	DRB1*04:04	DQB1*03:02
	CONTIG2	A*68:01	B*40:01	C*04:01	DRB1*08:01	DQB1*04:02
HC1847	CONTIG1	A*03:01	B*07:02	C*07:02	DRB1*15:01	DQB1*06:02
	CONTIG2	A*24:02	-	-	-	-
HC1848	CONTIG1	A*24:02	B*35:01	C*01:02	DRB1*07:01	DQB1*03:01
	CONTIG2	A*31:01	B*51:01	C*08:01	DRB1*11:01	DQB1*03:03
HC1849	CONTIG1	A*01:01	B*40:01	C*03:04	DRB1*07:01	DQB1*03:03
	CONTIG2	A*03:01	B*57:01	C*06:02	DRB1*13:01	DQB1*06:03
HC1850	CONTIG1	A*02:01	B*18:01	C*02:02	DRB1*14:54	DQB1*05:02

	CONTIG2	-	B*27:02	C*07:01	DRB1*16:01	DQB1*05:03
HC1851	CONTIG1	A*02:01	B*07:02	C*04:01	DRB1*15:01	DQB1*06:01
	CONTIG2	A*26:01	B*15:01	C*07:02	DRB1*15:02	DQB1*06:02
HC1852	CONTIG1	A*01:01	B*08:01	C*07:01	DRB1*03:01	DQB1*02:01
	CONTIG2	-	-	-	DRB1*15:01	DQB1*06:02
HC1853	CONTIG1	A*02:01	B*40:02	C*02:02	DRB1*11:01	DQB1*03:01
	CONTIG2	A*11:01	B*44:05	-	DRB1*16:01	DQB1*05:02
HC1854	CONTIG1	A*01:01	B*08:01	C*07:01	DRB1*03:01	DQB1*02:01
	CONTIG2	A*02:01	B*51:01	C*14:02	DRB1*08:01	DQB1*04:02
HC1855	CONTIG1	A*01:01	B*08:01	C*02:02	DRB1*03:01	DQB1*02:01
	CONTIG2	A*25:01	B*27:05	C*07:01	DRB1*11:01	DQB1*03:01
HC1856	CONTIG1	A*02:01	B*07:02	C*02:02	DRB1*10:01	DQB1*05:01
	CONTIG2	A*32:01	B*40:02	C*07:02	-	-
HC1857	CONTIG1	A*01:01	B*08:01	C*05:01	DRB1*01:01	DQB1*05:01
	CONTIG2	A*26:01	B*44:02	C*07:01	DRB1*16:01	DQB1*05:02
HC1858	CONTIG1	A*01:01	B*18:01	C*12:02	DRB1*03:01	DQB1*02:01
	CONTIG2	A*25:01	B*52:01	C*12:03	DRB1*07:01	DQB1*02:02
HC1859	CONTIG1	A*23:01	B*44:02	C*07:01	DRB1*11:01	DQB1*03:01
	CONTIG2	A*30:01	B*49:01	C*07:04	DRB1*15:02	DQB1*06:01
HC1860	CONTIG1	A*02:01	B*07:02	C*06:02	DRB1*07:01	DQB1*03:03
	CONTIG2	A*68:01	B*57:01	C*07:02	DRB1*13:02	DQB1*06:04
HC1861	CONTIG1	A*01:01	B*08:01	C*07:01	DRB1*03:01	DQB1*02:01
	CONTIG2	A*02:01	B*38:01	C*12:03	DRB1*15:02	DQB1*06:01
HC1862	CONTIG1	A*02:01	B*49:01	C*01:02	DRB1*03:01	DQB1*02:01
	CONTIG2	A*03:01	B*51:01	C*07:01	DRB1*15:01	DQB1*06:02
HC1863	CONTIG1	A*02:01	B*51:01	C*12:02	DRB1*01:01	DQB1*05:01
	CONTIG2	A*11:01	B*52:01	C*14:02	DRB1*15:02	DQB1*06:01
HC1864	CONTIG1	A*01:01	B*40:01	C*03:04	DRB1*08:01	DQB1*05:02
	CONTIG2	A*02:01	B*44:27	C*07:04	DRB1*16:01	DQB1*06:02
HC1865	CONTIG1	A*01:01	B*07:02	C*07:01	DRB1*01:01	DQB1*02:01
	CONTIG2	-	B*08:01	C*07:02	DRB1*03:01	DQB1*05:01
HC1866	CONTIG1	A*02:01	B*18:01	C*04:01	DRB1*07:01	DQB1*02:02
	CONTIG2	A*24:02	B*44:03	C*07:01	DRB1*16:01	DQB1*05:02
HC1867	CONTIG1	A*01:01	B*08:01	C*01:02	DRB1*01:01	DQB1*03:01
	CONTIG2	-	B*27:05	C*07:01	DRB1*11:01	DQB1*05:01
HC1868	CONTIG1	A*02:01	B*13:02	C*02:02	DRB1*01:01	DQB1*02:02
	CONTIG2	A*24:02	B*27:05	C*06:02	DRB1*07:01	DQB1*05:01
HC1869	CONTIG1	A*11:01	B*07:02	C*04:01	DRB1*08:01	DQB1*04:02
	CONTIG2	A*24:02	B*35:02	C*07:02	DRB1*15:01	DQB1*06:02
HC1870	CONTIG1	A*01:01	B*08:01	C*06:02	DRB1*03:01	DQB1*02:01
	CONTIG2	A*02:01	B*57:01	C*07:01	DRB1*07:01	DQB1*03:03
HC1871	CONTIG1	A*01:01	B*07:02	C*07:01	DRB1*03:01	DQB1*02:01
	CONTIG2	A*24:02	B*08:01	C*07:02	DRB1*15:01	DQB1*06:02
HC1872	CONTIG1	A*11:01	B*35:01	C*04:01	DRB1*11:01	DQB1*03:01
	CONTIG2	A*24:02	B*44:03	-	DRB1*11:04	-
HC1873	CONTIG1	A*01:01	B*08:01	C*07:01	DRB1*13:01	DQB1*06:02
	CONTIG2	A*25:01	B*18:01	C*12:03	DRB1*15:01	DQB1*06:03
HC1874	CONTIG1	A*03:01	B*27:05	C*02:02	DRB1*01:01	DQB1*05:01
	CONTIG2	A*24:02	B*35:01	C*04:01	-	-
HC1875	CONTIG1	A*02:01	B*27:05	C*02:02	DRB1*01:01	DQB1*03:03

	CONTIG2	A*25:01	B*57:01	C*06:02	DRB1*07:01	DQB1*05:01
HC1876	CONTIG1	A*01:01	B*08:01	C*07:01	DRB1*03:01	DQB1*02:01
	CONTIG2	-	-	-	DRB1*13:01	DQB1*06:03
HC1877	CONTIG1	A*01:01	B*08:01	C*04:01	DRB1*01:01	DQB1*02:01
	CONTIG2	A*03:01	B*35:01	C*07:01	DRB1*03:01	DQB1*05:01
HC1878	CONTIG1	A*02:01	B*15:01	C*01:02	DRB1*09:01	DQB1*03:01
	CONTIG2	-	B*40:02	C*02:02	DRB1*11:01	DQB1*03:03
HC1879	CONTIG1	A*01:01	B*08:01	C*06:02	DRB1*03:01	DQB1*02:01
	CONTIG2	A*23:01	B*57:01	C*07:01	DRB1*07:01	DQB1*03:03
HC1880	CONTIG1	A*03:01	B*07:02	C*03:03	DRB1*11:03	DQB1*03:01
	CONTIG2	A*11:01	B*15:01	C*07:02	DRB1*15:01	DQB1*06:02
HC1881	CONTIG1	A*02:01	B*27:05	C*02:02	DRB1*01:01	DQB1*05:01
	CONTIG2	A*25:01	B*51:01	-	DRB1*13:02	DQB1*06:04
HC1882	CONTIG1	A*32:01	B*15:01	C*02:02	DRB1*11:01	DQB1*03:01
	CONTIG2	A*68:01	B*40:02	C*03:03	DRB1*13:01	DQB1*06:03
HC1883	CONTIG1	A*02:01	B*15:01	C*03:04	DRB1*01:01	DQB1*03:02
	CONTIG2	A*68:01	B*35:03	C*04:01	DRB1*04:01	DQB1*05:01
HC1884	CONTIG1	A*26:01	B*07:02	C*07:02	DRB1*13:01	DQB1*06:02
	CONTIG2	A*31:01	B*38:01	C*12:03	DRB1*15:01	DQB1*06:03
HC1885	CONTIG1	A*11:01	B*35:01	C*01:02	DRB1*01:01	DQB1*02:02
	CONTIG2	-	B*56:01	C*04:01	DRB1*07:01	DQB1*05:01
HC1886	CONTIG1	A*23:01	B*40:02	C*02:02	DRB1*11:01	DQB1*03:01
	CONTIG2	A*32:01	B*49:01	C*07:01	-	-
HC1887	CONTIG1	A*24:02	B*37:01	C*12:03	DRB1*07:01	DQB1*02:02
	CONTIG2	A*25:01	B*38:01	-	DRB1*13:01	DQB1*06:03
HC1888	CONTIG1	A*03:01	B*07:02	C*03:04	DRB1*07:01	DQB1*03:01
	CONTIG2	A*26:01	B*40:02	C*07:02	DRB1*11:01	DQB1*03:03
HC1889	CONTIG1	A*02:01	B*40:01	C*03:04	DRB1*11:01	DQB1*03:01
	CONTIG2	A*11:01	B*51:01	C*15:02	DRB1*15:01	DQB1*06:02
HC1890	CONTIG1	A*01:01	B*08:01	C*02:02	DRB1*03:01	DQB1*02:01
	CONTIG2	A*02:01	B*27:02	C*07:01	DRB1*16:01	DQB1*05:02
HC1891	CONTIG1	A*02:01	B*44:02	C*05:01	DRB1*07:01	DQB1*02:02
	CONTIG2	-	-	-	DRB1*11:04	DQB1*03:01
HC1892	CONTIG1	A*01:01	B*08:01	C*07:01	DRB1*03:01	DQB1*02:01
	CONTIG2	A*30:04	B*57:03	C*07:01	DRB1*13:01	DQB1*06:03
HC1893	CONTIG1	A*01:01	B*07:02	C*07:01	DRB1*03:01	DQB1*02:01
	CONTIG2	A*03:01	B*08:01	C*07:02	DRB1*15:01	DQB1*06:02
HC1894	CONTIG1	A*02:01	B*07:02	C*06:02	DRB1*07:01	DQB1*02:02
	CONTIG2	A*24:02	B*13:02	C*07:02	DRB1*15:01	DQB1*06:02
HC1895	CONTIG1	A*25:01	B*13:02	C*04:01	DRB1*01:01	DQB1*02:02
	CONTIG2	A*30:01	B*35:01	C*06:02	DRB1*07:01	DQB1*05:01
HC1896	CONTIG1	A*01:01	B*08:01	C*07:01	DRB1*01:01	DQB1*02:01
	CONTIG2	A*02:01	B*39:01	C*12:03	DRB1*03:01	DQB1*05:01
HC1897	CONTIG1	A*02:01	B*08:01	C*04:01	DRB1*03:01	DQB1*02:01
	CONTIG2	-	B*44:03	C*07:01	DRB1*07:01	DQB1*02:02
HC1898	CONTIG1	A*02:01	B*07:02	C*07:02	DRB1*07:01	DQB1*02:02
	CONTIG2	-	B*41:02	C*17:03	DRB1*13:03	DQB1*03:01
HC1899	CONTIG1	A*03:01	B*07:02	C*04:01	DRB1*03:01	DQB1*02:01
	CONTIG2	A*25:01	B*35:03	C*07:02	DRB1*16:01	DQB1*05:02
HC1900	CONTIG1	A*03:01	B*27:05	C*06:02	DRB1*04:01	DQB1*03:02

	CONTIG2	A*11:01	B*52:01	C*12:02	DRB1*15:02	DQB1*06:01
HC1901	CONTIG1	A*03:01	B*07:02	C*06:02	DRB1*13:01	DQB1*06:03
	CONTIG2	A*11:01	B*44:02	C*07:02	-	-
HC1902	CONTIG1	A*02:01	B*07:02	C*07:02	DRB1*12:01	DQB1*03:01
	CONTIG2	A*31:01	B*39:01	C*12:03	DRB1*15:01	DQB1*06:02
HC1903	CONTIG1	A*01:01	B*56:01	C*01:02	DRB1*07:01	DQB1*03:03
	CONTIG2	A*26:01	B*57:01	C*06:02	DRB1*14:54	DQB1*05:03
HC1904	CONTIG1	A*01:01	B*08:01	C*07:01	DRB1*03:01	DQB1*02:01
	CONTIG2	A*25:01	B*18:01	C*12:03	DRB1*07:01	DQB1*02:02
HC1905	CONTIG1	A*02:01	B*27:02	C*02:02	DRB1*15:01	DQB1*05:02
	CONTIG2	-	B*38:01	C*12:03	DRB1*16:01	DQB1*06:02
HC1906	CONTIG1	A*02:01	B*15:01	C*03:04	DRB1*04:01	DQB1*03:02
	CONTIG2	-	B*51:01	C*14:02	DRB1*09:01	DQB1*03:03
HC1907	CONTIG1	A*30:01	B*13:02	C*03:04	DRB1*07:01	DQB1*02:02
	CONTIG2	A*33:03	B*15:01	C*06:02	DRB1*13:01	DQB1*06:03
HC1908	CONTIG1	A*02:01	B*18:03	C*01:02	DRB1*04:03	DQB1*03:02
	CONTIG2	A*03:01	B*51:01	C*07:01	DRB1*14:54	DQB1*05:03
HC1909	CONTIG1	A*11:01	B*07:02	C*07:02	DRB1*12:01	DQB1*03:01
	CONTIG2	A*33:01	B*39:01	C*12:03	DRB1*15:01	DQB1*06:02
HC1910	CONTIG1	A*24:02	B*27:05	C*01:02	DRB1*01:01	DQB1*05:01
	CONTIG2	A*25:01	B*35:01	C*04:01	-	-
HC1911	CONTIG1	A*24:02	B*07:02	C*07:02	DRB1*13:01	DQB1*05:02
	CONTIG2	A*26:01	B*38:01	C*12:03	DRB1*15:01	DQB1*06:03
HC1912	CONTIG1	A*02:01	B*38:01	C*02:02	DRB1*04:02	DQB1*03:01
	CONTIG2	A*26:01	B*40:02	C*12:03	DRB1*11:01	DQB1*03:02
HC1913	CONTIG1	A*02:01	B*14:02	C*01:02	DRB1*01:02	DQB1*03:01
	CONTIG2	A*03:01	B*51:01	C*08:02	DRB1*11:01	DQB1*05:01
HC1914	CONTIG1	A*26:01	B*13:02	C*06:02	DRB1*11:01	DQB1*03:01
	CONTIG2	A*30:01	B*38:01	C*12:03	DRB1*15:01	DQB1*06:02
HC1915	CONTIG1	A*30:01	B*13:02	C*06:02	DRB1*11:01	DQB1*03:01
	CONTIG2	A*32:01	B*49:01	C*07:01	DRB1*13:01	DQB1*06:03
HC1916	CONTIG1	A*02:01	B*07:02	C*04:01	DRB1*01:01	DQB1*04:02
	CONTIG2	A*03:01	B*15:01	C*07:02	DRB1*08:01	DQB1*05:01
HC1917	CONTIG1	A*23:01	B*07:02	C*07:01	DRB1*01:01	DQB1*02:01
	CONTIG2	A*24:02	B*08:01	C*07:02	DRB1*03:01	DQB1*05:01
HC1918	CONTIG1	A*03:01	B*07:02	C*04:01	DRB1*07:01	DQB1*03:01
	CONTIG2	A*11:01	B*35:01	C*07:02	DRB1*12:01	DQB1*03:03
HC1919	CONTIG1	A*01:01	B*08:01	C*06:02	DRB1*03:01	DQB1*02:01
	CONTIG2	A*02:01	B*13:02	C*07:01	DRB1*07:01	DQB1*02:02
HC1920	CONTIG1	A*11:01	B*35:02	C*04:01	DRB1*01:01	DQB1*03:01
	CONTIG2	A*24:02	B*52:01	C*12:02	DRB1*11:04	DQB1*05:01
HC1921	CONTIG1	A*02:01	B*13:02	C*05:01	DRB1*07:01	DQB1*02:02
	CONTIG2	A*11:01	B*44:02	C*06:02	DRB1*12:01	DQB1*03:01
HC1922	CONTIG1	A*25:01	B*27:02	C*02:02	DRB1*09:01	DQB1*03:03
	CONTIG2	A*32:01	B*27:05	-	DRB1*16:01	DQB1*05:02
HC1923	CONTIG1	A*02:01	B*18:01	C*02:02	DRB1*11:04	DQB1*03:01
	CONTIG2	-	B*44:05	C*12:03	DRB1*16:01	DQB1*05:02
HC1924	CONTIG1	A*02:01	B*44:02	C*01:02	DRB1*01:01	DQB1*04:02
	CONTIG2	A*03:01	B*51:01	C*05:01	DRB1*08:01	DQB1*05:01
HC1925	CONTIG1	A*01:01	B*13:02	C*03:04	DRB1*04:01	DQB1*02:02

	CONTIG2	A*02:05	B*15:01	C*06:02	DRB1*07:01	DQB1*03:02
HC1926	CONTIG1	A*02:01	B*07:05	C*05:01	DRB1*10:01	DQB1*03:01
	CONTIG2	A*30:01	B*44:02	C*15:05	DRB1*11:04	DQB1*05:01
HC1927	CONTIG1	A*02:01	B*38:01	C*02:02	DRB1*11:01	DQB1*03:01
	CONTIG2	A*26:01	B*44:02	C*12:03	DRB1*13:01	DQB1*06:03
HC1928	CONTIG1	A*02:01	B*35:01	C*04:01	DRB1*01:01	DQB1*05:01
	CONTIG2	A*26:01	B*52:01	C*12:02	DRB1*14:54	DQB1*05:03
HC1929	CONTIG1	A*03:01	B*07:02	C*07:01	DRB1*13:01	DQB1*06:02
	CONTIG2	A*68:01	B*58:01	C*07:02	DRB1*15:01	DQB1*06:03
HC1930	CONTIG1	A*24:02	B*18:01	C*06:02	DRB1*03:01	DQB1*02:01
	CONTIG2	A*25:01	B*50:01	C*12:03	DRB1*13:01	DQB1*06:03
HC1931	CONTIG1	A*02:01	B*15:01	C*02:02	DRB1*04:01	DQB1*02:02
	CONTIG2	-	B*27:05	C*03:04	DRB1*07:01	DQB1*03:02
HC1932	CONTIG1	A*11:01	B*18:01	C*12:03	DRB1*04:01	DQB1*03:02
	CONTIG2	A*25:01	B*51:01	C*15:02	DRB1*04:04	-
HC1933	CONTIG1	A*02:01	B*08:01	C*02:02	DRB1*01:01	DQB1*02:01
	CONTIG2	A*31:01	B*27:05	C*07:01	DRB1*03:01	DQB1*05:01
HC1934	CONTIG1	A*01:01	B*27:05	C*01:02	DRB1*01:01	DQB1*03:01
	CONTIG2	A*68:01	B*44:02	C*07:04	DRB1*11:01	DQB1*05:01
HC1935	CONTIG1	A*03:01	B*15:01	C*03:03	DRB1*11:01	DQB1*03:01
	CONTIG2	A*24:02	B*44:03	C*04:01	DRB1*13:01	DQB1*06:03
HC1936	CONTIG1	A*01:01	B*08:01	C*07:01	DRB1*03:01	DQB1*02:01
	CONTIG2	-	B*52:01	C*12:02	DRB1*15:02	DQB1*06:01
HC1937	CONTIG1	A*23:01	B*07:02	C*03:03	DRB1*01:01	DQB1*03:01
	CONTIG2	A*26:01	B*55:01	C*12:03	DRB1*11:03	DQB1*05:01
HC1938	CONTIG1	A*02:01	B*15:01	C*02:02	DRB1*13:01	DQB1*05:02
	CONTIG2	A*26:01	B*27:02	C*03:03	DRB1*16:01	DQB1*06:03
HC1939	CONTIG1	A*03:01	B*07:02	C*07:02	DRB1*13:01	DQB1*06:02
	CONTIG2	A*68:01	B*51:01	C*14:02	DRB1*15:01	DQB1*06:03
HC1940	CONTIG1	A*02:01	B*07:02	C*05:01	DRB1*15:01	DQB1*06:02
	CONTIG2	A*26:01	B*44:02	C*07:02	-	-
HC1941	CONTIG1	A*01:01	B*08:01	C*07:01	DRB1*03:01	DQB1*05:03
	CONTIG2	A*66:01	B*41:02	C*17:03	DRB1*14:54	DQB1*06:02
HC1942	CONTIG1	A*03:01	B*35:01	C*04:01	DRB1*01:01	DQB1*03:01
	CONTIG2	A*68:01	B*44:02	C*07:04	DRB1*11:01	DQB1*05:01
HC1943	CONTIG1	A*11:01	B*35:01	C*03:04	DRB1*11:01	DQB1*03:01
	CONTIG2	A*24:02	B*40:01	C*04:01	-	-
HC1944	CONTIG1	A*01:01	B*08:01	C*07:01	DRB1*11:01	DQB1*03:01
	CONTIG2	A*26:01	B*38:01	C*12:03	DRB1*13:01	DQB1*06:03
HC1945	CONTIG1	A*03:01	B*35:03	C*04:01	DRB1*07:01	DQB1*02:02
	CONTIG2	A*29:02	B*44:03	C*16:01	DRB1*08:01	DQB1*04:02
HC1946	CONTIG1	A*02:01	B*41:01	C*06:02	DRB1*07:01	DQB1*02:02
	CONTIG2	A*02:06	B*57:01	C*17:01	-	DQB1*03:03
HC1947	CONTIG1	A*02:01	B*07:02	C*07:02	DRB1*04:08i	DQB1*03:04
	CONTIG2	A*11:01	B*35:03	C*12:03	DRB1*15:01	DQB1*06:02
HC1948	CONTIG1	A*01:01	B*07:04	C*07:02	DRB1*15:01	DQB1*05:02
	CONTIG2	A*02:01	B*44:27	C*07:04	DRB1*16:01	DQB1*06:02
HC1949	CONTIG1	A*24:02	B*15:01	C*02:02	DRB1*11:01	DQB1*03:01
	CONTIG2	A*31:01	B*40:02	C*03:04	-	-
HC1950	CONTIG1	A*02:01	B*18:01	C*07:01	DRB1*11:04	DQB1*03:01

	CONTIG2	-	B*44:27	C*07:04	DRB1*16:01	DQB1*05:02
HC1951	CONTIG1	A*02:01	B*07:02	C*03:04	DRB1*08:01	DQB1*04:02
	CONTIG2	A*03:01	B*15:01	C*07:02	DRB1*15:01	DQB1*06:02
HC1952	CONTIG1	A*02:01	B*18:01	C*01:02	DRB1*01:01	DQB1*03:01
	CONTIG2	A*26:01	B*27:05	C*12:03	DRB1*11:01	DQB1*05:01
HC1953	CONTIG1	A*03:02	B*27:05	C*02:02	DRB1*13:01	DQB1*05:02
	CONTIG2	A*26:01	B*38:01	C*12:03	DRB1*16:01	DQB1*06:03
HC1954	CONTIG1	A*02:01	B*49:01	C*06:02	DRB1*07:01	DQB1*02:02
	CONTIG2	-	B*57:01	C*07:01	-	DQB1*03:03
HC1955	CONTIG1	A*02:01	B*07:02	C*04:01	DRB1*11:01	DQB1*03:01
	CONTIG2	A*03:01	B*35:03	C*07:02	DRB1*12:01	-
HC1956	CONTIG1	A*02:01	B*07:02	C*02:02	DRB1*11:01	DQB1*03:01
	CONTIG2	A*03:01	B*27:02	C*07:02	DRB1*15:01	DQB1*06:02
HC1957	CONTIG1	A*03:01	B*07:02	C*05:01	DRB1*11:01	DQB1*03:01
	CONTIG2	-	B*35:01	C*07:02	DRB1*13:01	DQB1*06:03
HC1958	CONTIG1	A*01:01	B*37:01	C*04:01	DRB1*07:01	DQB1*02:02
	CONTIG2	A*23:01	B*44:03	C*06:02	DRB1*16:01	DQB1*05:02
HC1959	CONTIG1	A*02:01	B*41:01	C*07:01	DRB1*01:01	DQB1*03:01
	CONTIG2	A*24:02	B*49:01	-	DRB1*08:04	DQB1*05:04
HC1960	CONTIG1	A*01:01	B*08:01	C*04:01	DRB1*03:01	DQB1*02:01
	CONTIG2	A*32:01	B*35:08	C*07:01	DRB1*15:01	DQB1*05:01
HC1961	CONTIG1	A*01:01	B*08:01	C*04:01	DRB1*01:01	DQB1*02:01
	CONTIG2	A*03:01	B*35:01	C*07:01	DRB1*03:01	DQB1*05:01
HC1962	CONTIG1	A*02:01	B*13:02	C*04:01	DRB1*01:01	DQB1*05:01
	CONTIG2	-	B*35:01	C*06:02	DRB1*15:01	DQB1*06:02
HC1963	CONTIG1	A*01:01	B*35:01	C*04:01	DRB1*11:04	DQB1*03:01
	CONTIG2	A*02:01	B*35:02	-	DRB1*13:05	-
HC1964	CONTIG1	A*01:01	B*38:01	C*03:04	DRB1*04:04	DQB1*03:02
	CONTIG2	A*68:01	B*40:01	C*12:03	DRB1*04:05	-
HC1965	CONTIG1	A*24:02	B*07:02	C*07:02	DRB1*04:01	DQB1*03:02
	CONTIG2	A*25:01	B*15:01	C*12:03	DRB1*15:01	DQB1*06:02
HC1966	CONTIG1	A*01:01	B*08:01	C*04:01	DRB1*03:01	DQB1*02:01
	CONTIG2	A*30:04	B*35:02	C*07:01	DRB1*11:04	DQB1*03:01
HC1967	CONTIG1	A*23:01	B*44:03	C*04:01	DRB1*07:01	DQB1*02:02
	CONTIG2	-	-	-	DRB1*11:04	DQB1*03:01
HC1968	CONTIG1	A*03:01	B*18:01	C*02:02	DRB1*11:04	DQB1*03:01
	CONTIG2	A*11:01	B*27:05	C*07:01	DRB1*16:01	DQB1*05:02
HC1969	CONTIG1	A*01:01	B*27:05	C*02:02	DRB1*01:01	DQB1*02:01
	CONTIG2	A*02:01	B*41:01	C*17:01	DRB1*03:01	DQB1*05:01
HC1970	CONTIG1	A*25:01	B*07:02	C*07:02	DRB1*01:01	DQB1*05:01
	CONTIG2	A*26:01	B*18:01	C*12:03	DRB1*15:01	DQB1*06:02
HC1971	CONTIG1	A*31:01	B*14:02	C*02:02	DRB1*01:02	DQB1*03:01
	CONTIG2	A*34:02	B*40:02	C*08:02	DRB1*11:01	DQB1*05:01
HC1972	CONTIG1	A*02:01	B*15:01	C*04:01	DRB1*07:01	DQB1*03:01
	CONTIG2	A*03:01	B*35:01	C*05:01	DRB1*11:01	DQB1*03:03
HC1973	CONTIG1	A*24:02	B*18:01	C*03:16	DRB1*04:01	DQB1*03:02
	CONTIG2	A*25:01	B*58:01	C*12:03	DRB1*13:02	DQB1*06:09
HC1974	CONTIG1	A*02:01	B*07:02	C*02:02	DRB1*11:01	DQB1*03:01
	CONTIG2	A*24:02	B*40:02	C*07:02	DRB1*15:01	DQB1*06:02
HC1975	CONTIG1	A*24:02	B*15:01	C*03:03	DRB1*13:01	DQB1*05:03

	CONTIG2	A*32:01	B*35:01	C*04:01	DRB1*14:01	DQB1*06:03
HC1976	CONTIG1	A*01:01	B*15:01	C*03:03	DRB1*07:01	DQB1*03:01
	CONTIG2	A*24:02	B*35:02	C*06:02	DRB1*11:03	DQB1*03:03
HC1977	CONTIG1	A*02:01	B*07:04	C*07:01	DRB1*13:01	DQB1*06:02
	CONTIG2	-	B*18:01	C*07:02	DRB1*15:01	DQB1*06:03
HC1978	CONTIG1	A*66:01	B*18:01	C*05:01	DRB1*03:01	DQB1*02:01
	CONTIG2	A*68:01	B*41:02	C*17:03	DRB1*13:03	DQB1*03:01
HC1979	CONTIG1	A*02:01	B*07:02	C*07:02	DRB1*11:01	DQB1*03:01
	CONTIG2	A*03:01	B*39:01	C*12:03	DRB1*15:01	DQB1*06:02
HC1980	CONTIG1	A*03:01	B*27:05	C*02:02	DRB1*07:01	DQB1*02:02
	CONTIG2	A*24:02	B*47:01	C*06:02	DRB1*13:01	DQB1*06:03
HC1981	CONTIG1	A*02:01	B*14:02	C*04:01	DRB1*13:02	DQB1*03:01
	CONTIG2	A*68:02	B*50:01	C*08:02	DRB1*13:03	DQB1*06:04
HC1982	CONTIG1	A*02:01	B*40:01	C*03:04	DRB1*15:01	DQB1*05:02
	CONTIG2	A*03:01	B*44:27	C*07:04	DRB1*16:01	DQB1*06:02
HC1983	CONTIG1	A*01:01	B*13:02	C*01:02	DRB1*07:01	DQB1*02:02
	CONTIG2	A*02:01	B*51:01	C*06:02	DRB1*11:01	DQB1*03:01
HC1984	CONTIG1	A*02:05	B*35:01	C*04:01	DRB1*01:01	DQB1*03:01
	CONTIG2	A*11:01	B*41:01	C*07:01	DRB1*08:04	DQB1*05:01
HC1985	CONTIG1	A*02:01	B*40:01	C*03:04	DRB1*15:01	DQB1*05:02
	CONTIG2	A*23:01	B*49:01	C*07:01	DRB1*16:01	DQB1*06:02
HC1986	CONTIG1	A*01:01	B*13:02	C*06:02	DRB1*07:01	DQB1*02:02
	CONTIG2	A*30:01	B*15:17	C*07:01	DRB1*13:02	DQB1*06:04
HC1987	CONTIG1	A*02:01	B*41:02	C*06:02	DRB1*04:05	DQB1*03:01
	CONTIG2	A*31:01	B*47:01	C*17:03	DRB1*13:03	DQB1*03:02
HC1988	CONTIG1	A*11:01	B*27:05	C*02:02	DRB1*11:01	DQB1*03:01
	CONTIG2	A*23:01	B*49:01	C*07:01	DRB1*16:01	DQB1*05:02
HC1989	CONTIG1	A*01:01	B*08:01	C*06:02	DRB1*03:01	DQB1*02:01
	CONTIG2	A*68:01	B*57:01	C*07:01	DRB1*07:01	DQB1*03:03
HC1990	CONTIG1	A*01:01	B*08:01	C*06:02	DRB1*03:01	DQB1*02:01
	CONTIG2	A*02:01	B*13:02	C*07:01	DRB1*07:01	DQB1*02:02
HC1991	CONTIG1	A*02:01	B*07:02	C*05:01	DRB1*07:01	DQB1*02:02
	CONTIG2	A*31:01	B*44:02	C*07:02	DRB1*15:01	DQB1*06:02
HC1992	CONTIG1	A*01:01	B*41:02	C*12:03	DRB1*11:04	DQB1*03:01
	CONTIG2	A*02:01	B*51:01	C*17:03	DRB1*13:03	-
HC1993	CONTIG1	A*01:01	B*52:01	C*06:02	DRB1*11:01	DQB1*03:01
	CONTIG2	A*25:01	B*57:01	C*12:02	DRB1*15:02	DQB1*06:01
HC1994	CONTIG1	A*01:01	B*08:01	C*07:01	DRB1*03:01	DQB1*02:01
	CONTIG2	A*26:01	B*38:01	C*12:03	DRB1*13:01	DQB1*06:03
HC1995	CONTIG1	A*30:01	B*07:05	C*03:04	DRB1*07:01	DQB1*03:03
	CONTIG2	A*32:01	B*40:01	C*15:05	DRB1*10:01	DQB1*05:01
HC1996	CONTIG1	A*02:01	B*07:02	C*02:02	DRB1*03:01	DQB1*02:01
	CONTIG2	A*11:01	B*40:02	C*07:02	DRB1*15:01	DQB1*06:02
HC1997	CONTIG1	A*02:01	B*44:02	C*05:01	DRB1*11:01	DQB1*03:01
	CONTIG2	A*68:01	B*51:01	C*15:02	DRB1*12:01	-
HC1998	CONTIG1	A*01:01	B*08:01	C*07:01	DRB1*03:01	DQB1*02:01
	CONTIG2	A*68:01	B*51:01	C*07:02	DRB1*11:01	DQB1*03:01
HC1999	CONTIG1	A*02:05	B*18:01	C*06:02	DRB1*07:01	DQB1*02:02
	CONTIG2	A*25:01	B*50:01	C*12:03	DRB1*15:01	DQB1*06:02
HC2000	CONTIG1	A*02:01	B*35:02	C*04:01	DRB1*11:01	DQB1*03:01

	CONTIG2	-	B*49:01	C*07:01	DRB1*11:04	-
HC2001	CONTIG1	A*02:01	B*07:02	C*07:02	DRB1*07:01	DQB1*02:02
	CONTIG2	A*03:01	B*44:03	C*16:01	DRB1*15:01	DQB1*06:02
HC2002	CONTIG1	A*02:01	B*18:01	C*12:03	DRB1*04:07	DQB1*03:01
	CONTIG2	A*30:01	B*38:01	-	DRB1*13:01	DQB1*06:03
HC2003	CONTIG1	A*01:01	B*08:01	C*07:01	DRB1*03:01	DQB1*02:01
	CONTIG2	-	-	-	-	-
HC2004	CONTIG1	A*11:01	B*14:02	C*04:01	DRB1*07:01	DQB1*02:02
	CONTIG2	A*24:02	B*35:02	C*08:02	DRB1*11:04	DQB1*03:01
HC2005	CONTIG1	A*24:02	B*13:02	C*05:01	DRB1*07:01	DQB1*02:02
	CONTIG2	A*30:01	B*44:02	C*06:02	DRB1*11:01	DQB1*03:01
HC2006	CONTIG1	A*02:01	B*35:01	C*03:03	DRB1*01:01	DQB1*05:01
	CONTIG2	A*32:01	B*40:01	C*03:04	DRB1*13:02	DQB1*06:04
HC2007	CONTIG1	A*23:01	B*35:02	C*04:01	DRB1*07:01	DQB1*02:02
	CONTIG2	A*24:02	B*44:03	-	DRB1*11:04	DQB1*03:01
HC2008	CONTIG1	A*01:01	B*35:01	C*03:04	DRB1*01:01	DQB1*04:02
	CONTIG2	A*03:01	B*40:01	C*04:01	DRB1*08:01	DQB1*05:01
HC2009	CONTIG1	A*01:01	B*55:01	C*03:03	DRB1*13:01	DQB1*05:03
	CONTIG2	A*11:01	B*57:01	C*06:02	DRB1*14:54	DQB1*06:03
HC2010	CONTIG1	A*11:01	B*38:01	C*03:04	DRB1*08:01	DQB1*03:01
	CONTIG2	A*24:02	B*40:01	C*12:03	-	DQB1*04:02
HC2011	CONTIG1	A*03:01	B*15:01	C*03:04	DRB1*04:04	DQB1*03:02
	CONTIG2	A*23:01	B*44:03	C*04:01	DRB1*08:01	DQB1*04:02
HC2012	CONTIG1	A*03:01	B*07:02	C*07:02	DRB1*15:01	DQB1*05:02
	CONTIG2	A*26:01	B*51:01	C*16:02	DRB1*16:01	DQB1*06:02
HC2013	CONTIG1	A*02:01	B*07:02	C*07:02	DRB1*07:01	DQB1*02:02
	CONTIG2	A*03:01	B*44:03	C*16:01	DRB1*15:01	DQB1*06:02
HC2014	CONTIG1	A*02:01	B*18:01	C*04:01	DRB1*04:01	DQB1*03:02
	CONTIG2	A*25:01	B*35:01	C*12:03	DRB1*14:01	DQB1*05:03
HC2015	CONTIG1	A*01:01	B*08:01	C*07:01	DRB1*11:01	DQB1*03:01
	CONTIG2	A*26:01	B*38:01	C*12:03	DRB1*13:01	DQB1*06:03
HC2016	CONTIG1	A*01:01	B*07:02	C*07:01	DRB1*11:04	DQB1*03:01
	CONTIG2	A*68:01	B*18:01	C*07:02	DRB1*15:01	DQB1*06:02
HC2017	CONTIG1	A*02:01	B*08:01	C*02:02	DRB1*01:01	DQB1*02:01
	CONTIG2	A*03:01	B*27:05	C*07:01	DRB1*03:01	DQB1*05:01
HC2018	CONTIG1	A*03:01	B*15:18	C*04:01	DRB1*01:01	DQB1*03:01
	CONTIG2	A*11:01	B*35:01	C*07:04	DRB1*13:03	DQB1*05:01
HC2019	CONTIG1	A*02:01	B*40:02	C*02:02	DRB1*03:01	DQB1*02:01
	CONTIG2	-	B*41:01	C*17:01	DRB1*13:01	DQB1*06:03
HC2020	CONTIG1	A*01:01	B*08:01	C*02:02	DRB1*03:01	DQB1*02:01
	CONTIG2	A*24:02	B*27:05	C*07:01	DRB1*16:01	DQB1*05:02
HC2021	CONTIG1	A*11:01	B*40:01	C*01:02	DRB1*08:01	DQB1*03:01
	CONTIG2	A*68:01	B*56:01	C*03:04	-	DQB1*04:02
HC2022	CONTIG1	A*03:01	B*35:01	C*04:01	DRB1*04:01	DQB1*02:02
	CONTIG2	A*31:01	B*39:01	C*12:03	DRB1*07:01	DQB1*03:02
HC2023	CONTIG1	A*02:01	B*18:01	C*07:01	DRB1*07:01	DQB1*02:02
	CONTIG2	A*24:02	B*35:08	C*12:03	DRB1*11:04	DQB1*03:01
HC2024	CONTIG1	A*02:01	B*15:18	C*01:02	DRB1*01:03	DQB1*03:02
	CONTIG2	A*11:01	B*56:01	C*07:04	DRB1*04:01	DQB1*05:01
HC2025	CONTIG1	A*02:01	B*44:02	C*03:03	DRB1*04:01	DQB1*03:01

	CONTIG2	A*11:01	B*55:01	C*05:01	DRB1*16:01	DQB1*05:02
HC2026	CONTIG1	A*29:02	B*07:02	C*07:02	DRB1*07:01	DQB1*02:02
	CONTIG2	A*31:01	B*44:03	C*16:01	DRB1*13:02	DQB1*06:04
HC2027	CONTIG1	A*01:01	B*38:01	C*07:04	DRB1*13:01	DQB1*05:02
	CONTIG2	A*26:01	B*44:27	C*12:03	DRB1*16:01	DQB1*06:03
HC2028	CONTIG1	A*11:01	B*07:02	C*04:01	DRB1*03:01	DQB1*02:01
	CONTIG2	A*32:01	B*35:01	C*07:02	DRB1*11:01	DQB1*03:01
HC2029	CONTIG1	A*03:01	B*07:02	C*07:02	DRB1*11:04	DQB1*03:01
	CONTIG2	A*68:02	B*14:02	C*08:02	DRB1*13:03	-
HC2030	CONTIG1	A*02:01	B*08:01	C*01:02	DRB1*03:01	DQB1*02:01
	CONTIG2	A*25:01	B*27:05	C*07:01	DRB1*11:01	DQB1*03:01
HC2031	CONTIG1	A*02:01	B*07:02	C*04:01	DRB1*13:01	DQB1*06:02
	CONTIG2	A*11:01	B*35:01	C*07:02	DRB1*15:01	DQB1*06:03
HC2032	CONTIG1	A*01:01	B*07:02	C*07:01	DRB1*03:01	DQB1*02:01
	CONTIG2	A*02:01	B*08:01	C*07:02	DRB1*15:01	DQB1*06:02
HC2033	CONTIG1	A*02:01	B*14:01	C*03:03	DRB1*04:01	DQB1*02:02
	CONTIG2	A*26:01	B*15:01	C*08:02	DRB1*07:01	DQB1*03:02
HC2034	CONTIG1	A*01:01	B*08:01	C*02:02	DRB1*03:01	DQB1*02:01
	CONTIG2	A*02:01	B*27:02	C*07:01	DRB1*15:01	DQB1*06:02
HC2035	CONTIG1	A*25:01	B*18:01	C*03:04	DRB1*15:01	DQB1*06:02
	CONTIG2	A*31:01	B*40:01	C*12:03	-	-
HC2036	CONTIG1	A*02:01	B*44:02	C*04:01	DRB1*04:01	DQB1*03:01
	CONTIG2	A*68:02	B*53:01	C*05:01	DRB1*13:02	DQB1*06:04
HC2037	CONTIG1	A*02:01	B*15:01	C*04:01	DRB1*12:01	DQB1*03:01
	CONTIG2	A*31:01	B*39:01	C*12:03	DRB1*13:01	DQB1*06:03
HC2038	CONTIG1	A*01:01	B*08:01	C*07:01	DRB1*03:01	DQB1*02:01
	CONTIG2	A*24:02	B*41:02	C*17:03	DRB1*13:03	DQB1*03:01
HC2039	CONTIG1	A*02:01	B*07:02	C*06:02	DRB1*07:01	DQB1*02:02
	CONTIG2	-	B*13:02	C*07:02	DRB1*15:01	DQB1*06:02
HC2040	CONTIG1	A*02:01	B*27:05	C*02:02	DRB1*03:01	DQB1*02:01
	CONTIG2	A*24:02	B*44:05	-	DRB1*16:01	DQB1*05:02
HC2041	CONTIG1	A*02:01	B*27:05	C*01:02	DRB1*01:01	DQB1*05:01
	CONTIG2	A*03:01	B*35:01	C*04:01	-	-
HC2042	CONTIG1	A*24:02	B*38:01	C*06:02	DRB1*04:03	DQB1*03:02
	CONTIG2	A*25:01	B*57:01	C*12:03	DRB1*07:01	DQB1*03:03
HC2043	CONTIG1	A*25:01	B*18:01	C*01:02	DRB1*04:01	DQB1*03:02
	CONTIG2	A*26:01	B*27:05	C*12:03	DRB1*09:01	DQB1*03:03
HC2044	CONTIG1	A*02:01	B*15:01	C*03:03	DRB1*07:01	DQB1*02:02
	CONTIG2	A*68:01	B*38:01	C*12:03	DRB1*13:01	DQB1*06:03
HC2045	CONTIG1	A*02:01	B*13:02	C*06:02	DRB1*07:01	DQB1*02:02
	CONTIG2	-	B*57:01	-	-	DQB1*03:03
HC2046	CONTIG1	A*02:01	B*15:01	C*01:02	DRB1*03:01	DQB1*02:01
	CONTIG2	A*30:02	B*18:01	C*05:01	DRB1*07:01	DQB1*02:02
HC2047	CONTIG1	A*01:01	B*37:01	C*03:03	DRB1*15:01	DQB1*06:02
	CONTIG2	A*11:01	B*44:02	C*06:02	-	-
HC2048	CONTIG1	A*03:01	B*07:02	C*07:02	DRB1*13:02	DQB1*06:02
	CONTIG2	A*68:01	-	-	DRB1*15:01	DQB1*06:04
HC2049	CONTIG1	A*02:01	B*35:03	C*06:02	DRB1*07:01	DQB1*02:02
	CONTIG2	A*02:05	B*50:01	C*12:03	DRB1*14:54	DQB1*05:03
HC2050	CONTIG1	A*01:01	B*07:02	C*07:01	DRB1*10:01	DQB1*05:01

	CONTIG2	A*24:02	B*08:01	C*07:02	DRB1*15:01	DQB1*06:02
HC2051	CONTIG1	A*01:01	B*44:03	C*04:01	DRB1*07:01	DQB1*03:01
	CONTIG2	A*24:02	B*57:01	C*06:02	DRB1*11:01	DQB1*03:03
HC2052	CONTIG1	A*03:01	B*35:01	C*04:01	DRB1*01:01	DQB1*03:01
	CONTIG2	A*11:01	B*56:01	-	DRB1*04:01	DQB1*05:01
HC2053	CONTIG1	A*11:01	B*18:01	C*01:02	DRB1*01:01	DQB1*03:02
	CONTIG2	A*25:01	B*56:01	C*12:03	DRB1*04:01	DQB1*05:01
HC2054	CONTIG1	A*03:01	B*07:02	C*07:02	DRB1*11:03	DQB1*03:01
	CONTIG2	A*26:01	B*38:01	C*12:03	DRB1*16:01	DQB1*05:02
HC2055	CONTIG1	A*02:01	B*13:02	C*02:02	DRB1*07:01	DQB1*02:02
	CONTIG2	A*24:02	B*27:02	C*06:02	DRB1*16:01	DQB1*05:02
HC2056	CONTIG1	A*03:01	B*08:01	C*07:01	DRB1*11:04	DQB1*03:01
	CONTIG2	A*68:01	B*51:01	C*12:03	DRB1*15:01	DQB1*06:02
HC2057	CONTIG1	A*02:01	B*35:03	C*07:02	DRB1*04:08	DQB1*03:01
	CONTIG2	A*03:01	B*39:01	C*12:03	DRB1*11:03	DQB1*03:04
HC2058	CONTIG1	A*01:01	B*08:01	C*03:04	DRB1*11:01	DQB1*03:01
	CONTIG2	A*24:02	B*40:01	C*07:01	DRB1*13:02	DQB1*06:04
HC2059	CONTIG1	A*24:02	B*38:01	C*12:02	DRB1*01:01	DQB1*02:01
	CONTIG2	A*26:01	B*52:01	C*12:03	DRB1*03:01	DQB1*05:01
HC2060	CONTIG1	A*01:01	B*18:01	C*06:02	DRB1*07:01	DQB1*03:03
	CONTIG2	A*03:01	B*57:01	C*12:03	DRB1*15:01	DQB1*06:02
HC2061	CONTIG1	A*11:01	B*35:01	C*04:01	DRB1*01:01	DQB1*02:02
	CONTIG2	A*29:02	B*44:03	C*16:01	DRB1*07:01	DQB1*05:01
HC2062	CONTIG1	A*01:01	B*38:01	C*06:02	DRB1*08:01	DQB1*04:02
	CONTIG2	A*02:01	B*39:06	C*12:03	DRB1*13:01	DQB1*06:03
HC2063	CONTIG1	A*02:01	B*08:01	C*04:01	DRB1*07:01	DQB1*03:03
	CONTIG2	A*11:01	B*51:01	C*07:01	DRB1*13:01	DQB1*06:03
HC2064	CONTIG1	A*24:02	B*27:02	C*02:02	DRB1*11:01	DQB1*03:01
	CONTIG2	A*66:01	B*41:02	C*17:03	DRB1*16:01	DQB1*05:02
HC2065	CONTIG1	A*02:01	B*18:01	C*07:01	DRB1*11:01	DQB1*03:01
	CONTIG2	A*32:01	B*39:01	C*12:03	DRB1*16:01	DQB1*05:02
HC2066	CONTIG1	A*02:01	B*35:08	C*04:01	DRB1*11:01	DQB1*03:01
	CONTIG2	A*03:01	B*45:01	C*06:02	DRB1*11:02	DQB1*03:19
HC2067	CONTIG1	A*02:01	B*38:01	C*12:03	DRB1*01:01	DQB1*03:01
	CONTIG2	A*66:01	B*51:01	C*14:02	DRB1*11:01	DQB1*05:01
HC2068	CONTIG1	A*02:05	B*07:02	C*06:02	DRB1*13:02	DQB1*05:02
	CONTIG2	A*03:01	B*50:01	C*07:02	DRB1*16:01	DQB1*06:04
HC2069	CONTIG1	A*03:01	B*44:02	C*05:01	DRB1*04:01	DQB1*03:01
	CONTIG2	A*68:01	B*51:01	C*07:02	DRB1*16:01	DQB1*05:02
HC2070	CONTIG1	A*02:17	B*44:02	C*03:03	DRB1*01:01	DQB1*05:01
	CONTIG2	A*26:01	B*55:01	C*05:01	DRB1*13:01	DQB1*06:03
HC2071	CONTIG1	A*02:01	B*07:02	C*06:02	DRB1*07:01	DQB1*02:02
	CONTIG2	A*24:02	B*13:02	C*07:02	DRB1*11:04	DQB1*03:01
HC2072	CONTIG1	A*02:01	B*08:01	C*07:01	DRB1*03:01	DQB1*02:01
	CONTIG2	A*24:02	B*18:01	-	DRB1*11:04	DQB1*03:01
HC2073	CONTIG1	A*01:01	B*08:01	C*07:01	DRB1*03:01	DQB1*02:01
	CONTIG2	A*02:01	B*18:01	C*12:03	DRB1*15:01	DQB1*06:02
HC2074	CONTIG1	A*02:01	B*15:01	C*03:04	DRB1*13:01	DQB1*06:02
	CONTIG2	A*31:01	B*18:01	C*12:03	DRB1*15:01	DQB1*06:03
HC2075	CONTIG1	A*02:01	B*40:02	C*02:02	DRB1*11:03	DQB1*03:01

	CONTIG2	A*03:01	B*42:05	C*07:02	DRB1*15:01	DQB1*06:02
HC2076	CONTIG1	A*02:01	B*44:02	C*05:01	DRB1*04:01	DQB1*03:02
	CONTIG2	A*26:01	B*57:01	C*06:02	DRB1*07:01	DQB1*03:03
HC2077	CONTIG1	A*02:01	B*44:02	C*02:02	DRB1*03:01	DQB1*02:01
	CONTIG2	A*24:02	B*44:05	C*07:04	DRB1*11:01	DQB1*03:01
HC2078	CONTIG1	A*01:01	B*15:01	C*03:03	DRB1*07:01	DQB1*02:02
	CONTIG2	A*23:01	B*44:03	C*04:01	-	-
HC2079	CONTIG1	A*02:01	B*07:02	C*01:02	DRB1*01:01	DQB1*03:02
	CONTIG2	A*11:01	B*27:05	C*07:02	DRB1*04:01	DQB1*05:01
HC2080	CONTIG1	A*02:01	B*07:02	C*07:02	DRB1*07:01	DQB1*03:03
	CONTIG2	A*25:01	B*18:01	C*12:03	DRB1*10:01	DQB1*05:01
HC2081	CONTIG1	A*01:01	B*39:01	C*07:02	DRB1*01:01	DQB1*03:01
	CONTIG2	A*03:01	B*56:01	C*12:03	DRB1*12:01	DQB1*05:01
HC2082	CONTIG1	A*02:01	B*27:05	C*02:02	DRB1*04:07	DQB1*03:01
	CONTIG2	A*03:01	B*44:02	C*05:01	DRB1*13:01	DQB1*06:03
HC2083	CONTIG1	A*03:01	B*35:03	C*12:03	DRB1*14:04	DQB1*05:03
	CONTIG2	A*32:01	B*40:02	C*15:02	DRB1*14:54	-
HC2084	CONTIG1	A*01:01	B*08:01	C*03:04	DRB1*03:01	DQB1*02:01
	CONTIG2	A*25:01	B*15:01	C*07:01	DRB1*04:01	DQB1*03:02
HC2085	CONTIG1	A*02:01	B*18:01	C*07:01	DRB1*11:01	DQB1*03:01
	CONTIG2	-	B*39:01	C*12:03	DRB1*11:04	-
HC2086	CONTIG1	A*01:01	B*18:01	C*07:01	DRB1*13:01	DQB1*03:01
	CONTIG2	A*66:01	B*41:02	C*17:03	DRB1*13:03	DQB1*06:03
HC2087	CONTIG1	A*02:01	B*07:02	C*07:02	DRB1*15:01	DQB1*06:02
	CONTIG2	A*24:02	-	-	-	-
HC2088	CONTIG1	A*02:01	B*44:02	C*05:01	DRB1*09:01	DQB1*03:01
	CONTIG2	A*24:02	B*48:01	C*08:03	DRB1*12:01	DQB1*03:03
HC2089	CONTIG1	A*02:01	B*07:02	C*06:02	DRB1*07:01	DQB1*03:01
	CONTIG2	A*03:01	B*13:02	C*07:02	DRB1*11:01	DQB1*03:03
HC2090	CONTIG1	A*01:01	B*07:02	C*07:02	DRB1*01:01	DQB1*03:01
	CONTIG2	A*11:01	B*51:01	C*12:03	DRB1*11:04	DQB1*05:01
HC2091	CONTIG1	A*11:01	B*07:02	C*07:01	DRB1*03:01	DQB1*02:01
	CONTIG2	A*31:01	B*08:01	C*07:02	DRB1*04:05	DQB1*02:02
HC2092	CONTIG1	A*01:01	B*08:01	C*04:01	DRB1*11:01	DQB1*03:01
	CONTIG2	A*11:01	B*35:01	C*07:01	DRB1*14:54	DQB1*05:03
HC2093	CONTIG1	A*02:01	B*18:01	C*07:01	DRB1*11:04	DQB1*03:01
	CONTIG2	-	-	-	-	-
HC2094	CONTIG1	A*02:01	B*07:02	C*07:02	DRB1*11:01	DQB1*03:01
	CONTIG2	A*03:01	B*44:27	C*07:04	DRB1*16:01	DQB1*05:02
HC2095	CONTIG1	A*11:01	B*18:01	C*12:02	DRB1*01:01	DQB1*03:02
	CONTIG2	A*25:01	B*52:01	C*12:03	DRB1*04:01	DQB1*05:01
HC2096	CONTIG1	A*02:01	B*07:05	C*05:01	DRB1*10:01	DQB1*03:01
	CONTIG2	A*30:01	B*44:02	C*15:05	DRB1*12:01	DQB1*05:01
HC2097	CONTIG1	A*24:02	B*15:17	C*04:01	DRB1*01:01	DQB1*05:01
	CONTIG2	A*68:01	B*35:01	C*07:01	DRB1*15:02	DQB1*06:01
HC2098	CONTIG1	A*02:01	B*35:01	C*04:01	DRB1*01:01	DQB1*05:01
	CONTIG2	A*03:01	B*44:02	C*05:01	DRB1*13:02	DQB1*06:04
HC2099	CONTIG1	A*02:01	B*27:02	C*02:02	DRB1*01:01	DQB1*05:01
	CONTIG2	A*03:01	B*27:05	-	DRB1*16:01	DQB1*05:02
HC2100	CONTIG1	A*02:01	B*07:02	C*07:02	DRB1*03:01	DQB1*02:01

	CONTIG2	-	B*51:01	C*16:02	DRB1*04:08i	DQB1*03:04
HC2101	CONTIG1	A*02:01	B*27:02	C*02:02	DRB1*04:01	DQB1*03:01
	CONTIG2	A*03:01	B*40:02	C*03:04	DRB1*16:01	DQB1*05:02
HC2102	CONTIG1	A*03:01	B*18:01	C*12:03	DRB1*15:01	DQB1*05:02
	CONTIG2	A*25:01	B*39:05	-	DRB1*16:01	DQB1*06:02
HC2103	CONTIG1	A*02:01	B*50:01	C*06:02	DRB1*07:01	DQB1*02:02
	CONTIG2	A*02:05	B*57:01	-	-	DQB1*03:03
HC2104	CONTIG1	A*01:01	B*07:02	C*06:02	DRB1*12:01	DQB1*03:01
	CONTIG2	A*24:02	B*57:01	C*07:02	DRB1*15:01	DQB1*06:02
HC2105	CONTIG1	A*01:01	B*08:01	C*02:02	DRB1*04:01	DQB1*02:02
	CONTIG2	A*11:01	B*27:02	C*07:01	DRB1*07:01	DQB1*03:02
HC2106	CONTIG1	A*02:01	B*07:02	C*05:01	DRB1*15:01	DQB1*06:02
	CONTIG2	A*03:01	B*44:02	C*07:02	-	-
HC2107	CONTIG1	A*01:01	B*56:01	C*01:02	DRB1*01:01	DQB1*02:02
	CONTIG2	A*25:01	B*57:01	C*06:02	DRB1*07:01	DQB1*05:01
HC2108	CONTIG1	A*02:01	B*15:01	C*03:03	DRB1*13:01	DQB1*06:03
	CONTIG2	A*24:02	B*15:125	-	-	-
HC2109	CONTIG1	A*24:02	B*18:01	C*02:02	DRB1*01:02	DQB1*03:01
	CONTIG2	A*32:01	B*40:02	C*12:03	DRB1*11:04	DQB1*05:01
HC2110	CONTIG1	A*02:01	B*07:02	C*03:04	DRB1*04:04	DQB1*03:02
	CONTIG2	A*11:01	B*15:01	C*07:02	DRB1*13:01	DQB1*06:03
HC2111	CONTIG1	A*03:01	B*14:01	C*03:04	DRB1*04:04	DQB1*03:02
	CONTIG2	A*34:02	B*40:01	C*08:02	DRB1*04:05	-
HC2112	CONTIG1	A*02:01	B*27:02	C*02:02	DRB1*04:01	DQB1*03:01
	CONTIG2	A*03:01	B*40:02	C*03:04	DRB1*16:01	DQB1*05:02
HC2113	CONTIG1	A*02:01	B*15:01	C*02:02	DRB1*09:01	DQB1*03:03
	CONTIG2	A*68:01	B*27:05	C*03:04	DRB1*16:01	DQB1*05:02
HC2114	CONTIG1	A*23:01	B*18:01	C*04:01	DRB1*01:01	DQB1*05:01
	CONTIG2	A*24:02	B*44:03	C*12:03	DRB1*15:01	DQB1*06:02
HC2115	CONTIG1	A*01:01	B*08:01	C*03:03	DRB1*01:01	DQB1*02:01
	CONTIG2	A*32:01	B*35:01	C*07:01	DRB1*03:01	DQB1*05:01
HC2116	CONTIG1	A*02:01	B*15:01	C*02:02	DRB1*13:01	DQB1*06:02
	CONTIG2	-	B*27:02	C*03:03	DRB1*15:01	DQB1*06:03
HC2117	CONTIG1	A*11:01	B*07:02	C*04:01	DRB1*01:01	DQB1*05:01
	CONTIG2	A*24:02	B*35:01	C*07:02	DRB1*14:54	DQB1*05:03
HC2118	CONTIG1	A*02:01	B*18:01	C*04:01	DRB1*01:01	DQB1*03:01
	CONTIG2	A*03:01	B*35:01	C*07:01	DRB1*11:04	DQB1*05:01
HC2119	CONTIG1	A*02:01	B*44:02	C*05:01	DRB1*04:04	DQB1*03:02
	CONTIG2	-	B*44:27	C*07:04	DRB1*13:02	DQB1*06:04
HC2120	CONTIG1	A*25:01	B*15:01	C*03:03	DRB1*04:01	DQB1*03:02
	CONTIG2	A*26:01	B*18:01	C*12:03	DRB1*13:01	DQB1*06:03
HC2121	CONTIG1	A*01:01	B*07:02	C*07:02	DRB1*01:01	DQB1*05:01
	CONTIG2	A*25:01	B*18:01	C*12:03	DRB1*15:01	DQB1*06:02
HC2122	CONTIG1	A*02:01	B*07:02	C*07:02	DRB1*07:01	DQB1*02:02
	CONTIG2	-	B*39:01	C*12:03	DRB1*12:01	DQB1*03:01
HC2123	CONTIG1	A*01:01	B*07:02	C*02:02	DRB1*13:01	DQB1*06:02
	CONTIG2	A*25:01	B*27:05	C*07:02	DRB1*15:01	DQB1*06:03
HC2124	CONTIG1	A*01:01	B*08:01	C*03:04	DRB1*04:01	DQB1*02:02
	CONTIG2	A*68:01	B*15:01	C*07:01	DRB1*07:01	DQB1*03:02
HC2125	CONTIG1	A*01:01	B*08:01	C*02:02	DRB1*03:01	DQB1*02:01

	CONTIG2	A*32:01	B*27:05	C*07:01	DRB1*09:01	DQB1*03:03
HC2126	CONTIG1	A*11:01	B*27:05	C*01:02	DRB1*01:01	DQB1*05:01
	CONTIG2	A*68:01	B*52:01	C*12:02	DRB1*15:02	DQB1*06:01
HC2127	CONTIG1	A*01:01	B*08:01	C*07:01	DRB1*03:01	DQB1*02:01
	CONTIG2	A*02:01	B*51:01	C*15:02	DRB1*04:04	DQB1*03:02
HC2128	CONTIG1	A*26:01	B*18:01	C*03:04	DRB1*04:03	DQB1*03:02
	CONTIG2	-	B*40:01	C*12:03	DRB1*15:01	DQB1*06:03
HC2129	CONTIG1	A*02:01	B*07:02	C*02:02	DRB1*04:03	DQB1*03:01
	CONTIG2	A*24:02	B*40:02	C*07:02	DRB1*11:01	DQB1*03:02
HC2130	CONTIG1	A*25:01	B*15:03	C*02:10	DRB1*13:03	DQB1*03:01
	CONTIG2	A*29:02	B*18:01	C*12:03	DRB1*15:01	DQB1*06:02
HC2131	CONTIG1	A*02:01	B*13:02	C*06:02	DRB1*07:01	DQB1*02:02
	CONTIG2	A*24:03	B*51:01	C*15:04	DRB1*10:01	DQB1*05:01
HC2132	CONTIG1	A*02:01	B*08:01	C*03:03	DRB1*03:01	DQB1*02:01
	CONTIG2	A*03:01	B*55:01	C*07:01	DRB1*11:03	DQB1*03:01
HC2133	CONTIG1	A*24:02	B*18:01	C*01:02	DRB1*14:54	DQB1*05:03
	CONTIG2	-	B*51:01	C*07:01	DRB1*15:01	DQB1*06:02
HC2134	CONTIG1	A*01:01	B*08:01	C*03:03	DRB1*11:03	DQB1*03:01
	CONTIG2	A*68:02	B*44:02	C*07:01	DRB1*12:01	-
HC2135	CONTIG1	A*01:01	B*13:02	C*06:02	DRB1*07:01	DQB1*03:03
	CONTIG2	A*02:06	B*57:01	-	DRB1*13:01	DQB1*06:03
HC2136	CONTIG1	A*02:01	B*13:02	C*04:01	DRB1*11:01	DQB1*03:01
	CONTIG2	A*68:01	B*35:03	C*06:02	DRB1*11:04	-
HC2137	CONTIG1	A*01:01	B*07:02	C*07:01	DRB1*03:01	DQB1*02:01
	CONTIG2	A*03:01	B*08:01	C*07:02	DRB1*04:01	DQB1*03:02
HC2138	CONTIG1	A*02:01	B*13:02	C*06:02	DRB1*07:01	DQB1*02:02
	CONTIG2	A*25:01	B*18:01	C*12:03	DRB1*08:03	DQB1*03:01
HC2139	CONTIG1	A*23:01	B*35:01	C*04:01	DRB1*11:01	DQB1*03:01
	CONTIG2	A*24:02	B*49:01	C*07:01	DRB1*11:03	-
HC2140	CONTIG1	A*02:01	B*44:02	C*01:02	DRB1*04:03	DQB1*03:05
	CONTIG2	-	B*51:01	C*05:01	DRB1*13:02	DQB1*06:09
HC2141	CONTIG1	A*03:01	B*07:02	C*04:01	DRB1*01:01	DQB1*02:02
	CONTIG2	A*23:01	B*44:03	C*07:02	DRB1*07:01	DQB1*05:01
HC2142	CONTIG1	A*11:01	B*38:01	C*12:03	DRB1*14:01	DQB1*05:03
	CONTIG2	A*32:01	B*51:01	C*15:02	DRB1*15:01	DQB1*06:02
HC2143	CONTIG1	A*02:01	B*13:02	C*02:02	DRB1*04:04	DQB1*03:01
	CONTIG2	A*03:01	B*40:02	C*06:02	DRB1*12:01	DQB1*03:02
HC2144	CONTIG1	A*01:01	B*08:01	C*06:02	DRB1*03:01	DQB1*02:01
	CONTIG2	A*02:01	B*57:01	C*07:01	DRB1*13:02	DQB1*06:04
HC2145	CONTIG1	A*02:01	B*15:01	C*03:04	DRB1*01:01	DQB1*03:01
	CONTIG2	-	B*40:01	-	DRB1*12:01	DQB1*05:01
HC2146	CONTIG1	A*02:01	B*15:01	C*01:02	DRB1*08:01	DQB1*04:02
	CONTIG2	A*03:01	B*51:01	C*04:01	DRB1*15:01	DQB1*06:02
HC2147	CONTIG1	A*11:01	B*44:02	C*07:04	DRB1*11:01	DQB1*03:01
	CONTIG2	A*68:01	B*52:01	C*12:02	DRB1*15:02	DQB1*06:01
HC2148	CONTIG1	A*02:01	B*07:02	C*04:01	DRB1*01:01	DQB1*03:01
	CONTIG2	A*23:01	B*44:03	C*07:02	DRB1*07:01	DQB1*05:01
HC2149	CONTIG1	A*02:01	B*44:05	C*02:02	DRB1*01:01	DQB1*02:02
	CONTIG2	-	B*50:01	C*06:02	DRB1*07:01	DQB1*05:01
HC2150	CONTIG1	A*03:01	B*35:01	C*01:02	DRB1*11:01	DQB1*03:01

	CONTIG2	A*11:01	B*51:01	C*04:01	DRB1*13:01	DQB1*06:03
HC2151	CONTIG1	A*02:01	B*07:02	C*07:02	DRB1*04:04	DQB1*03:02
	CONTIG2	-	B*51:01	C*15:02	DRB1*13:01	DQB1*06:03
HC2152	CONTIG1	A*01:01	B*08:01	C*06:02	DRB1*03:01	DQB1*02:01
	CONTIG2	A*32:01	B*57:01	C*07:01	DRB1*07:01	DQB1*03:03
HC2153	CONTIG1	A*23:01	B*38:01	C*12:03	DRB1*07:01	DQB1*02:02
	CONTIG2	A*29:02	B*44:03	C*16:01	DRB1*13:01	DQB1*06:03
HC2154	CONTIG1	A*01:01	B*13:02	C*06:02	DRB1*07:01	DQB1*02:02
	CONTIG2	A*23:01	B*41:01	C*17:01	-	-
HC2155	CONTIG1	A*24:02	B*07:02	C*07:02	DRB1*11:01	DQB1*03:01
	CONTIG2	A*68:02	B*14:02	C*08:02	DRB1*13:03	-
HC2156	CONTIG1	A*01:01	B*07:02	C*03:04	DRB1*13:02	DQB1*06:02
	CONTIG2	A*02:01	B*40:01	C*07:02	DRB1*15:01	DQB1*06:04
HC2157	CONTIG1	A*01:01	B*35:01	C*04:01	DRB1*04:04	DQB1*03:02
	CONTIG2	A*02:01	B*57:01	C*06:02	DRB1*14:54	DQB1*05:03
HC2158	CONTIG1	A*01:01	B*08:01	C*03:03	DRB1*03:01	DQB1*02:01
	CONTIG2	A*24:02	B*15:01	C*07:01	DRB1*13:01	DQB1*06:03
HC2159	CONTIG1	A*01:01	B*07:02	C*03:04	DRB1*04:01	DQB1*03:02
	CONTIG2	A*03:01	B*40:01	C*07:02	DRB1*04:04	-
HC2160	CONTIG1	A*01:01	B*08:01	C*04:01	DRB1*03:01	DQB1*02:01
	CONTIG2	A*23:01	B*44:03	C*07:01	DRB1*07:01	DQB1*02:02
HC2161	CONTIG1	A*02:01	B*44:02	C*05:01	DRB1*04:01	DQB1*03:01
	CONTIG2	A*24:02	-	C*07:01	DRB1*11:01	-
HC2162	CONTIG1	A*02:01	B*41:02	C*02:02	DRB1*01:01	DQB1*03:01
	CONTIG2	A*66:01	B*44:05	C*17:03	DRB1*13:03	DQB1*05:01
HC2163	CONTIG1	A*02:01	B*18:01	C*07:02	DRB1*04:01	DQB1*03:01
	CONTIG2	-	B*39:01	C*12:03	DRB1*12:01	DQB1*03:02
HC2164	CONTIG1	A*25:01	B*07:02	C*06:02	DRB1*01:01	DQB1*03:03
	CONTIG2	A*26:01	B*57:01	C*07:02	DRB1*07:01	DQB1*05:01
HC2165	CONTIG1	A*01:01	B*07:02	C*02:02	DRB1*15:01	DQB1*05:02
	CONTIG2	A*02:01	B*27:02	C*07:02	DRB1*16:01	DQB1*06:02
HC2166	CONTIG1	A*24:02	B*08:01	C*07:01	DRB1*03:01	DQB1*02:01
	CONTIG2	A*25:01	B*51:01	C*15:02	DRB1*11:01	DQB1*03:01
HC2167	CONTIG1	A*02:01	B*07:02	C*03:04	DRB1*04:01	DQB1*03:02
	CONTIG2	A*03:01	B*15:01	C*07:02	DRB1*15:01	DQB1*06:02
HC2168	CONTIG1	A*24:02	B*13:02	C*03:03	DRB1*04:01	DQB1*02:02
	CONTIG2	A*30:01	B*15:01	C*06:02	DRB1*07:01	DQB1*03:02
HC2169	CONTIG1	A*02:01	B*07:04	C*07:02	DRB1*07:01	DQB1*02:02
	CONTIG2	A*25:01	B*18:01	C*12:03	DRB1*15:01	DQB1*06:02
HC2170	CONTIG1	A*03:01	B*18:01	C*04:01	DRB1*04:01	DQB1*03:02
	CONTIG2	A*25:01	B*35:03	C*12:03	DRB1*08:01	DQB1*04:02
HC2171	CONTIG1	A*02:01	B*18:01	C*07:01	DRB1*11:04	DQB1*03:01
	CONTIG2	-	B*44:02	C*16:04	-	-
HC2172	CONTIG1	A*03:01	B*35:01	C*04:01	DRB1*01:01	DQB1*03:01
	CONTIG2	A*11:01	B*38:01	-	DRB1*11:01	DQB1*05:01
HC2173	CONTIG1	A*02:01	B*18:01	C*02:02	DRB1*15:01	DQB1*05:02
	CONTIG2	A*25:01	B*27:02	C*12:03	DRB1*16:01	DQB1*06:02
HC2174	CONTIG1	A*01:01	B*08:01	C*06:02	DRB1*03:01	DQB1*02:01
	CONTIG2	A*25:01	B*13:02	C*07:01	DRB1*07:01	DQB1*02:02
HC2175	CONTIG1	A*03:01	B*35:01	C*04:01	DRB1*11:01	DQB1*03:01

	CONTIG2	A*68:01	B*44:02	C*07:04	DRB1*12:01	-
HC2176	CONTIG1	A*02:01	B*18:01	C*04:01	DRB1*07:01	DQB1*02:02
	CONTIG2	A*23:01	B*44:03	C*12:03	DRB1*15:01	DQB1*06:02
HC2177	CONTIG1	A*01:01	B*07:02	C*06:02	DRB1*07:01	DQB1*02:02
	CONTIG2	A*03:01	B*13:02	C*07:02	DRB1*15:01	DQB1*06:39
HC2178	CONTIG1	A*02:01	B*07:02	C*06:02	DRB1*07:01	DQB1*02:02
	CONTIG2	A*03:01	B*13:02	C*07:02	DRB1*16:01	DQB1*05:02
HC2179	CONTIG1	A*24:02	B*15:01	C*03:03	DRB1*11:01	DQB1*03:01
	CONTIG2	A*25:01	B*44:02	C*07:04	DRB1*16:02	DQB1*05:02
HC2180	CONTIG1	A*02:01	B*07:02	C*06:02	DRB1*08:01	DQB1*03:01
	CONTIG2	A*11:01	B*13:02	C*07:02	DRB1*11:04	DQB1*04:02
HC2181	CONTIG1	A*24:02	B*38:01	C*02:02	DRB1*11:01	DQB1*03:01
	CONTIG2	A*68:01	B*40:02	C*12:03	DRB1*11:04	-
HC2182	CONTIG1	A*02:01	B*15:01	C*01:02	DRB1*01:01	DQB1*03:03
	CONTIG2	A*26:01	B*51:01	C*03:04	DRB1*07:01	DQB1*05:01
HC2183	CONTIG1	A*02:01	B*27:05	C*02:02	DRB1*01:01	DQB1*05:01
	CONTIG2	A*32:01	B*44:03	C*04:01	DRB1*01:02	-
HC2184	CONTIG1	A*23:01	B*15:01	C*03:03	DRB1*15:01	DQB1*05:02
	CONTIG2	A*24:02	B*49:01	C*07:01	-	DQB1*06:02
HC2185	CONTIG1	A*01:01	B*35:01	C*03:04	DRB1*01:01	DQB1*03:01
	CONTIG2	A*02:01	B*40:01	C*04:01	DRB1*12:01	DQB1*05:01
HC2186	CONTIG1	A*02:01	B*35:01	C*04:01	DRB1*01:01	DQB1*05:01
	CONTIG2	A*03:01	B*44:02	C*05:01	DRB1*11:01	DQB1*05:02
HC2187	CONTIG1	A*02:01	B*27:02	C*02:02	DRB1*09:01	DQB1*03:03
	CONTIG2	-	B*44:02	C*05:01	DRB1*16:01	DQB1*05:02
HC2188	CONTIG1	A*01:01	B*15:01	C*03:03	DRB1*11:01	DQB1*03:01
	CONTIG2	A*24:02	B*57:01	C*06:02	DRB1*13:01	DQB1*06:03
HC2189	CONTIG1	A*01:01	B*07:02	C*07:01	DRB1*03:01	DQB1*02:01
	CONTIG2	A*24:02	B*08:01	C*07:02	DRB1*15:01	DQB1*06:02
HC2190	CONTIG1	A*30:02	B*18:01	C*05:01	DRB1*01:01	DQB1*05:01
	CONTIG2	A*68:01	B*52:01	C*12:02	DRB1*15:02	DQB1*06:01
HC2191	CONTIG1	A*02:01	B*07:02	C*03:03	DRB1*07:01	DQB1*03:03
	CONTIG2	A*03:01	B*15:01	C*07:02	DRB1*15:01	DQB1*06:02
HC2192	CONTIG1	A*03:01	B*13:02	C*02:02	DRB1*01:01	DQB1*02:02
	CONTIG2	A*30:01	B*27:05	C*06:02	DRB1*07:01	DQB1*05:01
HC2193	CONTIG1	A*01:01	B*08:01	C*05:01	DRB1*03:01	DQB1*02:01
	CONTIG2	A*02:01	B*44:02	C*07:01	DRB1*04:01	DQB1*03:01
HC2194	CONTIG1	A*02:01	B*27:05	C*02:02	DRB1*04:01	DQB1*03:01
	CONTIG2	A*24:02	B*44:02	C*05:01	-	DQB1*03:02
HC2195	CONTIG1	A*23:01	B*27:05	C*01:02	DRB1*01:01	DQB1*02:02
	CONTIG2	A*24:02	B*44:03	C*04:01	DRB1*07:01	DQB1*05:01
HC2196	CONTIG1	A*02:01	B*07:02	C*03:04	DRB1*04:01	DQB1*03:02
	CONTIG2	A*29:01	B*15:01	C*07:02	DRB1*15:01	DQB1*06:02
HC2197	CONTIG1	A*02:01	B*18:01	C*04:01	DRB1*11:01	DQB1*03:01
	CONTIG2	A*68:01	B*35:03	C*07:01	DRB1*11:04	-
HC2198	CONTIG1	A*11:01	B*15:01	C*03:03	DRB1*11:01	DQB1*03:01
	CONTIG2	A*23:01	B*49:01	C*07:01	DRB1*13:01	DQB1*06:03
HC2199	CONTIG1	A*02:01	B*14:01	C*05:01	DRB1*07:01	DQB1*02:02
	CONTIG2	A*26:01	B*44:02	C*08:02	DRB1*13:01	DQB1*06:03
HC2200	CONTIG1	A*02:01	B*15:01	C*02:02	DRB1*04:01	DQB1*03:01

	CONTIG2	-	B*40:02	C*03:03	DRB1*11:03	DQB1*03:02
HC2201	CONTIG1	A*01:01	B*18:01	C*05:01	DRB1*03:01	DQB1*02:01
	CONTIG2	A*02:01	B*35:03	C*12:03	DRB1*04:08	DQB1*03:04
HC2202	CONTIG1	A*03:01	B*07:02	C*04:01	DRB1*01:01	DQB1*04:02
	CONTIG2	A*11:01	B*35:01	C*07:02	DRB1*08:01	DQB1*05:01
HC2203	CONTIG1	A*25:01	B*08:01	C*07:01	DRB1*03:01	DQB1*02:01
	CONTIG2	A*68:01	B*51:01	C*15:02	DRB1*13:01	DQB1*06:03
HC2204	CONTIG1	A*02:01	B*18:01	C*02:02	DRB1*09:01	DQB1*03:01
	CONTIG2	A*26:01	B*27:05	C*07:01	DRB1*11:04	DQB1*03:03
HC2205	CONTIG1	A*01:01	B*08:01	C*06:02	DRB1*03:01	DQB1*02:01
	CONTIG2	A*02:05	B*50:01	C*07:01	DRB1*07:01	DQB1*02:02
HC2206	CONTIG1	A*24:02	B*18:01	C*12:02	DRB1*01:01	DQB1*03:02
	CONTIG2	A*25:01	B*52:01	C*12:03	DRB1*04:01	DQB1*05:01
HC2207	CONTIG1	A*02:01	B*15:220	C*04:01	DRB1*04:04	DQB1*03:02
	CONTIG2	A*24:02	B*39:01	C*07:02	DRB1*13:02	DQB1*06:04
HC2208	CONTIG1	A*24:02	B*27:02	C*02:02	DRB1*11:01	DQB1*03:01
	CONTIG2	-	B*40:02	C*03:04	DRB1*16:01	DQB1*05:02
HC2209	CONTIG1	A*32:01	B*14:02	C*05:01	DRB1*03:01	DQB1*02:01
	CONTIG2	A*33:01	B*44:02	C*08:02	DRB1*12:01	DQB1*03:01
HC2210	CONTIG1	A*03:01	B*40:01	C*03:04	DRB1*01:01	DQB1*03:01
	CONTIG2	A*24:02	B*41:02	C*17:03	DRB1*13:03	DQB1*05:01
HC2211	CONTIG1	A*02:01	B*44:02	C*05:01	DRB1*11:01	DQB1*03:01
	CONTIG2	A*25:01	-	C*07:04	DRB1*12:01	-
HC2212	CONTIG1	A*01:01	B*07:02	C*06:02	DRB1*07:01	DQB1*03:03
	CONTIG2	A*24:02	B*57:01	C*07:02	DRB1*15:01	DQB1*06:02
HC2213	CONTIG1	A*01:01	B*27:05	C*01:02	DRB1*01:01	DQB1*02:02
	CONTIG2	A*03:02	B*44:03	C*04:01	DRB1*07:01	DQB1*05:01
HC2214	CONTIG1	A*02:01	B*27:05	C*01:02	DRB1*09:01	DQB1*03:03
	CONTIG2	-	B*56:01	-	DRB1*15:01	DQB1*06:02
HC2215	CONTIG1	A*23:01	B*35:08	C*03:03	DRB1*04:03	DQB1*03:02
	CONTIG2	A*24:02	B*55:01	C*04:01	DRB1*14:54	DQB1*05:03
HC2216	CONTIG1	A*01:01	B*13:02	C*03:04	DRB1*07:01	DQB1*02:02
	CONTIG2	A*02:01	B*40:01	C*06:02	DRB1*13:01	DQB1*06:03
HC2217	CONTIG1	A*01:01	B*44:03	C*04:01	DRB1*07:01	DQB1*02:02
	CONTIG2	A*23:01	B*57:01	C*06:02	DRB1*13:01	DQB1*06:03
